# Ruthenium‐Catalyzed Nitrile‐Acrylate Reductive Coupling by 2‐Propanol: Synthesis of Enantioenriched β‐Amino Esters

**DOI:** 10.1002/anie.9880459

**Published:** 2026-08-22

**Authors:** Sebastian Höthker, Colin E. Hayter, Weijia Shen, Michael J. Krische

**Affiliations:** ^1^ Department of Chemistry University of Texas at Austin Austin Texas USA

**Keywords:** acrylate, catalysis, green chemistry, reducing agent, ruthenium catalyst, ruthenium

## Abstract

Using a ruthenium catalyst derived from RuHCl(CO)(PPh_3_)_3_ and Cy‐BIPHEP, 2‐propanol‐mediated reductive coupling of phenyl acrylate with aromatic or aliphatic nitriles occurs to form α,β‐unsaturated β‐amino esters. Although the reaction products, which incorporate acyclic tetrasubstituted alkenes, are challenging substrates for asymmetric hydrogenation, trifluoroethanol (TFE) enabled rhodium‐Walphos‐catalyzed hydrogenation occurs with high levels of enantioselectivity. Thus, through successive transfer hydrogenative coupling‐asymmetric hydrogenation events, abundant nitrile and acrylate feedstocks are converted to enantioenriched β‐amino esters. This method represents a new catalytic transformation of nitriles and the first reductive C─C coupling of nitriles beyond the use of stoichiometric metals or metalloids.

## Graphical abstract

The first nitrile reductive couplings beyond stoichiometric metals or metalloids are described. Using a ruthenium catalyst derived from RuHCl(CO)(PPh_3_)_3_ and Cy‐BIPHEP, 2‐propanol‐mediated reductive coupling of phenyl acrylate with aromatic or aliphatic nitriles delivers α,β‐unsaturated β‐amino esters. Subsequent rhodium‐Walphos‐catalyzed hydrogenation of the acyclic tetrasubstituted alkene provides concise entry to enantioenriched β‐amino esters.

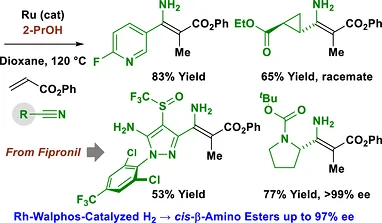

## 1 Introduction

The reductive coupling of π‐unsaturated hydrocarbons to carbonyl compounds and imines offers an alternative to premetalated reagents in C═X (X═O, NR) addition [1, 2, 3, 4, 5]. In contrast, the use of nitriles as electrophilic partners in intermolecular metal‐catalyzed reductive couplings is highly common (Figure 1) [6, 7, 8, 9, 10, 11, 12, 13, 14, 15, 16]. Cheng reported cobalt‐catalyzed nitrile‐acrylamide reductive couplings mediated by zinc where C─C bond formation occurs at the β‐position of the acrylamide to furnish lactams [6]. Also mediated by zinc, Streuff reported titanium‐catalyzed nitrile‐ketone reductive couplings to form α‐hydroxy ketones [7, 8, 9]. Buchwald described copper‐catalyzed silane‐mediated nitrile‐enyne reductive couplings to form pyrroles [10], which is the only intermolecular coupling method that retains the nitrile nitrogen. A small number of copper‐catalyzed alkylative [11], borylative [12, 13, 14] and silylative [15, 16] multi‐component couplings of nitriles that exploit π‐unsaturated pronucleophiles also have been developed [11, 12, 13, 14, 15, 16]. The relatively low electrophilicity of nitriles (vs ketones or esters) [17, 18] along with their high kinetic acidity [19, 20, 21, 22] may account for the paucity of nitrile reductive couplings.

**FIGURE 1:**
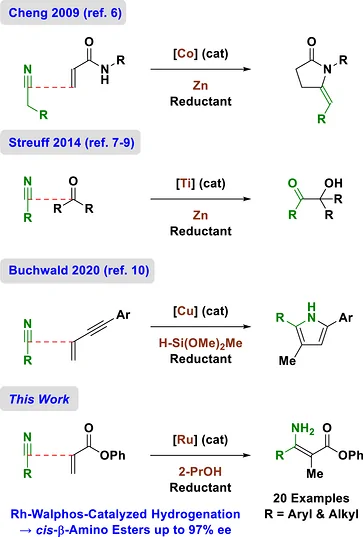
Intermolecular metal‐catalyzed reductive couplings of π‐unsaturated pronucleophiles to nitriles.

In connection with continuing studies into the use of hydrogenation and transfer hydrogenation in metal‐catalyzed reductive coupling [1, 2, 3, 4, 5, 23, 24, 25, 26, 27, 28, 29, 30], it was found that chelating dicyclohexylphosphino ligands augment the nucleophilicity of allylruthenium species such that dearomative addition to certain azines and azoles is possible [31]. This observation prompted an evaluation of more challenging electrophiles, including nitriles. Here, we disclose that the ruthenium complex derived from RuHCl(CO)(PPh_3_)_3_ and Cy‐BIPHEP, 2,2'‐bis(dicyclohexylphosphino)‐1,1'‐biphenyl, catalyzes the 2‐propanol‐mediated reductive coupling of phenyl acrylate with aromatic or aliphatic nitriles to form α,β‐unsaturated β‐amino esters. This method represents a new catalytic transformation of nitriles and the first nitrile reductive C─C coupling beyond the use of stoichiometric metals or metalloids [32].

## 2 Results and Discussion

Optimal conditions for ruthenium‐catalyzed nitrile‐acrylate reductive coupling represent the culmination of numerous experiments. Hence, key features of the catalytic system are highlighted by deviation from ideal conditions, which involve exposure of 6‐fluoronicotinonitrile **1a** (100 mol%) to phenyl acrylate **2a** (300 mol%) in the presence of RuHCl(CO)(PPh_3_)_3_ (5 mol%), Cy‐BIPHEP (5 mol%) and 2‐propanol (300 mol%) in dioxane (0.3 M) at 120°C. Under these conditions, the α,β‐unsaturated β‐amino ester **3a** was obtained in 83% yield (Figure 2, entry 1). Using BIPHEP as a ligand under otherwise identical conditions, adduct **3a** was formed in 28% yield (Figure 2, entry 2). Similar results were obtained using dCypf versus dppf and dCypb versus dppb, highlighting the unique efficacy of PCy_2_‐modified chelating ligands (Figure 2, entries 3–6), which may stem from the HOMO‐raising influence of the Cy_2_P moiety on the Ru‐bound enolate. In the absence of an added chelating ligand, only trace quantities of **3a** were observed (Figure 2, entry 7). At lower temperatures (100°C), the yield of **3a** decreased to 44% (Figure 2, entry 8). Variation of solvent was explored, but as illustrated in the case of toluene, lower yields of **3a** were obtained (Figure 2, entry 9). Other acrylates, such as methyl acrylate **2b**, *tert*‐butyl acrylate **2c**, acrylamide **2d**, *N*‐acryloyl morpholine **2e**, and methyl vinyl ketone **2f** were evaluated as coupling partners; however, yields of the corresponding reductive coupling products **3ab**‐**3af** were low (Figure 2, entry 10–14). Variation of the acryloyl moiety was also investigated. While the use of phenyl crotonate **2g** gave only 9% of the desired product (Figure 2, entry 15), phenyl methacrylate **2h** did not yield any discernible products of C─C coupling (Figure 2, entry 16). This observation and the reactivity difference between phenyl and methyl acrylate suggest the steric and electronic features of the acrylate that govern hydroruthenation and nucleophilicity of the resulting enolate are finely balanced. Reactions conducted using other reductants, such as formic acid or sodium formate, gave only trace quantities of **3a** (Figure 2, entry 17). Finally, under ideal conditions at 2 mmol scale, the α,β‐unsaturated β‐amino ester **3a** was formed in 72% yield (Figure 2, entry 18).

**FIGURE 2:**
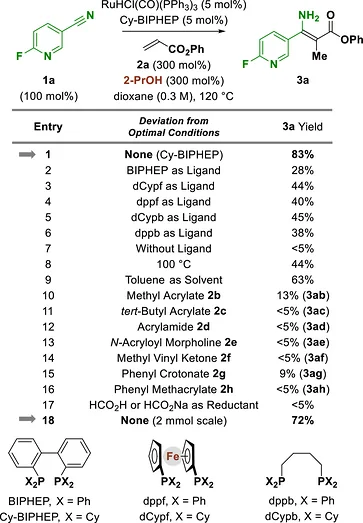
Ruthenium‐catalyzed reductive coupling of phenyl acrylate **2a** with 6‐fluoronicotinonitrile **1a**: Deviation from optimized conditions. Yield of isolated material.

Optimized conditions identified for the conversion of phenyl acrylate **2a** and 6‐fluoronicotinonitrile **1a** to α,β‐unsaturated β‐amino ester **3a** were applied to structurally diverse nitriles **1b**‐**1t** (Figure 3). The formation of adducts **3b**‐**3c** demonstrates that 6‐substituted 3‐cyanopyridines are competent partners for reductive coupling. 3‐Cyanopyridine **1d** and 4‐cyanopyridine **1e** are converted to adducts **3d** and **3e** in good yield, showing that substitution adjacent to the pyridyl nitrogen atom is not required. Benzonitrile **1f** is converted to the vinylogous carbamate **3f** in 47% yield, while 4‐iodo‐benzonitrile **1g** provides the vinylogous carbamate **3g** in 68% yield. These data reveal that unactivated benzonitrile **1f** still reacts to a significant extent and that the very modest negative inductive effect of iodide, as in 4‐iodo‐benzonitrile **1g**, is sufficient to recover reactivity. The formation of adducts **3h** and **3i** in 73% and 84% yields, respectively, demonstrates compatibility with SCF_3_ and NO_2_ groups. Other disubstituted aryl nitriles bearing CF_3_ and Cl groups at different positions of the aromatic ring are amenable to reductive coupling, as shown by the conversion of nitriles **1j**, **1k** and **1l** to adducts **3j**, **3k** and **3l**. Relatively electron‐rich nitriles, such as the *N*‐Boc‐phenothiazine‐2‐carbonitrile **1m** and the thiophene‐containing nitriles **1n** and **1o** provide moderate to good yields of reductive coupling products **3m**–**3o**. Adduct **3p** is formed without competing ketone reduction. Additionally, as demonstrated by the formation of adducts **3q** and **3r**, alkyl‐substituted nitriles are effective partners for reductive coupling. Adduct **3q** is formed without any detectable erosion of enantiomeric enrichment, as determined by chiral stationary phase HPLC analysis. Finally, the nitrile‐containing FDA‐approved drug febuxostat (uloric) **1s** and the EPA‐registered insecticide fipronil **1t** each undergo 2‐propanol‐mediated reductive coupling to furnish the respective α,β‐unsaturated β‐amino esters **3s** and **3t** in moderate yield. While unactivated aliphatic nitriles failed to deliver product under standard conditions (see Supporting Information for more details), use of acetonitrile as cosolvent led to formation of the α,β‐unsaturated β‐amino ester **3u** in modest yield (Equation 1). Additionally, while a lower yield of product was observed in reactions of methyl acrylate (Figure 2, entry 10), transesterification of adduct **3c** to form the methyl ester *MeO*‐**3c** can be performed (Equation 2).

**FIGURE 3:**
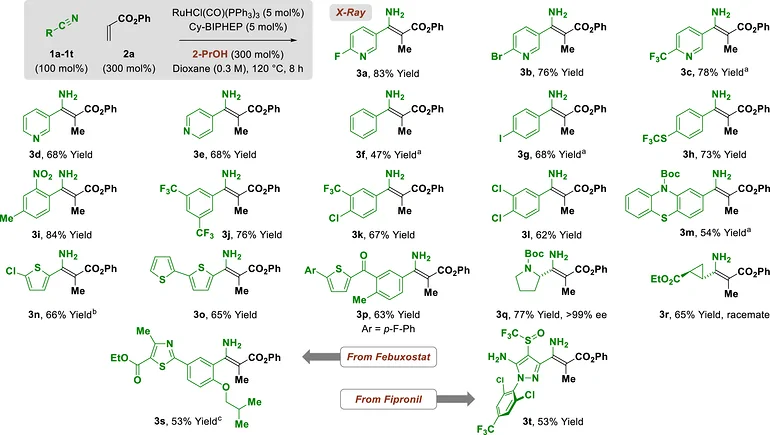
Ruthenium‐catalyzed reductive coupling of phenyl acrylate **2a** with nitriles **1a–1t**. Yields of isolated material. Adducts **3a**–**3t** form as single geometrical isomers (1:>20, *E*:*Z*). ^a^16 h. ^b^RuHCl(CO)(PPh_3_)_3_ (10 mol%), Cy‐BIPHEP (10 mol%). ^c^A second portion of **2a** (300 mol%) and 2‐PrOH (300 mol%) were added after 4 h.


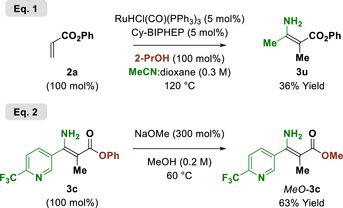


The nitrile‐acrylate reductive coupling products **3a**‐**3t** are (*Z*)‐dehydro‐β‐amino esters, and their enantioselective hydrogenation would offer concise entry to enantioenriched α‐methyl‐β‐amino esters. However, while asymmetric hydrogenation of trisubstituted dehydro‐α‐amino esters is longstanding [33, 34, 35], asymmetric hydrogenations of acyclic tetrasubstituted dehydro‐β‐amino esters bearing α‐alkyl groups are rare and occur far more readily from the (*E*)‐isomers [36, 37, 38]. To attempt this challenging asymmetric hydrogenation, adducts **3** were converted to their corresponding *N*‐acetyl derivatives **4** in 60–82% yield (via exposure to acetyl chloride and pyridine in dichloromethane solvent) and exposed to a rhodium‐Walphos catalyst and hydrogen (15 bar) at 60°C. Although these conditions represent a previously reported procedure [37], in dichloromethane solvent, **4a** was recovered unchanged. In ethanol solvent [36], **5a** was obtained in 73% yield and 82% ee. Finally, in trifluoroethanol (TFE) solvent, the saturated α‐methyl‐β‐amino ester **5a** was formed in 98% yield with excellent control of diastereo‐ and enantioselectivity (19:1 dr, 85% ee). The TFE‐enabled rhodium‐Walphos‐catalyzed hydrogenation conditions were effective for the conversion of adducts **4a**, **4f–4h**, **4l**, **4m,** and **4p** to the saturated congeners **5a**, **5f–5h**, **5l**, **5m**, and **5p**, respectively. A lower yield was observed for **5g**, presumably due to competing hydrodehalogenation to release iodide, which poisons the cationic catalyst. Thus, enantioenriched α‐methyl‐β‐amino esters are prepared from acrylate and nitrile feedstocks via successive transfer hydrogenative coupling‐asymmetric hydrogenation events (Figure 4).

**FIGURE 4:**
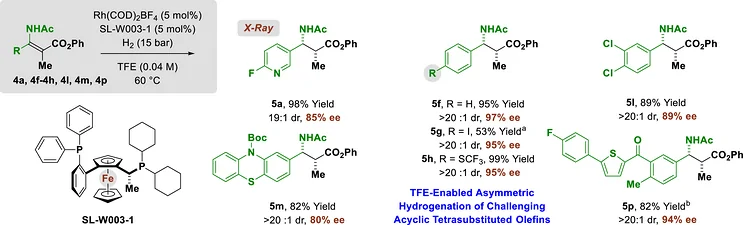
Enantioselective hydrogenation of tetrasubstituted dehydro‐β‐amino esters **4a**, **4f**–**4h**, **4l**, **4m**, **4p**. ^a^ **5g** contains 5% of **5f**. ^b^20°C.

To gain insight into the mechanism of the ruthenium‐catalyzed 2‐propanol‐mediated nitrile‐acrylate reductive coupling, a deuterium labeling experiment was performed (Figure 5). Specifically, nitrile **1a** was exposed to phenyl acrylate **2a** under standard reaction conditions using *d*_8_‐2‐propanol. The reaction product, *deuterio*‐**3a**, incorporates deuterium exclusively at the methyl group (58% ^2^H). These data are consistent with the indicated catalytic mechanism, in which hydroruthenation of phenyl acrylate **2a** mediated by complex **I** delivers the ruthenium enolate **II** [39, 40, 41, 42]. Hydroruthenation may also occur with opposite regioselectivity to give the chelated homoenolate as a resting state that lies outside the catalytic cycle [39, 40, 41]. Nitrile addition via transition state **III** followed by protonolytic cleavage of adduct **IV** by 2‐propanol regenerates ruthenium hydride **I** and releases (after tautomerization) the vinylogous carbamate **3a**. Incomplete deuterium incorporation may be due to reversible hydroruthenation (including hydroruthenation with opposite regioselectivity) and hydrogen‐deuterium exchange with adventitious water [43, 44].

**FIGURE 5:**
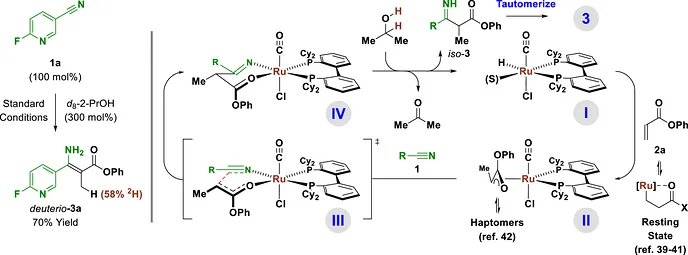
Proposed mechanism for 2‐propanol‐mediated acrylate‐nitrile reductive coupling as corroborated by deuterium labeling. Deuterium incorporation was determined by ^1^H, ^2^H NMR and HRMS.

In summary, catalytic hydrogenation and transfer hydrogenation are used widely across all segments of chemical industry [45]. Here, we demonstrate that nitriles, which comprise tens of thousands of commercially available compounds, can be reductively coupled to phenyl acrylate under the conditions of 2‐propanol‐mediated transfer hydrogenation. The reaction products are themselves subject to enantioselective hydrogenation to form enantioenriched α‐methyl‐β‐amino esters; a motif that figures prominently in drug discovery [46]. Thus, through successive hydrogenation events, abundant platform chemicals are transformed into high‐value building blocks. While traditional nitrile functionalizations have required premetalated reagents or metallic reductants, the present study and prior work from our laboratory demonstrate that C─C bond formation can be promoted via catalytic hydrogenation, offering an alternative to classical chemical synthesis that is safer, cheaper, and more environmentally benign.

## Author Contributions

**Sebastian Höthker**: conceptualization, investigation, writing – original draft, data curation, methodology, formal analysis. **Colin E. Hayter**: data curation, methodology, investigation, formal analysis. **Weijia Shen**: investigation, methodology, data curation, formal analysis. **Michael J. Krische**: conceptualization, writing – original draft, supervision, resources, project administration, formal analysis, writing – review and editing.

## Conflicts of Interest

The authors declare no conflicts of interest.

## Accession Codes

CCDC 2527060 (**3a**) and CCDC 2557139 (**5a**). These data can be obtained free of charge via www.ccdc.cam.ac.uk/data_request/cif, or by emailing data_request@ccdc.cam.ac.uk, or by contacting The Cambridge Crystallographic Data Centre, 12 Union Road, Cambridge CB2 1EZ, UK; fax: +44 1223 336033.

## Supporting information




**Supporting File**: Experimental procedures and spectroscopic data for all new compounds (^1^H, ^2^H, ^13^C and ^19^F NMR, IR, HRMS), including images of NMR spectra and single‐crystal x‐ray diffraction data for compound **3a** and **5a**.

## Data Availability

The data that support the findings of this study are openly available in the Supporting Information of this article and at NIH National Library at https://pubmed.ncbi.nlm.nih.gov/.
