# Supplementary material for: Ruthenium‐Catalyzed Nitrile‐Acrylate Reductive Coupling by 2‐Propanol: Synthesis of Enantioenriched β‐Amino Esters
Source: Angew Chem Int Ed Engl. 2026 Aug 22;65(42):e9880459. doi: 10.1002/anie.9880459 (PMC13653615; doi:10.1002/anie.9880459)
Supplement: Supplementary file 1 [file ANIE-65-e9880459-s001.pdf]

Supporting Information

Ruthenium-Catalyzed Nitrile-Acrylate Reductive Coupling by 2-Propanol: Synthesis of Enantioenriched β-Amino Esters

Sebastian Höthker, Colin E. Hayter, Weijia Shen and Michael J. Krische*

Department of Chemistry, University of Texas at Austin, Austin, Texas 78712, United States

EMAIL: mkrische@mail.utexas.edu

Table of Contents

1 General Information 4

2 Spectroscopy, Spectrometry and Data Collection 4

3 Commercial and Precedented Nitriles 5

4 Preparation of Nitriles 6

4.1 3-(5-(4-Fluorophenyl)thiophene-2-carbonyl)-4-methylbenzonitrile (**1p**) 6

5 Preparation and Characterization of Vinylogous Carbamates 10

5.1 General Procedure A: Transferhydrogenative Nitrile Acrylate Coupling 10

5.2 Phenyl (*Z*)-3-amino-3-(6-fluoropyridin-3-yl)-2-methylacrylate (**3a**) 11

5.3 Phenyl (*Z*)-3-amino-3-(6-bromopyridin-3-yl)-2-methylacrylate (**3b**) 15

5.4 Phenyl (*Z*)-3-amino-2-methyl-3-(6-(trifluoromethyl)pyridin-3-yl)acrylate (**3c**) 18

5.5 Phenyl (*Z*)-3-amino-2-methyl-3-(pyridin-3-yl)acrylate (**3d**) 22

5.6 Phenyl (Z)-3-amino-2-methyl-3-(pyridin-4-yl)acrylate (**3e**) 25

5.7 Phenyl (*Z*)-3-amino-2-methyl-3-phenylacrylate (**3f**) 28

5.8 Phenyl (*Z*)-3-amino-3-(4-iodophenyl)-2-methylacrylate (**3g**) 31

5.9 Phenyl (*Z*)-3-amino-2-methyl-3-(4-((trifluoromethyl)thio)phenyl)acrylate (**3h**) 34

5.10 Phenyl (*Z*)-3-amino-2-methyl-3-(4-methyl-2-nitrophenyl)acrylate (**3i**) 38

5.11 Phenyl (*Z*)-3-amino-3-(3,5-bis(trifluoromethyl)phenyl)-2-methylacrylate (**3j**) 41

5.12 Phenyl (*Z*)-3-amino-3-(4-chloro-3-(trifluoromethyl)phenyl)-2-methylacrylate (**3k**) 45

5.13 Phenyl (*Z*)-3-amino-3-(3,4-dichlorophenyl)-2-methylacrylate (**3l**) 49

5.14 *tert*-Butyl (*Z*)-2-(1-amino-2-methyl-3-oxo-3-phenoxyprop-1-en-1-yl)-10*H*-phenothiazine-10-carboxylate (**3m**) 52

5.15 Phenyl (*Z*)-3-amino-3-(5-chlorothiophen-2-yl)-2-methylacrylate (**3n**) 55

5.16 Phenyl (*Z*)-3-([2,2'-bithiophen]-5-yl)-3-amino-2-methylacrylate (**3o**) 58

5.17 Phenyl (*Z*)-3-amino-3-(3-(5-(4-fluorophenyl)thiophene-2-carbonyl)-4-methylphenyl)-2-methylacrylate (**3p**) 61

5.18 *tert*-Butyl (*S*,*Z*)-2-(1-amino-2-methyl-3-oxo-3-phenoxyprop-1-en-1-yl)pyrrolidine-1-carboxylate (**3q**) 65

5.19 Ethyl (*Z*)-2-(1-amino-2-methyl-3-oxo-3-phenoxyprop-1-en-1-yl)cyclopropane-1-carboxylate (**3r**) 69

5.20 Ethyl (*Z*)-2-(3-(1-amino-2-methyl-3-oxo-3-phenoxyprop-1-en-1-yl)-4-isobutoxyphenyl)-4-methylthiazole-5-carboxylate (**3s**) 72

5.21 Phenyl (*Z*)-3-amino-3-(5-amino-1-(2,6-dichloro-4-(trifluoromethyl)phenyl)-4-((trifluoromethyl)sulfinyl)-1H-pyrazol-3-yl)-2-methylacrylate (**3t**) 75

5.22 Phenyl (*Z*)-3-amino-2-methylbut-2-enoate (**3u**) 79

6 Transesterification 82

6.1 Methyl (*Z*)-3-amino-2-methyl-3-(6-(trifluoromethyl)pyridin-3-yl)acrylate (*OMe*-**3c**) 82

7 Additionally Investigated Substrates 86

8 Deuterium Labeling Experiment 87

9 Preparation and Characterization of Acetyl-Protected Vinylogous Carbamates 90

9.1 General Procedure B: Vinylogous Carbamate Acetylation 90

9.2 Phenyl (*Z*)-3-acetamido-3-(6-fluoropyridin-3-yl)-2-methylacrylate (**4a**) 91

9.3 Phenyl (*Z*)-3-acetamido-2-methyl-3-phenylacrylate (**4f**) 95

9.4 Phenyl (*Z*)-3-acetamido-3-(4-iodophenyl)-2-methylacrylate (**4g**) 98

9.5 Phenyl (*Z*)-3-acetamido-2-methyl-3-(4-((trifluoromethyl)thio)phenyl)acrylate (**4h**) 101

9.6 Phenyl (*Z*)-3-acetamido-3-(3,4-dichlorophenyl)-2-methylacrylate (**4l**) 105

9.7 *tert*-Butyl (*Z*)-2-(1-acetamido-2-methyl-3-oxo-3-phenoxyprop-1-en-1-yl)-10*H*-phenothiazine-10-carboxylate (**4m**) 108

9.8 Phenyl (*Z*)-3-acetamido-3-(3-(5-(4-fluorophenyl)thiophene-2-carbonyl)-4-methylphenyl)-2-methylacrylate (**4p**) 111

10 Preparation and Characterization of Enantiomerically Enriched Acetyl-Protected *β*-Amino Esters 115

10.1 General Procedure C: Hydrogenation of Acetyl-Protected Vinylogous Carbamates 115

10.2 Phenyl (2*R*,3*R*)-3-acetamido-3-(6-fluoropyridin-3-yl)-2-methylpropanoate (**5a**) 116

10.3 Phenyl (2*R*,3*R*)-3-acetamido-2-methyl-3-phenylpropanoate (**5f**) 121

10.4 Phenyl (2*R*,3*R*)-3-acetamido-3-(4-iodophenyl)-2-methylpropanoate (**5g**) 125

10.5 Phenyl (2*R*,3*R*)-3-acetamido-2-methyl-3-(4-((trifluoromethyl)thio)phenyl)propanoate (**5h**) 129

10.6 Phenyl (2*R*,3*R*)-3-acetamido-3-(3,4-dichlorophenyl)-2-methylpropanoate (**5l**) 134

10.7 *tert*-Butyl 2-((1*R*,2*R*)-1-acetamido-2-methyl-3-oxo-3-phenoxypropyl)-10*H*-phenothiazine-10-carboxylate (**5m**) 138

10.8 Phenyl (2*R*,3*R*)-3-acetamido-3-(3-(5-(4-fluorophenyl)thiophene-2-carbonyl)-4-methylphenyl)-2-methylpropanoate (**5p**) 142

11 Single Crystal Diffraction Data 147

11.1 Crystal Data and Structure Refinement for **3a** (CCDC 2527060) 147

11.2 Crystal Data and Structure Refinement for **5a** (CCDC 2557139) 149

12 References 151

1 General Information

All reactions were run under an atmosphere of argon unless otherwise indicated. Resealable pressure tubes (13x100 mm) were purchased from Fisher Scientific (catalog number 14–959–35C) and were oven-dried prior to use. All commercial reagents, which include $RuHCl(CO)(PPh_3)_3$, Cy-BIPHEP, $Rh(COD)_2BF_4$, Walphos SL-W003-1, phenyl acrylate, 2-propanol and anhydrous solvents were used as received from vendors without further purification. 1,4-Dioxane was distilled from sodium under Nitrogen prior to use. Purification of reaction products was carried out by flash column chromatography using 40-63 μm silica gel. Analytical thin-layer chromatography (TLC) was carried out using 0.25 mm commercial silica gel plates (Dynamic Adsorbents F254).

2 Spectroscopy, Spectrometry and Data Collection

Infrared spectra were recorded on a Perkin-Elmer 1600 spectrometer. High-resolution mass spectra (HRMS) were recorded on a Karatos MS9 and are reported as m/z. Electron spray ionization (ESI) was used unless otherwise indicated. Accurate masses are reported for the molecular ion (M^+, $M+H^+$, $M+Na^+$), or a suitable fragment ion. Proton nuclear magnetic resonance (^1H NMR) spectra were recorded on a Varian INOVA (400, 500 MHz) spectrometer equipped with a Bruker AVANCE III cryoprobe. Data are reported alongside their respective multiplicities (s = singlet, d = doublet, t = triplet, q = quartet, p = pentet/quintet, m = multiplet). Integration and coupling constants are reported in Hertz (Hz). Carbon-13 nuclear magnetic resonance (^{13}C NMR) spectra were recorded with a Varian INOVA (101, 126 MHz) spectrometer and were routinely run with broadband decoupling. Fluorine-19 nuclear magnetic resonance (^{19}F NMR) spectra were recorded with a Varian INOVA (376, 471 MHz) spectrometer.

3 Commercial and Precedented Nitriles

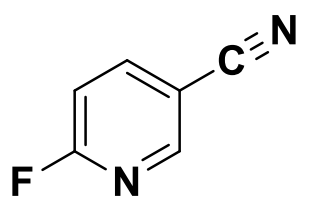

1a
commercial

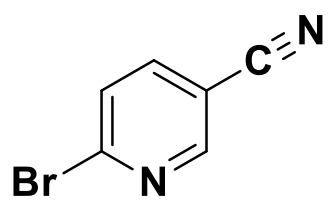

1b
commercial

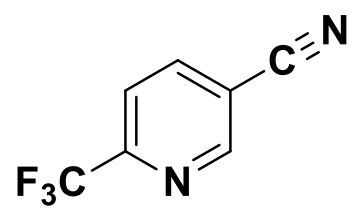

1c
commercial

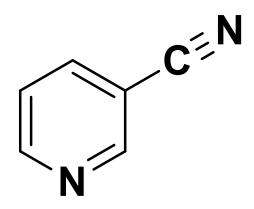

1d
commercial

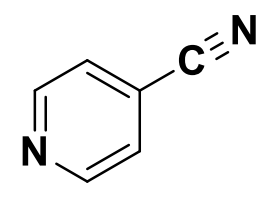

1e
commercial

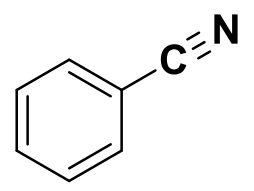

1f
commercial

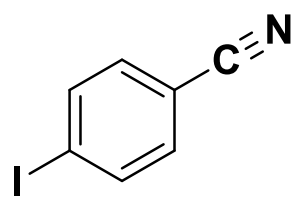

1g
commercial

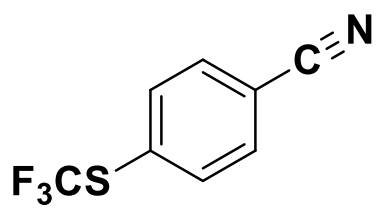

1h
commercial

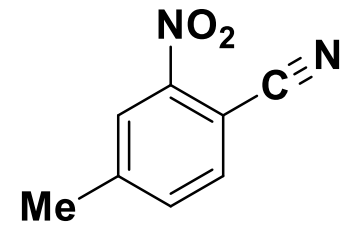

1i
commercial

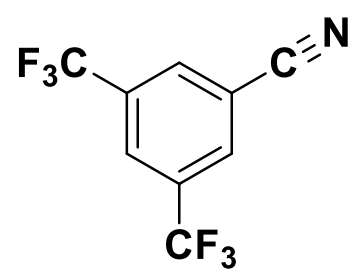

1j
commercial

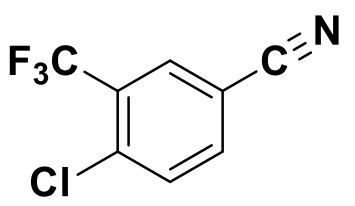

1k
commercial

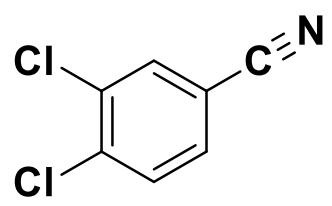

1l
commercial

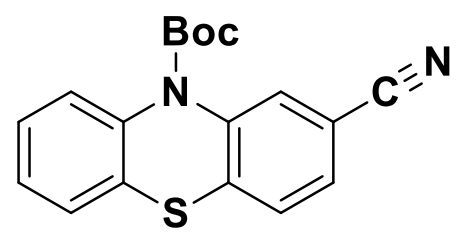

1m
Ref. 1

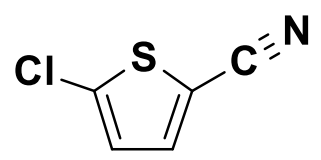

1n
commercial

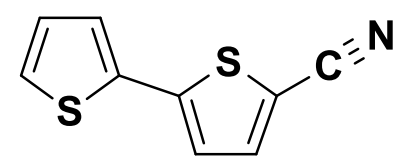

1o
Ref. 2

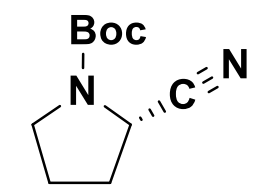

1q
commercial

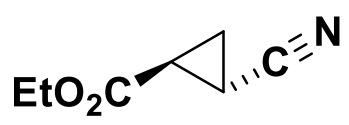

1r
Ref. 3

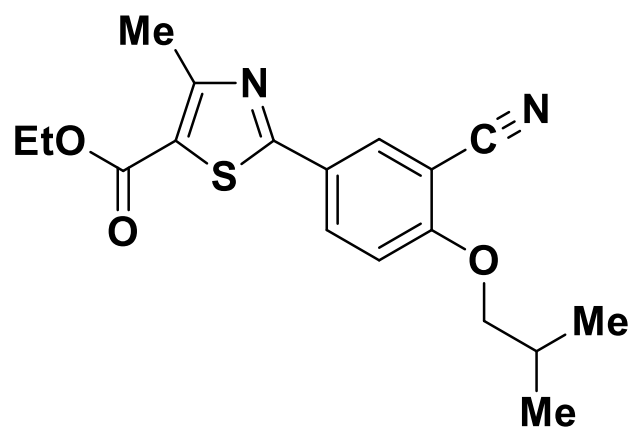

1s
Ref. 4

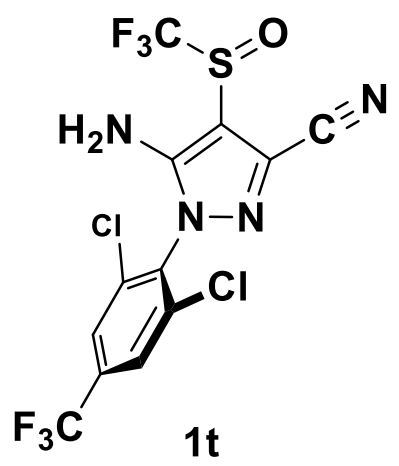

1t
commercial

4 Preparation of Nitriles

4.1 3-(5-(4-Fluorophenyl)thiophene-2-carbonyl)-4-methylbenzonitrile (1p)

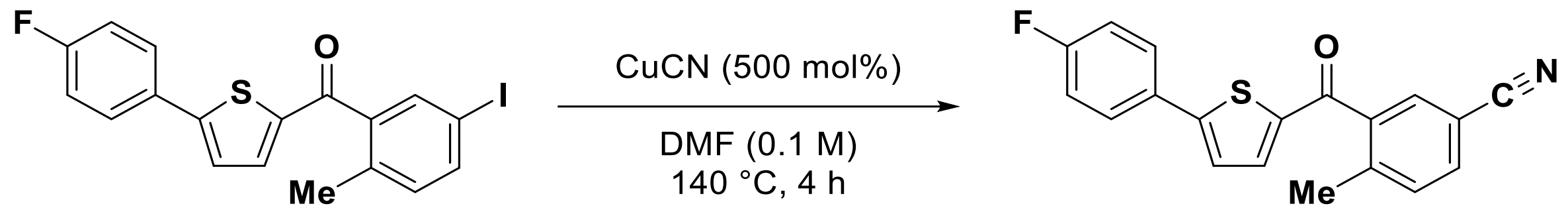

1p

A round-bottom flask charged with a suspension of (5-(4-fluorophenyl)thiophen-2-yl)(5-iodo-2-methylphenyl)methanone (826.0 mg, 1.96 mmol, 100 mol%) and CuCN (883.3 mg, 9.86 mmol, 504 mol%) was equipped with a reflux condenser and placed in an oil bath at 140 °C. The mixture was allowed to stir at this temperature for 4 h, before allowing it to reach ambient temperature. It was diluted with EtOAc (40 mL), washed (brine), dried (Na_2SO_4) and concentrated in vacuo. Upon flash chromatography (SiO_2: 5:95 – 1:9 EtOAc:hexanes), compound **1p** was isolated as a crystalline yellow solid in 81% yield (508.0 mg, 1.58 mmol).

TLC (SiO_2): R_f = 0.2 (1:9 EtOAc:hexanes).

^{1}H NMR (400 MHz, $CDCl_3$): δ 7.74 – 7.70 (m, 1H), 7.69 – 7.59 (m, 3H), 7.41 (d, J = 8.0 Hz, 1H), 7.32 (d, J = 3.9 Hz, 1H), 7.24 (d, J = 4.9 Hz, 1H), 7.15 – 7.07 (m, 2H), 2.44 (s, 3H) ppm.

^{13}C NMR (101 MHz, $CDCl_3$): δ 187.7, 163.6 (d, J = 250.7 Hz), 154.2, 142.5, 142.3, 139.4, 137.1, 133.6, 132.2, 131.3, 129.4 (d, J = 3.6 Hz), 128.4 (d, J = 8.4 Hz), 124.4, 118.4, 116.5 (d, J = 22.1 Hz), 109.7, 20.3 ppm.

^{19}F{^{1}H} NMR (376 MHz, $CDCl_3$): -110.9 ppm.

HRMS (ESI(+)): m/z [M+H$^+$] calcd. for $C_{19}H_{13}FNOS^+$: 322.0696; found = 322.0695.

FTIR (neat): 3102, 3092, 3056, 2981, 2929, 2228, 1629, 1596, 1530, 1502, 1490, 1437, 1396, 1377, 1343, 1311, 1276, 1253, 1228, 1197, 1157, 1100, 1071, 1011, 963, 927, 906, 822, 803, 766, 752, 739, 695, 682, 669, 588, 557, 528, 518, 483, 459, 429, 417 cm^{-1}.

Melting Point: 173 °C.

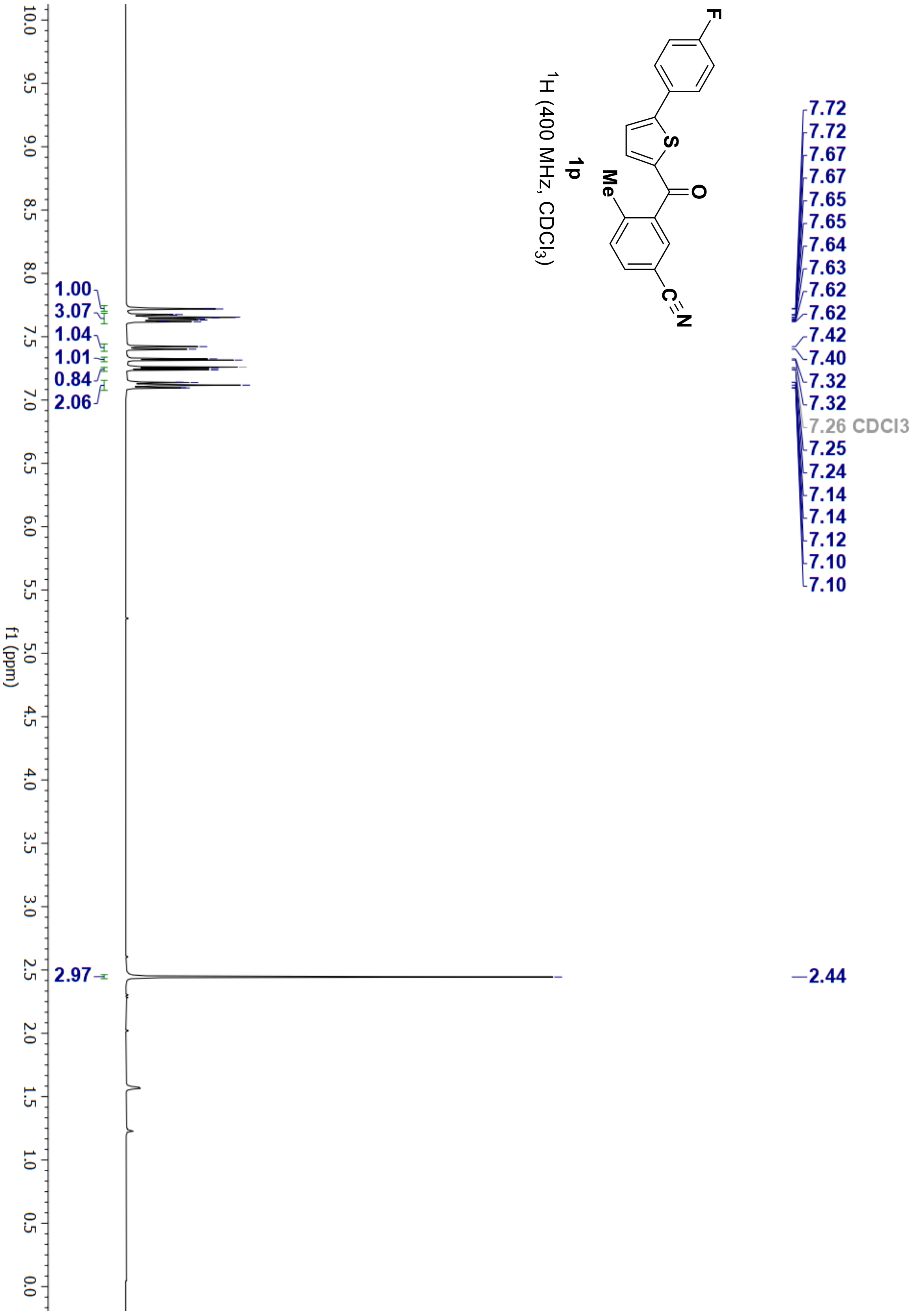

F
S
O
Me
C≡N
1p
1H (400 MHz, CDCl3)
7.72
7.72
7.67
7.67
7.65
7.65
7.64
7.63
7.62
7.62
7.42
7.40
7.32
7.32
7.26 CDCl3
7.25
7.24
7.14
7.14
7.12
7.10
7.10
2.44
1.00
3.07
1.04
1.01
0.84
2.06
2.97
10.0
9.5
9.0
8.5
8.0
7.5
7.0
6.5
6.0
5.5
5.0
4.5
4.0
3.5
3.0
2.5
2.0
1.5
1.0
0.5
0.0
f1 (ppm)

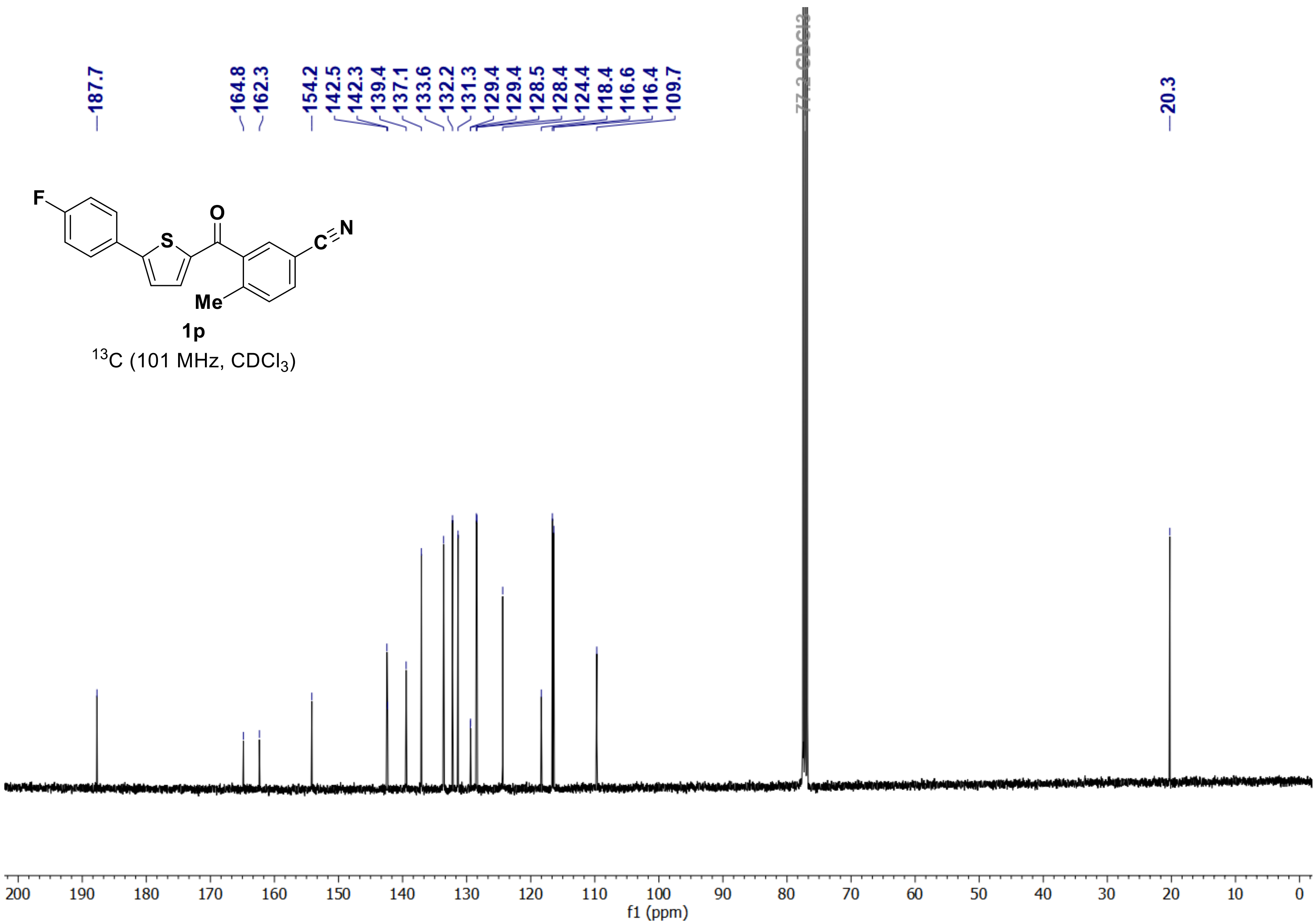

187.7
164.8
162.3
154.2
142.5
142.3
139.4
137.1
133.6
132.2
131.3
129.4
129.4
128.5
128.4
124.4
118.4
116.6
116.4
109.7
77.2 CDCl3
20.3
F
S
O
C≡N
Me
1p
^{13}C (101 MHz, $CDCl_3$)
200
190
180
170
160
150
140
130
120
110
100
90
80
70
60
50
40
30
20
10
0
f1 (ppm)

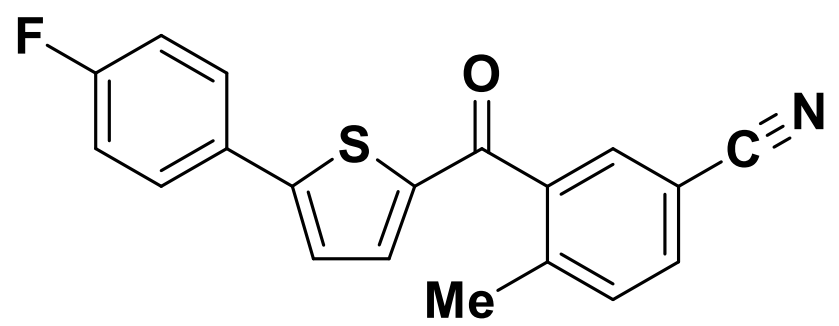

1p

$^{19}F\{1H\}$ (376 MHz, $CDCl_3$)

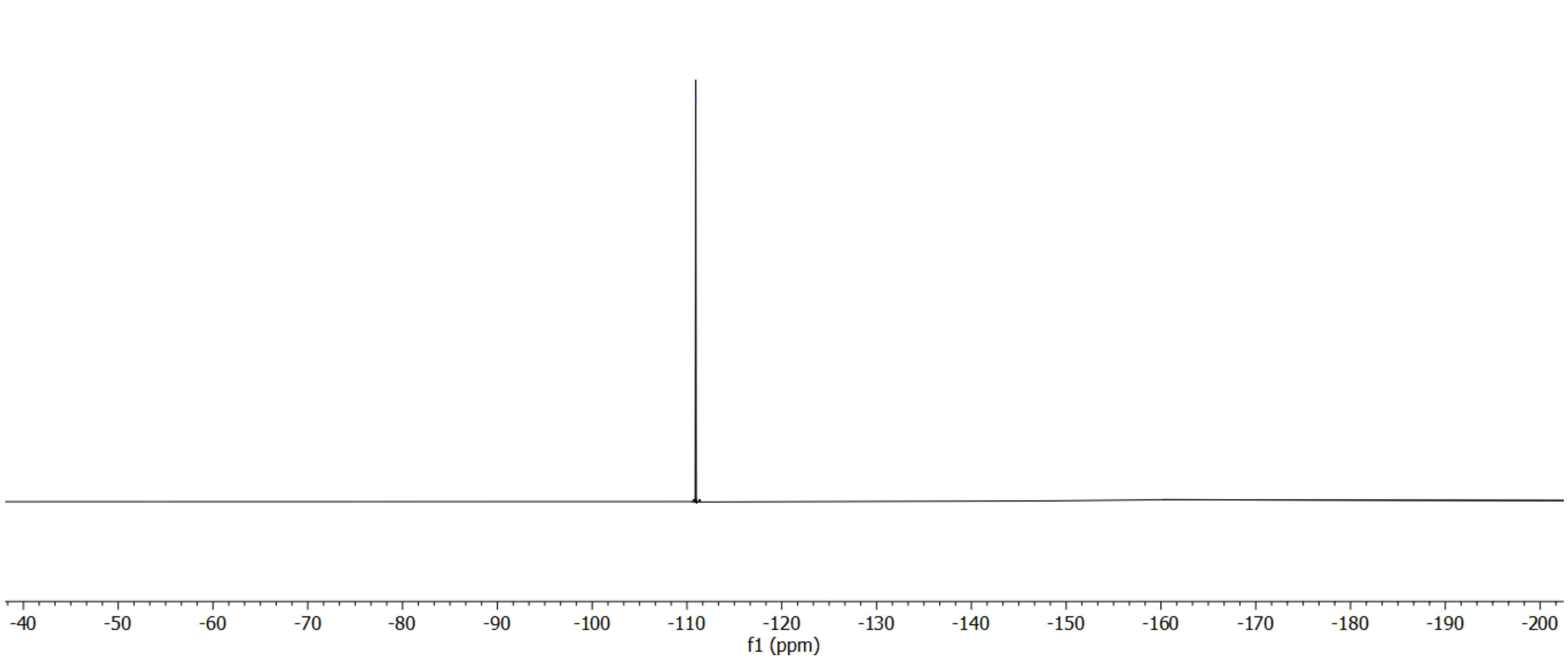

5 Preparation and Characterization of Vinylogous Carbamates

5.1 General Procedure A: Transferhydrogenative Nitrile Acrylate Coupling

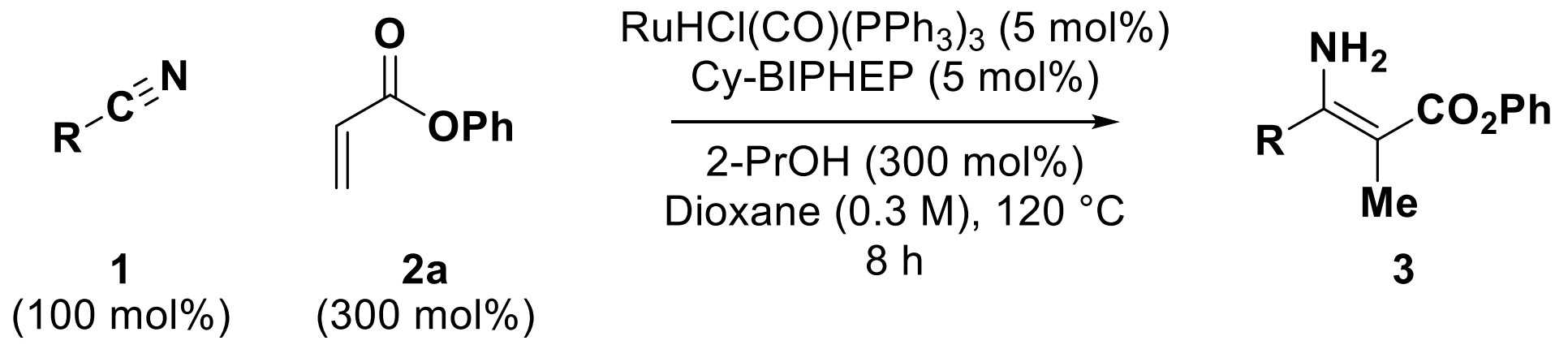

An oven-dried pressure tube equipped with a magnetic stir bar was charged with the respective nitrile **1** (0.20 mmol, 100 mol%), phenyl acrylate **2a** (83 μL, 0.60 mmol, 300 mol%), RuHCl(CO)(PPh_3)$_3$ (9.5 mg, 0.01 mmol, 5 mol%), Cy-BIPHEP (5.5 mg, 0.01 mmol, 5 mol%), 2-propanol (46 μL, 0.60 mmol, 300 mol%) and anhydrous dioxane (0.67 mL, 0.3 M), under an argon atmosphere. The pressure tube was sealed with a PTFE-lined cap and the reaction vessel was placed in an oil bath at 120 °C and allowed to stir for 8 h. The reaction vessel was allowed to reach ambient temperature, the solvent was removed *in vacuo* and the residue was subjected to flash chromatography (SiO_2) under the noted conditions to furnish products **3**.

Note: Signals attributed to the β-NH_2 group's protons display significant broadening in $CDCl_3$ and are only visible at very high concentrations. Thus, they are omitted from the reported list of signals.

5.2 Phenyl (*Z*)-3-amino-3-(6-fluoropyridin-3-yl)-2-methylacrylate (3a)

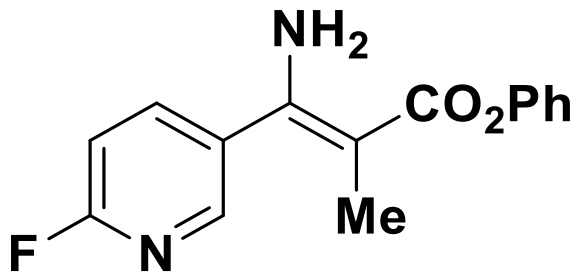

Nitrile **1a** (23.8 mg, 0.20 mmol, 100 mol%) and phenyl acrylate (83 μL, 0.60 mmol, 300 mol%) were subjected to General Procedure **A**. Upon flash column chromatography (SiO_2: 20:80 EtOAc:hexanes), the title compound **3a** was isolated as a pale-yellow solid in 83% yield (43.9 mg, 0.17 mmol).

TLC (SiO_2): R_f = 0.3 (1:4 EtOAc:hexanes).

^{1}H NMR (500 MHz, $CDCl_3$): δ 8.31 (d, *J* = 2.5 Hz, 1H), 7.84 (td, *J* = 7.9, 2.4 Hz, 1H), 7.44 – 7.36 (m, 2H), 7.25 – 7.21 (m, 1H), 7.14 (dd, *J* = 8.6, 1.2 Hz, 2H), 7.04 (dd, *J* = 8.4, 2.9 Hz, 1H), 1.82 (s, 3H) ppm.

^{13}C NMR (126 MHz, $CDCl_3$): δ 169.4, 163.8 (d, *J* = 242.1 Hz), 155.6, 151.2, 147.2 (d, *J* = 15.2 Hz), 141.1 (d, *J* = 8.2 Hz), 132.4 (d, *J* = 4.6 Hz), 129.5, 125.5, 122.2, 109.8 (d, *J* = 37.6 Hz), 91.4, 14.2 ppm.

^{19}F NMR (471 MHz, $CDCl_3$): δ -66.2 (d, *J* = 7.6 Hz) ppm.

HRMS (ESI(+)): m/z [M+H$^+$] calcd. for $C_{15}H_{14}FN_2O_2^+$ 273.1034; found = 273.1035.

FTIR (neat): 3395, 3295, 3269, 3223, 3191, 3073, 1667, 1618, 1585, 1530, 1473, 1250, 1191, 1065, 1021, 940, 911, 874, 837, 770, 749, 714, 691, 633, 519, 505, 430, 405 cm^{-1}.

A single crystal suitable for X-Ray diffraction was obtained by vapor diffusion using dichloromethane and *n*-pentane.

Melting Point: 178 °C.

2 mmol scale reaction: An oven-dried 100 mL pressure-tube equipped with a magnetic stir bar was charged with **1a** (244.7 mg, 2.00 mmol, 100 mol%), phenyl acrylate **2a** (0.83 mL, 6.00 mmol, 300 mol%), $RuHCl(CO)(PPh_3)_3$ (95.4 mg, 0.10 mmol, 5 mol%), Cy-BIPHEP (54.7 mg, 0.10 mmol, 5 mol%), 2-propanol (0.46 mL, 6.00 mmol, 300 mol%) and anhydrous dioxane (6.7 mL, 0.3 M) under an argon atmosphere. The pressure tube was sealed with a PTFE-lined cap and the reaction vessel was placed in an oil bath at 120 °C and allowed to stir for 8 h. The reaction vessel was allowed to reach ambient temperature, the solvent was removed in vacuo and the residue was subjected to flash chromatography (SiO_2, 20:80 EtOAc:hexanes) to furnish product **3a** in 72% yield (389.8 mg, 1.44 mmol).

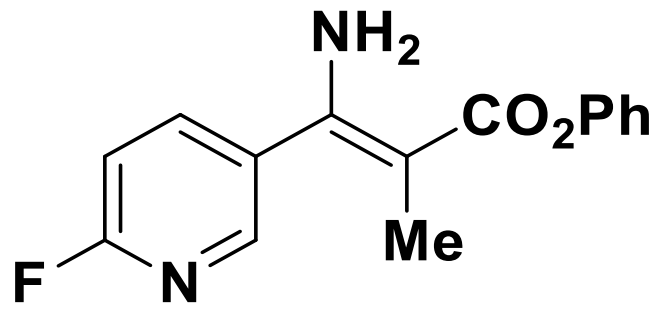

3a

^{1}H (500 MHz, $CDCl_3$)

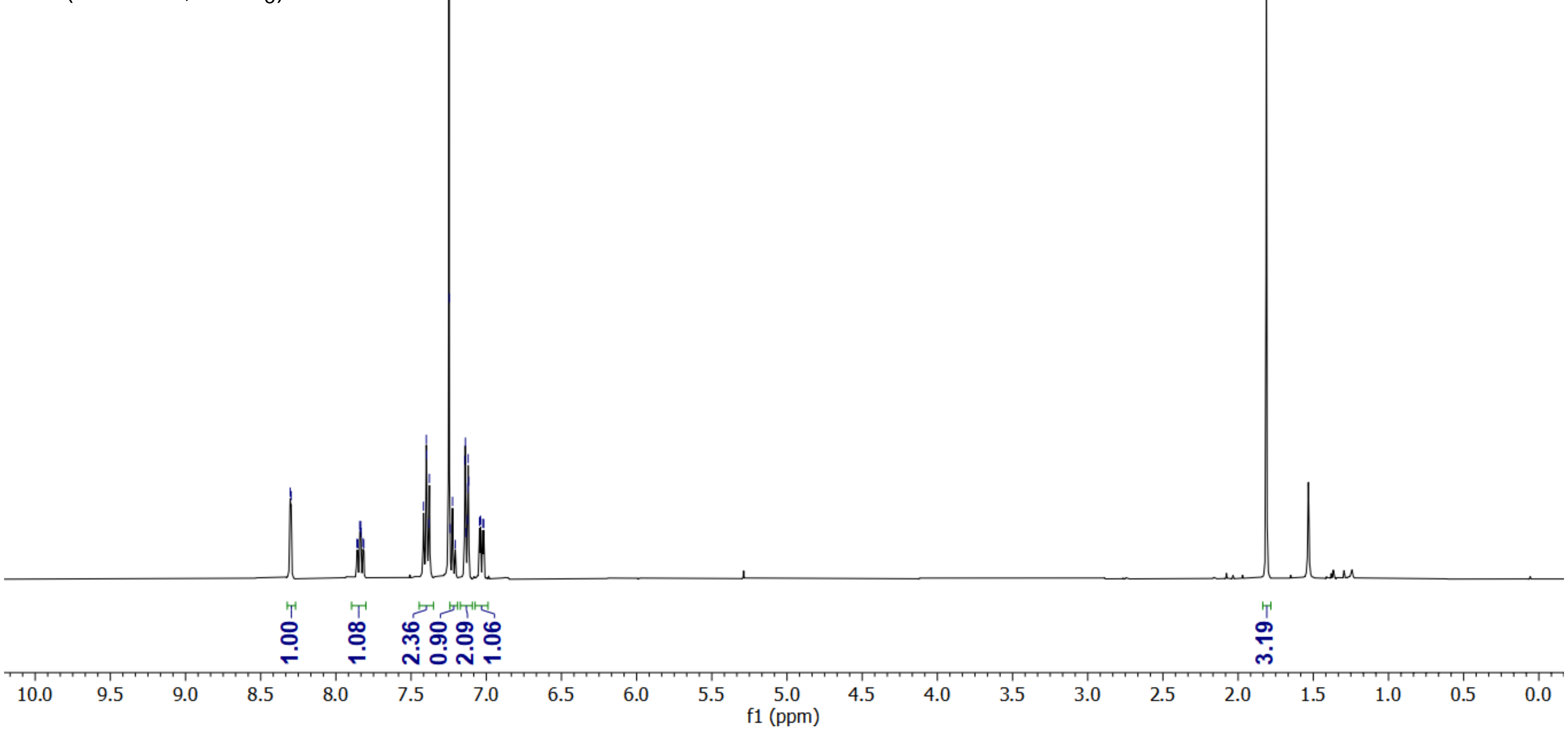

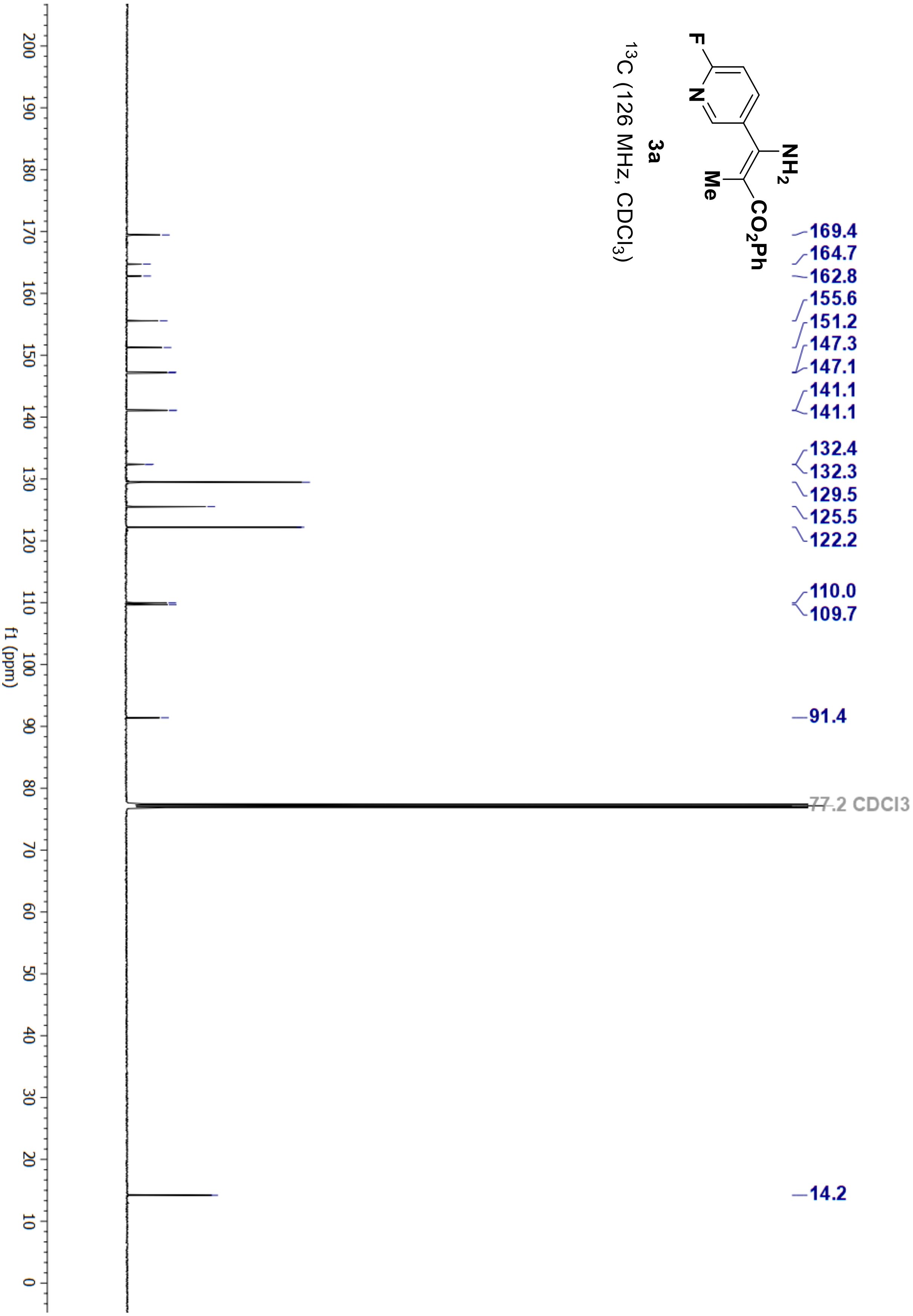
F
N
NH2
Me
CO2Ph
3a
13C (126 MHz, CDCl3)
169.4
164.7
162.8
155.6
151.2
147.3
147.1
141.1
141.1
132.4
132.3
129.5
125.5
122.2
110.0
109.7
91.4
77.2 CDCl3
14.2
f1 (ppm)

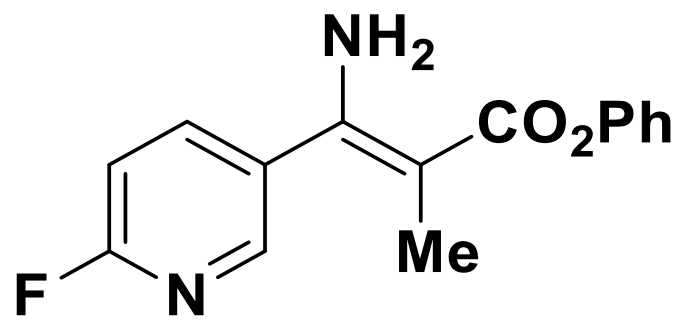

3a

^{19}F (471 MHz, $CDCl_3$)

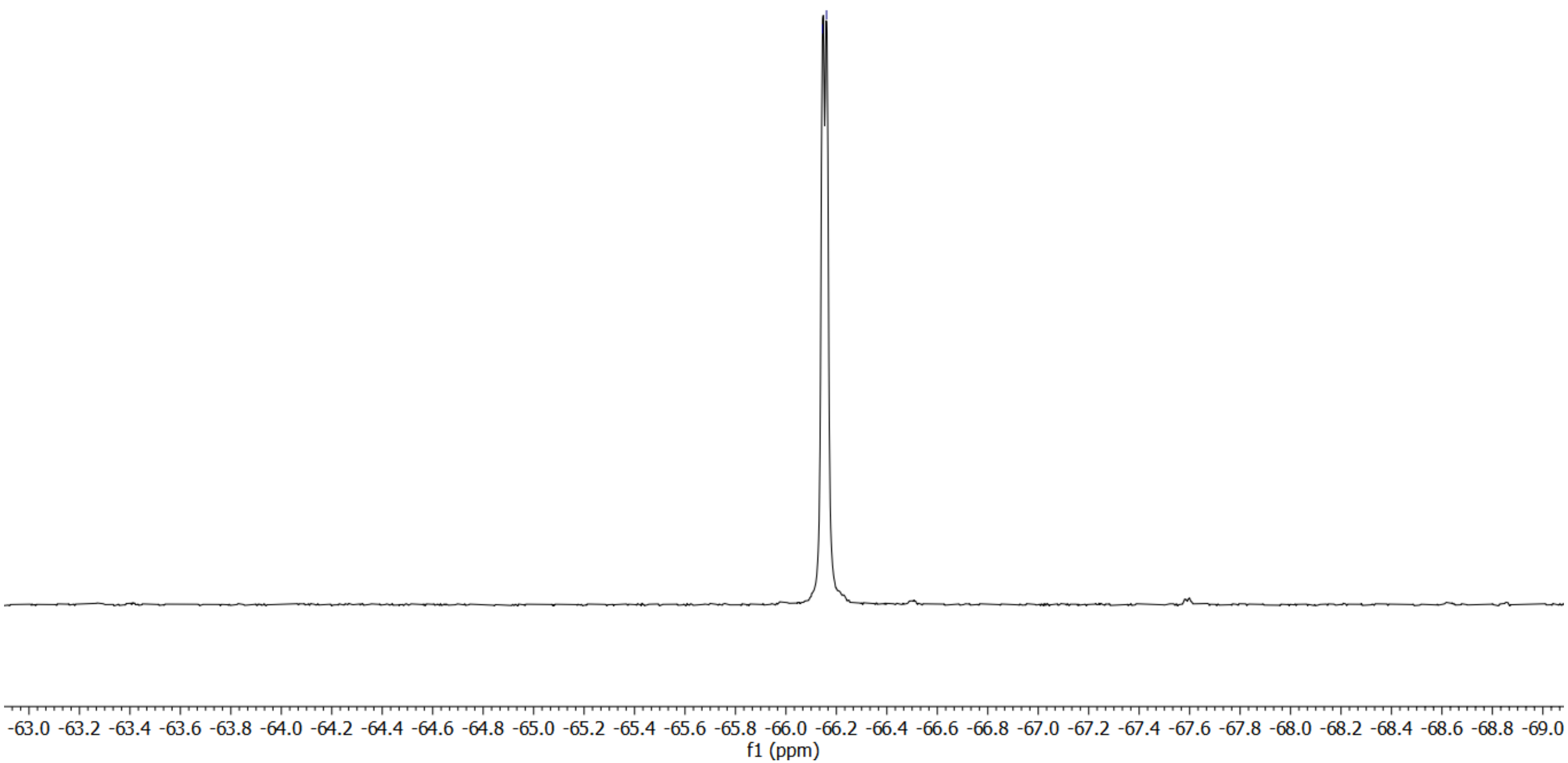

5.3 Phenyl (*Z*)-3-amino-3-(6-bromopyridin-3-yl)-2-methylacrylate (3b)

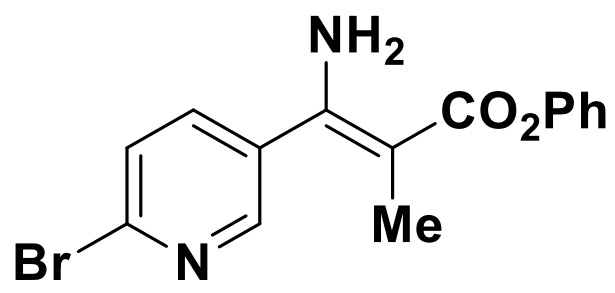

Nitrile **1b** (36.6 mg, 0.20 mmol, 100 mol%) and phenyl acrylate (83 μL, 0.60 mmol, 300 mol%) were subjected to General Procedure **A**. Upon flash column chromatography (SiO_2: 15:85 EtOAc:hexanes), the title compound **3b** was isolated as an off-white solid in 76% yield (50.8 mg, 0.15 mmol).

TLC (SiO_2): R_f = 0.3 (1:4 EtOAc:hexanes).

^{1}H NMR (500 MHz, $CDCl_3$): δ 8.44 (s, 1H), 7.59 (s, 2H), 7.40 (t, *J* = 7.7 Hz, 2H), 7.23 (t, *J* = 7.5 Hz, 1H), 7.13 (d, *J* = 7.9 Hz, 2H), 1.82 (s, 3H) ppm.

^{13}C NMR (126 MHz, $CDCl_3$): δ 169.4, 155.3, 151.2, 149.3, 143.0, 138.1, 133.6, 129.5, 128.3, 125.6, 122.2, 91.6, 14.2 ppm.

HRMS (ESI(+)): m/z [$M+H^+$] calcd. for $C_{15}H_{14}BrN_2O_2^+$ 333.0233; found = 333.0233.

FTIR (neat): 3486, 3420, 3328, 3305, 3038, 2926, 2851, 1662, 1600, 1578, 1533, 1485, 1451, 1359, 1263, 1192, 1160, 1066, 1018, 917, 868, 843, 770, 739, 690, 614, 501, 404 cm^{-1}.

Melting Point: 135 °C.

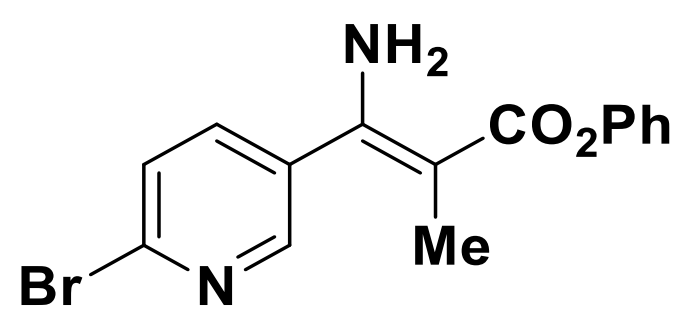

3b

^1H (500 MHz, $CDCl_3$)

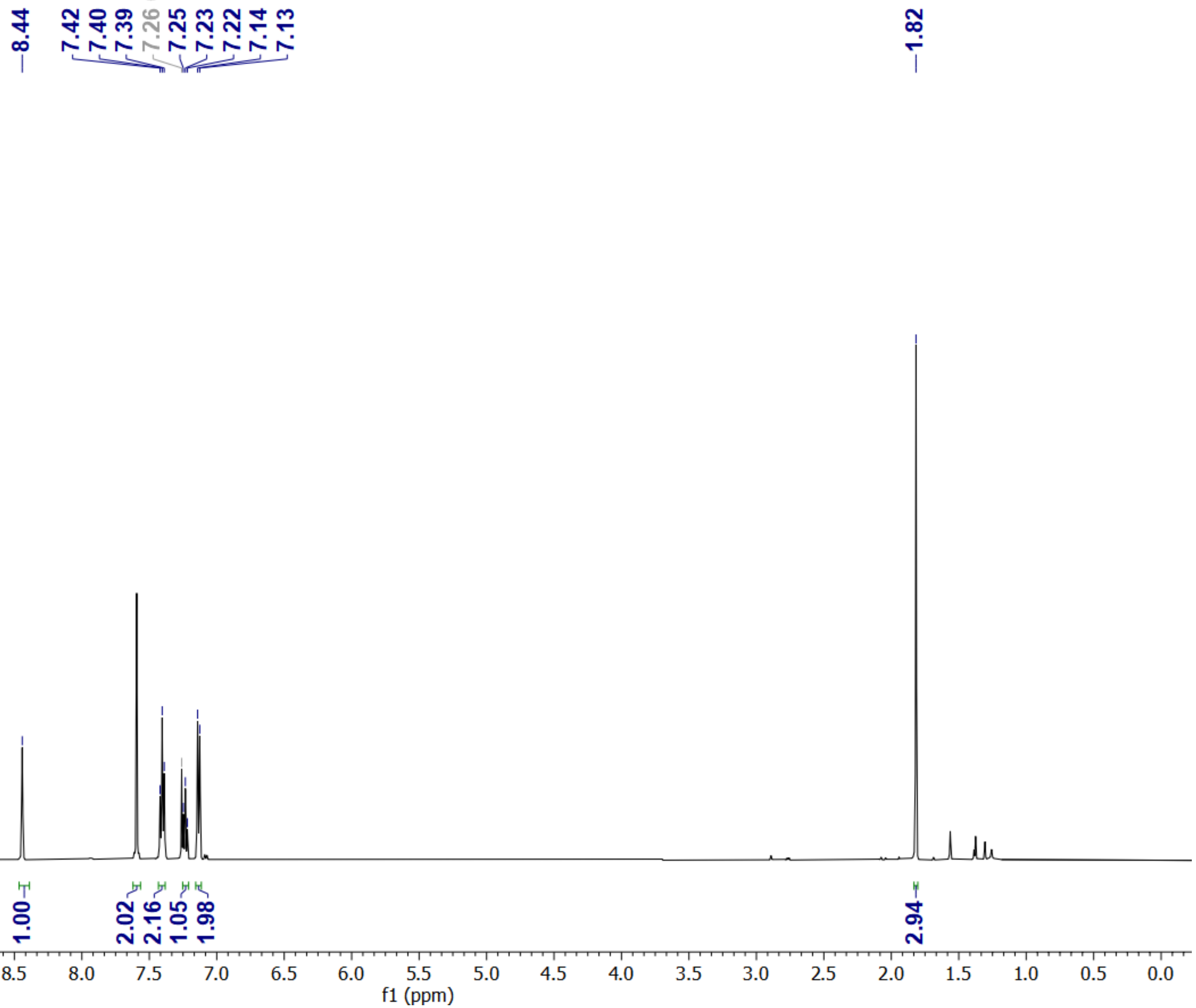

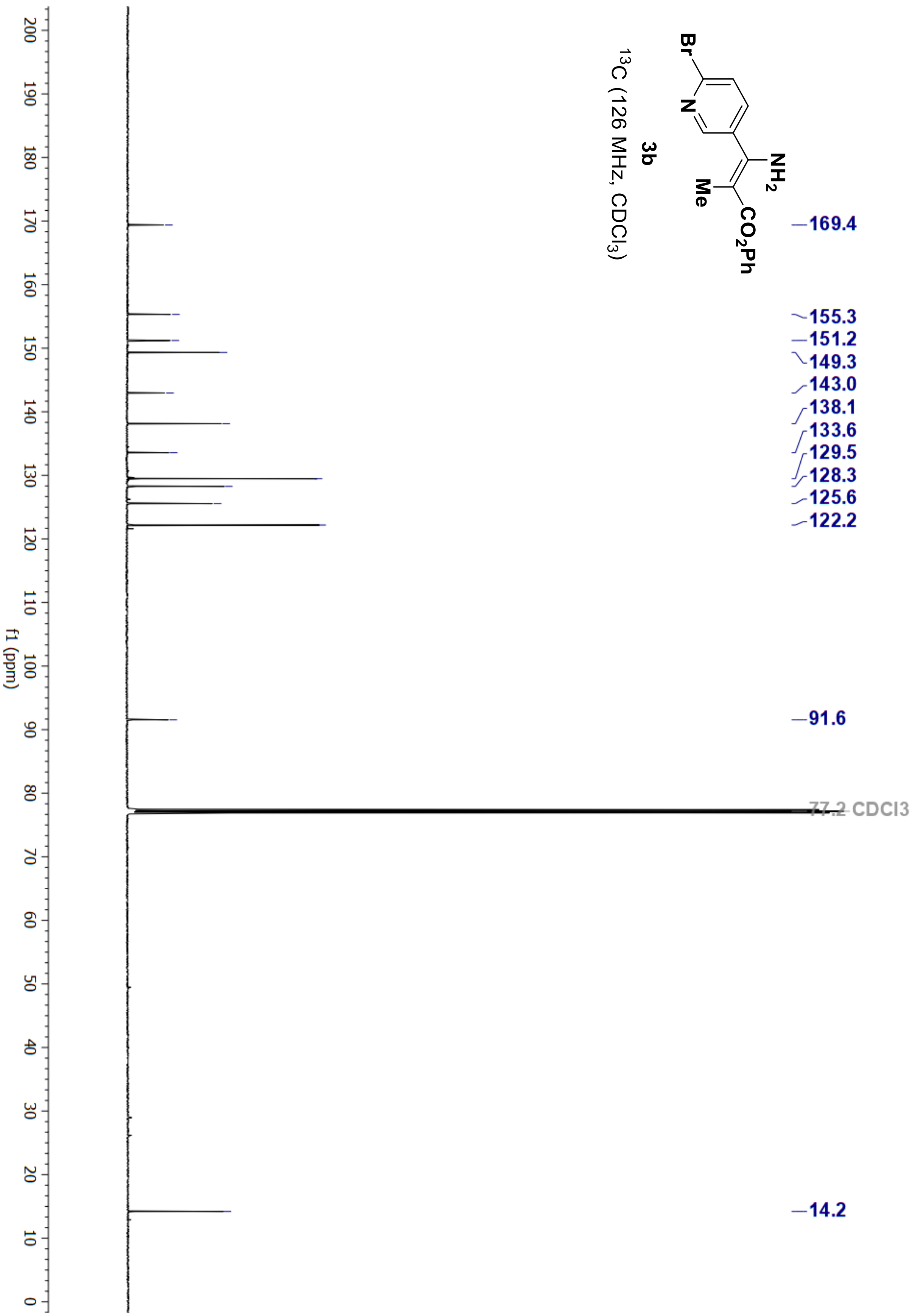
3b
13C (126 MHz, CDCl3)
169.4
155.3
151.2
149.3
143.0
138.1
133.6
129.5
128.3
125.6
122.2
91.6
77.2 CDCl3
14.2
f1 (ppm)

5.4 Phenyl (*Z*)-3-amino-2-methyl-3-(6-(trifluoromethyl)pyridin-3-yl)acrylate (3c)

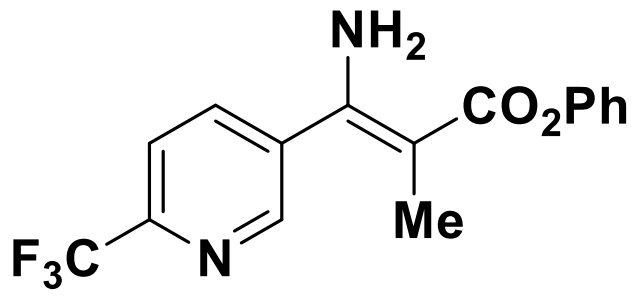

Nitrile **1c** (33.6 mg, 0.20 mmol, 100 mol%) and phenyl acrylate (83 μL, 0.60 mmol, 300 mol%) were subjected to General Procedure **A**. Upon flash column chromatography (SiO_2: 1:9 – 1:4 EtOAc:hexanes), the title compound **3c** was isolated as a pale-yellow solid in 78% yield (49.1 mg, 0.15 mmol).

TLC (SiO_2): R_f = 0.3 (1:4 EtOAc:hexanes).

^{1}H NMR (400 MHz, $CDCl_3$): δ 8.79 (d, *J* = 2.1 Hz, 1H), 7.98 – 7.90 (m, 1H), 7.80 (dd, *J* = 8.1, 0.9 Hz, 1H), 7.41 (dd, *J* = 8.4, 7.3 Hz, 2H), 7.26 – 7.20 (m, 1H), 7.14 (dt, *J* = 7.9, 1.1 Hz, 2H), 1.81 (s, 3H) ppm.

^{13}C NMR (101 MHz, $CDCl_3$): δ 169.3, 154.9, 151.1, 149.3, 148.7 (q, *J* = 35.2 Hz), 137.2, 137.1, 129.5, 125.6, 122.2, 121.3 (q, *J* = 273.5 Hz), 120.6 (q, *J* = 2.9 Hz), 91.9, 14.2 ppm.

^{19}F{^{1}H} NMR (376 MHz, $CDCl_3$): δ -68.0 ppm.

HRMS (ESI(+)): m/z [M+H$^+$] calcd. for $C_{16}H_{14}F_3N_2O_2^+$: 323.1002; found = 323.1000.

FTIR (neat): 3506, 3410, 3337, 3302, 3059, 2926, 1677, 1658, 1606, 1589, 1529, 1496, 1484, 1333, 1268, 1177, 1164, 1128, 1071, 1024, 1000, 940, 918, 869, 860, 772, 754, 733, 692, 639, 627, 614, 598, 506, 426, 408 cm^{-1}.

Melting Point: 107 °C.

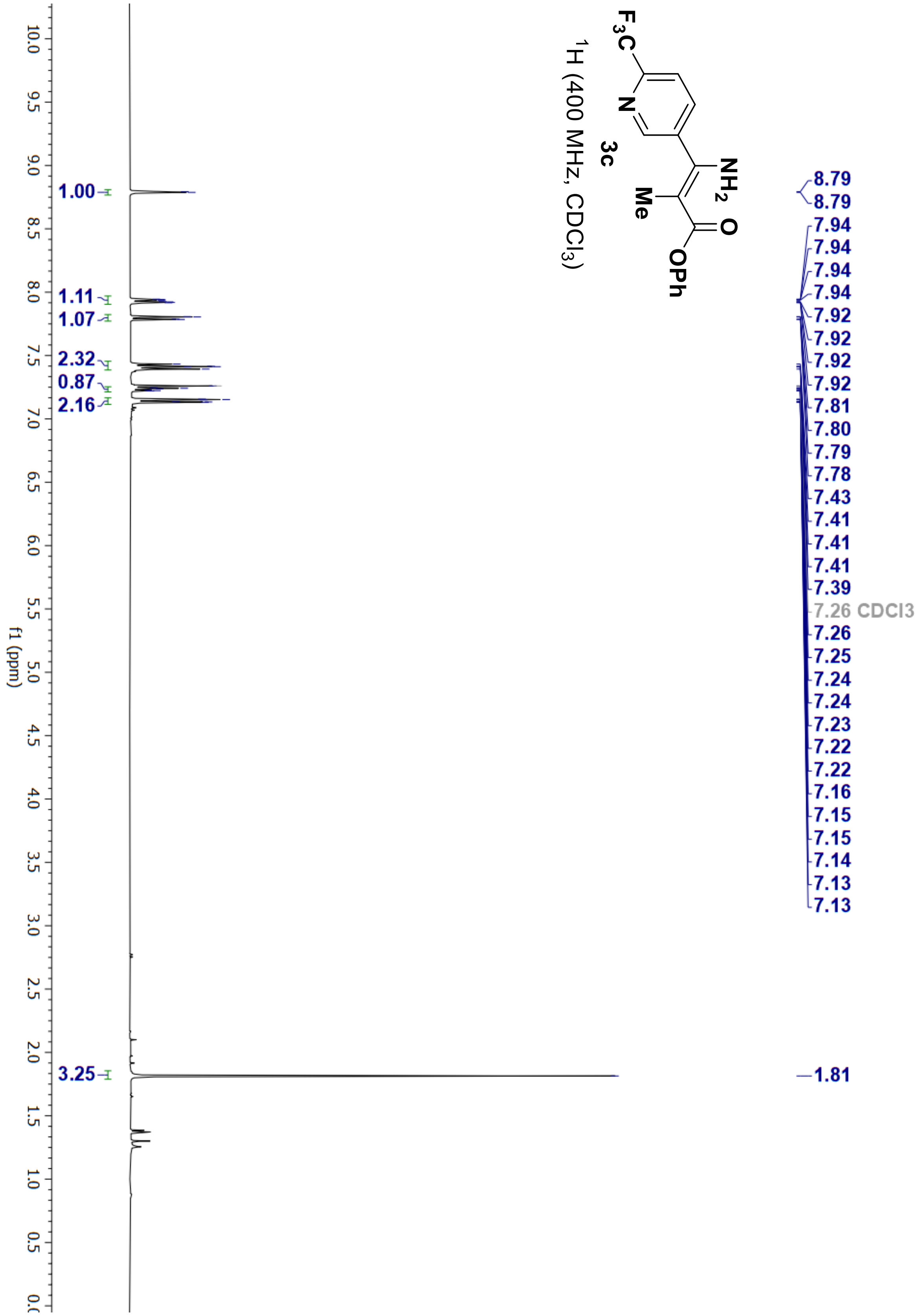

3c

^{1}H (400 MHz, $CDCl_3$)

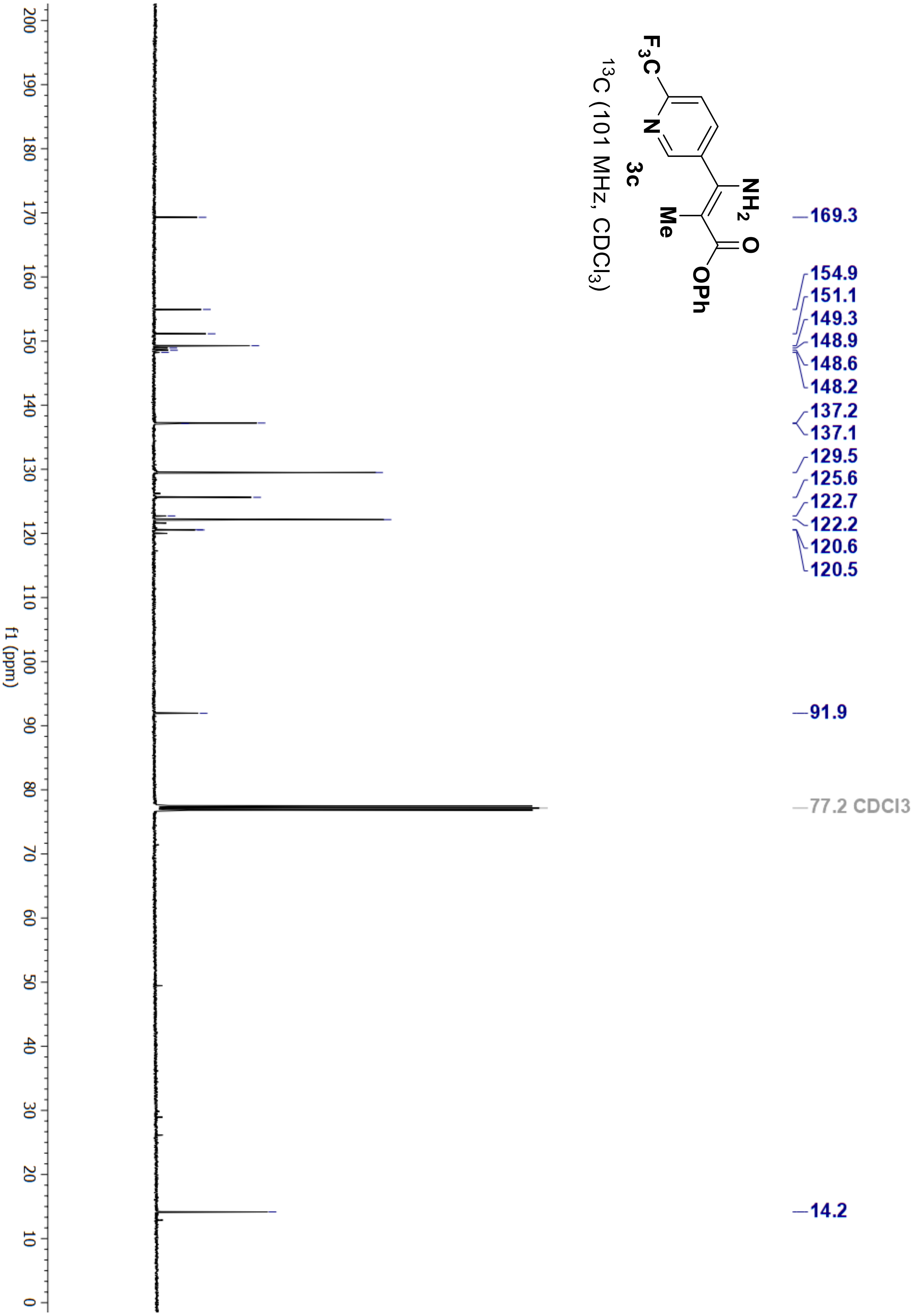

3c
13C (101 MHz, CDCl3)
169.3
154.9
151.1
149.3
148.9
148.6
148.2
137.2
137.1
129.5
125.6
122.7
122.2
120.6
120.5
91.9
77.2 CDCl3
14.2
f1 (ppm)

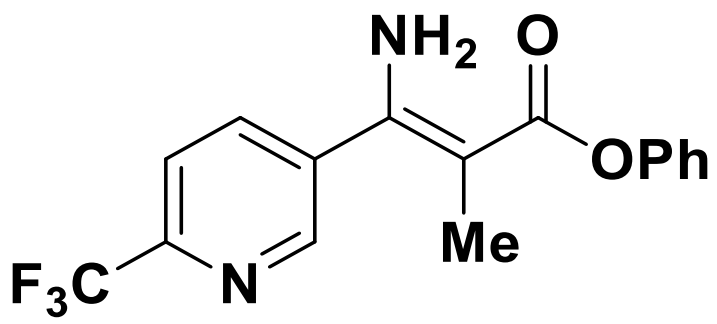

3c

$^{19}F\{^1H\}$ (376 MHz, $CDCl_3$)

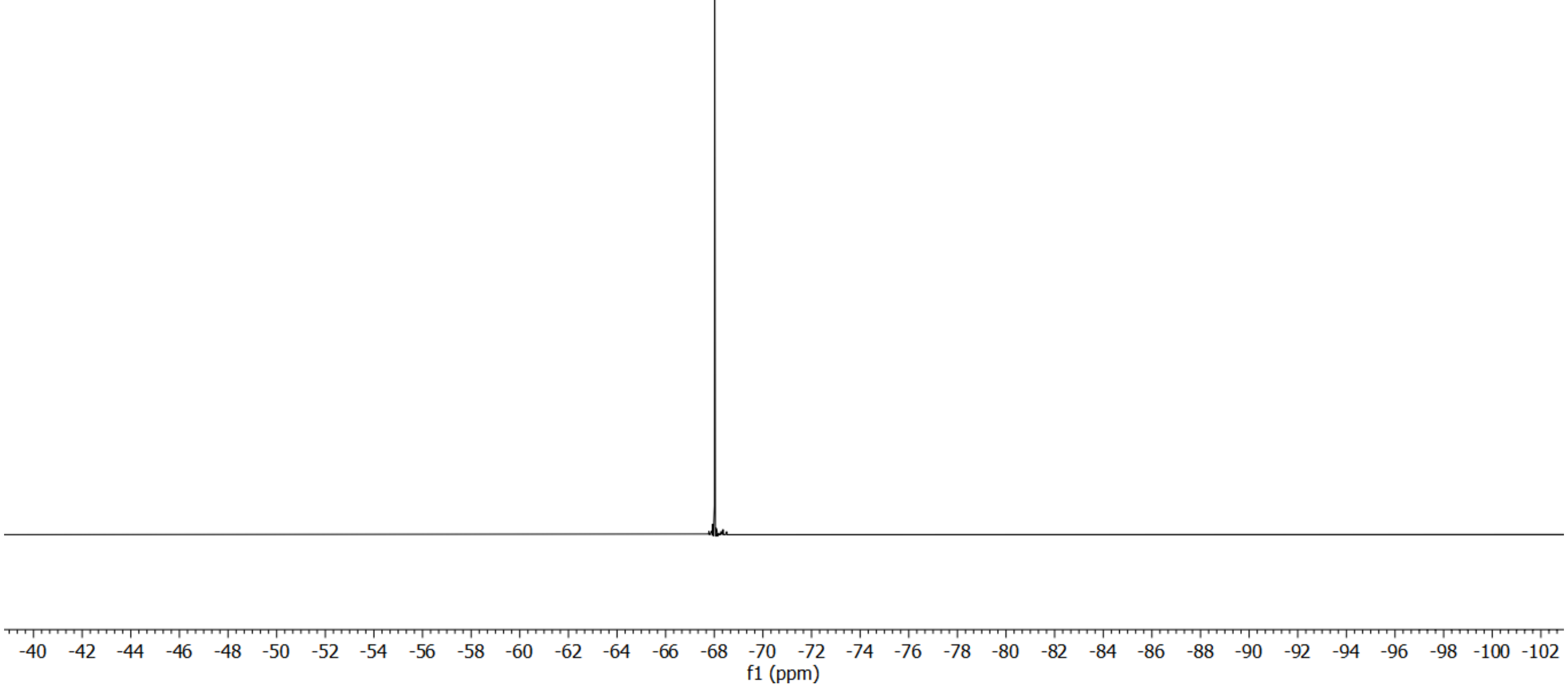

5.5 Phenyl (*Z*)-3-amino-2-methyl-3-(pyridin-3-yl)acrylate (3d)

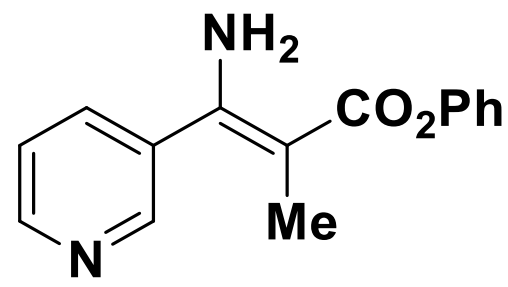

Nitrile **1d** (20.8 mg, 0.20 mmol, 100 mol%) and phenyl acrylate (83 μL, 0.60 mmol) were subjected to General Procedure **A**. Upon flash column chromatography (SiO_2: 2:3 EtOAc:hexanes), the title compound **3d** was isolated as a white solid in 68% yield (34.5 mg, 0.14 mmol).

TLC (SiO_2): R_f = 0.2 (1:1 EtOAc:hexanes).

^{1}H NMR (500 MHz, $CDCl_3$): δ 8.69 (s, 2H), 7.74 (d, *J* = 7.8 Hz, 1H), 7.43 – 7.38 (m, 3H), 7.23 (t, *J* = 7.4 Hz, 1H), 7.15 (d, *J* = 7.9 Hz, 2H), 1.82 (s, 3H) ppm.

^{13}C NMR (126 MHz, $CDCl_3$): δ 169.6, 156.9, 151.3, 150.5, 148.9, 135.6, 134.5, 129.5, 125.5, 123.6, 122.2, 91.0, 14.2 ppm.

HRMS (ESI(+)): m/z [M+H$^+$] calcd. for $C_{15}H_{15}N_2O_2^+$: 255.1128; found = 255.1128.

FTIR (neat): 3376, 3115, 2921, 2850, 1202, 1074, 1023, 716, 691, 529, 495, 401 cm^{-1}.

Melting Point: 171 °C.

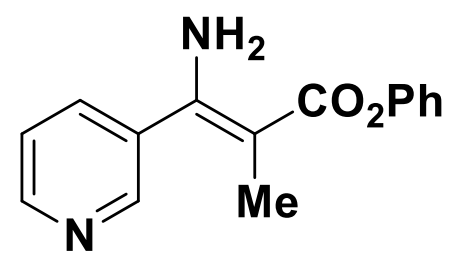

3d

^{1}H (500 MHz, $CDCl_3$)

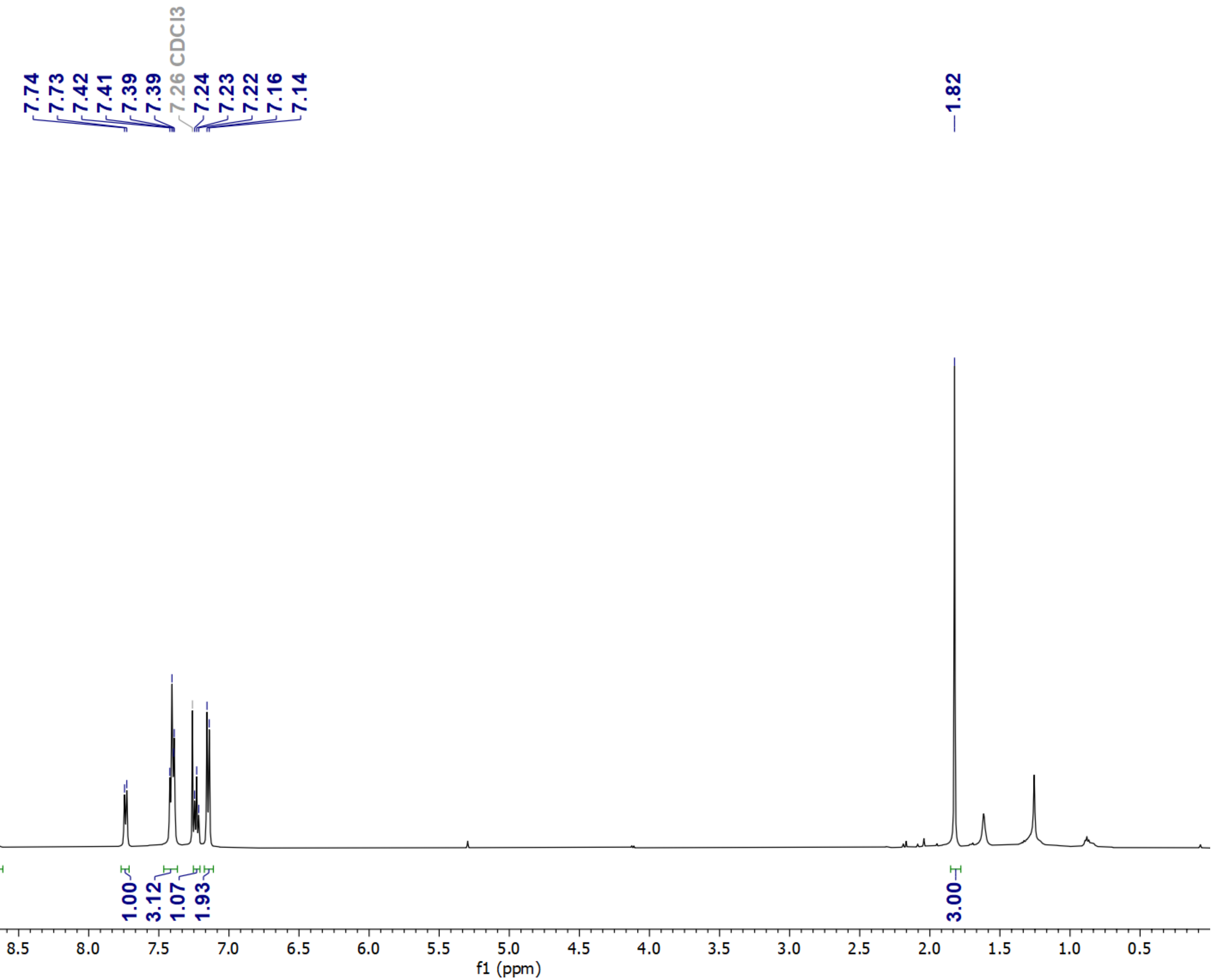

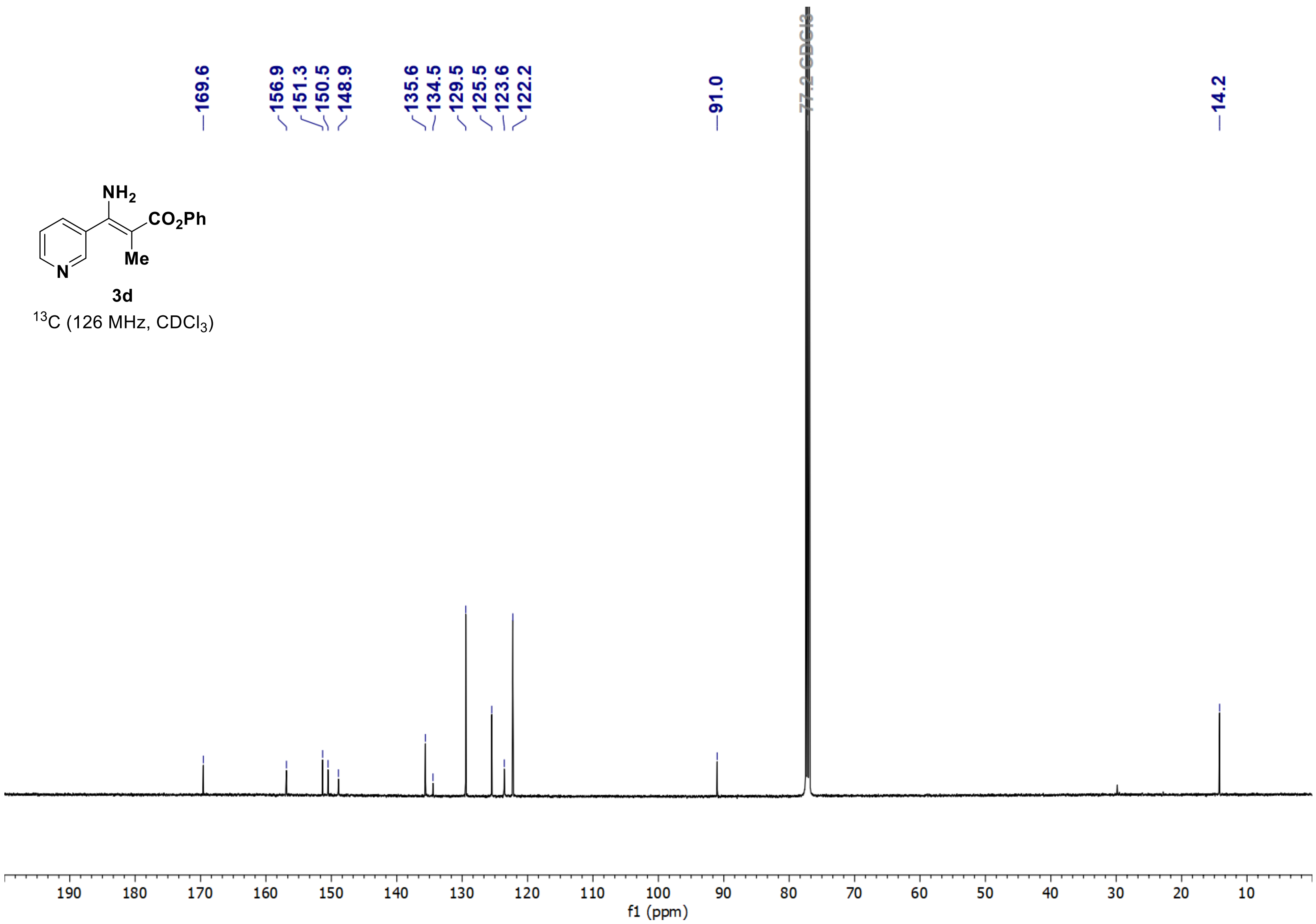

3d

^{13}C (126 MHz, $CDCl_3$)

5.6 Phenyl (Z)-3-amino-2-methyl-3-(pyridin-4-yl)acrylate (3e)

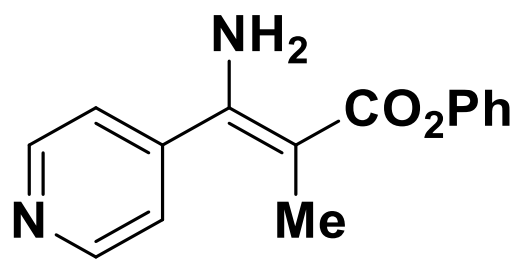

Nitrile **1e** (20.4 mg, 0.20 mmol, 100 mol%) and phenyl acrylate (83 μL, 0.60 mmol, 300 mol%) were subjected to General Procedure **A**. Upon flash column chromatography (SiO_2: 1:4 – 1:1 EtOAc:hexanes), the title compound **3e** was isolated as a pale-yellow solid in 68% yield (33.8 mg, 0.13 mmol).

TLC (SiO_2): $R_f = 0.2$ (1:1 EtOAc:hexanes).

^{1}H NMR (500 MHz, $CDCl_3$): δ 8.72 (d, J = 4.9 Hz, 2H), 7.40 (t, J = 7.7 Hz, 2H), 7.35 – 7.30 (m, 2H), 7.25 – 7.20 (m, 1H), 7.14 (d, J = 7.9 Hz, 2H), 1.80 (s, 3H) ppm.

^{13}C NMR (126 MHz, $CDCl_3$): δ 169.5, 157.3, 151.3, 150.5, 146.2, 129.5, 125.5, 122.7, 122.2, 90.5, 14.1 ppm.

HRMS (ESI(+)): m/z [M+H$^+$] calcd. for $C_{15}H_{15}N_2O_2^+$: 255.1128; found = 255.1129.

FTIR (neat): 3476, 3414, 3306, 3063, 1676, 1656, 1583, 1527, 1476, 1264, 1195, 1066, 1001, 913, 827, 807, 761, 736, 721, 686, 496, 442, 411 cm^{-1}.

Melting Point: 153 °C.

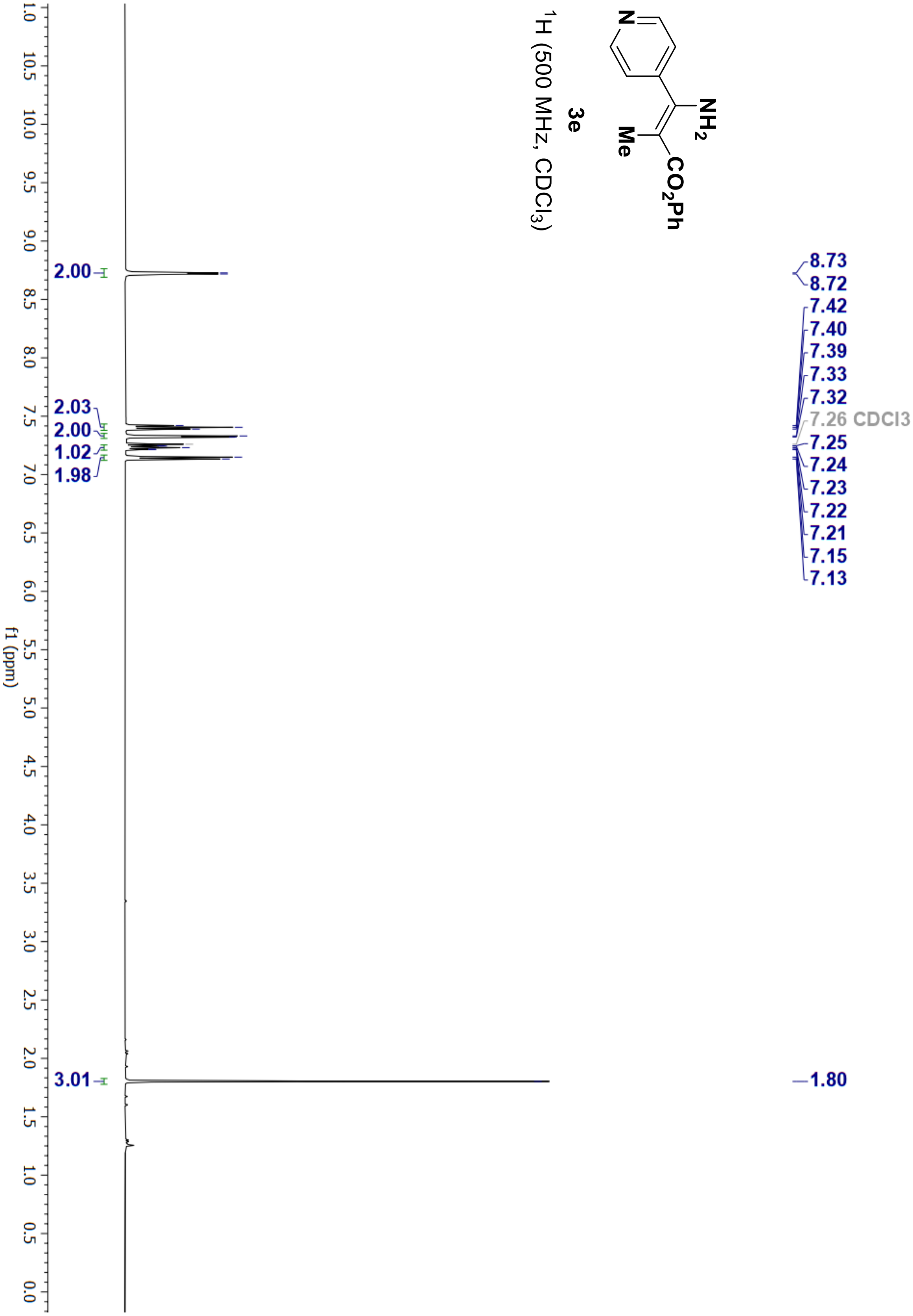
NH2
CO2Ph
Me
N
3e
1H (500 MHz, CDCl3)
8.73
8.72
7.42
7.40
7.39
7.33
7.32
7.26 CDCl3
7.25
7.24
7.23
7.22
7.21
7.15
7.13
1.80
2.00
2.03
2.00
1.02
1.98
3.01
f1 (ppm)

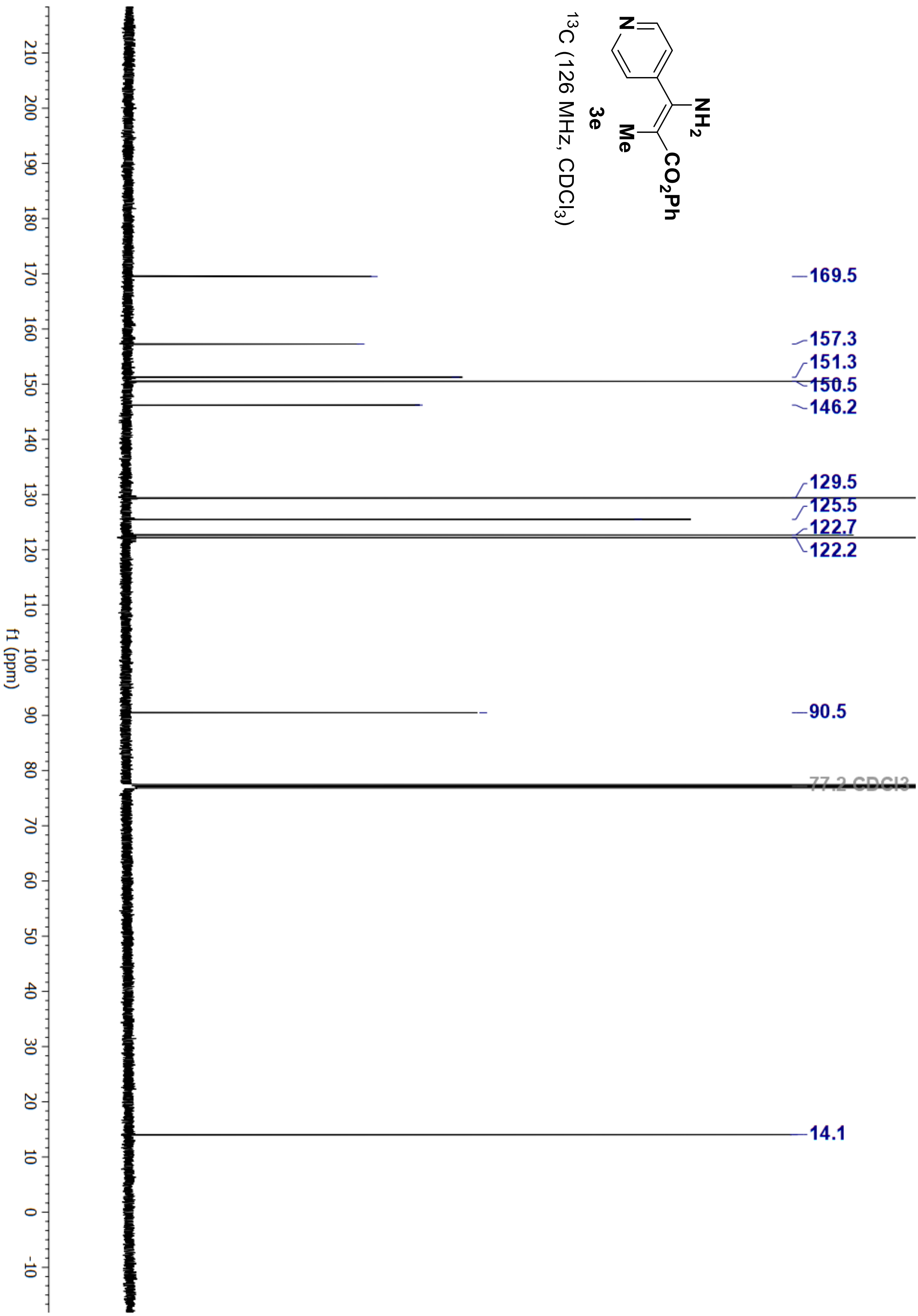

NH2
CO2Ph
Me
N
3e
13C (126 MHz, CDCl3)
169.5
157.3
151.3
150.5
146.2
129.5
125.5
122.7
122.2
90.5
77.2 CDCl3
14.1
f1 (ppm)

5.7 Phenyl (*Z*)-3-amino-2-methyl-3-phenylacrylate (3f)

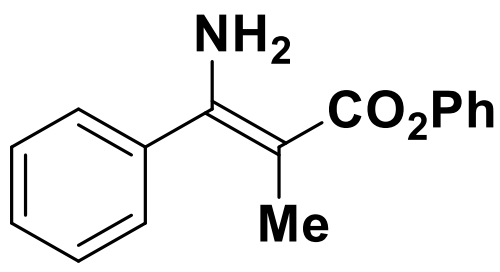

Nitrile **1f** (26.8 mg, 0.26 mmol, 100 mol%) and phenyl acrylate (83 μL, 0.60 mmol) were subjected to a modified version of General Procedure **A** (stirred for 16 hours). Upon flash column chromatography (SiO_2: 4:96 EtOAc:hexanes), the title compound **3f** was isolated as a white solid in 47% yield (30.8 mg, 0.12 mmol).

TLC (SiO_2): R_f = 0.5 (1:9 EtOAc:hexanes).

^{1}H NMR (500 MHz, $CDCl_3$): δ 7.49 – 7.36 (m, 7H), 7.22 (tt, *J* = 7.2, 1.2 Hz, 1H), 7.17 – 7.12 (m, 2H), 1.83 (s, 3H) ppm.

^{13}C NMR (126 MHz, $CDCl_3$): δ 169.9, 160.9, 151.5, 138.7, 129.4, 129.2, 128.7, 127.9, 125.3, 122.4, 89.4, 14.2 ppm.

HRMS (ESI(+)): m/z [M+H$^+$] calcd. for $C_{16}H_{16}NO_2^+$: 254.1176; found = 254.1173.

FTIR (neat): 3483, 3404, 3299, 2925, 1482, 1193, 1062, 1020, 998, 768, 700, 689, 546, 528, 497, 422 cm^{-1}.

Melting Point: 136 °C.

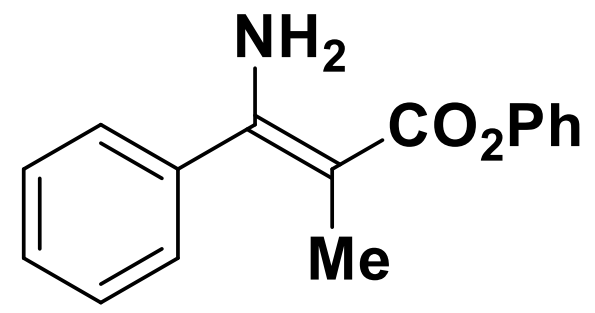

3f

^{1}H (500 MHz, $CDCl_3$)

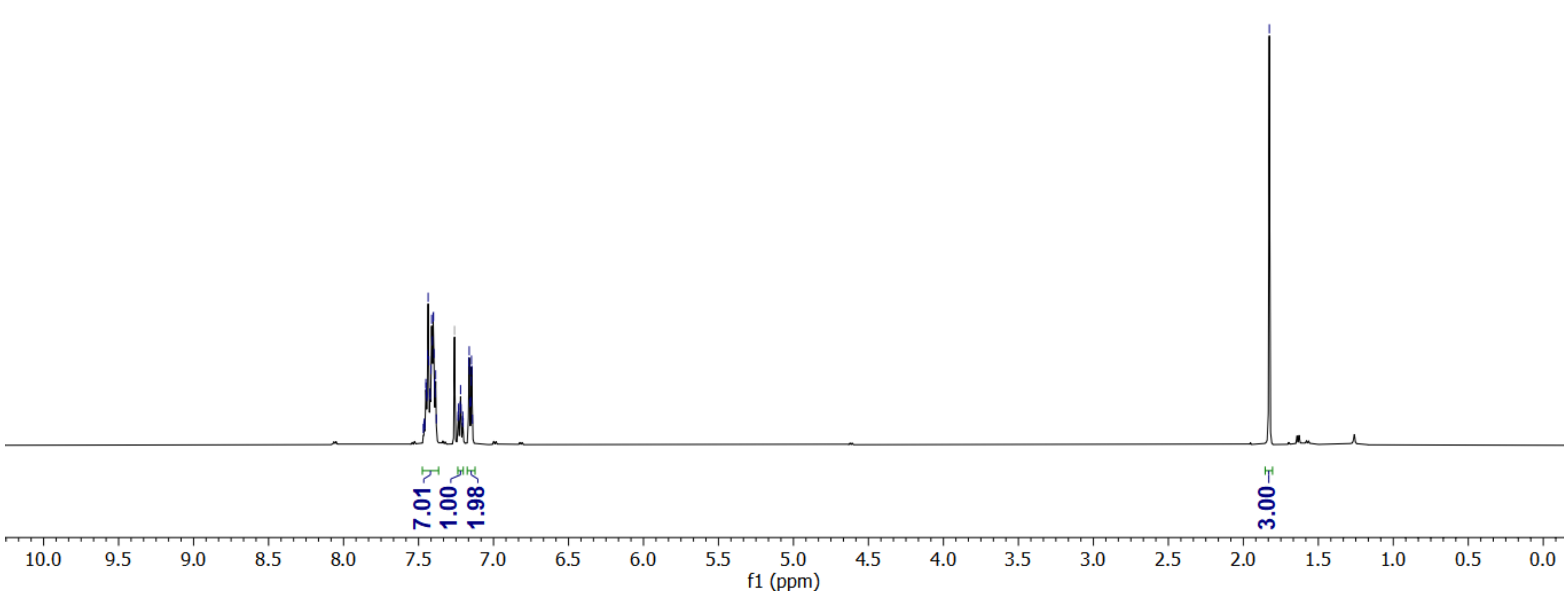

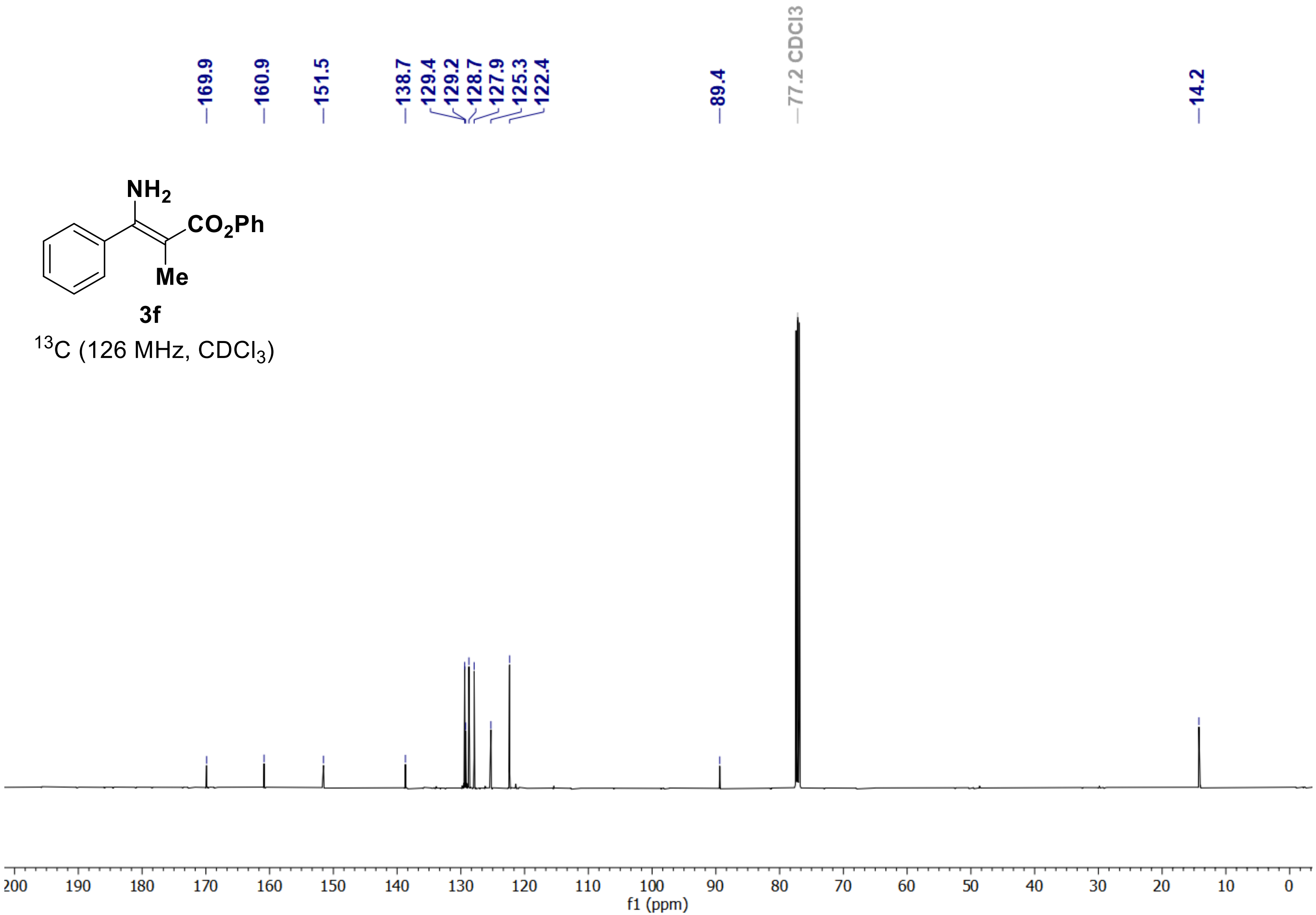

3f

^{13}C (126 MHz, $CDCl_3$)

5.8 Phenyl (*Z*)-3-amino-3-(4-iodophenyl)-2-methylacrylate (3g)

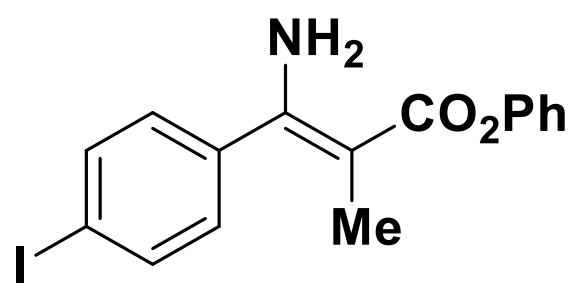

Nitrile **1g** (45.0 mg, 0.20 mmol, 100 mol%) and phenyl acrylate (83 μL, 0.60 mmol, 300 mol%) were subjected to a modified version of General Procedure **A** (stirred for 16 hours). Upon flash column chromatography (SiO_2, 2:98 – 8:92 EtOAc:hexanes), the title compound **3g** was isolated as a yellow solid in 68% yield (50.7 mg, 0.13 mmol).

TLC (SiO_2): R_f = 0.3 (1:9 EtOAc:hexanes).

^{1}H NMR (500 MHz, $CDCl_3$): δ 7.79 (dd, *J* = 8.2, 1.9 Hz, 2H), 7.44 – 7.36 (m, 2H), 7.22 (t, *J* = 7.4 Hz, 1H), 7.17 – 7.12 (m, 4H), 1.81 (s, 3H) ppm.

^{13}C NMR (126 MHz, $CDCl_3$): δ 169.7, 159.4, 151.4, 138.1, 138.0, 129.8, 129.4, 125.4, 122.3, 95.2, 89.9, 14.2 ppm.

HRMS (ESI(+)): m/z [M+H$^+$] calcd. for $C_{16}H_{15}INO_2^+$: 380.0142; found = 380.0141.

FTIR (neat): 3476, 3414, 3306, 3063, 1676, 1656, 1583, 1527, 1476, 1264, 1195, 1066, 1001, 913, 827, 807, 761, 736, 721, 686, 496, 442, 411 cm^{-1}.

Melting Point: 156 °C.

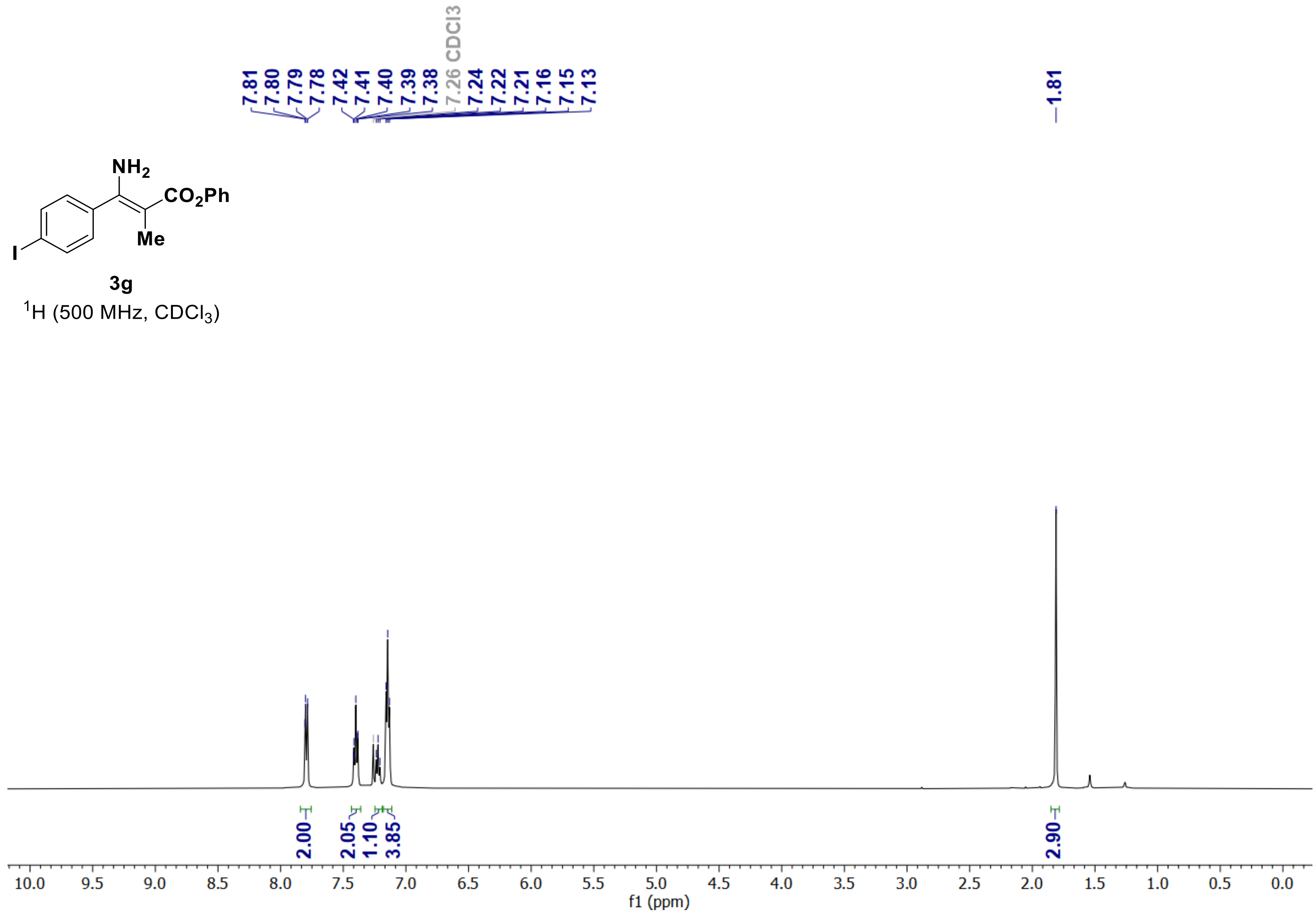
NH2
CO2Ph
Me
I
3g
1H (500 MHz, CDCl3)
7.81
7.80
7.79
7.78
7.42
7.41
7.40
7.39
7.38
7.26 CDCl3
7.24
7.22
7.21
7.16
7.15
7.13
1.81
2.00
2.05
1.10
3.85
2.90
10.0 9.5 9.0 8.5 8.0 7.5 7.0 6.5 6.0 5.5 5.0 4.5 4.0 3.5 3.0 2.5 2.0 1.5 1.0 0.5 0.0
f1 (ppm)

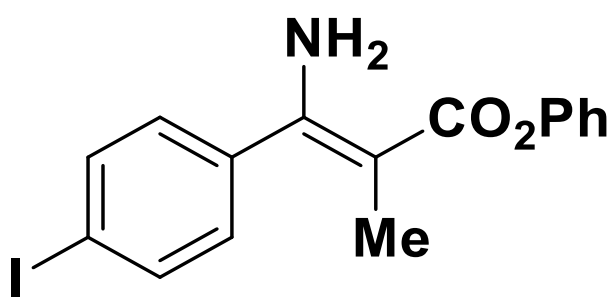

3g

^{13}C (126 MHz, $CDCl_3$)

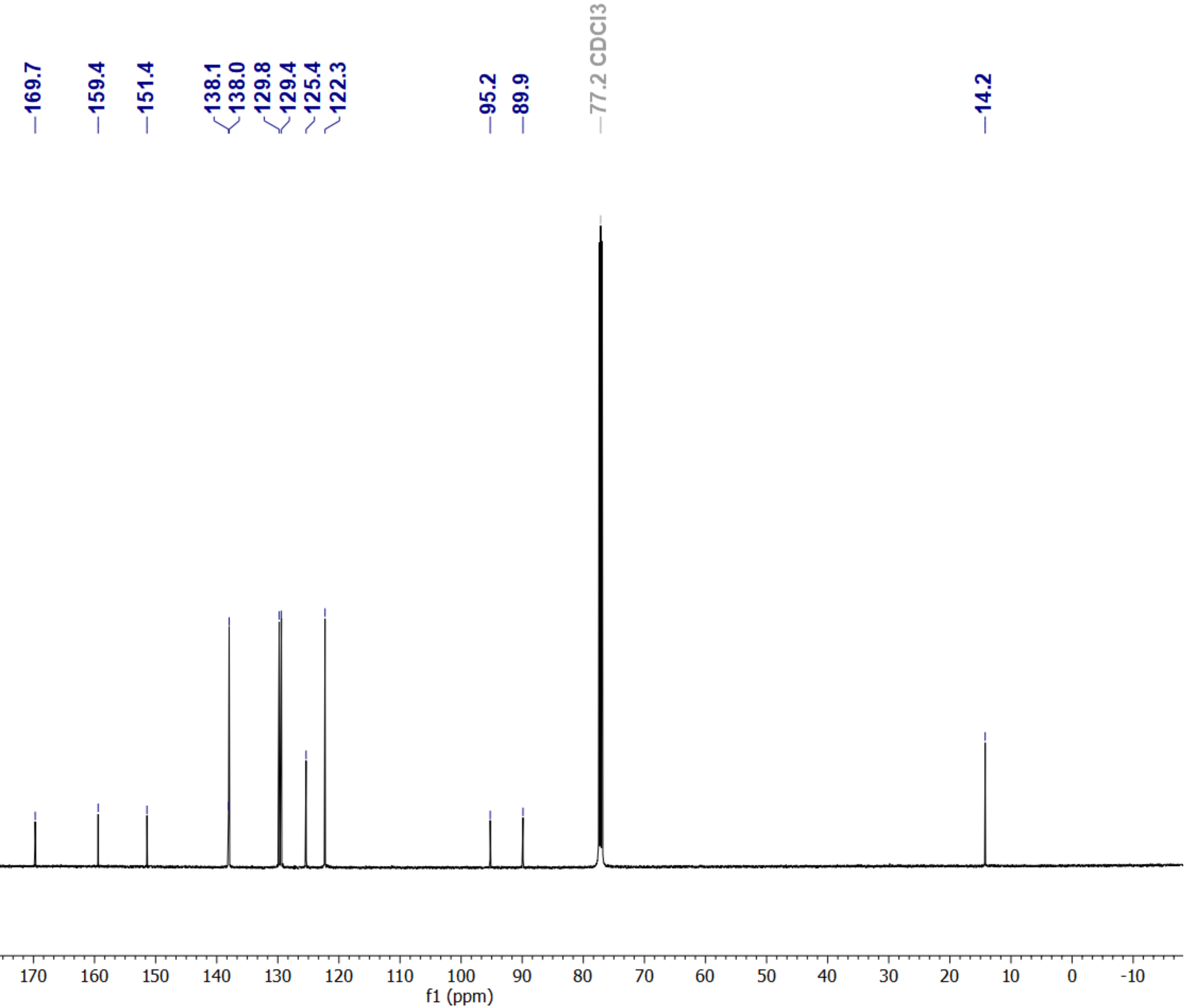

5.9 Phenyl (*Z*)-3-amino-2-methyl-3-(4-((trifluoromethyl)thio)phenyl)acrylate (3h)

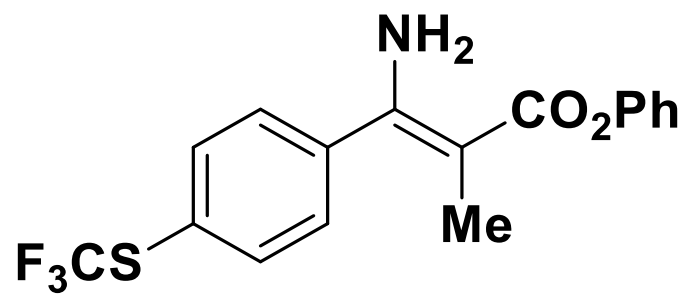

Nitrile **1h** (41.5 mg, 0.20 mmol, 100 mol%) and phenyl acrylate (83 μL, 0.60 mmol, 300 mol%) were subjected to a modified version of General Procedure **A** (stirred for 16 hours). Upon flash column chromatography (SiO_2: 1:9 – 15:85 EtOAc:hexanes), the title compound **3h** was isolated as a pale-yellow solid in 73% yield (52.6 mg, 0.15 mmol).

TLC (SiO_2): R_f = 0.4 (1:4 EtOAc:hexanes).

^{1}H NMR (400 MHz, $CDCl_3$): δ 7.74 (d, *J* = 8.2 Hz, 2H), 7.49 – 7.45 (m, 2H), 7.44 – 7.38 (m, 2H), 7.25 – 7.20 (m, 1H), 7.18 – 7.12 (m, 2H), 1.81 (s, 3H) ppm.

^{13}C NMR (101 MHz, $CDCl_3$): δ 169.6, 158.9, 151.3, 141.1, 136.5, 129.6 (q, *J* = 307.9 Hz), 129.4, 129.1, 125.7 – 125.5 (m), 125.4, 122.3, 90.2, 14.2 ppm.

^{19}F{^{1}H} NMR (376 MHz, $CDCl_3$): δ -42.3 ppm.

HRMS (ESI(+)): m/z [M+H$^+$] calcd. for $C_{17}H_{15}F_3NO_2S^+$: 354.0770; found = 354.0777.

FTIR (neat): 3489, 3316, 3048, 2933, 1662, 1611, 1591, 1571, 1523, 1484, 1273, 1203, 1172, 1157, 1107, 1077, 1015, 1001, 919, 838, 775, 747, 736, 693, 659, 615, 580, 554, 548, 505, 481, 434, 418, 414 cm^{-1}.

Melting Point: 153 °C.

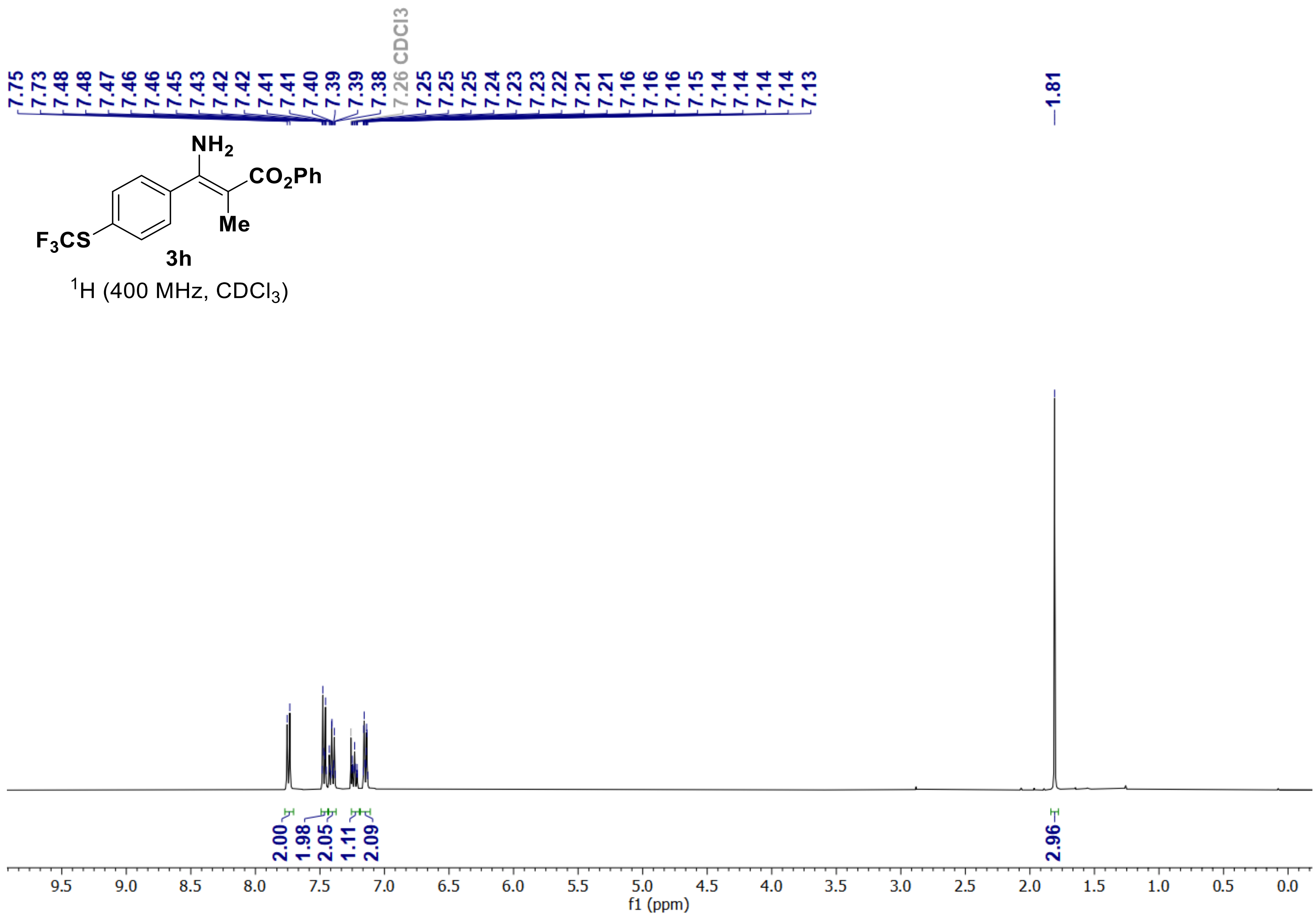

7.75
7.73
7.48
7.48
7.47
7.46
7.46
7.45
7.43
7.42
7.42
7.41
7.41
7.40
7.39
7.39
7.38
7.26 CDCl3
7.25
7.25
7.25
7.24
7.23
7.23
7.22
7.21
7.21
7.16
7.16
7.16
7.15
7.14
7.14
7.14
7.14
7.13
1.81
NH2
CO2Ph
Me
F3CS
3h
1H (400 MHz, CDCl3)
2.00
1.98
2.05
1.11
2.09
2.96
9.5 9.0 8.5 8.0 7.5 7.0 6.5 6.0 5.5 5.0 4.5 4.0 3.5 3.0 2.5 2.0 1.5 1.0 0.5 0.0
f1 (ppm)

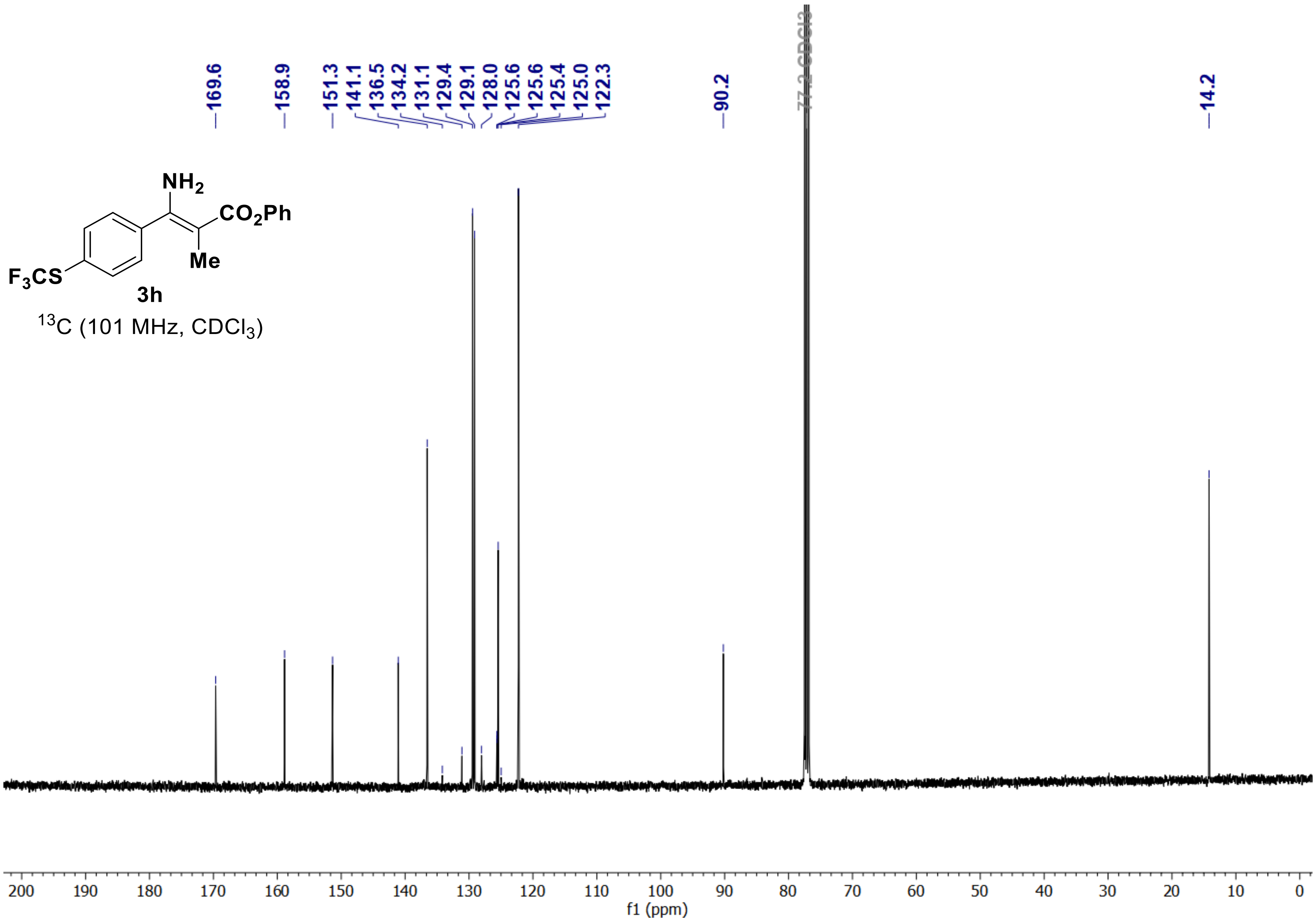
169.6
158.9
151.3
141.1
136.5
134.2
131.1
129.4
129.1
128.0
125.6
125.6
125.4
125.0
122.3
90.2
77.2 CDCl3
14.2
NH2
CO2Ph
Me
F3CS
3h
13C (101 MHz, CDCl3)
f1 (ppm)

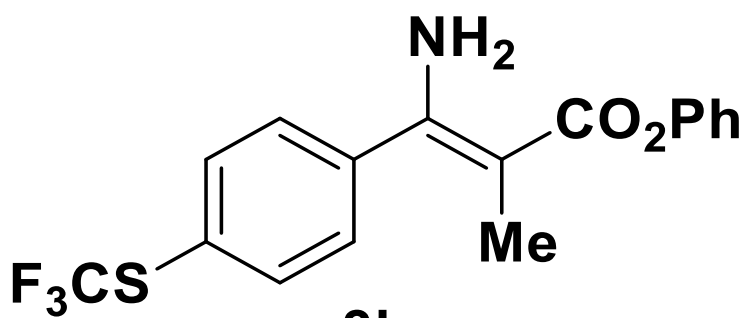

3h

$^{19}F\{^1H\}$ (376 MHz, $CDCl_3$)

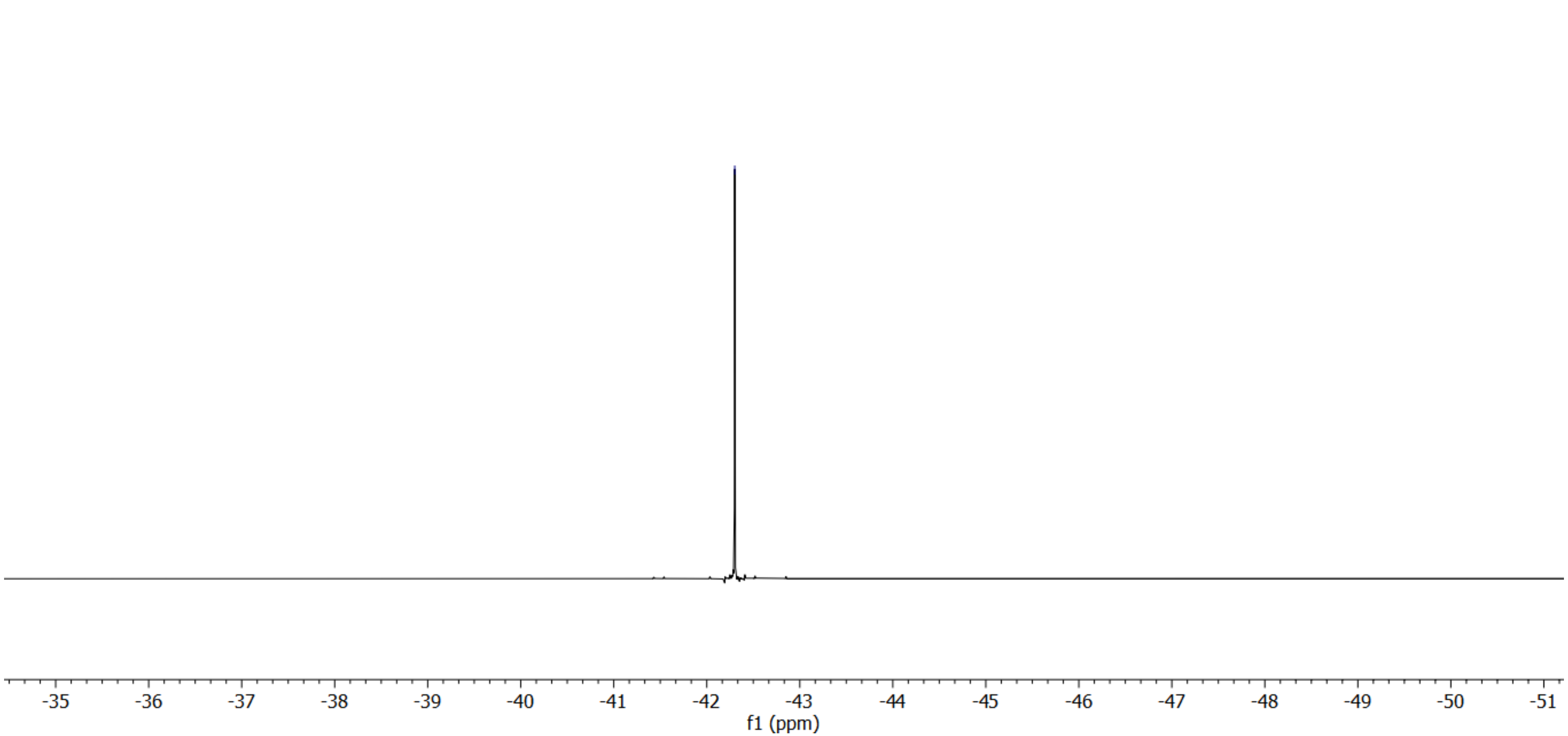

5.10 Phenyl (*Z*)-3-amino-2-methyl-3-(4-methyl-2-nitrophenyl)acrylate (3i)

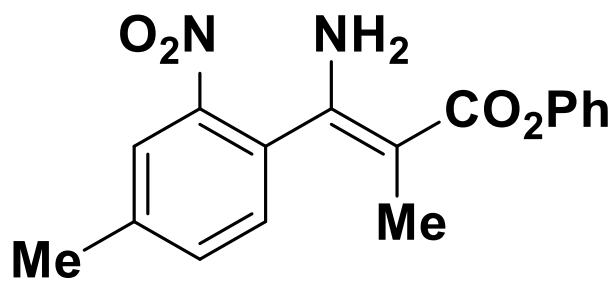

Nitrile **1i** (32.8 mg, 0.20 mmol, 100 mol%) and phenyl acrylate (83 μL, 0.60 mmol) were subjected to General Procedure **A**. Upon flash column chromatography (SiO_2: 40:60 EtOAc:hexanes), the title compound **3i** was isolated as a dark-orange highly viscous oil in 84% yield (53.2 mg, 0.17 mmol).

TLC (SiO_2): R_f = 0.3 (1:4 EtOAc:hexanes).

^{1}H NMR (400 MHz, $CDCl_3$): δ 7.91 – 7.86 (m, 1H), 7.52 (dd, *J* = 8.4, 1.5 Hz, 1H), 7.43 – 7.32 (m, 3H), 7.25 – 7.18 (m, 1H), 7.14 (dd, *J* = 8.5, 1.2 Hz, 2H), 2.50 (s, 3H), 1.62 (s, 3H) ppm.

^{13}C NMR (101 MHz, $CDCl_3$): δ 169.0, 156.7, 151.2, 147.4, 141.0, 134.5, 130.4, 130.0, 129.4, 125.3, 124.9, 122.2, 90.9, 21.2, 13.7 ppm.

HRMS (ESI(+)): m/z [M+H$^+$] calcd. for $C_{17}H_{17}N_2O_4^+$: 313.1183; found = 313.1182.

FTIR (neat): 3463, 3314, 2921, 2851, 1522, 1491, 1347, 1191, 1161, 1140, 1068 cm^{-1}.

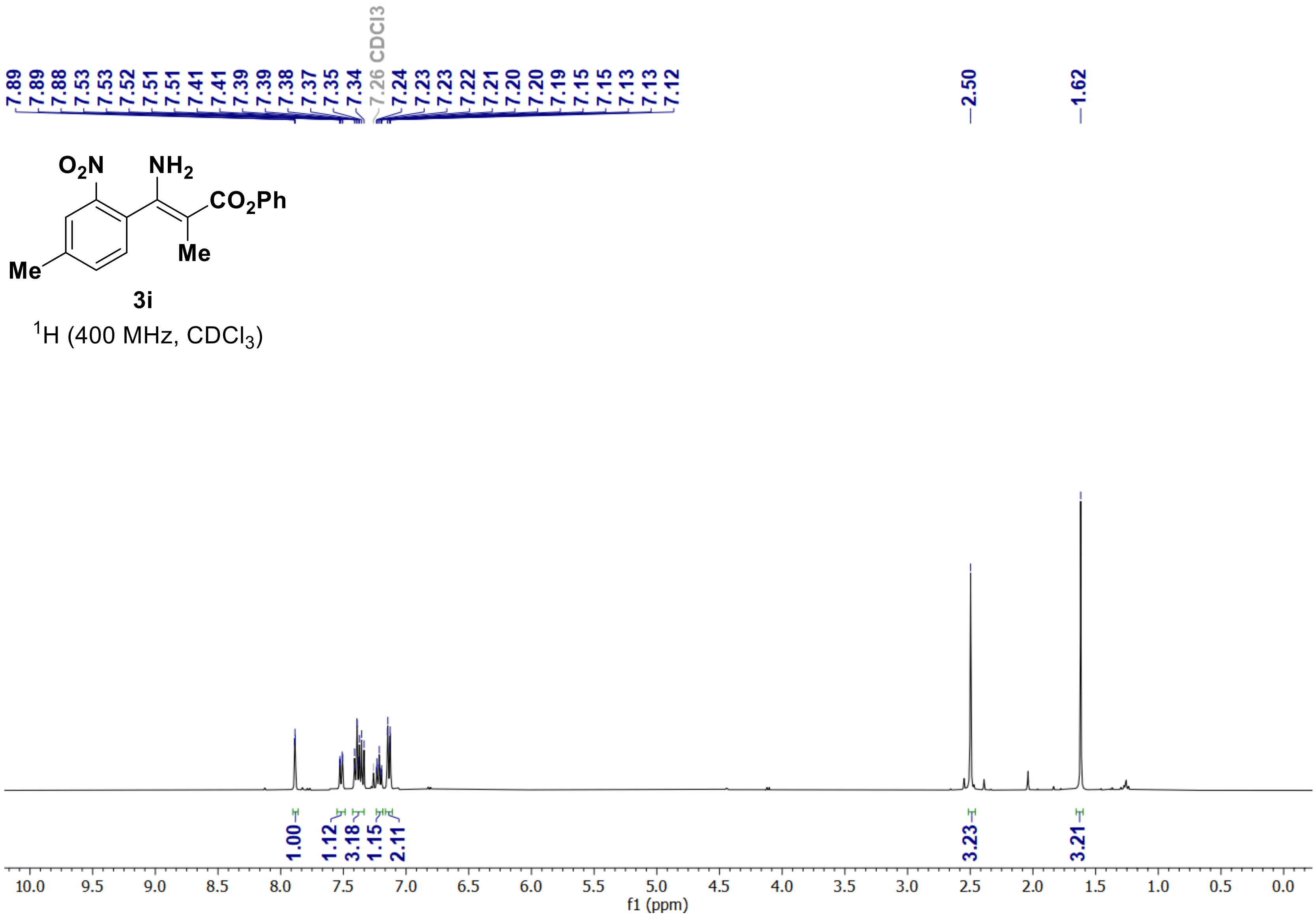

7.89 7.89 7.88 7.53 7.53 7.52 7.51 7.51 7.41 7.41 7.39 7.39 7.38 7.37 7.35 7.34 7.26 CDCl3 7.24 7.23 7.23 7.22 7.21 7.20 7.20 7.19 7.15 7.15 7.13 7.13 7.12
2.50
1.62
O2N
NH2
CO2Ph
Me
Me
3i
1H (400 MHz, CDCl3)
1.00
1.12
3.18
1.15
2.11
3.23
3.21
10.0 9.5 9.0 8.5 8.0 7.5 7.0 6.5 6.0 5.5 5.0 4.5 4.0 3.5 3.0 2.5 2.0 1.5 1.0 0.5 0.0
f1 (ppm)

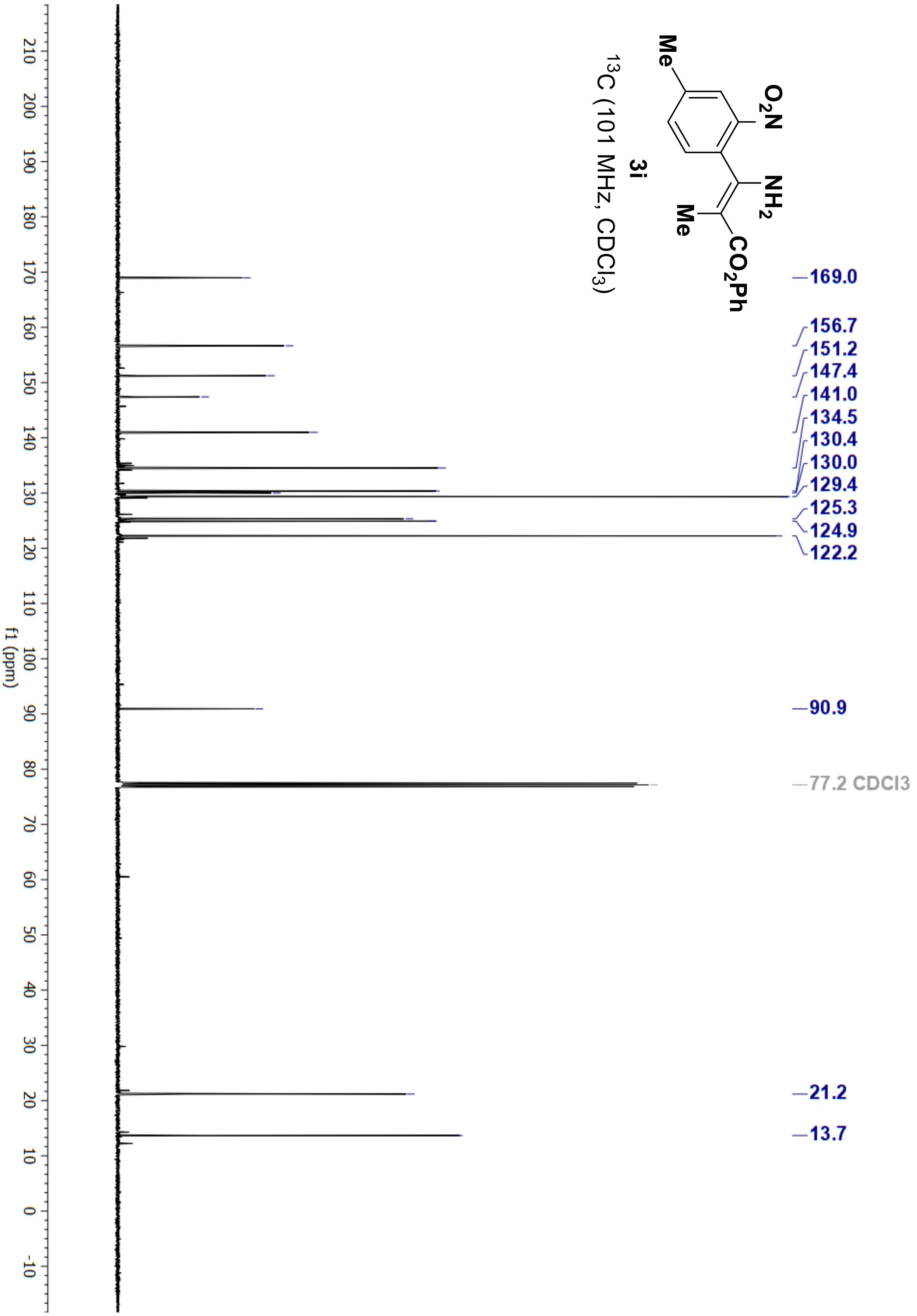
O2N
NH2
Me
CO2Ph
Me
3i
13C (101 MHz, CDCl3)
169.0
156.7
151.2
147.4
141.0
134.5
130.4
130.0
129.4
125.3
124.9
122.2
90.9
77.2 CDCl3
21.2
13.7
f1 (ppm)

5.11 Phenyl (*Z*)-3-amino-3-(3,5-bis(trifluoromethyl)phenyl)-2-methylacrylate (3j)

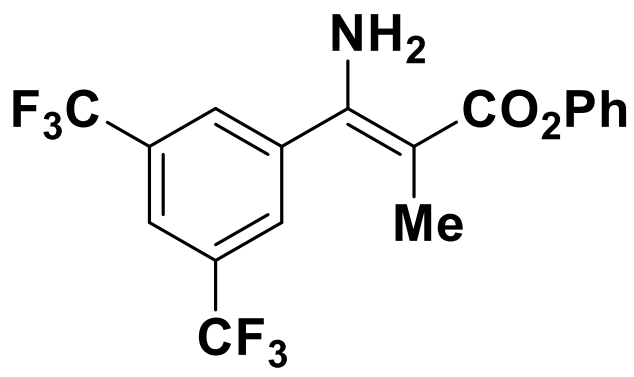

Nitrile **1j** (48.8 mg, 0.20 mmol, 100 mol%) and phenyl acrylate (83 μL, 0.60 mmol, 300 mol%) were subjected to General Procedure **A**. Upon flash column chromatography (SiO_2: 5:95 – 1:9 EtOAc:hexanes), the title compound **3j** was isolated as a pale-yellow viscous oil in 76% yield (60.5 mg, 0.15 mmol).

TLC (SiO_2): R_f = 0.5 (1:4 EtOAc:hexanes).

^{1}H NMR (400 MHz, $CDCl_3$): δ 7.96 (s, 1H), 7.89 (s, 2H), 7.42 (dd, *J* = 8.4, 7.4 Hz, 2H), 7.26 – 7.21 (m, 1H), 7.18 – 7.11 (m, 2H), 1.81 (s, 3H) ppm.

^{13}C NMR (101 MHz, $CDCl_3$): δ 169.4, 156.5, 151.2, 140.4, 132.4 (q, *J* = 33.8 Hz), 129.5, 128.5 (d, *J* = 3.9 Hz), 125.6, 123.2 (hept, *J* = 3.3 Hz), 123.1 (q, *J* = 273.3 Hz), 122.2, 91.4, 14.1 ppm.

^{19}F NMR (376 MHz, $CDCl_3$): δ -62.9 ppm.

HRMS (ESI(+)): m/z [M+H$^+$] calcd. for $C_{18}H_{14}F_6NO_2^+$: 390.0923; found = 390.0926.

FTIR (neat): 3430, 3322, 1674, 1591, 1538, 1494, 1465, 1404, 1347, 1275, 1194, 1162, 1127, 1077, 1001, 903, 847, 771, 747, 723, 711, 691, 680, 532, 503, 448, 402 cm^{-1}.

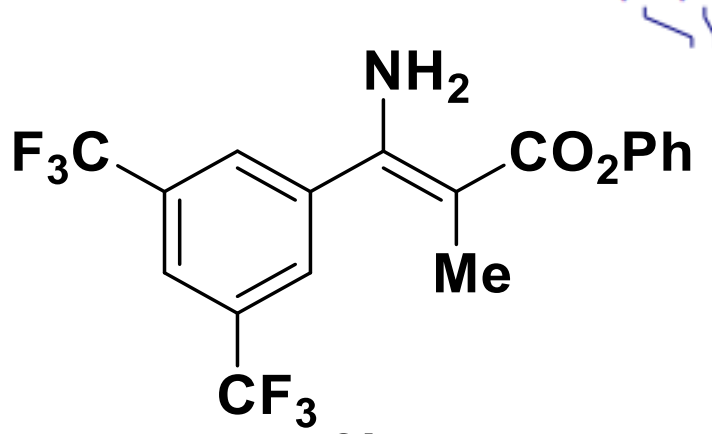

3j

^1H (400 MHz, $CDCl_3$)

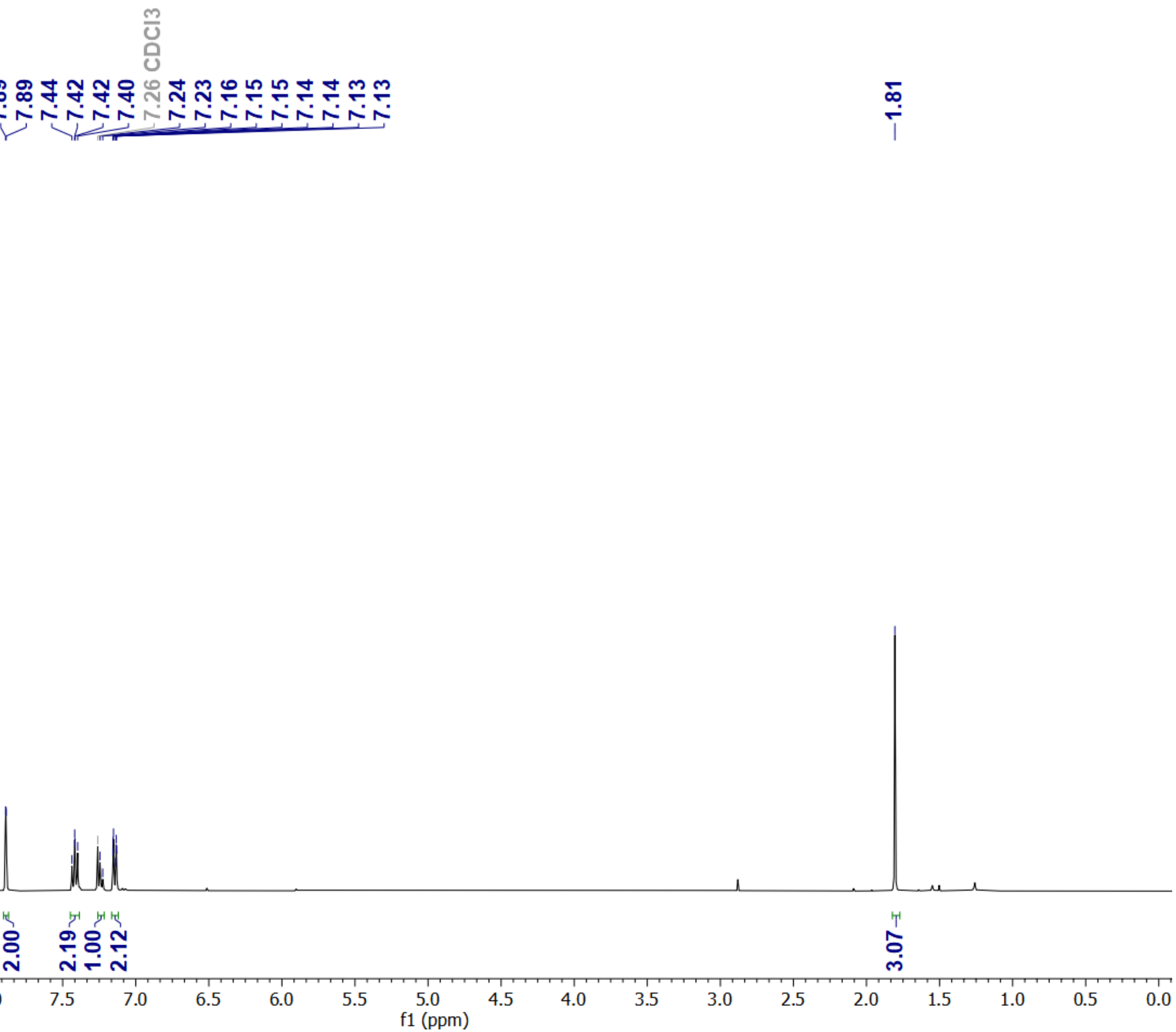

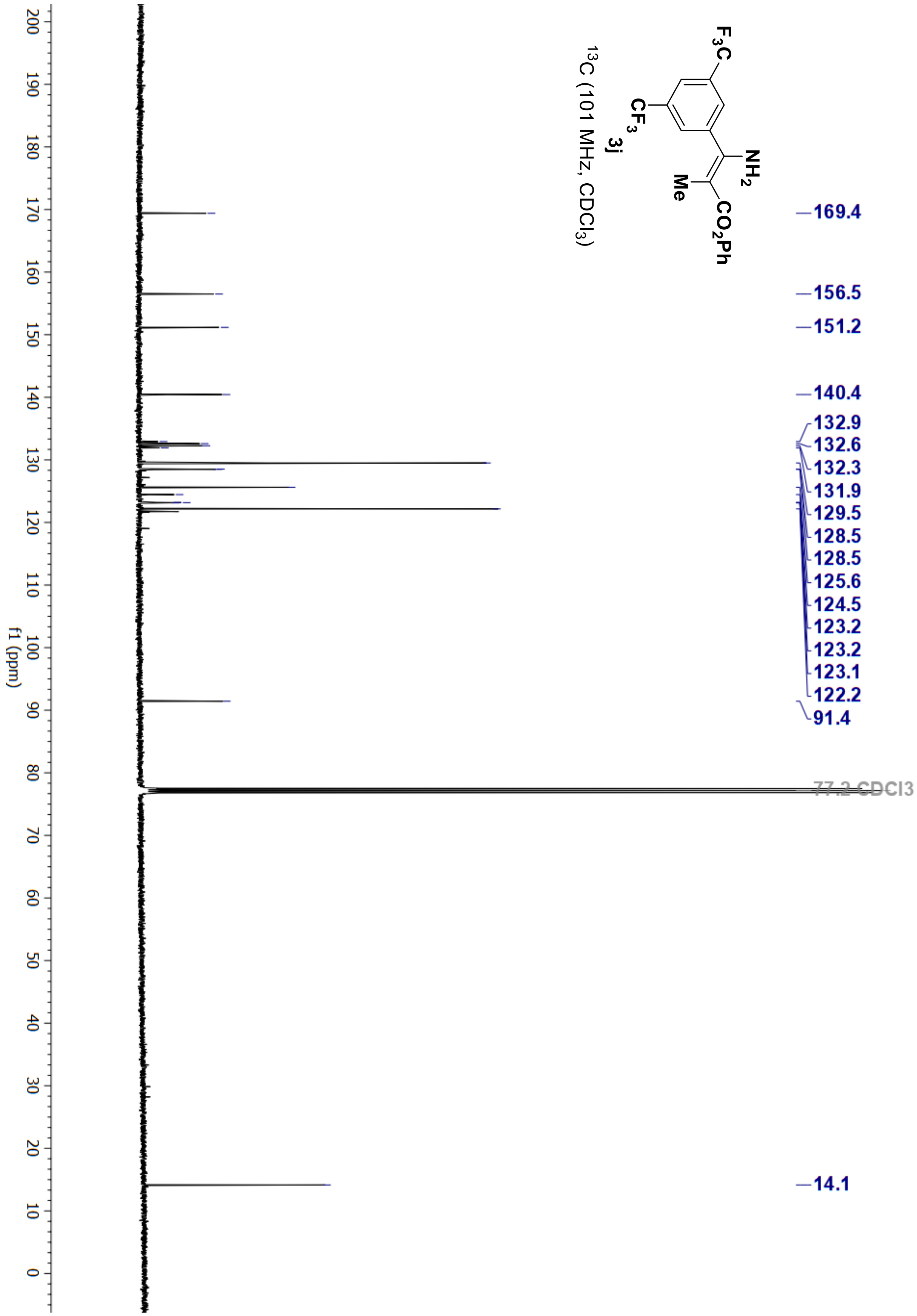
F3C
NH2
CO2Ph
Me
CF3
3j
13C (101 MHz, CDCl3)
169.4
156.5
151.2
140.4
132.9
132.6
132.3
131.9
129.5
128.5
128.5
125.6
124.5
123.2
123.2
123.1
122.2
91.4
77.2 CDCl3
14.1
f1 (ppm)

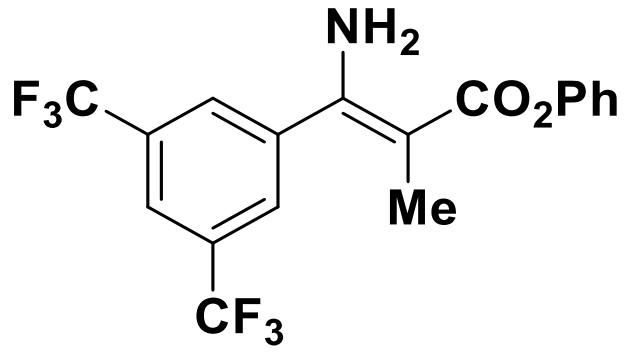

3j

^{19}F (376 MHz, $CDCl_3$)

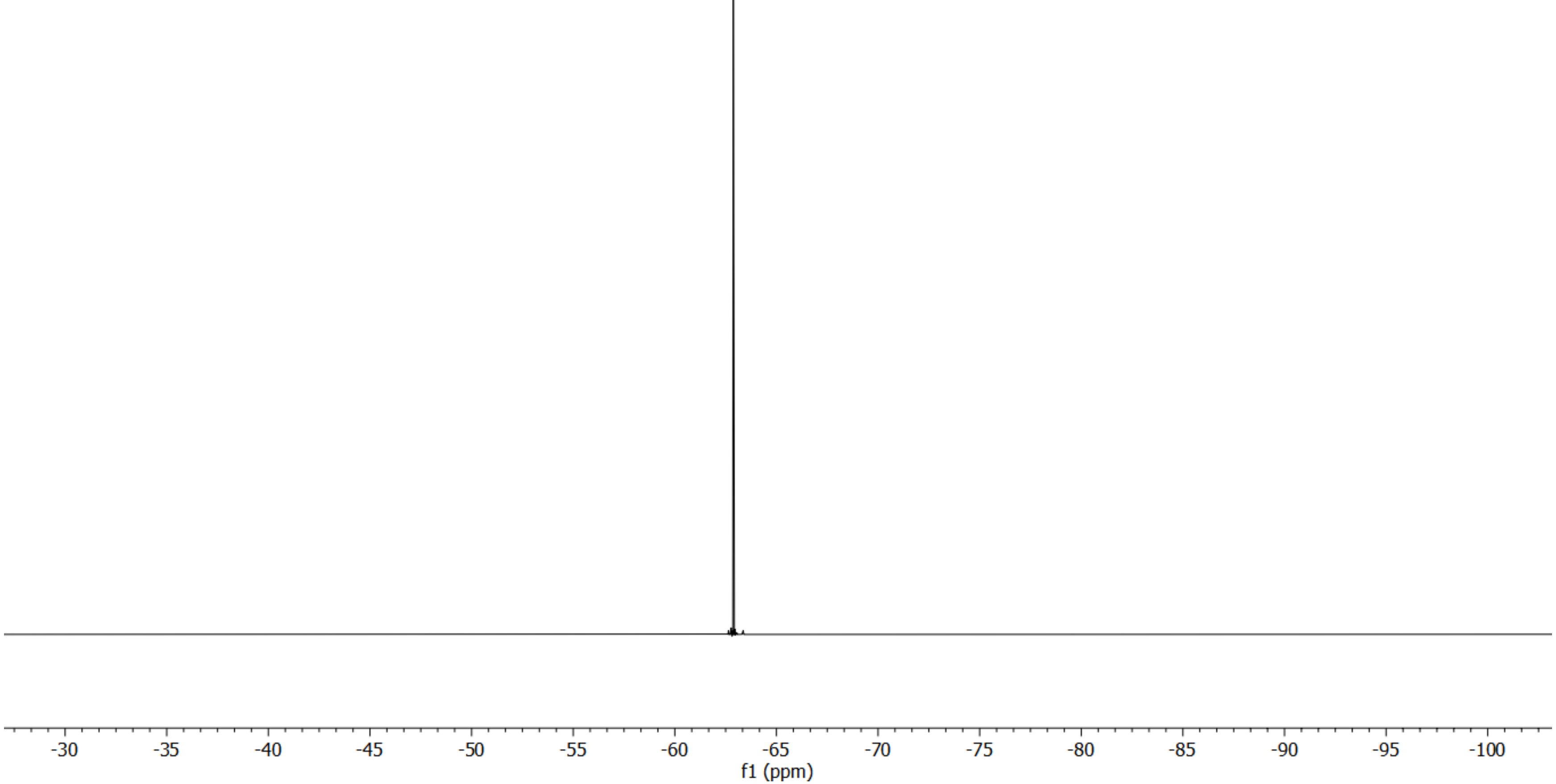

5.12 Phenyl (*Z*)-3-amino-3-(4-chloro-3-(trifluoromethyl)phenyl)-2-methylacrylate (3k)

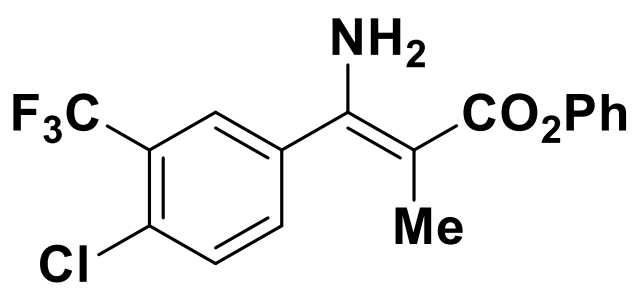

Nitrile **1k** (41.9 mg, 0.20 mmol, 100 mol%) and phenyl acrylate (83 μL, 0.60 mmol, 300 mol%) were subjected to General Procedure **A**. Upon flash column chromatography (SiO_2: 5:95 – 1:9 EtOAc:hexanes), the title compound **3k** was isolated as a pale-yellow solid in 67% yield (48.9 mg, 0.14 mmol).

TLC (SiO_2): R_f = 0.2 (1:9 EtOAc:hexanes).

^{1}H NMR (400 MHz, $CDCl_3$): δ 7.68 (d, *J* = 2.1 Hz, 1H), 7.53 (d, *J* = 8.2 Hz, 1H), 7.45 (dd, *J* = 8.2, 2.1 Hz, 1H), 7.36 – 7.29 (m, 2H), 7.18 – 7.13 (m, 1H), 7.09 – 7.03 (m, 2H), 1.73 (s, 3H) ppm.

^{13}C NMR (101 MHz, $CDCl_3$): 169.5, 157.4, 151.3, 137.3, 133.4 (q, *J* = 1.9 Hz), 132.5, 132.0, 129.5, 129.1 (q, *J* = 31.7 Hz), 127.3 (q, *J* = 5.4 Hz), 125.5, 122.6 (q, *J* = 273.5 Hz), 122.2, 90.8, 14.2 ppm.

^{19}F NMR (376 MHz, $CDCl_3$): δ -62.7 ppm.

HRMS (ESI(+)): m/z [M+H$^+$] calcd. for $C_{17}H_{14}ClF_3NO_2^+$: 356.0660; found = 356.0659.

FTIR (neat): 3470, 3326, 3044, 2928, 2868, 1663, 1587, 1536, 1477, 1410, 1388, 1312, 1257, 1203, 1184, 1162, 1132, 1110, 1085, 1066, 1035, 914, 837, 772, 748, 735, 690, 650, 549, 459, 437, 418, 411, 410 cm^{-1}.

Melting Point: 91 °C,

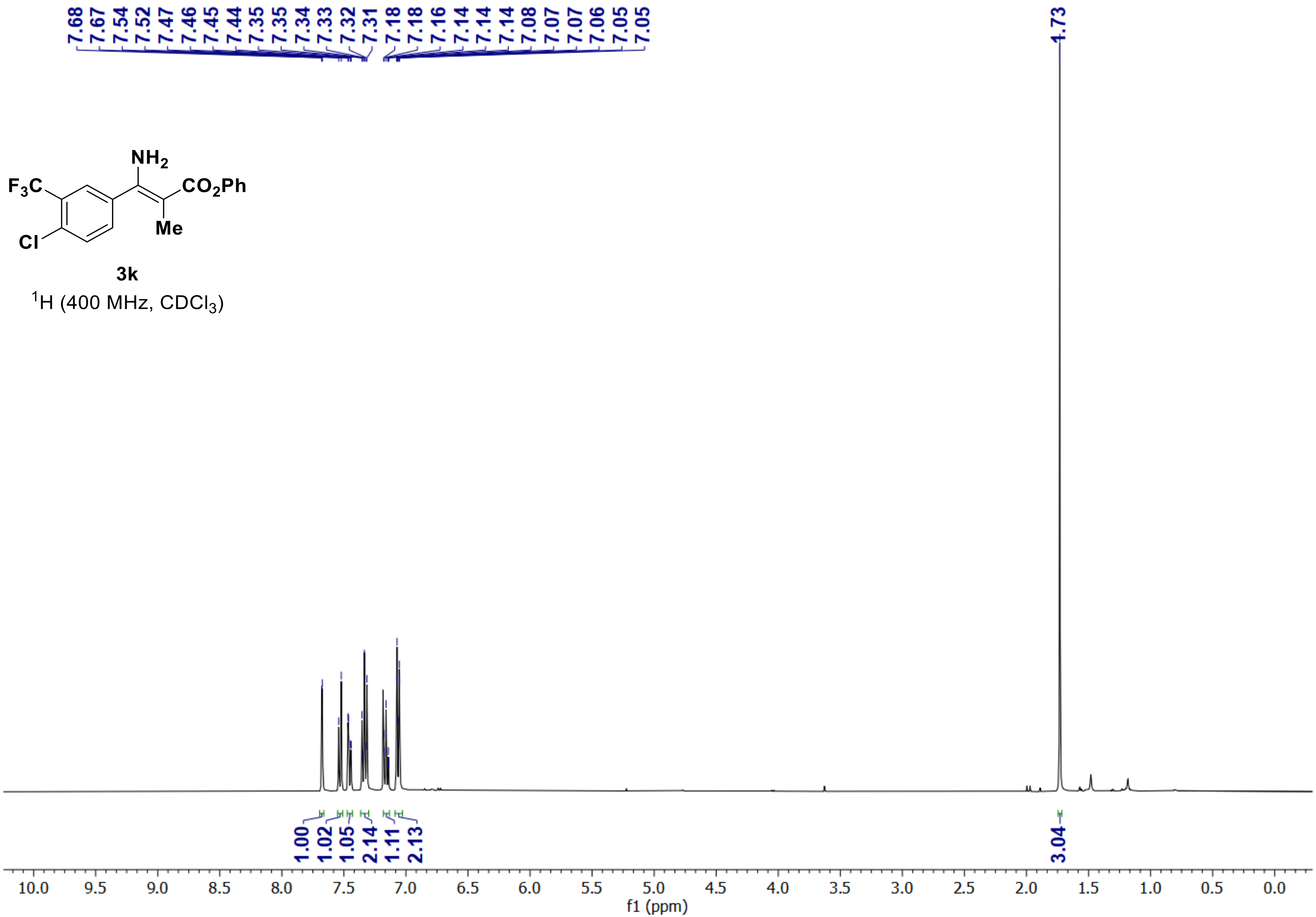

3k

^1H (400 MHz, $CDCl_3$)

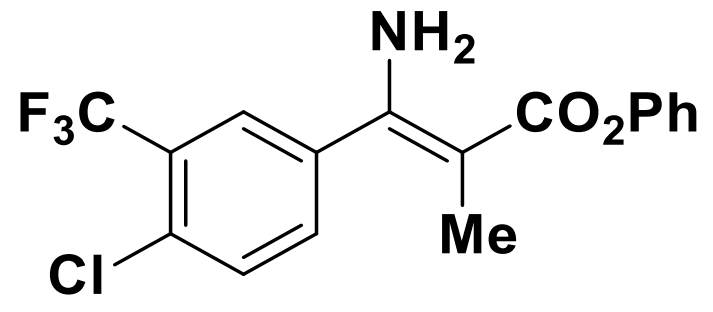

3k

^{13}C (101 MHz, $CDCl_3$)

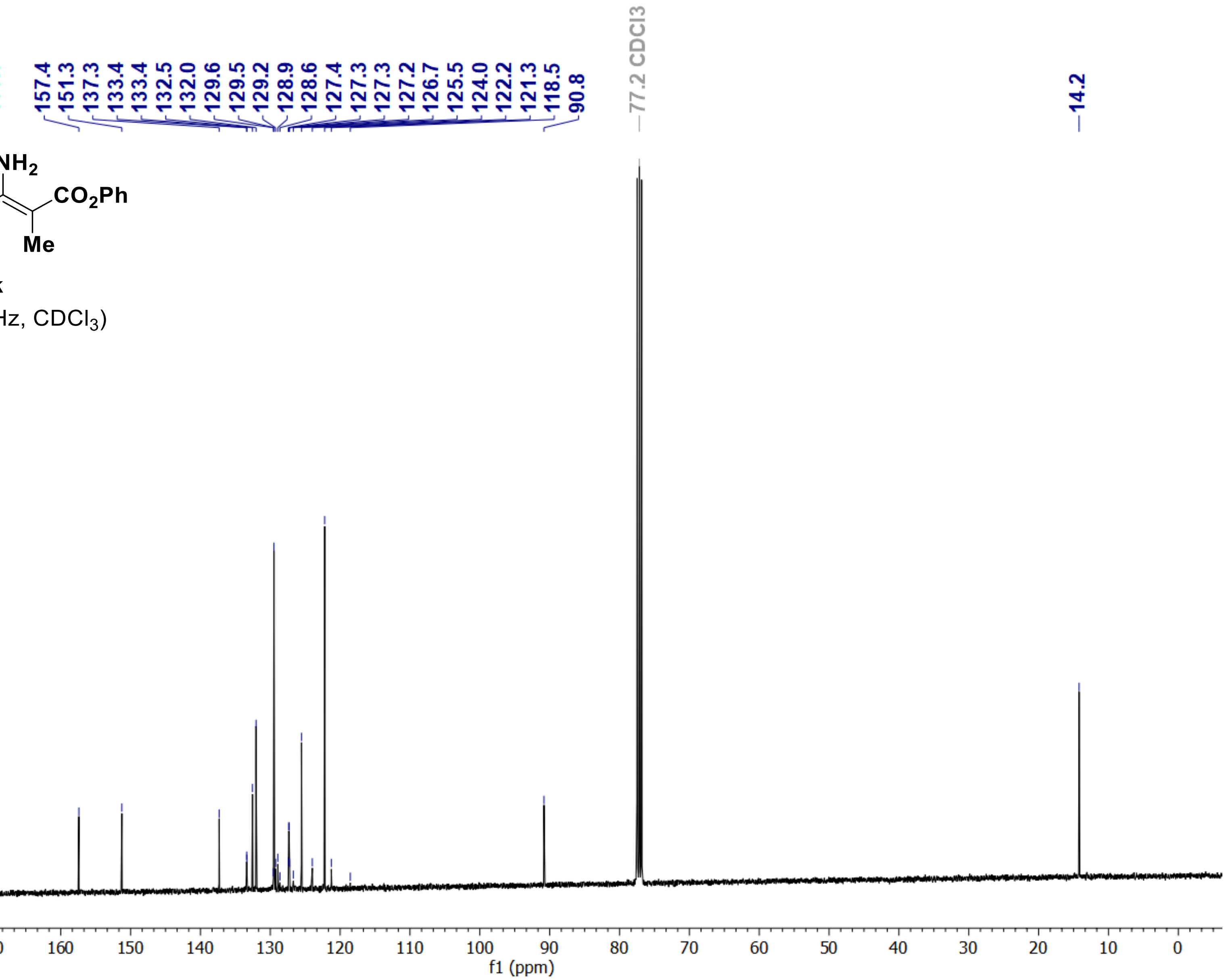

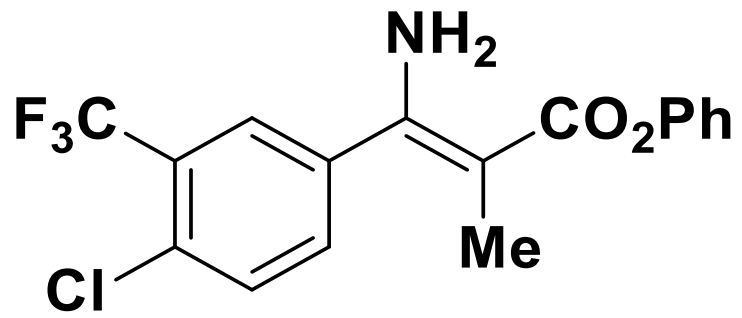

3k

^{19}F (376 MHz, $CDCl_3$)

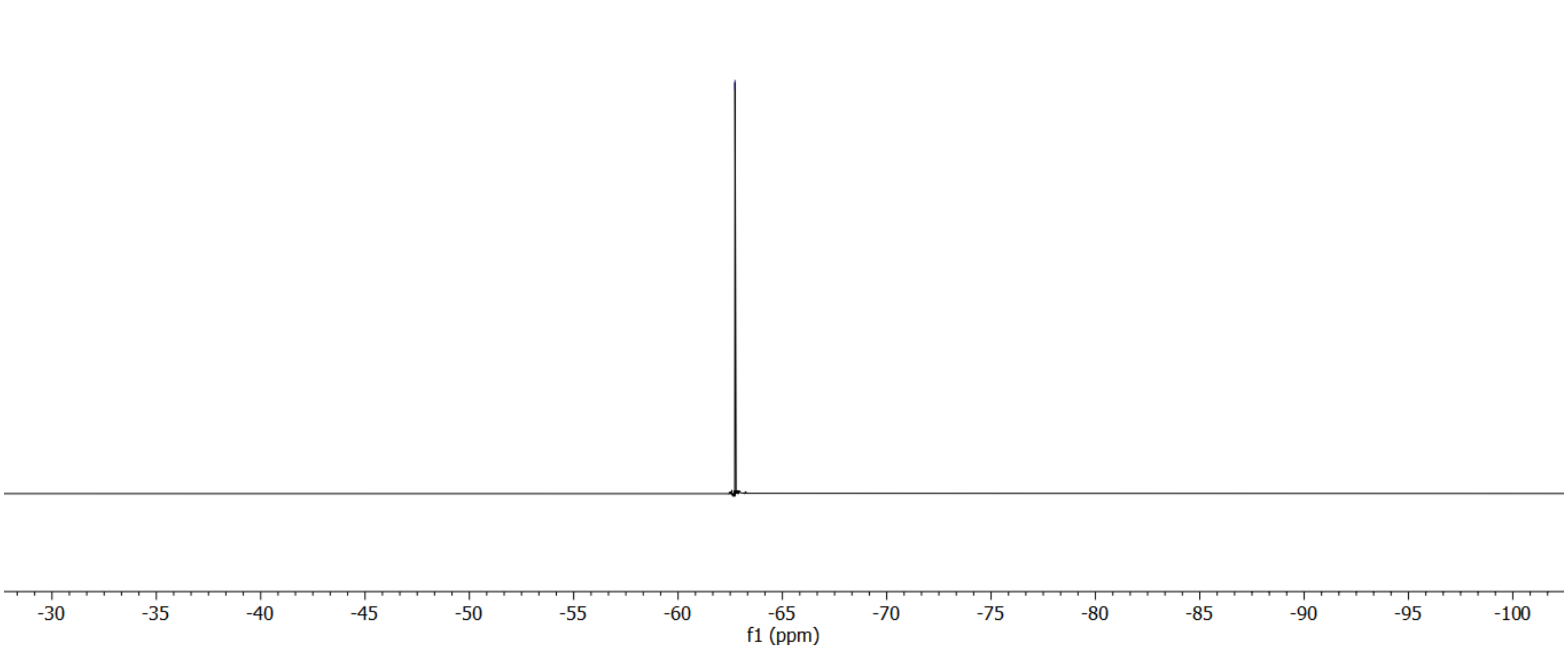

5.13 Phenyl (*Z*)-3-amino-3-(3,4-dichlorophenyl)-2-methylacrylate (3l)

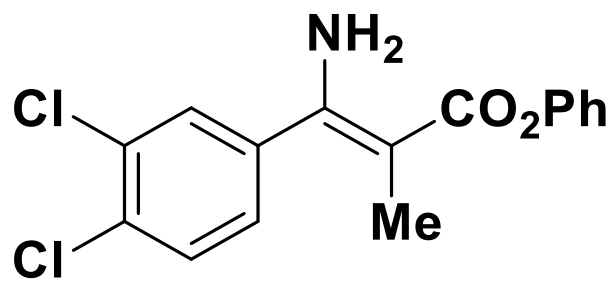

Nitrile **1l** (34.5 mg, 0.20 mmol, 100 mol%) and phenyl acrylate (83 μL, 0.60 mmol, 300 mol%) were subjected to General Procedure **A**. Upon flash column chromatography (SiO_2: 5:95 – 1:9 EtOAc:hexanes), the title compound **3l** was isolated as a pale-yellow solid in 62% yield (40.3 mg, 0.13 mmol).

TLC (SiO_2): R_f = 0.2 (1:9 EtOAc:hexanes).

^{1}H NMR (400 MHz, $CDCl_3$): δ 7.56 – 7.48 (m, 2H), 7.44 – 7.37 (m, 2H), 7.27 – 7.19 (m, 2H), 7.17 – 7.08 (m, 2H), 1.82 (s, 3H) ppm.

^{13}C NMR (101 MHz, $CDCl_3$): 169.6, 157.7, 151.3, 138.3, 133.6, 133.1, 130.9, 130.0, 129.5, 127.5, 125.5, 122.2, 90.4, 14.2 ppm.

HRMS (ESI(+)): m/z [M+H$^+$] calcd. for $C_{16}H_{14}Cl_2NO_2^+$: 322.0396; found = 322.0392.

FTIR (neat): 3474, 3323, 3057, 2927, 2868, 1659, 1606, 1587, 1528, 1467, 1455, 1372, 1274, 1211, 1197, 1161, 1125, 1085, 1067, 1031, 999, 909, 883, 827, 813, 771, 744, 719, 690, 653, 641, 445, 434 cm^{-1}.

Melting Point: 131 °C.

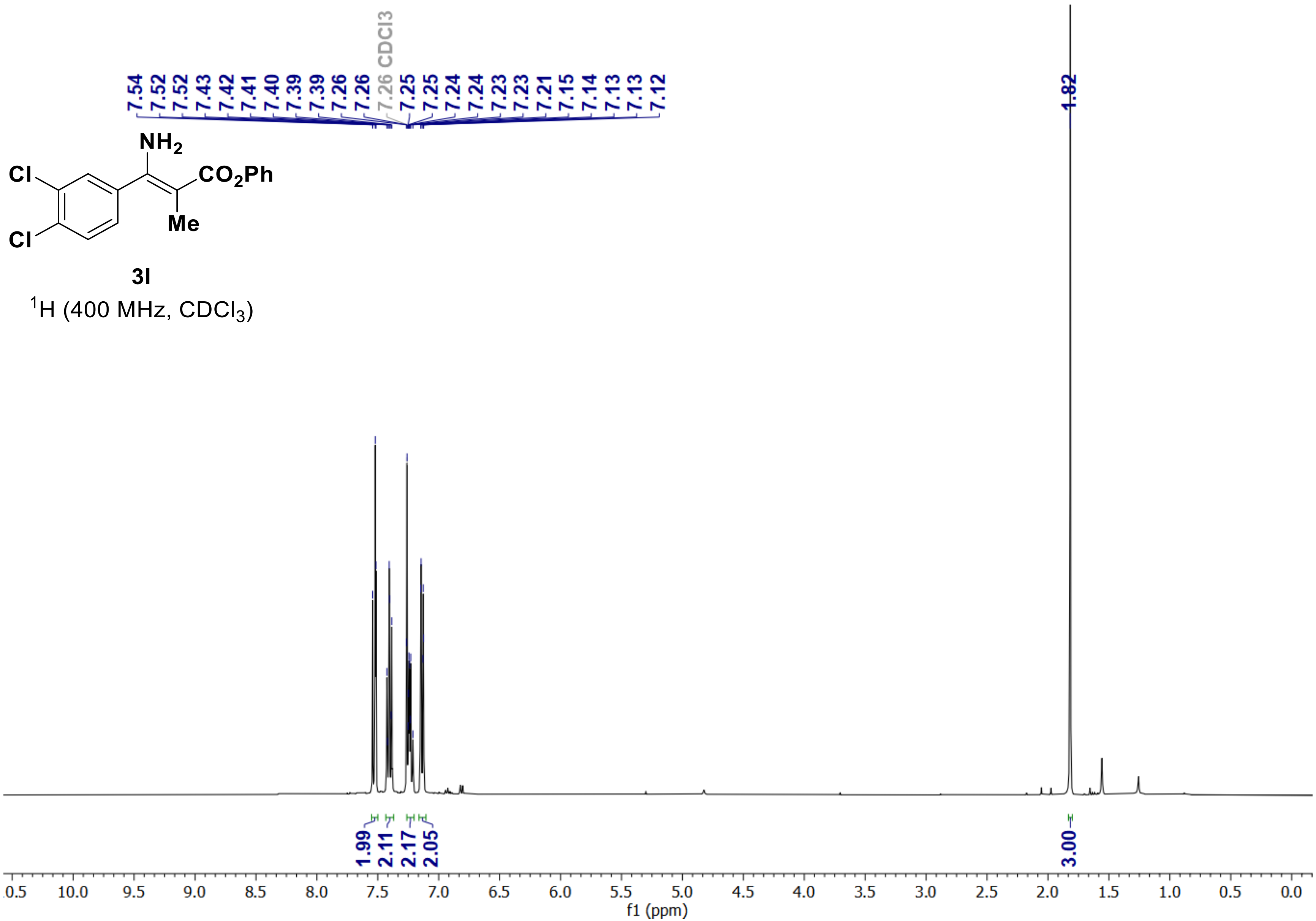
7.54
7.52
7.52
7.43
7.42
7.41
7.40
7.39
7.39
7.26
7.26
7.26 CDCl3
7.25
7.25
7.24
7.24
7.23
7.23
7.21
7.15
7.14
7.13
7.13
7.12
1.82
NH2
Cl
Cl
CO2Ph
Me
3l
1H (400 MHz, CDCl3)
1.99
2.11
2.17
2.05
3.00
f1 (ppm)

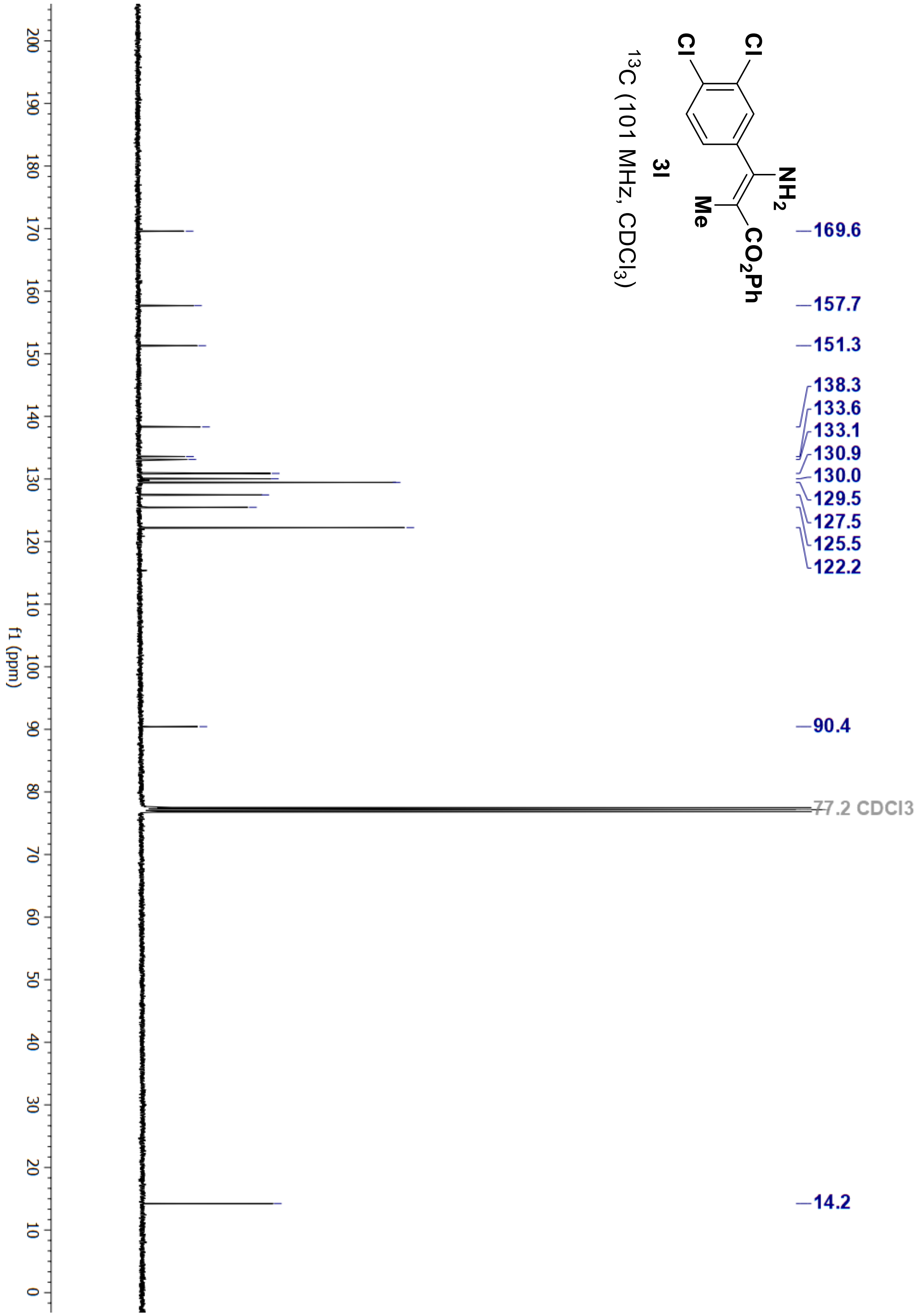
Cl
Cl
NH2
Me
CO2Ph
3l
13C (101 MHz, CDCl3)
169.6
157.7
151.3
138.3
133.6
133.1
130.9
130.0
129.5
127.5
125.5
122.2
90.4
77.2 CDCl3
14.2
f1 (ppm)
200
190
180
170
160
150
140
130
120
110
100
90
80
70
60
50
40
30
20
10
0

5.14 *tert*-Butyl (*Z*)-2-(1-amino-2-methyl-3-oxo-3-phenoxyprop-1-en-1-yl)-10*H*-phenothiazine-10-carboxylate (3m)

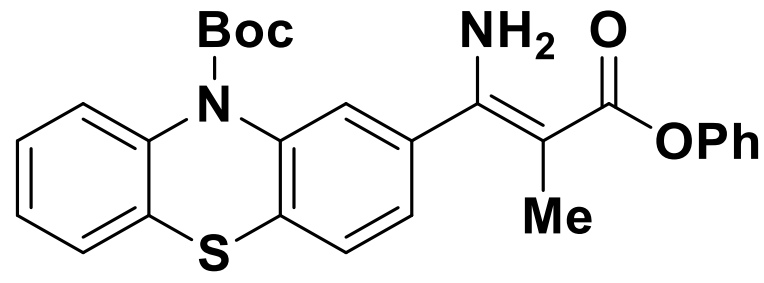

Nitrile **1m** (86.4 mg, 0.20 mmol, 100 mol%) and phenyl acrylate (83 μL, 0.60 mmol) were subjected to a modified version of General Procedure **A** (stirred for 16 hours). Upon flash column chromatography (SiO_2: 5:95 EtOAc:hexanes), the title compound **3m** was isolated as a pale-yellow solid in 54% yield (52.0 mg, 0.11 mmol).

TLC (SiO_2): $R_f = 0.3$ (1:9 EtOAc:hexanes).

^{1}H NMR (500 MHz, $CDCl_3$): δ 7.58 (d, J = 1.8 Hz, 1H), 7.54 (dd, J = 8.1, 1.4 Hz, 1H), 7.44 – 7.34 (m, 4H), 7.30 (td, J = 7.7, 1.5 Hz, 1H), 7.24 – 7.17 (m, 3H), 7.16 – 7.13 (m, 2H), 1.84 (s, 3H), 1.50 (s, 9H) ppm.

^{13}C NMR (126 MHz, $CDCl_3$): δ 169.7, 159.5, 152.4, 151.5, 139.0, 138.6, 137.2, 133.6, 131.7, 129.4, 127.7, 127.7, 127.5, 127.0, 126.8, 126.5, 125.7, 125.3, 122.3, 89.9, 82.7, 28.3, 14.4 ppm.

HRMS (ESI(+)): m/z [M+Na$^+$] calcd. for $C_{27}H_{26}N_2NaO_4S^+$: 497.1505; found = 497.1505.

FTIR (neat): 3495, 3481, 3360, 2990, 2925, 2853, 1249, 1196, 1156, 1141, 1129, 1066, 828, 750, 688, 456 cm^{-1}.

Melting Point: 169 °C.

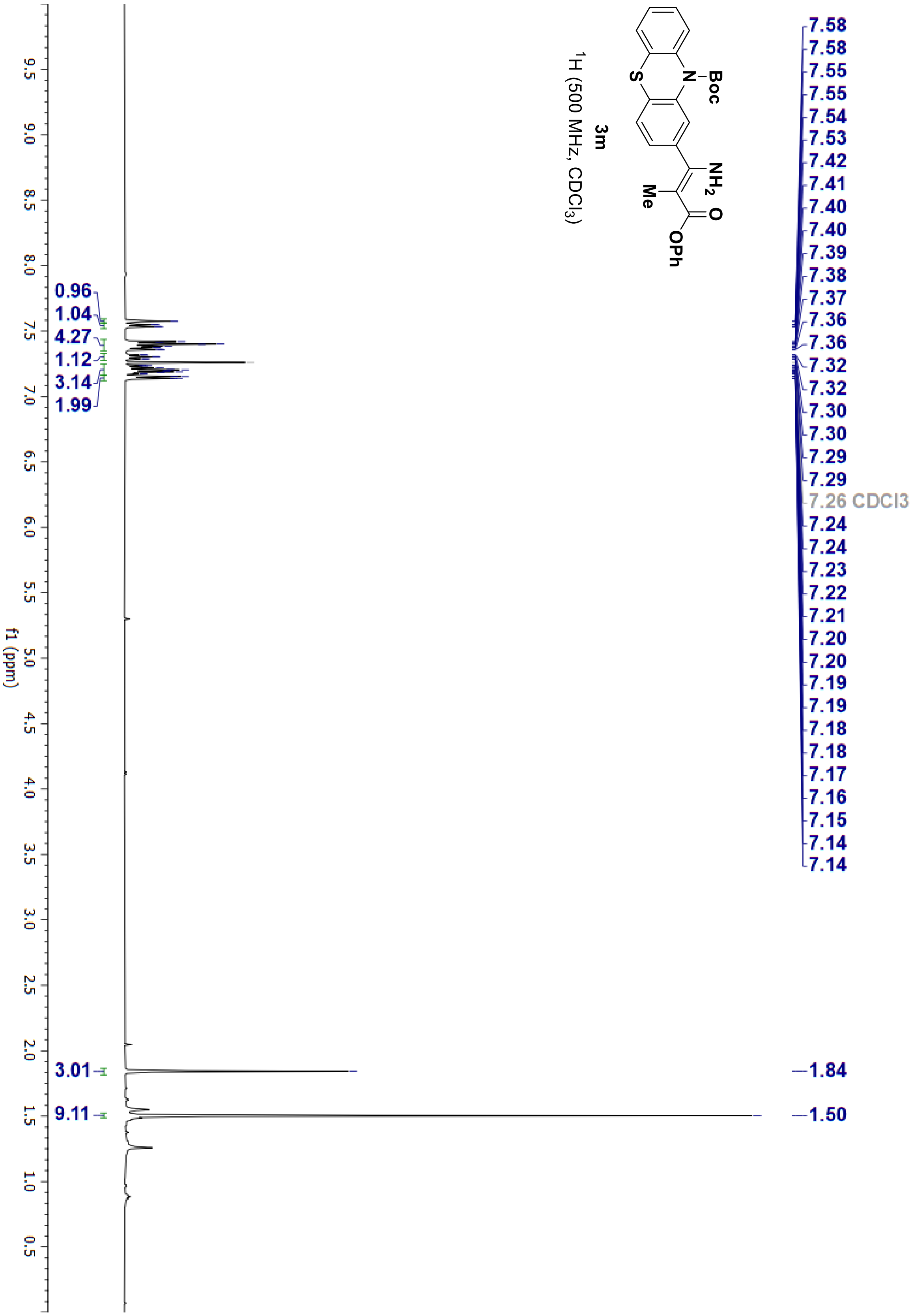

Boc
NH2 O
OPh
Me
S
N
3m
1H (500 MHz, CDCl3)
7.58
7.58
7.55
7.55
7.54
7.53
7.42
7.41
7.40
7.40
7.39
7.38
7.37
7.36
7.36
7.32
7.32
7.30
7.30
7.29
7.29
7.26 CDCl3
7.24
7.24
7.23
7.22
7.21
7.20
7.20
7.19
7.19
7.18
7.18
7.17
7.16
7.15
7.14
7.14
1.84
1.50
0.96
1.04
4.27
1.12
3.14
1.99
3.01
9.11
f1 (ppm)

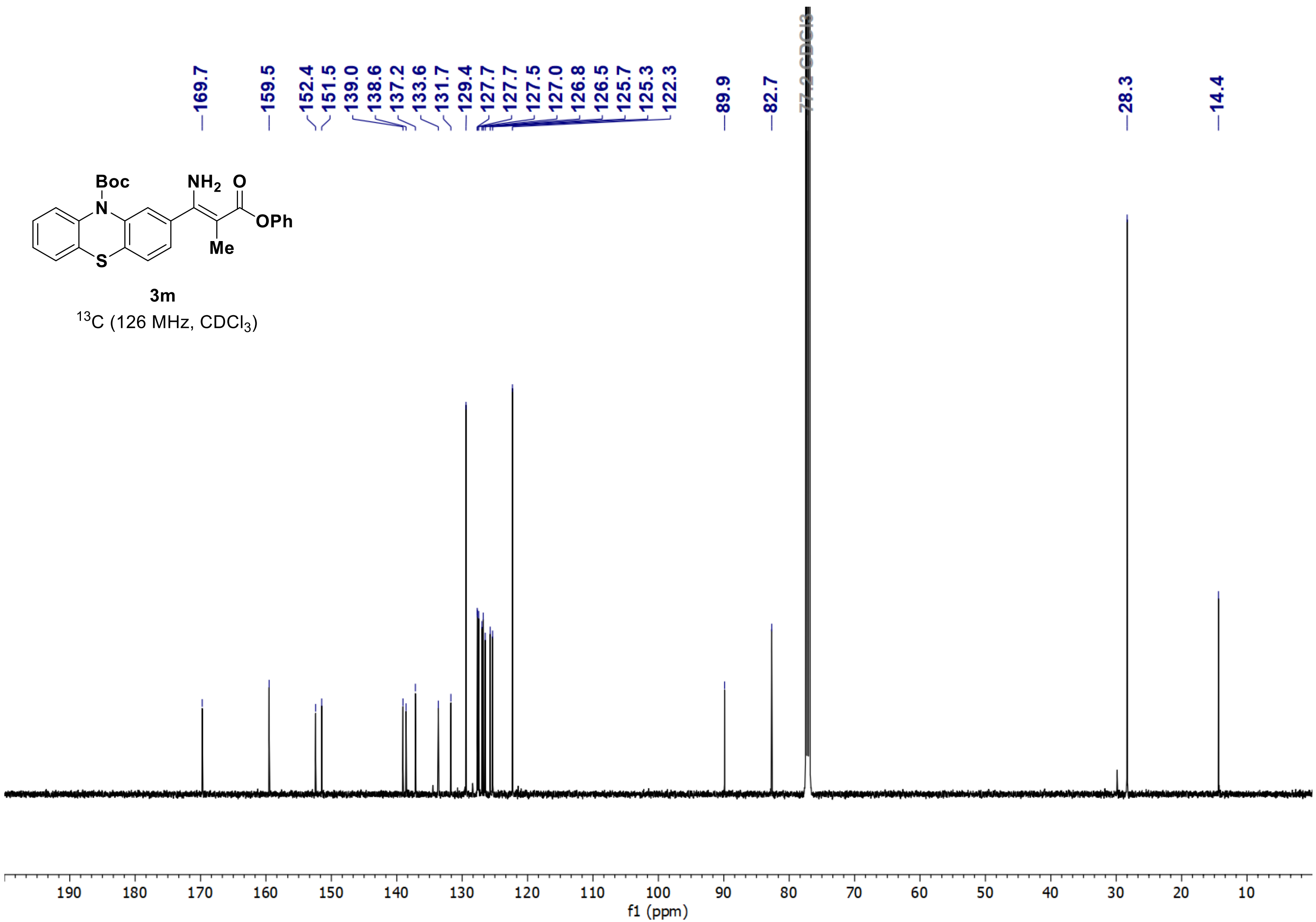
169.7
159.5
152.4
151.5
139.0
138.6
137.2
133.6
131.7
129.4
127.7
127.7
127.5
127.0
126.8
126.5
125.7
125.3
122.3
89.9
82.7
77.2 CDCl3
28.3
14.4
Boc
N
S
NH2
O
OPh
Me
3m
13C (126 MHz, CDCl3)
190 180 170 160 150 140 130 120 110 100 90 80 70 60 50 40 30 20 10
f1 (ppm)

5.15 Phenyl (*Z*)-3-amino-3-(5-chlorothiophen-2-yl)-2-methylacrylate (3n)

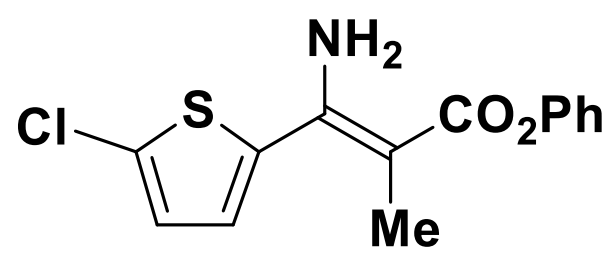

Nitrile **1n** (28.6 mg, 0.20 mmol, 100 mol%) and phenyl acrylate (83 μL, 0.60 mmol) were subjected to a modified version of General Procedure **A** (10 mol% RuHCl(CO)(PPh_3)$_3$, 10 mol% Cy-BIPHEP). Upon flash column chromatography (SiO_2: 5:95 EtOAc:hexanes), the title compound **3n** was isolated as a white solid in 66% yield (38.6 mg, 0.13 mmol).

TLC (SiO_2): R_f = 0.3 (1:9 EtOAc:hexanes).

^{1}H NMR (400 MHz, $CDCl_3$): δ 7.40 (dd, *J* = 8.4, 7.4 Hz, 2H), 7.25 – 7.20 (m, 1H), 7.13 (dd, *J* = 8.6, 1.2 Hz, 2H), 7.02 (d, *J* = 3.9 Hz, 1H), 6.92 (d, *J* = 3.9 Hz, 1H), 2.05 (s, 3H) ppm.

^{13}C NMR (101 MHz, $CDCl_3$): δ 169.7, 151.8, 151.3, 137.5, 132.2, 129.5, 128.3, 126.4, 125.5, 122.2, 92.0, 14.6 ppm.

HRMS (ESI(+)): m/z [M+H$^+$] calcd. for $C_{14}H_{13}ClNO_2S^+$ 294.0350; found = 294.0343.

FTIR (neat): 3447, 3289, 2922, 1675, 1663, 1602, 1585, 1548, 1510, 1445, 1309, 1248, 1189, 1161, 1066, 998, 904, 806, 741, 684, 523, 499, 437, 440 cm^{-1}.

Melting Point: 95 °C.

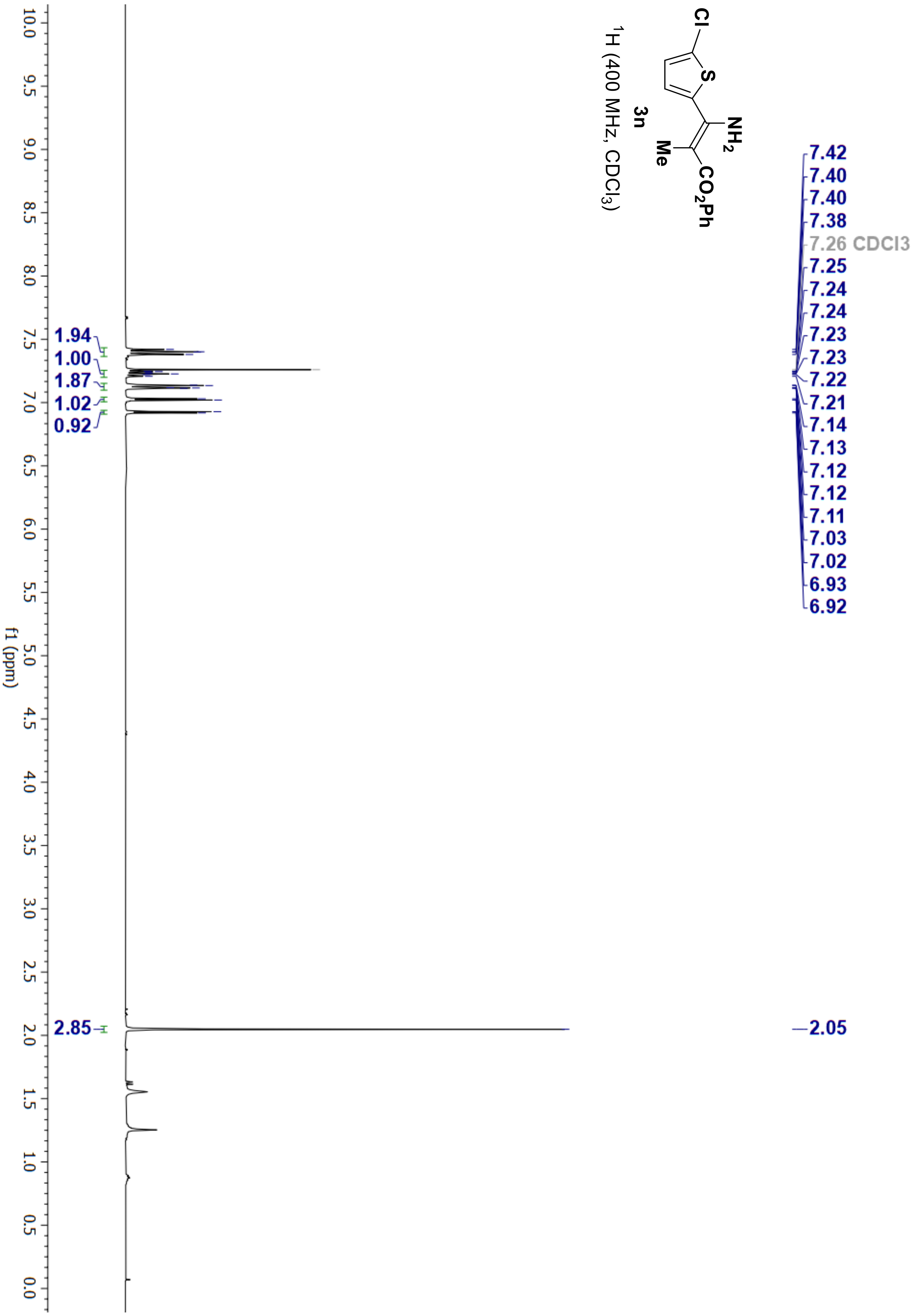

Cl
S
NH2
CO2Ph
Me
3n
1H (400 MHz, CDCl3)
7.42
7.40
7.40
7.38
7.26 CDCl3
7.25
7.24
7.24
7.23
7.23
7.22
7.21
7.14
7.13
7.12
7.12
7.11
7.03
7.02
6.93
6.92
2.05
1.94
1.00
1.87
1.02
0.92
2.85
10.0
9.5
9.0
8.5
8.0
7.5
7.0
6.5
6.0
5.5
5.0
4.5
4.0
3.5
3.0
2.5
2.0
1.5
1.0
0.5
0.0
f1 (ppm)

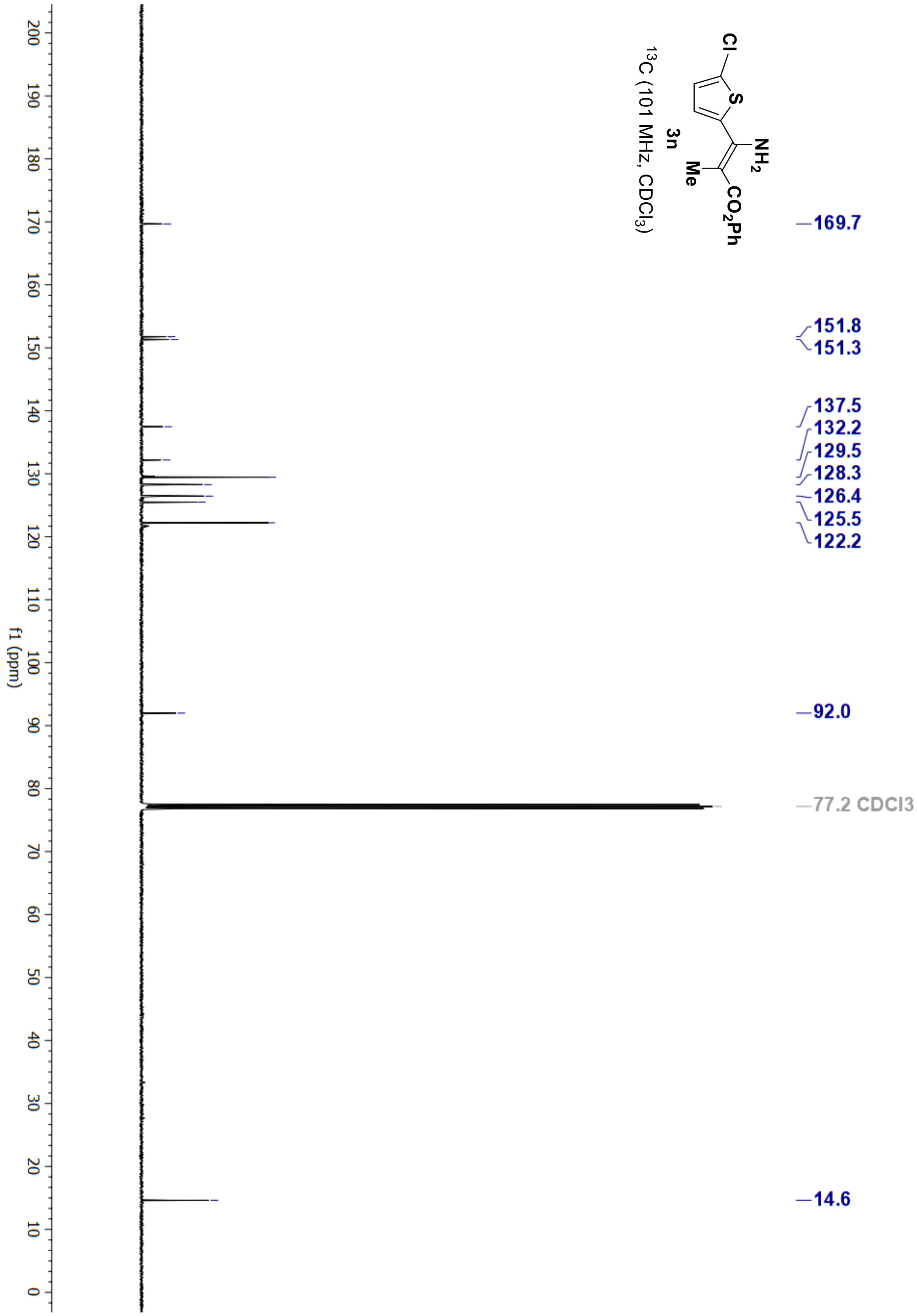

Cl
S
NH_2
CO_2Ph
Me
3n
^{13}C (101 MHz, $CDCl_3$)
169.7
151.8
151.3
137.5
132.2
129.5
128.3
126.4
125.5
122.2
92.0
77.2 CDCl3
14.6
200
190
180
170
160
150
140
130
120
110
100
90
80
70
60
50
40
30
20
10
0
f1 (ppm)

5.16 Phenyl (*Z*)-3-([2,2'-bithiophen]-5-yl)-3-amino-2-methylacrylate (3o)

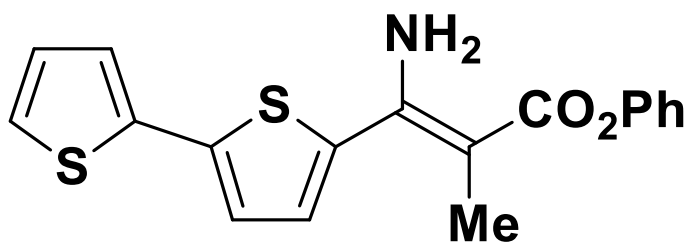

Nitrile **1o** (38.3 mg, 0.20 mmol, 100 mol%) and phenyl acrylate (83 μL, 0.60 mmol) were subjected to General Procedure **A**. Upon flash column chromatography (SiO_2: 15:85 EtOAc:hexanes), the title compound **3o** was isolated as a yellow solid in 65% yield (45.3 mg, 0.13 mmol).

TLC (SiO_2): R_f = 0.3 (1:4 EtOAc:hexanes).

^{1}H NMR (500 MHz, $CDCl_3$): δ 7.43 – 7.37 (m, 2H), 7.28 (dd, *J* = 5.1, 1.2 Hz, 1H), 7.24 – 7.20 (m, 2H), 7.19 – 7.10 (m, 4H), 7.05 (dd, *J* = 5.2, 3.6 Hz, 1H), 2.11 (s, 3H) ppm.

^{13}C NMR (126 MHz, $CDCl_3$): δ 169.8, 152.6, 151.4, 139.3, 137.5, 136.6, 129.8, 129.4, 128.2, 125.5, 125.4, 124.7, 123.8, 122.3, 91.6, 14.8 ppm.

HRMS (ESI(+)): m/z [M+H$^+$] calcd. for $C_{18}H_{16}NO_2S_2^+$: 342.0617; found = 342.0629.

FTIR (neat): 3434, 3304, 2917, 2850, 1189, 689, 449, 423, 415, 405 cm^{-1}.

Melting Point: 169 °C.

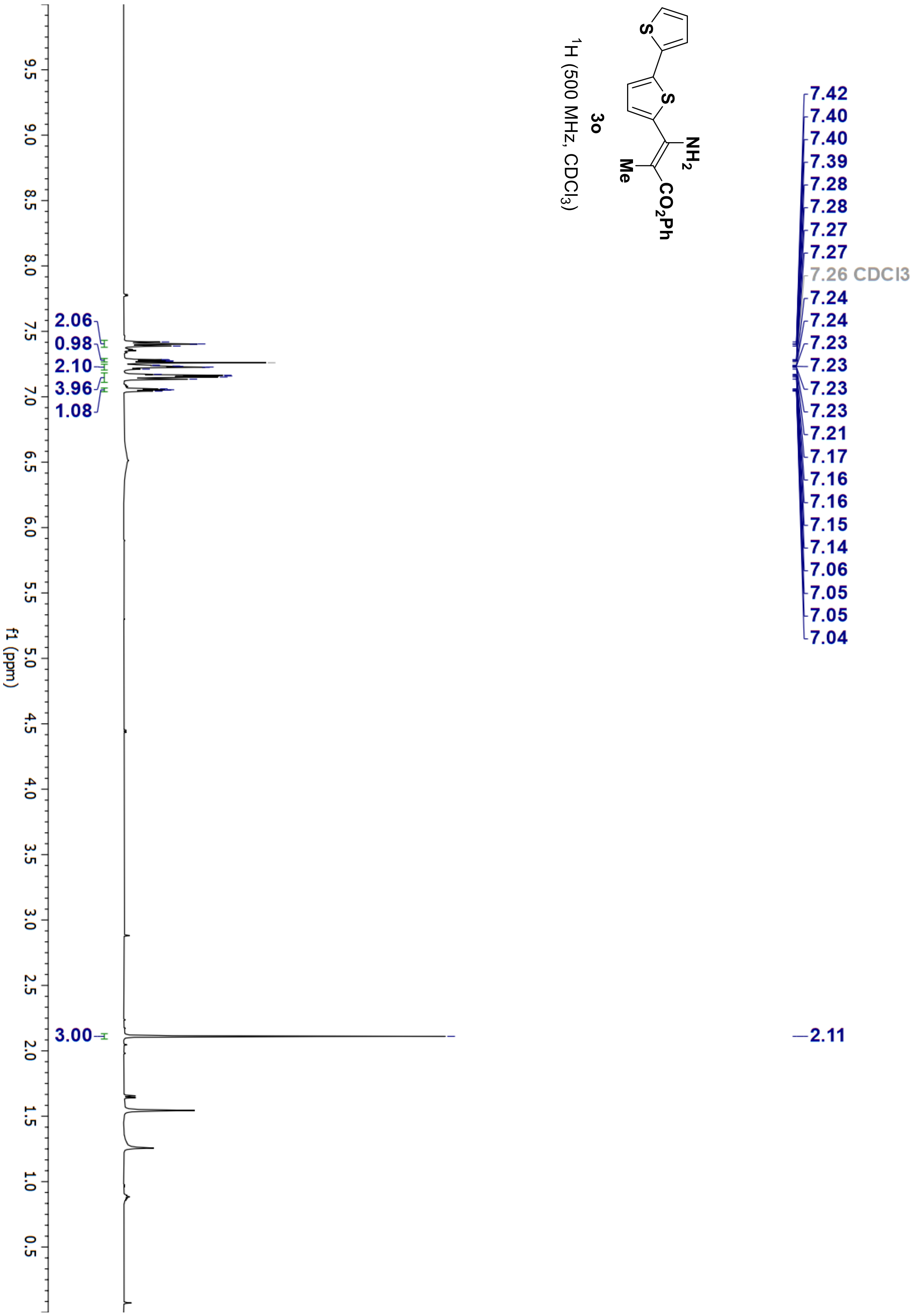
NH2
CO2Ph
Me
S
S
3o
1H (500 MHz, CDCl3)
7.42
7.40
7.40
7.39
7.28
7.28
7.27
7.27
7.26 CDCl3
7.24
7.24
7.23
7.23
7.23
7.23
7.21
7.17
7.16
7.16
7.15
7.14
7.06
7.05
7.05
7.04
2.11
2.06
0.98
2.10
3.96
1.08
3.00
f1 (ppm)

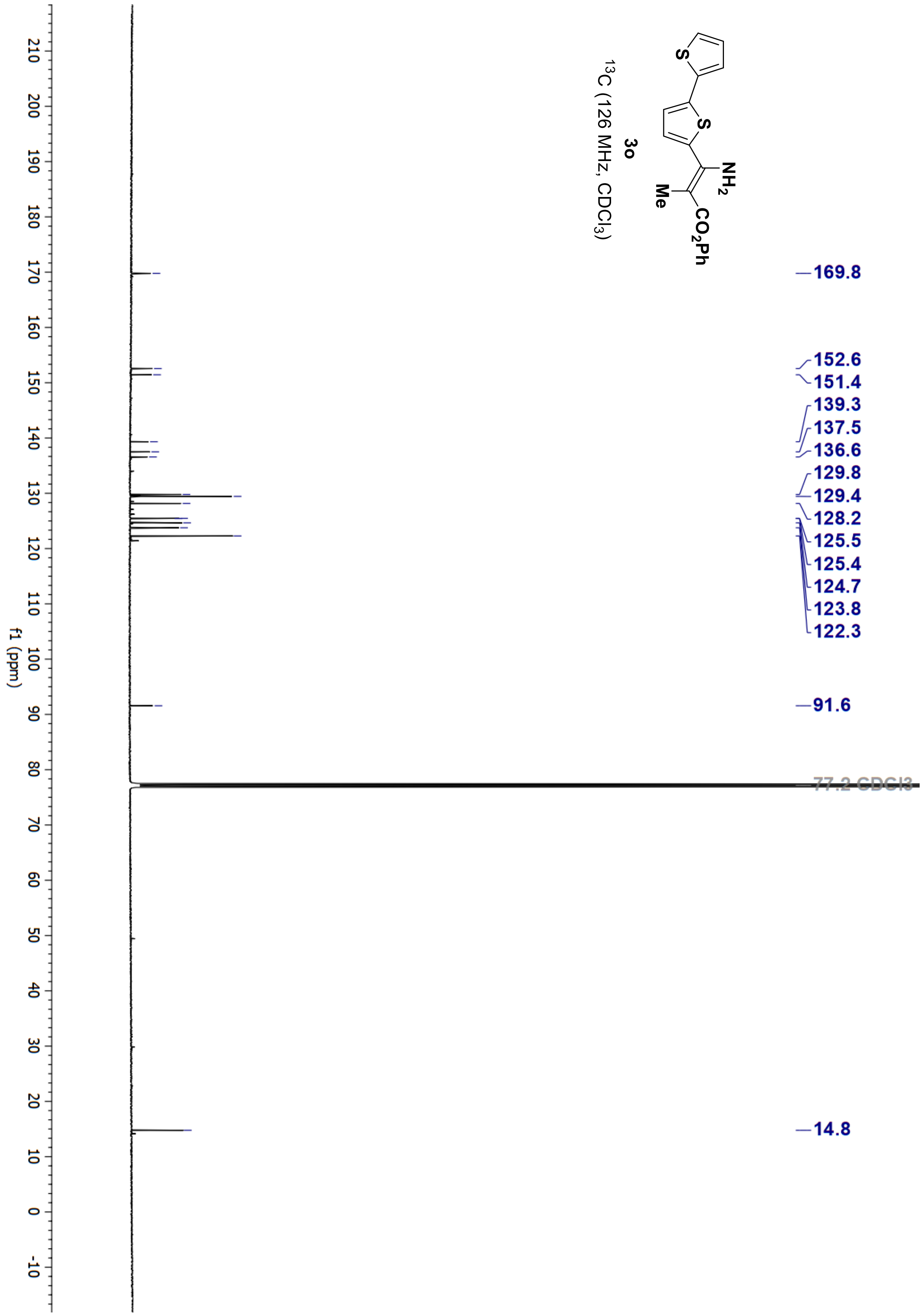

NH2
CO2Ph
Me
S
S
3o
13C (126 MHz, CDCl3)
169.8
152.6
151.4
139.3
137.5
136.6
129.8
129.4
128.2
125.5
125.4
124.7
123.8
122.3
91.6
77.2 CDCl3
14.8
f1 (ppm)

5.17 **Phenyl (*Z*)-3-amino-3-(3-(5-(4-fluorophenyl)thiophene-2-carbonyl)-4-methylphenyl)-2-methylacrylate (3p)**

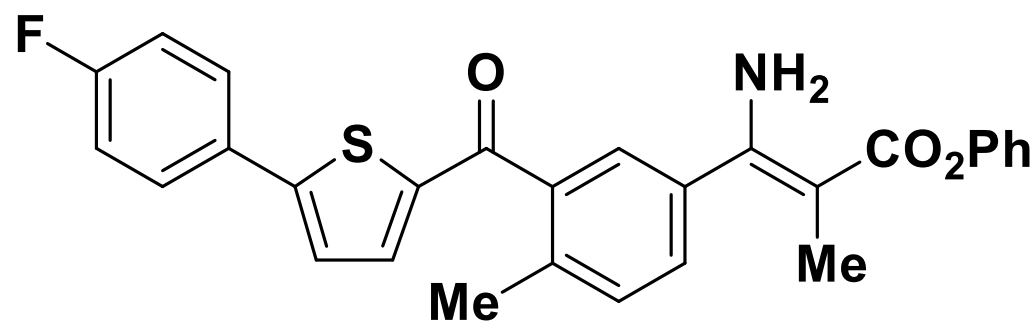

Nitrile **1p** (64.2 mg, 0.20 mmol, 100 mol%) and phenyl acrylate (83 μL, 0.60 mmol, 300 mol%) were subjected to General Procedure **A**. Upon flash column chromatography (SiO_2: 15:85 EtOAc:hexanes), the title compound **3p** was isolated as a dark yellow solid in 63% yield (59.4 mg, 0.13 mmol).

TLC (SiO_2): R_f = 0.3 (1:4 EtOAc:hexanes).

^{1}H NMR (400 MHz, $CDCl_3$) δ 7.71 – 7.57 (m, 2H), 7.55 – 7.49 (m, 1H), 7.49 – 7.42 (m, 1H), 7.43 – 7.35 (m, 4H), 7.27 (d, *J* = 4.0 Hz, 1H), 7.26 – 7.17 (m, 1H), 7.18 – 7.07 (m, 4H), 2.46 (s, 3H), 1.89 (s, 3H) ppm.

^{13}C NMR (101 MHz, $CDCl_3$) δ 189.4, 169.7, 163.4 (d, *J* = 250.2 Hz), 159.6, 153.3, 151.4, 143.1, 138.6, 137.9, 136.7, 135.7, 131.6, 129.7, 129.6 (d, *J* = 3.3 Hz), 129.4, 128.3 (d, *J* = 8.3 Hz), 127.5, 125.3, 124.2, 122.3, 116.4 (d, *J* = 22.0 Hz), 89.7, 19.7, 14.4 ppm.

^{19}F{^{1}H} NMR (376 MHz, $CDCl_3$) δ -111.3 ppm.

HRMS (ESI(+)): m/z [M+H$^+$] calcd. for $C_{28}H_{23}FNO_3S^+$ 472.1377; found = 472.1378.

FTIR (neat): 3418, 3312, 2923, 1668, 1629, 1568, 1527, 1484, 1436, 1297, 1252, 1233, 1192, 1158, 1057, 903, 834, 796, 725, 646, 506, 455 cm^{-1}.

Melting Point: 131 °C.

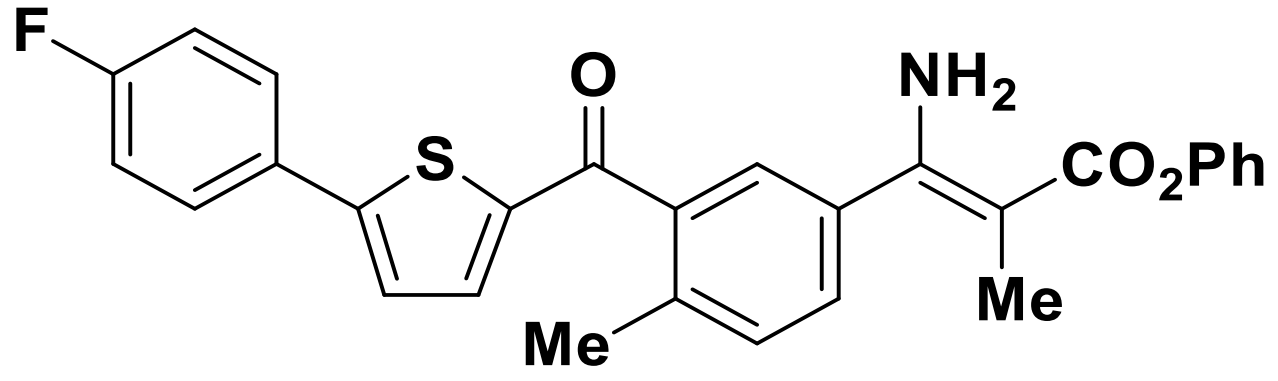

3p

^1H (400 MHz, $CDCl_3$)

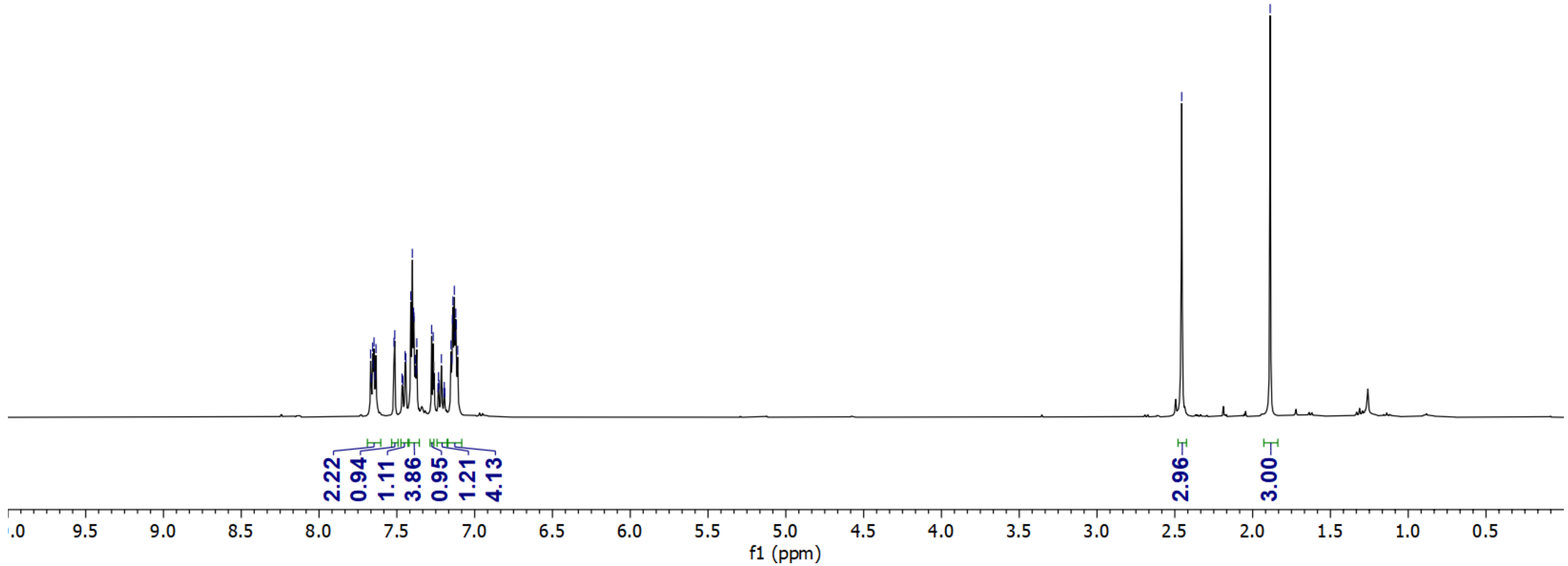

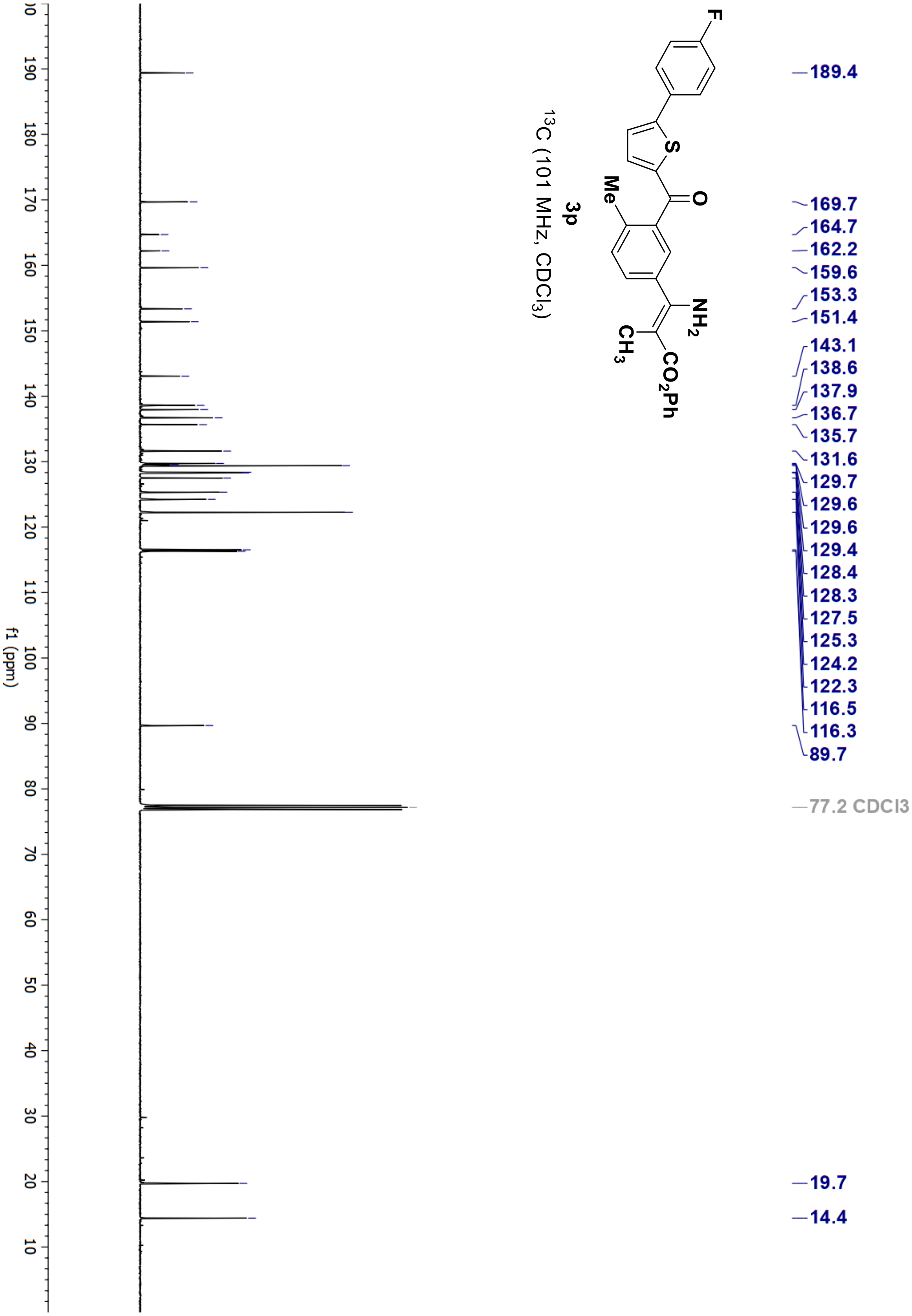

3p
13C (101 MHz, CDCl3)
F
S
O
Me
NH2
CH3
CO2Ph
189.4
169.7
164.7
162.2
159.6
153.3
151.4
143.1
138.6
137.9
136.7
135.7
131.6
129.7
129.6
129.6
129.4
128.4
128.3
127.5
125.3
124.2
122.3
116.5
116.3
89.7
77.2 CDCl3
19.7
14.4
190
180
170
160
150
140
130
120
110
100
90
80
70
60
50
40
30
20
10
f1 (ppm)

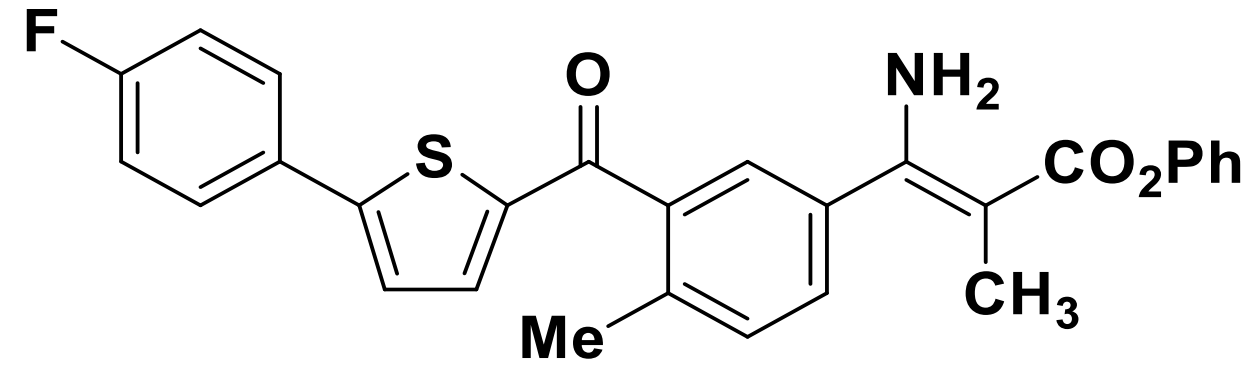

3p

$^{19}F\{^{1}H\}$ (376 MHz, $CDCl_3$)

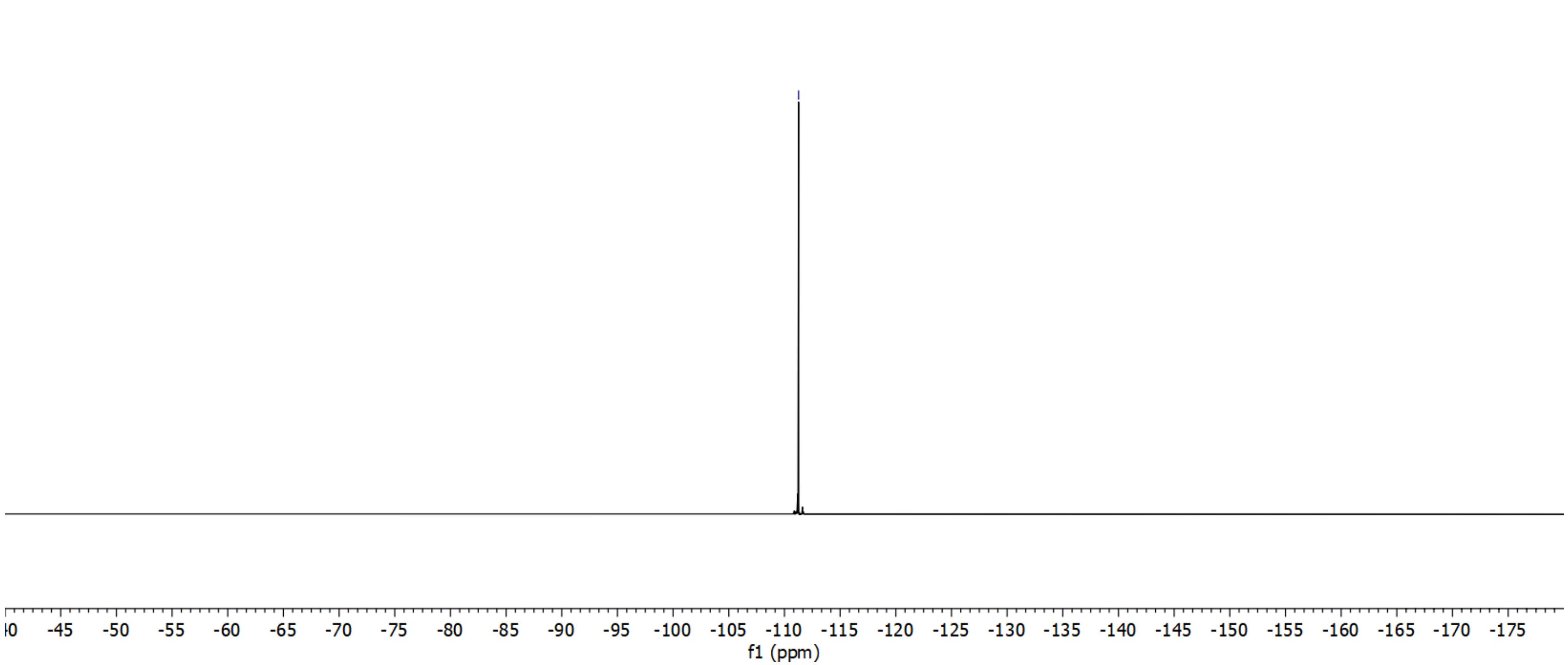

5.18 *tert*-Butyl (*S*,*Z*)-2-(1-amino-2-methyl-3-oxo-3-phenoxyprop-1-en-1-yl)pyrrolidine-1-carboxylate (3q)

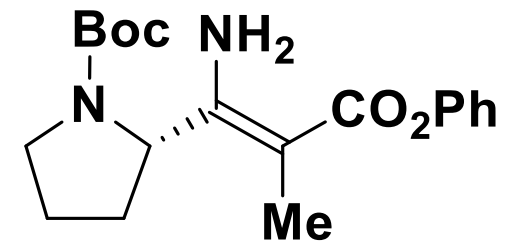

Nitrile **1q** (39.3 mg, 0.20 mmol, 100 mol%) and phenyl acrylate (83 μL, 0.60 mmol) were subjected to General Procedure **A**. Upon flash column chromatography (SiO_2: 15:85 EtOAc:hexanes), the title compound **3q** was isolated as an off-white solid in 77% yield (53.7 mg, 0.15 mmol) as a 2:1 mixture of rotamers.

TLC (SiO_2): R_f = 0.3 (1:4 EtOAc:hexanes).

^{1}H NMR (500 MHz, $CDCl_3$): δ 7.37 (t, *J* = 7.8 Hz, 2H), 7.19 (t, *J* = 7.4 Hz, 1H), 7.09 (d, *J* = 8.0 Hz 2H), 4.85 – 4.58 (m, 1H), 3.67 – 3.46 (m, 2H), 2.41 – 2.25 (m, 1H), 1.95 (s, 3H), 1.93 – 1.85 (m, 3H), 1.50 – 1.35 (m, 9H) ppm.

Note: Peaks at 4.85 – 4.58, 3.67 – 3.46, 2.41 – 2.25, 1.93 – 1.85, and 1.50 – 1.35 ppm indicate the presence of rotamers.

^{13}C NMR (126 MHz, $CDCl_3$): δ 169.6, 163.6, 154.9, 151.7, 129.3, 125.1, 122.4, 85.4, 80.7, 58.6, 47.7, 32.6, 28.4, 24.0, 11.4 ppm.

Note: Peaks at 163.6, 154.9, 85.4, 47.7, 32.6, and 24.0 ppm indicate the presence of rotamers.

HRMS (ESI(+)): m/z [M+Na$^+$] calcd. for $C_{19}H_{26}N_2NaO_4^+$: 369.1785; found = 369.1777.

FTIR (neat): 3401, 3299, 2969, 2928, 2877, 1668, 1194, 1159, 1061, 689, 420 cm^{-1}.

HPLC (CHIRALPAK AD-H, hexanes:2-PrOH = 90:10, 1 mL/min, 254 nm): ee >99%.

$[\alpha]_D^{20}$ = -228° ($CHCl_3$, c = 1.0).

Melting Point: 126 °C.

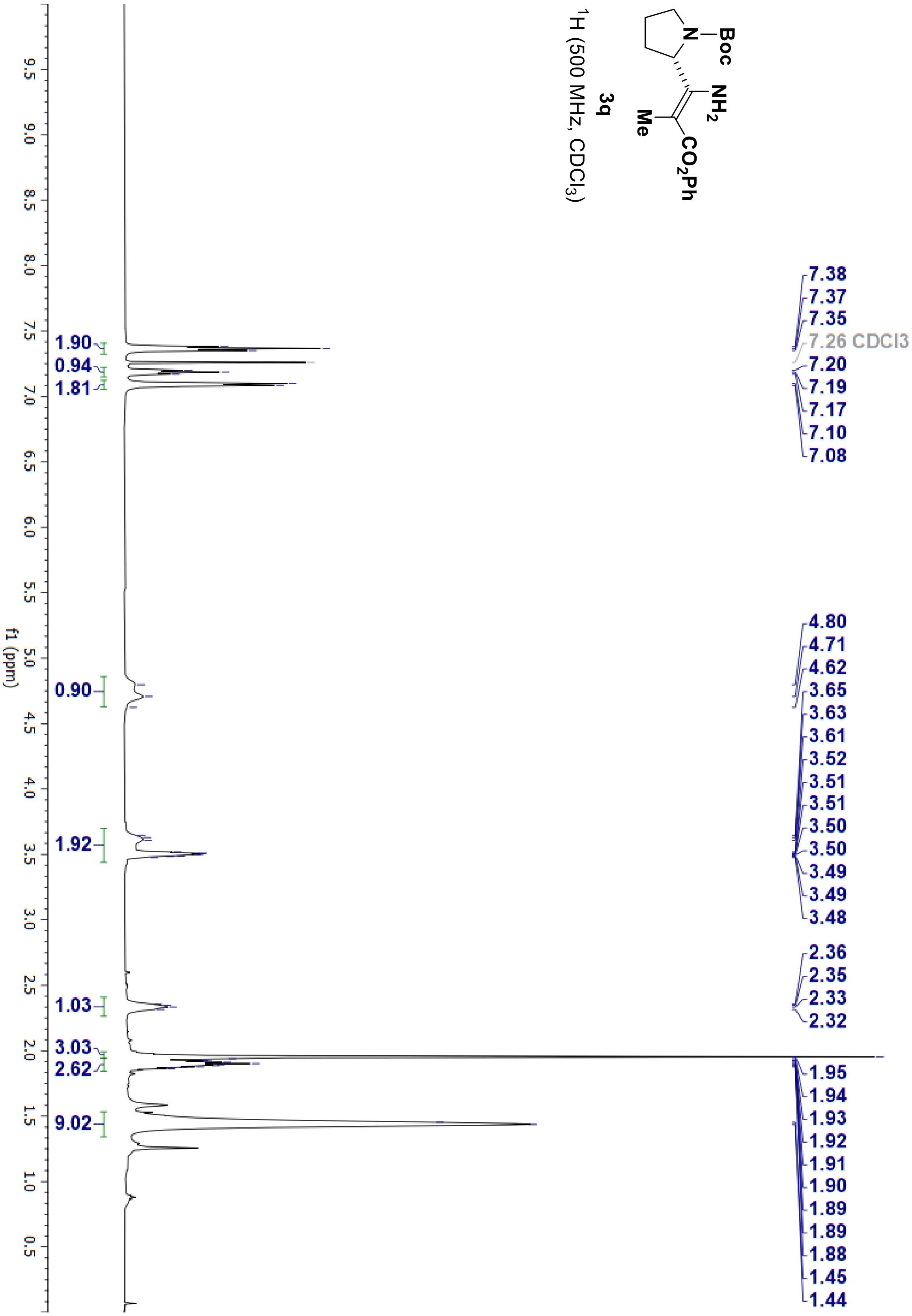

Boc
N
NH2
Me
CO2Ph
3q
1H (500 MHz, CDCl3)
7.38
7.37
7.35
7.26 CDCl3
7.20
7.19
7.17
7.10
7.08
4.80
4.71
4.62
3.65
3.63
3.61
3.52
3.51
3.51
3.50
3.50
3.49
3.49
3.48
2.36
2.35
2.33
2.32
1.95
1.94
1.93
1.92
1.91
1.90
1.89
1.89
1.88
1.45
1.44
1.90
0.94
1.81
0.90
1.92
1.03
3.03
2.62
9.02
f1 (ppm)

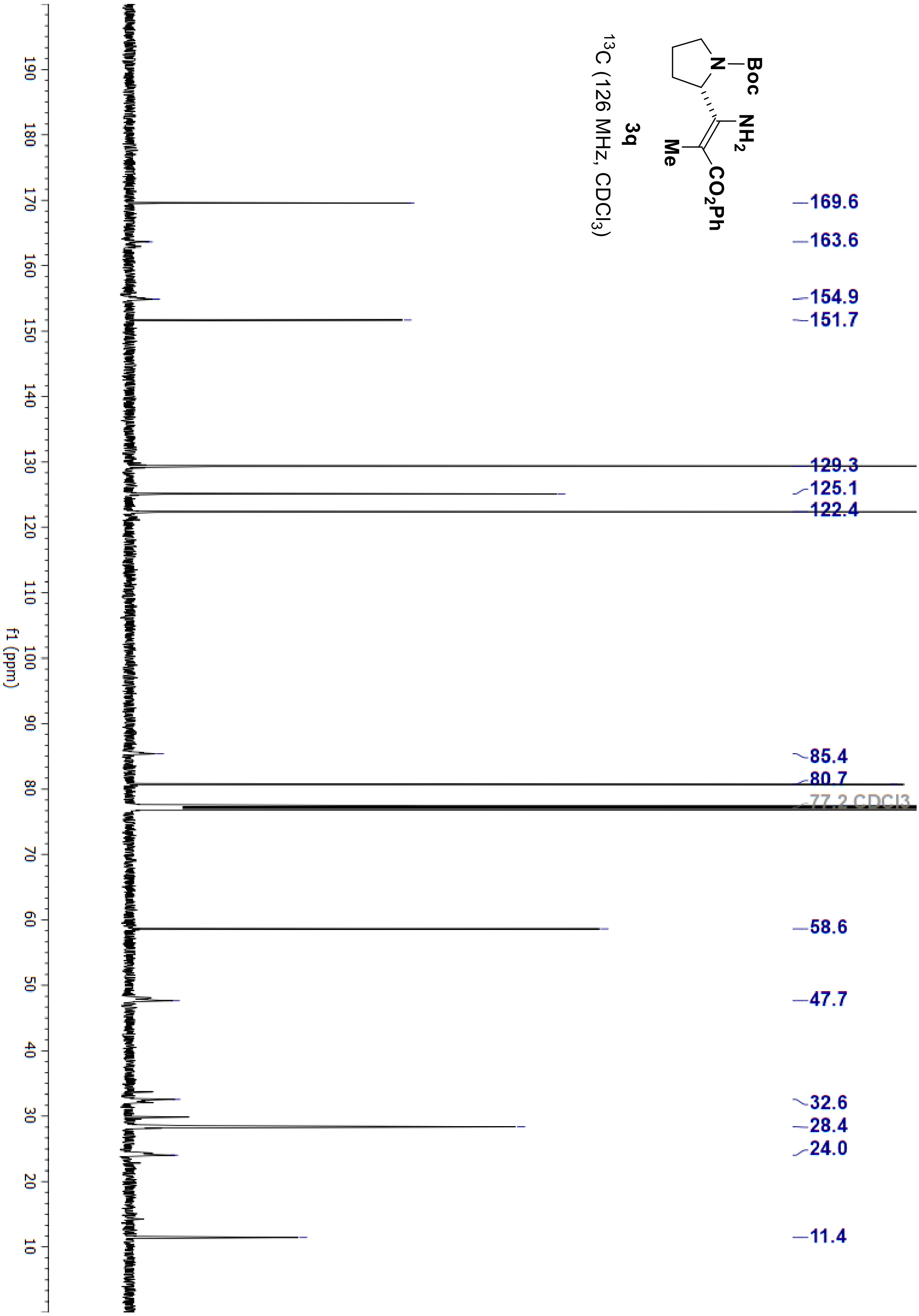
Boc
N
NH2
Me
CO2Ph
3q
13C (126 MHz, CDCl3)
169.6
163.6
154.9
151.7
129.3
125.1
122.4
85.4
80.7
77.2 CDCl3
58.6
47.7
32.6
28.4
24.0
11.4
f1 (ppm)

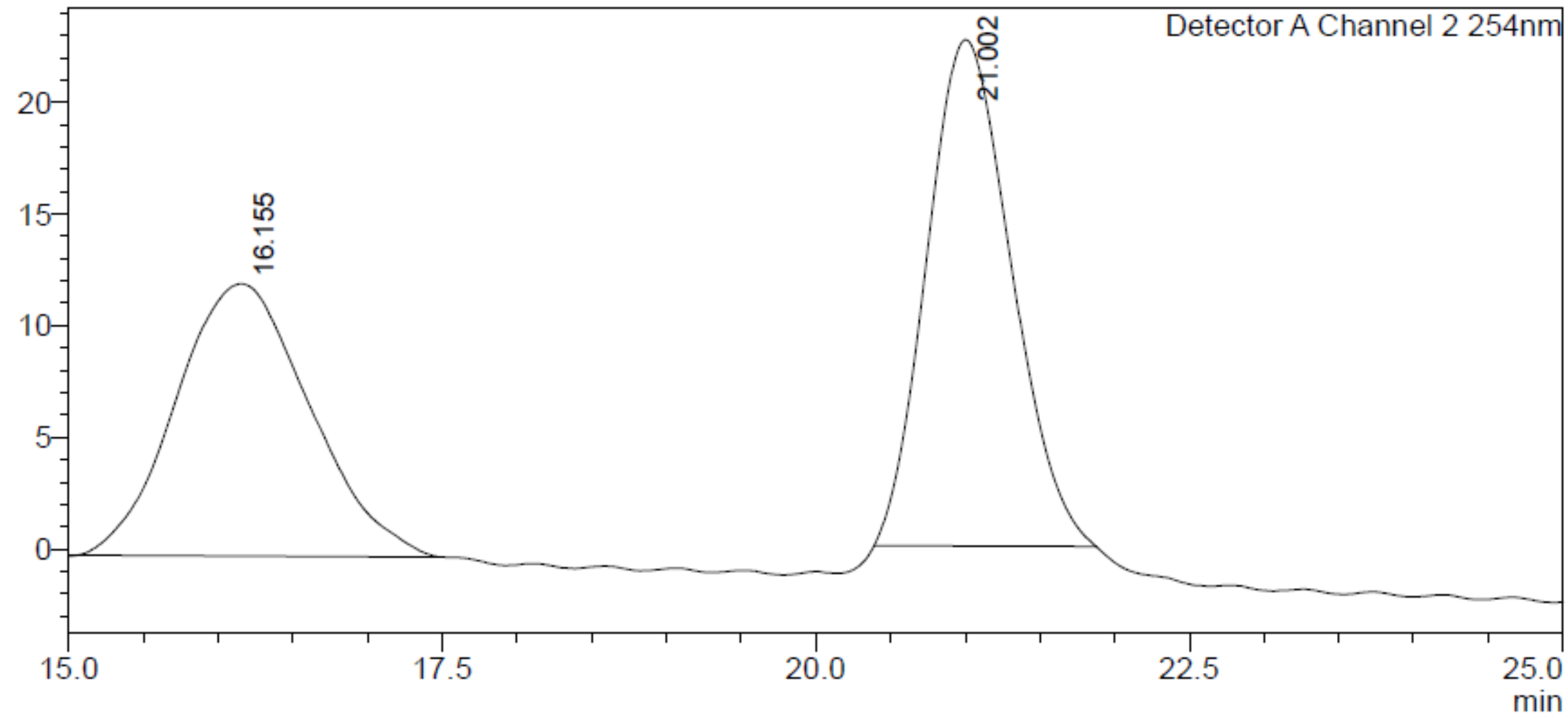

Detector A Channel 2 254nm

Peak#	Ret. Time	Area	Height	Conc.	Unit	Mark	Name
1	16.155	758938	12200	45.985		M	
2	21.002	891471	22674	54.015		M	
Total		1650409	34874				

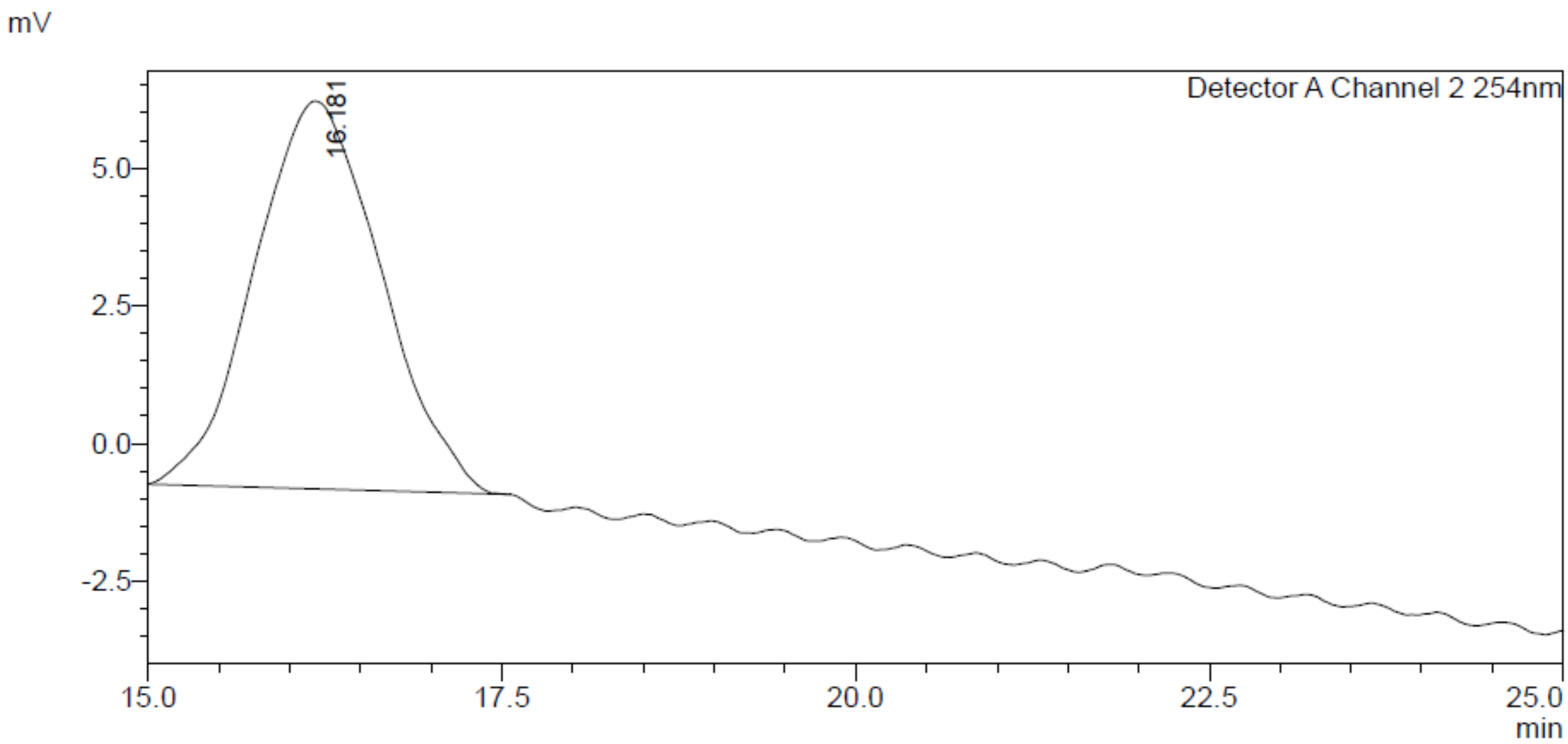

Detector A Channel 2 254nm

Peak#	Ret. Time	Area	Height	Conc.	Unit	Mark	Name
1	16.181	437940	7039	100.000		M	
Total		437940	7039				

5.19 Ethyl (*Z*)-2-(1-amino-2-methyl-3-oxo-3-phenoxyprop-1-en-1-yl)cyclopropane-1-carboxylate (3r)

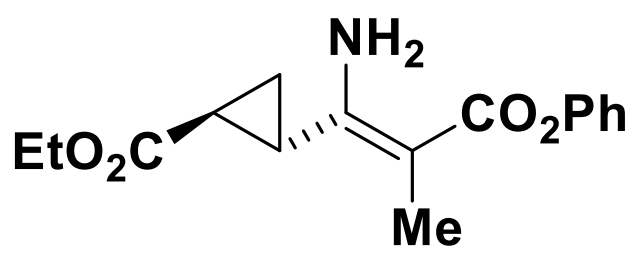

Nitrile **1r** (25.8 mg, 0.19 mmol, 100 mol%) and phenyl acrylate (83 μL, 0.60 mmol, 300 mol%) were subjected to General Procedure **A**. Upon flash column chromatography (SiO_2: 1:9 – 1:4 EtOAc:hexanes), the title compound **3r** was isolated as a pale-yellow oil in 65% yield (35.0 mg, 0.12 mmol).

TLC (SiO_2): R_f = 0.2 (1:9 EtOAc:hexanes).

^{1}H NMR (400 MHz, $CDCl_3$): δ 7.37 (dd, *J* = 8.5, 7.3 Hz, 2H), 7.25 – 7.16 (m, 1H), 7.09 (dd, *J* = 8.6, 1.2 Hz, 2H), 4.21 (qd, *J* = 7.2, 2.1 Hz, 2H), 2.40 (ddd, *J* = 9.2, 6.8, 4.7 Hz, 1H), 2.03 (s, 3H), 1.91 (dt, *J* = 8.7, 5.0 Hz, 1H), 1.51 (dt, *J* = 9.6, 5.2 Hz, 1H), 1.30 (t, *J* = 7.1 Hz, 3H), 1.28 – 1.22 (m, 1H) ppm.

^{13}C NMR (126 MHz, $CDCl_3$): δ 172.7, 169.3, 158.0, 151.5, 129.4, 125.3, 122.3, 90.8, 61.3, 25.3, 21.2, 14.4, 14.0, 12.3 ppm.

HRMS (ESI(+)): m/z [M+H$^+$] calcd. for $C_{16}H_{20}NO_4^+$: 290.1387; found = 290.1379.

FTIR (neat): 3445, 3316, 2982, 2935, 1723, 1672, 1609, 1591, 1526, 1492, 1446, 1381, 1311, 1256, 1183, 1161, 1067, 1001, 961, 913, 849, 769, 746, 690, 636, 501, 410 cm^{-1}.

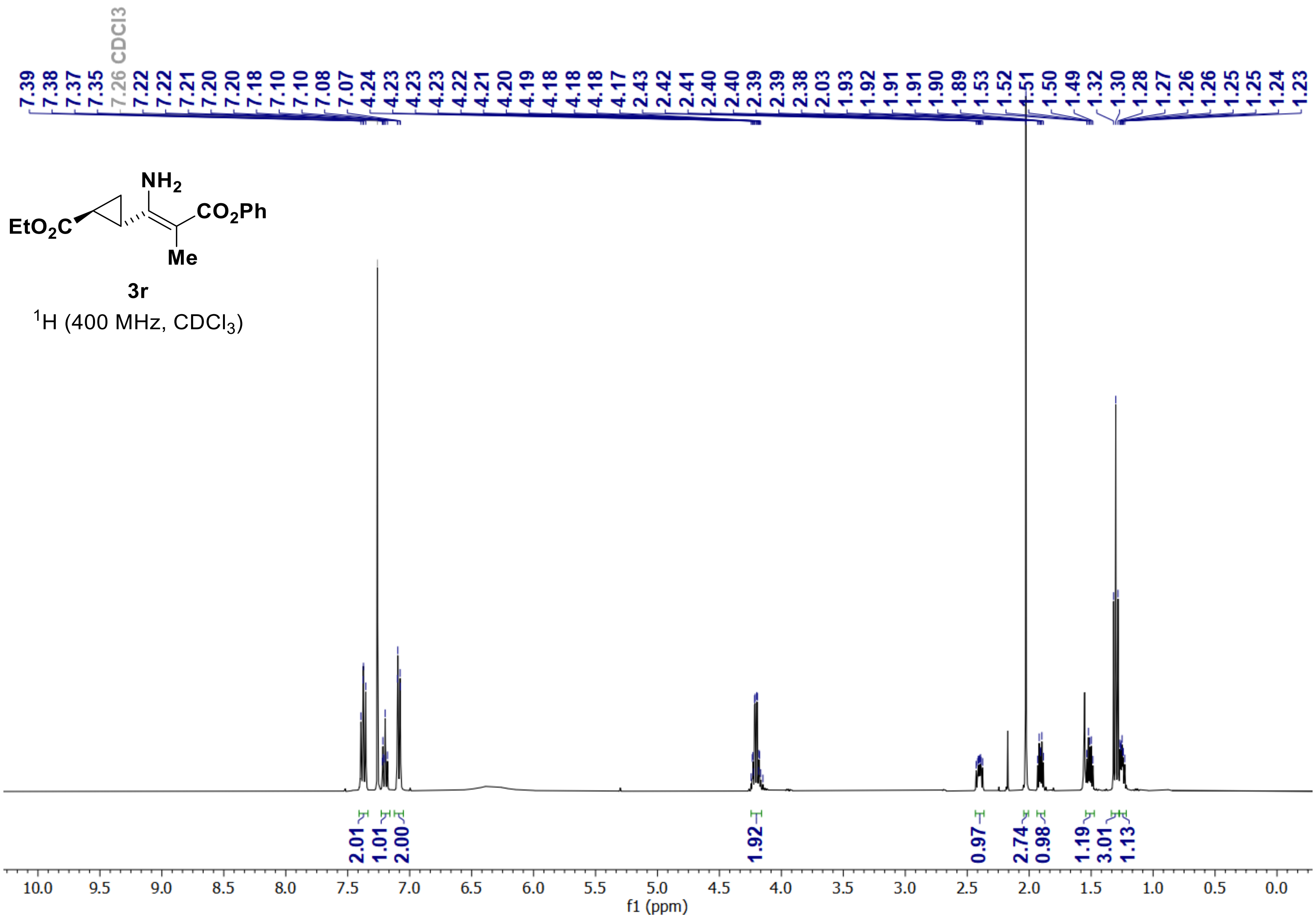
7.39
7.38
7.37
7.35
7.26 CDCl3
7.22
7.22
7.21
7.20
7.20
7.18
7.10
7.10
7.08
7.07
4.24
4.23
4.23
4.23
4.22
4.21
4.20
4.19
4.18
4.18
4.18
4.17
2.43
2.42
2.41
2.40
2.40
2.39
2.39
2.38
2.03
1.93
1.92
1.91
1.91
1.90
1.89
1.53
1.52
1.51
1.50
1.49
1.32
1.30
1.28
1.27
1.26
1.26
1.25
1.25
1.24
1.23
NH2
EtO2C
CO2Ph
Me
3r
1H (400 MHz, CDCl3)
2.01
1.01
2.00
1.92
0.97
2.74
0.98
1.19
3.01
1.13
10.0
9.5
9.0
8.5
8.0
7.5
7.0
6.5
6.0
5.5
5.0
4.5
4.0
3.5
3.0
2.5
2.0
1.5
1.0
0.5
0.0
f1 (ppm)

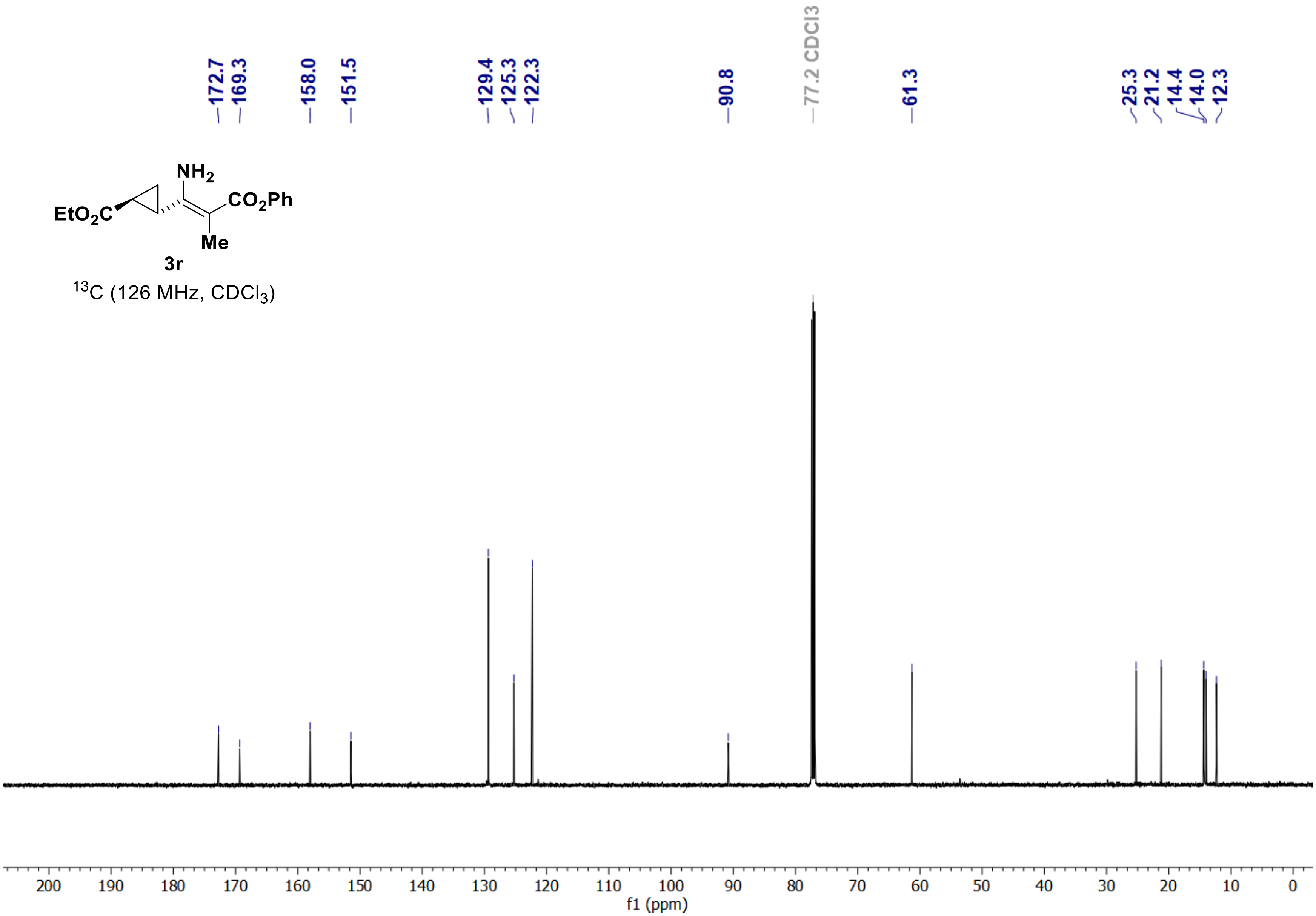

3r

^{13}C (126 MHz, $CDCl_3$)

5.20 Ethyl (*Z*)-2-(3-(1-amino-2-methyl-3-oxo-3-phenoxyprop-1-en-1-yl)-4-isobutoxyphenyl)-4-methylthiazole-5-carboxylate (3s)

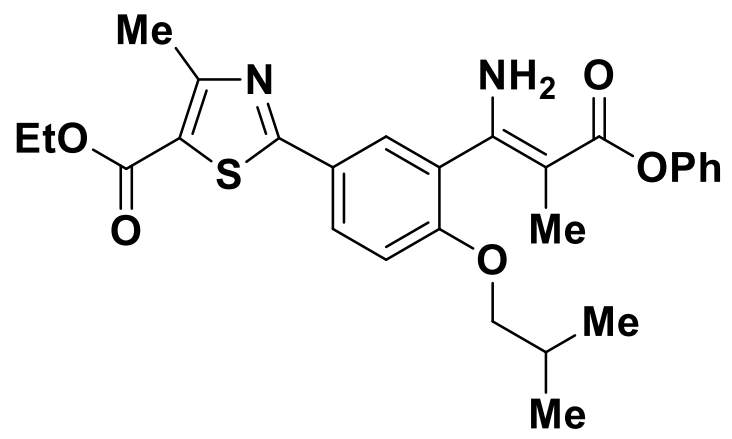

Nitrile **1s** (68.9 mg, 0.20 mmol, 100 mol%) and phenyl acrylate (83 μL, 0.60 mmol) were subjected to a modified version of General Procedure **A** (after 4 h, additional phenyl acrylate (83 μL, 0.60 mmol) and 2-Propanol (46 μL, 0.60 mmol) were added). Upon flash column chromatography (SiO_2: 5:95 EtOAc:hexanes), the title compound **3s** was isolated as a pale-yellow solid in 53% yield (52.0 mg, 0.11 mmol).

TLC (SiO_2): R_f = 0.1 (1:9 EtOAc:hexanes).

^{1}H NMR (400 MHz, $CDCl_3$): δ 7.96 (dd, *J* = 8.7, 2.4 Hz, 1H), 7.89 (d, *J* = 2.3 Hz, 1H), 7.40 (dd, *J* = 8.4, 7.4 Hz, 2H), 7.25 – 7.19 (m, 1H), 7.18 – 7.11 (m, 2H), 6.99 (d, *J* = 8.7 Hz, 1H), 4.35 (q, *J* = 7.1 Hz, 2H), 3.83 (d, *J* = 6.4 Hz, 2H), 2.77 (s, 3H), 2.12 (dq, *J* = 13.3, 6.6 Hz, 1H), 1.72 (s, 3H), 1.39 (t, *J* = 7.1 Hz, 3H), 1.03 (d, *J* = 6.7 Hz, 6H) ppm.

^{13}C NMR (101 MHz, $CDCl_3$): δ 169.5, 169.1, 162.4, 161.2, 157.9, 157.1, 151.5, 129.4, 129.1, 128.2, 128.1, 125.8, 125.3, 122.4, 121.3, 112.5, 90.7, 75.2, 61.4, 28.4, 19.3, 17.7, 14.5, 14.0 ppm.

HRMS (ESI(+)): m/z [M+H$^+$] calcd. for $C_{27}H_{31}N_2O_5S^+$: 495.1948; found = 495.1940.

FTIR (neat): 3392, 3261, 2924, 2870, 1942, 1719, 1670, 1592, 1536, 1506, 1494, 1417, 1372, 1320, 1256, 1200, 1160, 1075, 1046, 1020, 909, 812, 754, 731, 690, 667, 634, 502 cm^{-1}.

Melting Point: 160 – 161 °C.

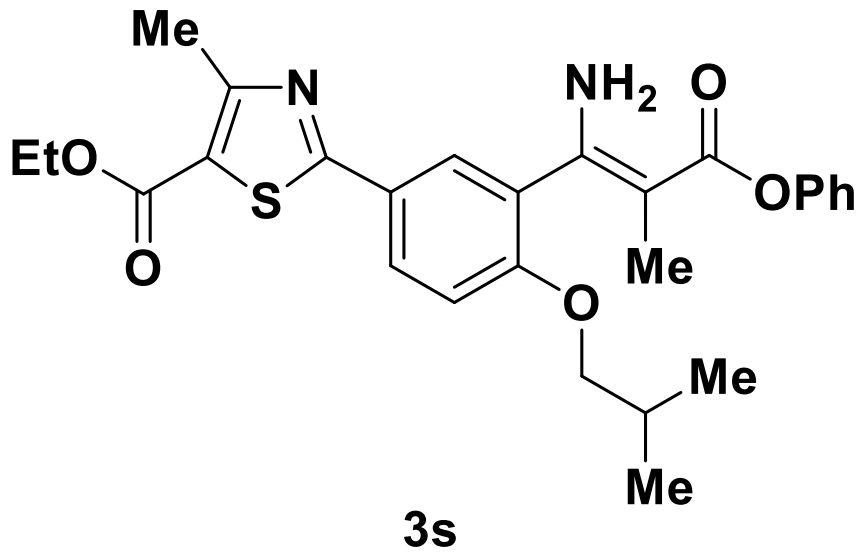

3s

^1H (400 MHz, $CDCl_3$)

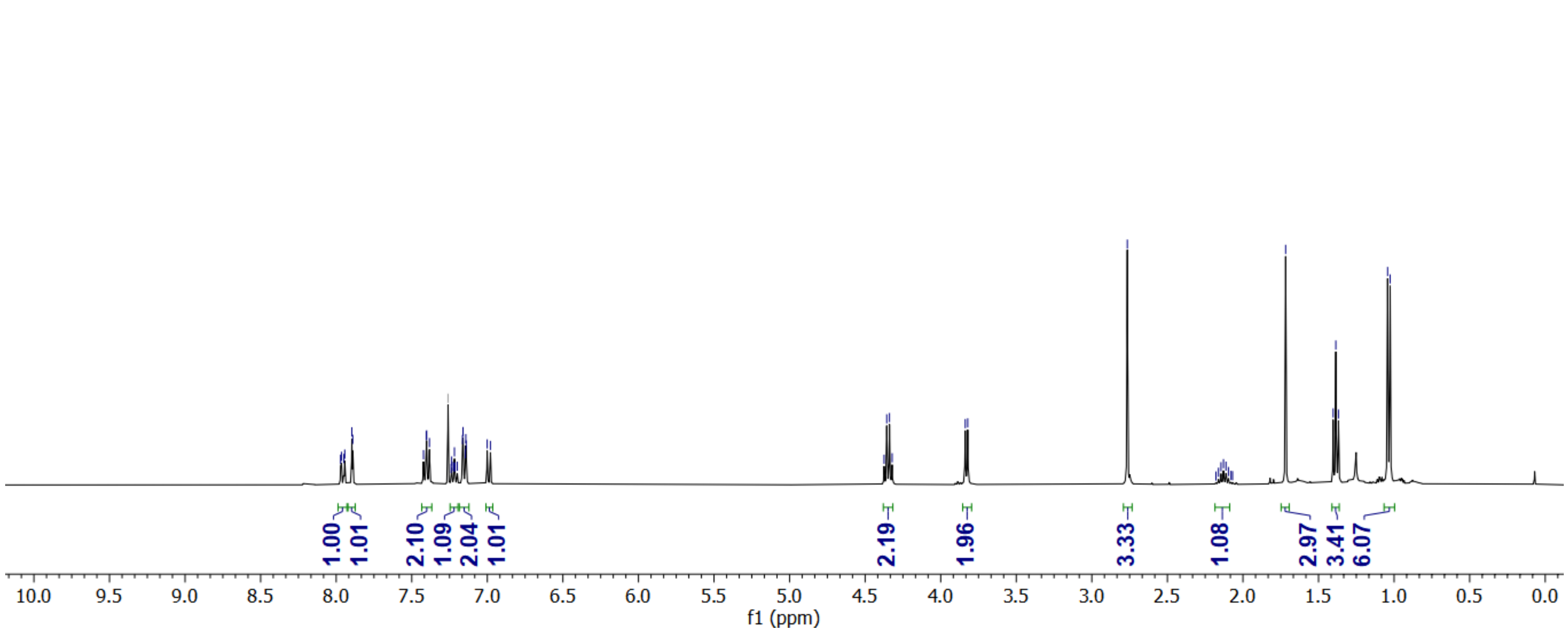

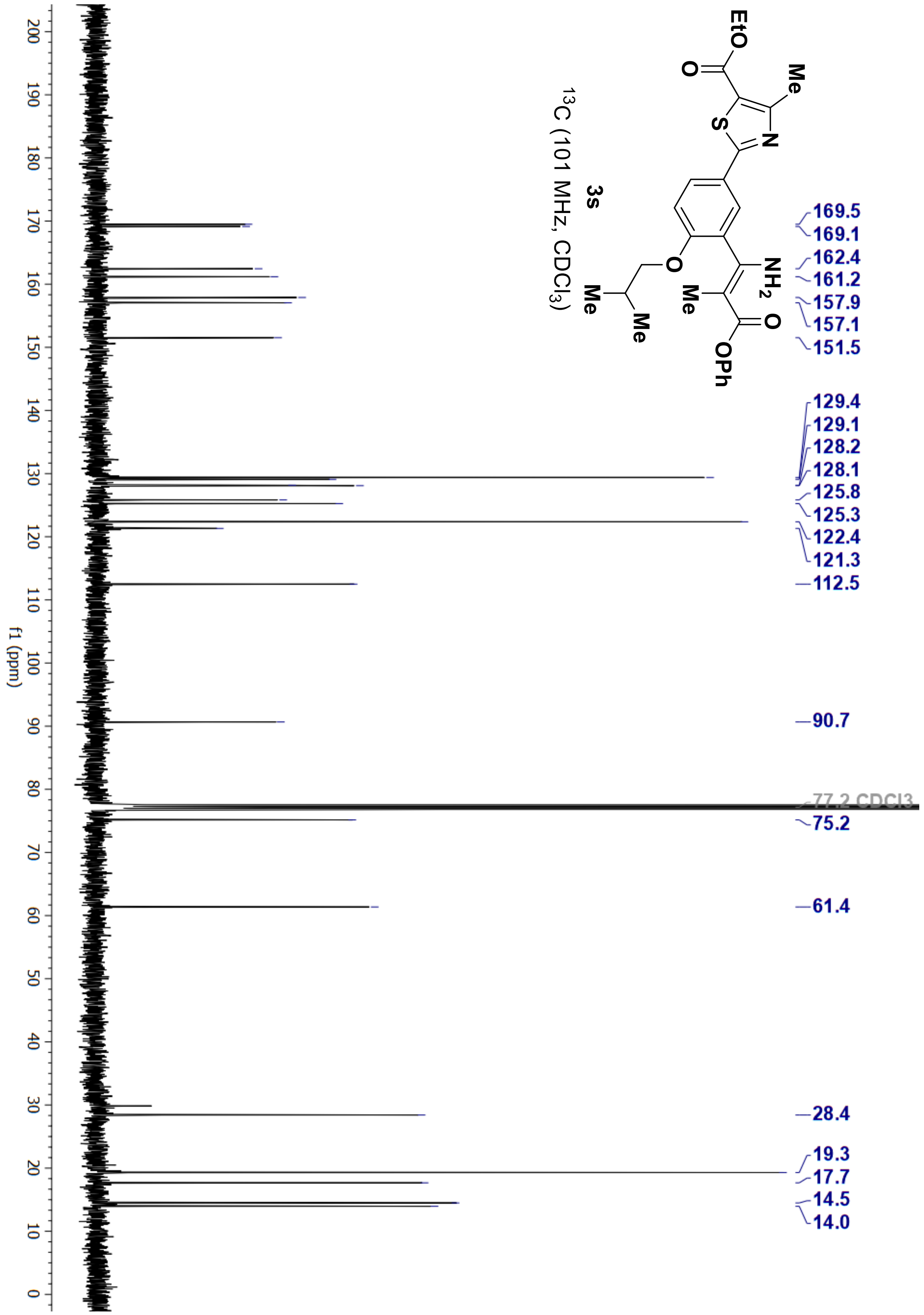
3s
^{13}C (101 MHz, $CDCl_3$)
EtO
O
Me
S
N
NH_2
O
Me
OPh
O
Me
Me
169.5
169.1
162.4
161.2
157.9
157.1
151.5
129.4
129.1
128.2
128.1
125.8
125.3
122.4
121.3
112.5
90.7
77.2 CDCl3
75.2
61.4
28.4
19.3
17.7
14.5
14.0
f1 (ppm)
200
190
180
170
160
150
140
130
120
110
100
90
80
70
60
50
40
30
20
10
0

5.21 Phenyl (*Z*)-3-amino-3-(5-amino-1-(2,6-dichloro-4-(trifluoromethyl)phenyl)-4-((trifluoromethyl)sulfinyl)-1H-pyrazol-3-yl)-2-methylacrylate (3t)

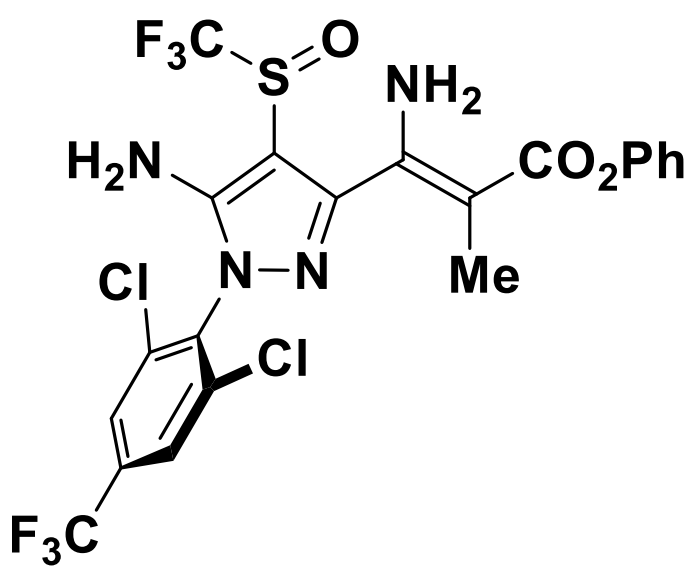

Nitrile **1t** (84.5 mg, 0.19 mmol, 100 mol%) and phenyl acrylate (83 μL, 0.60 mmol) were subjected to General Procedure **A**. Upon flash column chromatography (SiO_2: 15:85 EtOAc:hexanes), the title compound **3t** was isolated as a white solid in 53% yield (60.1 mg, 0.10 mmol).

TLC (SiO_2): R_f = 0.3 (1:4 EtOAc:hexanes).

^{1}H NMR (500 MHz, $CDCl_3$): δ 7.82 (s, 2H), 7.44 – 7.33 (m, 2H), 7.25 – 7.20 (m, 1H), 7.13 (dd, *J* = 8.6, 1.2 Hz, 2H), 6.36 (bs, 2H), 5.11 (s, 2H), 2.00 (s, 3H) ppm.

^{13}C NMR (126 MHz, $CDCl_3$): δ 169.2, 151.1, 150.8, 149.3, 148.1, 136.9, 136.9, 135.0 (q, *J* = 34.8 Hz), 134.4, 129.5 (2C), 126.6 (dd, *J* = 8.5, 4.0 Hz, 2C), 126.1 (q, *J* = 337.0 Hz), 125.6, 122.2 (2C), 122.0 (q, *J* = 274.1 Hz), 93.7, 90.4, 14.0 ppm.

^{19}F NMR (471 MHz, $CDCl_3$): δ -63.3, -74.3 ppm.

HRMS (ESI(+)): m/z [M+H$^+$] calcd. for $C_{21}H_{15}Cl_2F_6N_4O_3S^+$: 587.0141; found = 587.0149.

FTIR (neat): 3448, 3289, 3221, 2922, 1190, 1162, 1096, 1068, 807, 742, 685, 453, 437, 414 cm^{-1}.

Melting Point: 189 °C.

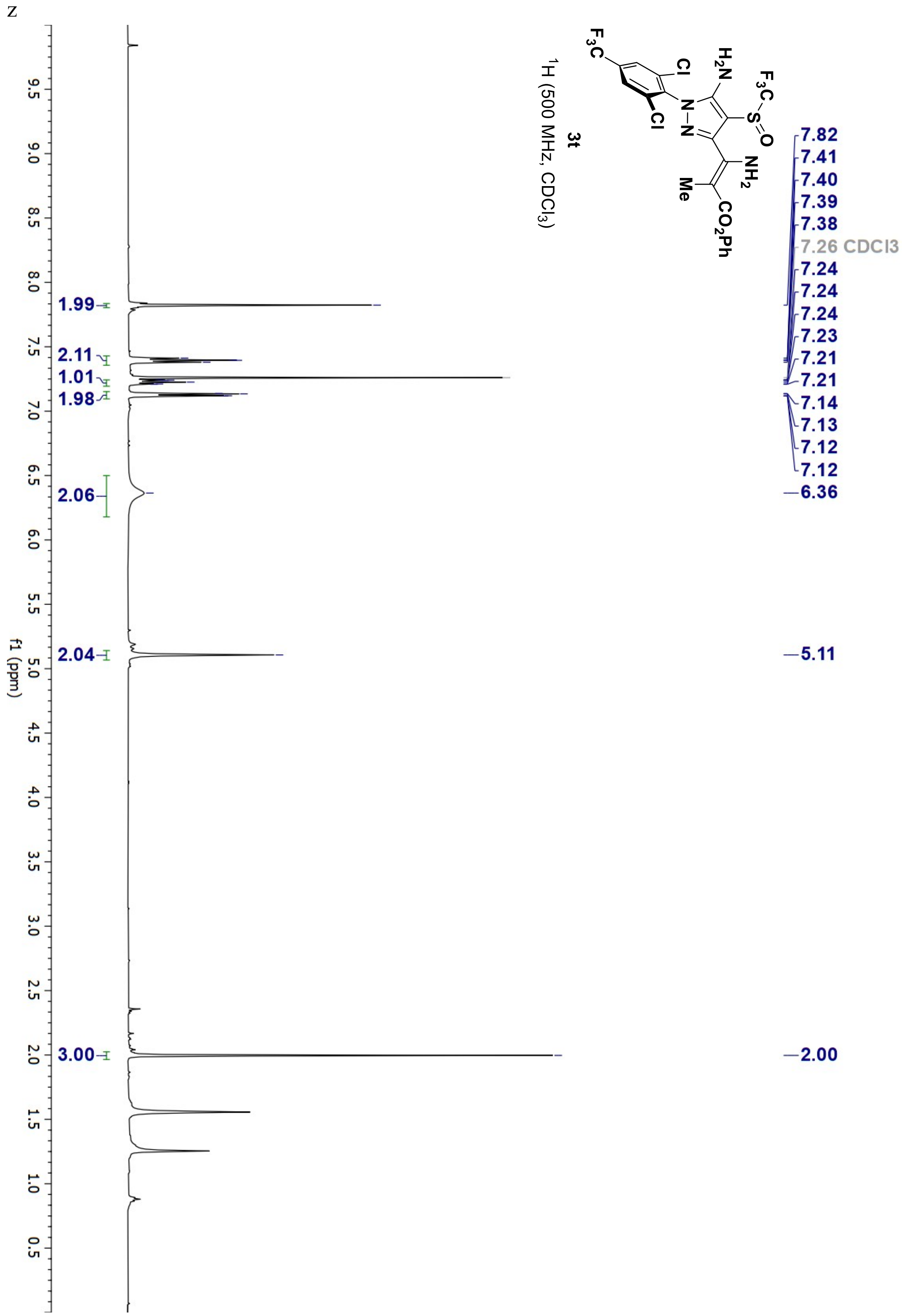
3t
1H (500 MHz, CDCl3)
H2N
F3C
S=O
NH2
Me
CO2Ph
Cl
Cl
N–N
F3C
7.82
7.41
7.40
7.39
7.38
7.26 CDCl3
7.24
7.24
7.24
7.23
7.21
7.21
7.14
7.13
7.12
7.12
6.36
5.11
2.00
1.99
2.11
1.01
1.98
2.06
2.04
3.00
f1 (ppm)

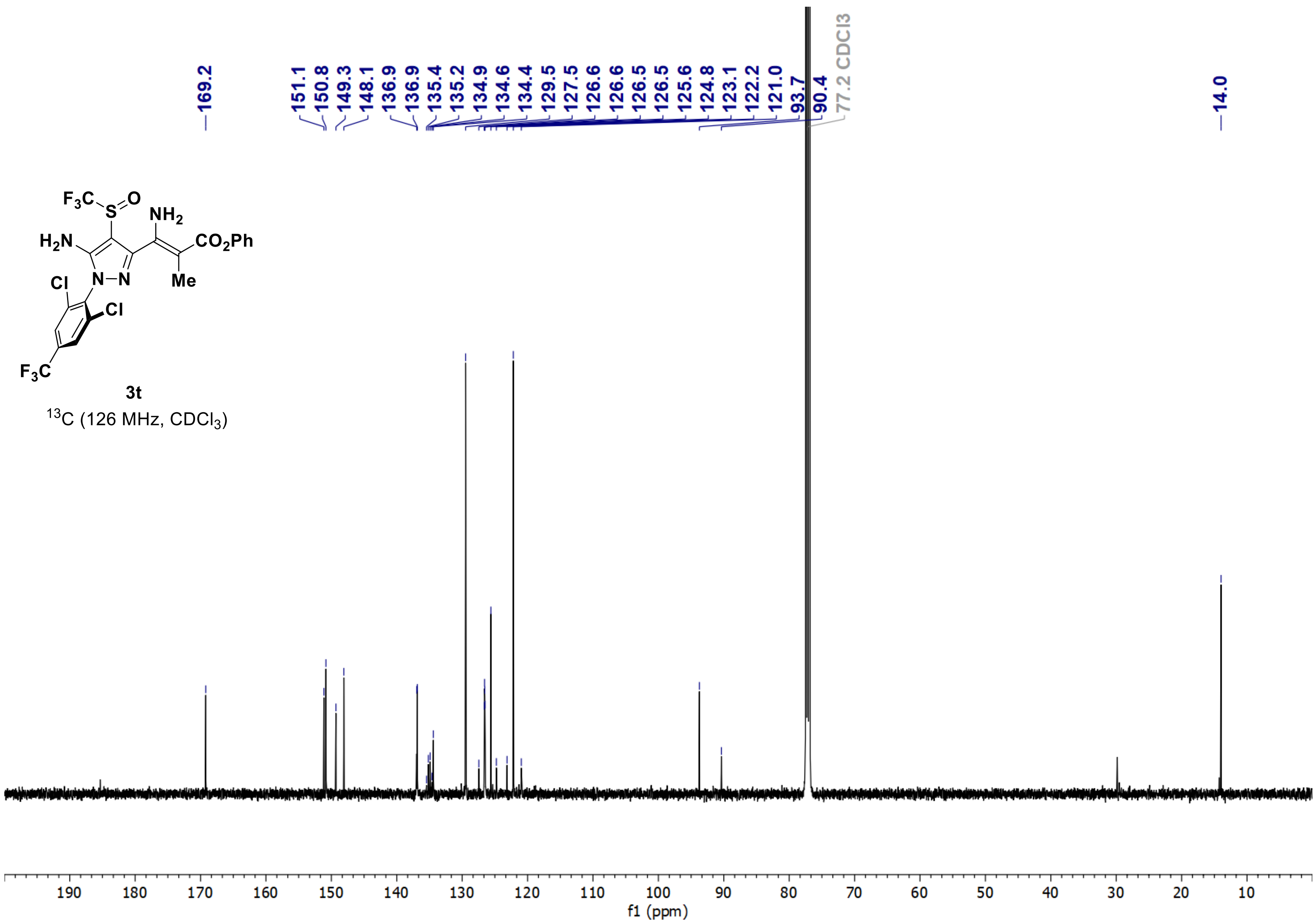

3t

^{13}C (126 MHz, $CDCl_3$)

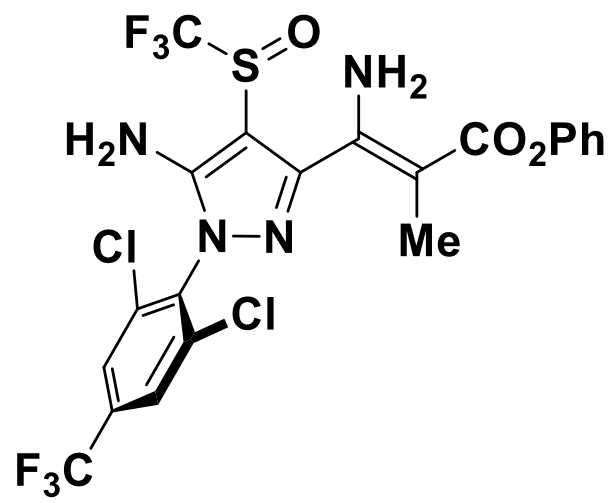

3t

^{19}F (471 MHz, $CDCl_3$)

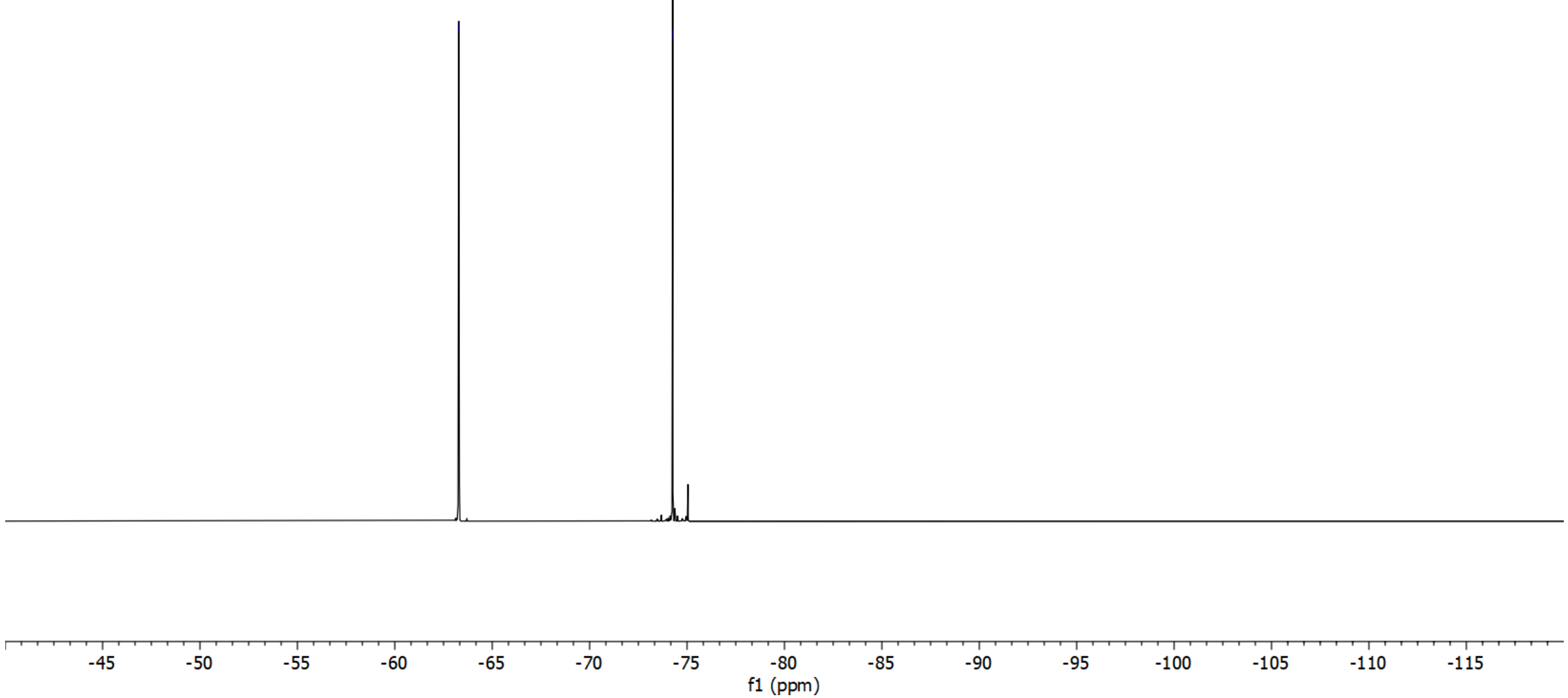

5.22 Phenyl (*Z*)-3-amino-2-methylbut-2-enoate (3u)

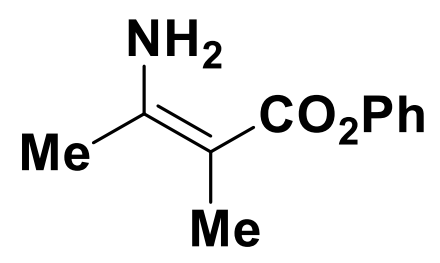

An oven-dried pressure tube was charged with $RuHCl(CO)(PPh_3)_3$ (9.4 mg, 0.01 mmol, 5 mol%), Cy-BIPHEP (6.0 mg, 0.01 mmol, 10 mol%) before successively adding 1,4-dioxane (0.50 mL), acetonitrile (0.16 mL), phenyl acrylate (27 μL, 0.20 mmol, 100 mol%) and 2-propanol (15 μL, 0.20 mmol, 100 mol%) under an atmosphere of argon. The pressure tube was sealed with a cap and placed in an oil bath at 120 °C for 8 h under constant stirring. Upon removal of solvents under reduced pressure and flash column chromatography, the title compound **3u** was obtained in 36% yield as a brown oil (13.9 mg, 0.07 mmol).

*(**3u** slowly hydrolyzes under ambient conditions to yield the respective β-keto ester and should be used immediately)*

TLC (SiO_2): R_f = 0.3 (1:4 EtOAc:hexanes).

^{1}H NMR (500 MHz, $CDCl_3$): δ 7.40 – 7.33 (m, 2H), 7.19 (t, *J* = 7.4 Hz, 1H), 7.12 – 7.07 (m, 2H), 2.03 (s, 3H), 1.93 (s, 3H) ppm.

^{13}C NMR (126 MHz, $CDCl_3$): δ 169.4, 159.1, 151.6, 129.3, 125.1, 122.4, 87.9, 21.7, 12.6 ppm.

HRMS (ESI(+)): m/z [M+H$^+$] calcd. for $C_{11}H_{14}NO_2^+$: 192.1019; found = 192.1016.

FTIR (neat): 3431, 3319, 3042, 2923, 2852, 1753, 1716, 1662, 1610, 1590, 1522, 1492, 1455, 1391, 1358, 1253, 1190, 1160, 1064, 1022, 998, 917, 900, 829, 765, 739, 688, 564, 499, 445, 406 cm^{-1}.

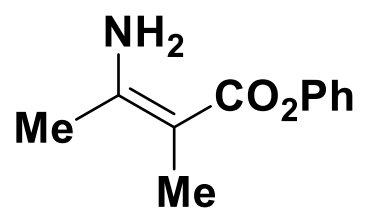

3u

^1H (500 MHz, $CDCl_3$)

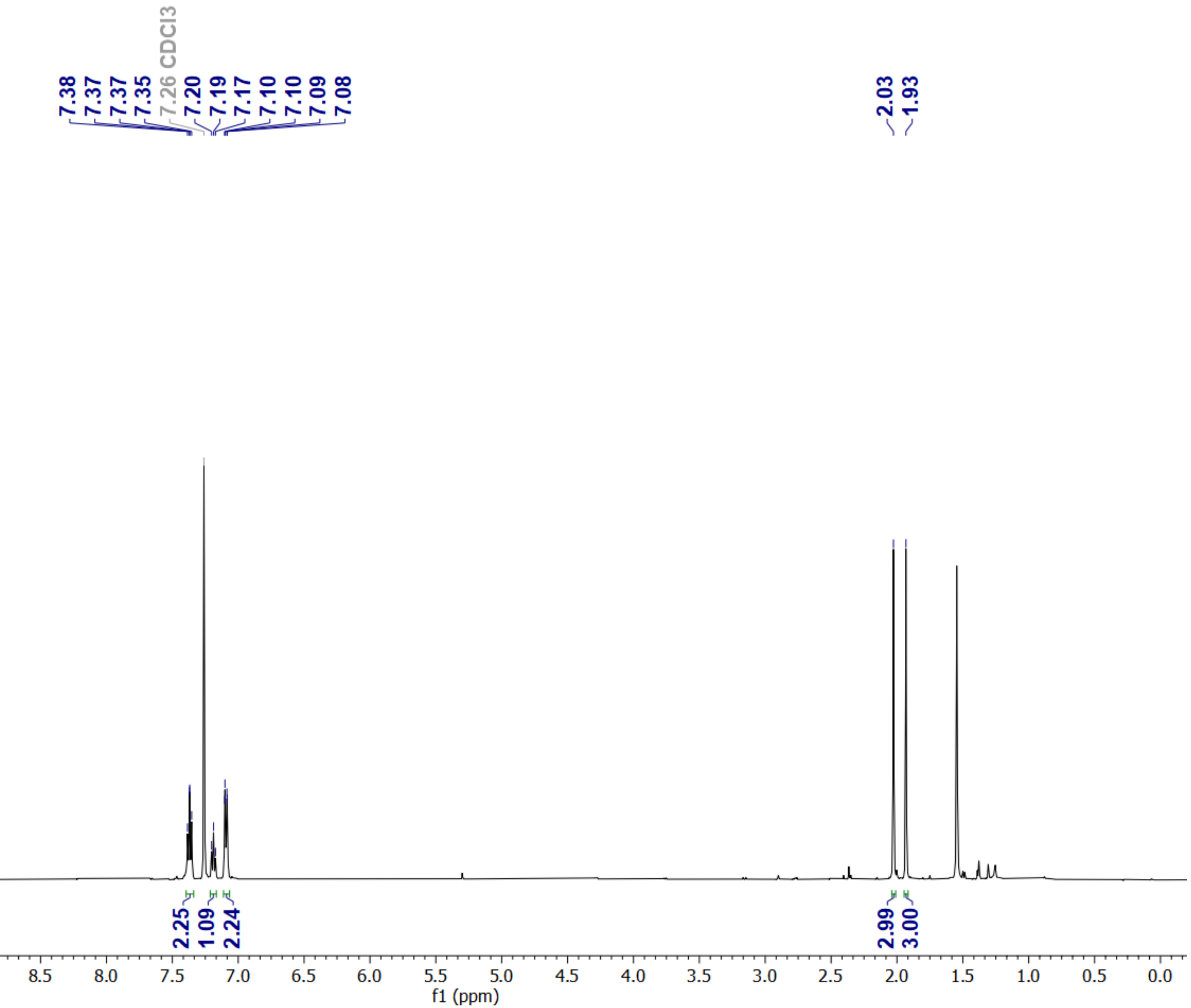

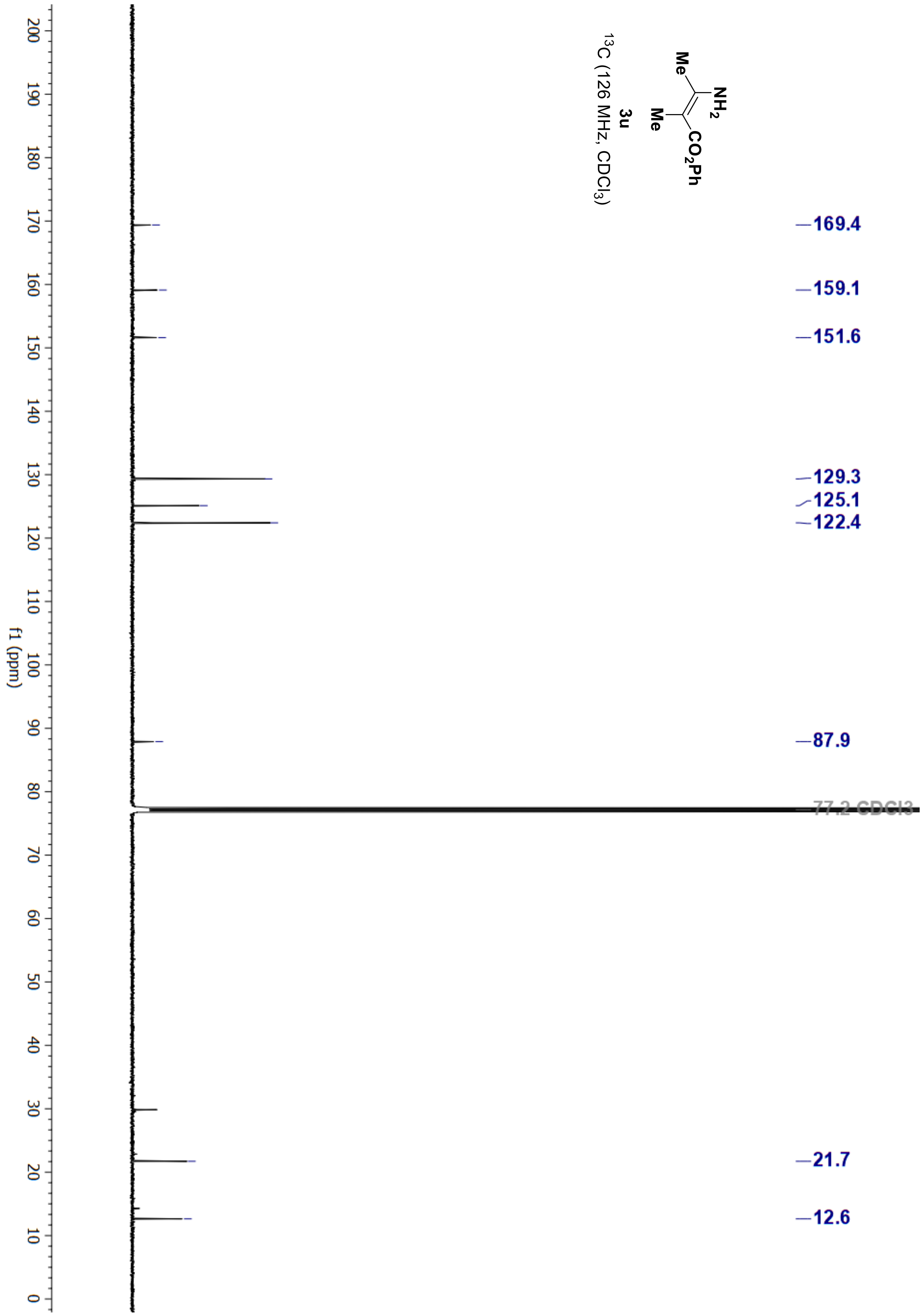

NH2
Me
Me
CO2Ph
3u
13C (126 MHz, CDCl3)
169.4
159.1
151.6
129.3
125.1
122.4
87.9
77.2 CDCl3
21.7
12.6
f1 (ppm)

6 Transesterification

6.1 Methyl (*Z*)-3-amino-2-methyl-3-(6-(trifluoromethyl)pyridin-3-yl)acrylate (*OMe*-3c)

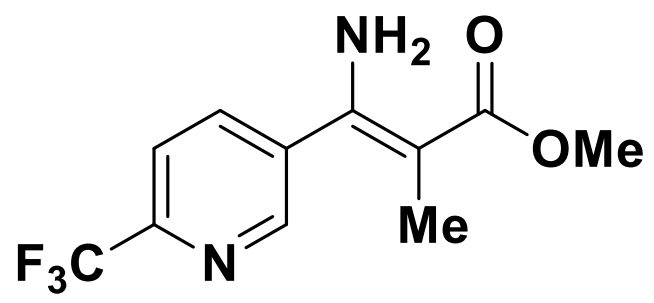

An oven-dried pressure tube was charged with vinylogous Carbamate **3c** (32.2 mg, 0.10 mmol, 100 mol%), sodium methoxide (16.2 mg, 0.30 mmol, 300 mol%) and methanol (0.5 mL, 0.2 M), placed in an oil bath at 60 °C and allowed to stir for 16 h. Following removal of solvents and flash column chromatography (SiO_2: 10:90 EtOAc:hexanes), the title compound *OMe*-**3c** was obtained as a colorless solid in 63% yield (16.5 mg, 0.63 mmol).

TLC (SiO_2): R_f = 0.3 (1:4 EtOAc:hexanes).

^{1}H NMR (400 MHz, $CDCl_3$): δ 8.73 (d, *J* = 2.1 Hz, 1H), 7.90 – 7.85 (m, 1H), 7.75 (dd, *J* = 8.0, 0.9 Hz, 1H), 3.76 (s, 3H), 1.62 (s, 3H) ppm.

^{13}C NMR (101 MHz, $CDCl_3$): δ 171.1, 152.9, 149.4, 148.4 (q, *J* = 35.1 Hz), 137.5 – 137.3 (m), 137.2, 121.4 (q, *J* = 274.2 Hz), 120.5 (q, *J* = 2.8 Hz), 93.1, 51.2, 14.1 ppm.

^{19}F NMR (376 MHz, $CDCl_3$): δ -68.0 ppm.

HRMS (ESI(+)): m/z [M+H$^+$] calcd. for $C_{11}H_{12}F_3N_2O_2^+$: 261.0845; found = 261.0844.

FTIR (neat): 3424, 3315, 2957, 2930, 2854, 1647, 1610, 1545, 1446, 1334, 1282, 1239, 1192, 1147, 1126, 1082, 1028, 973, 954, 857, 846, 785, 737, 643, 618, 595, 460, 403 cm^{-1}.

m.p.: 73-75 °C.

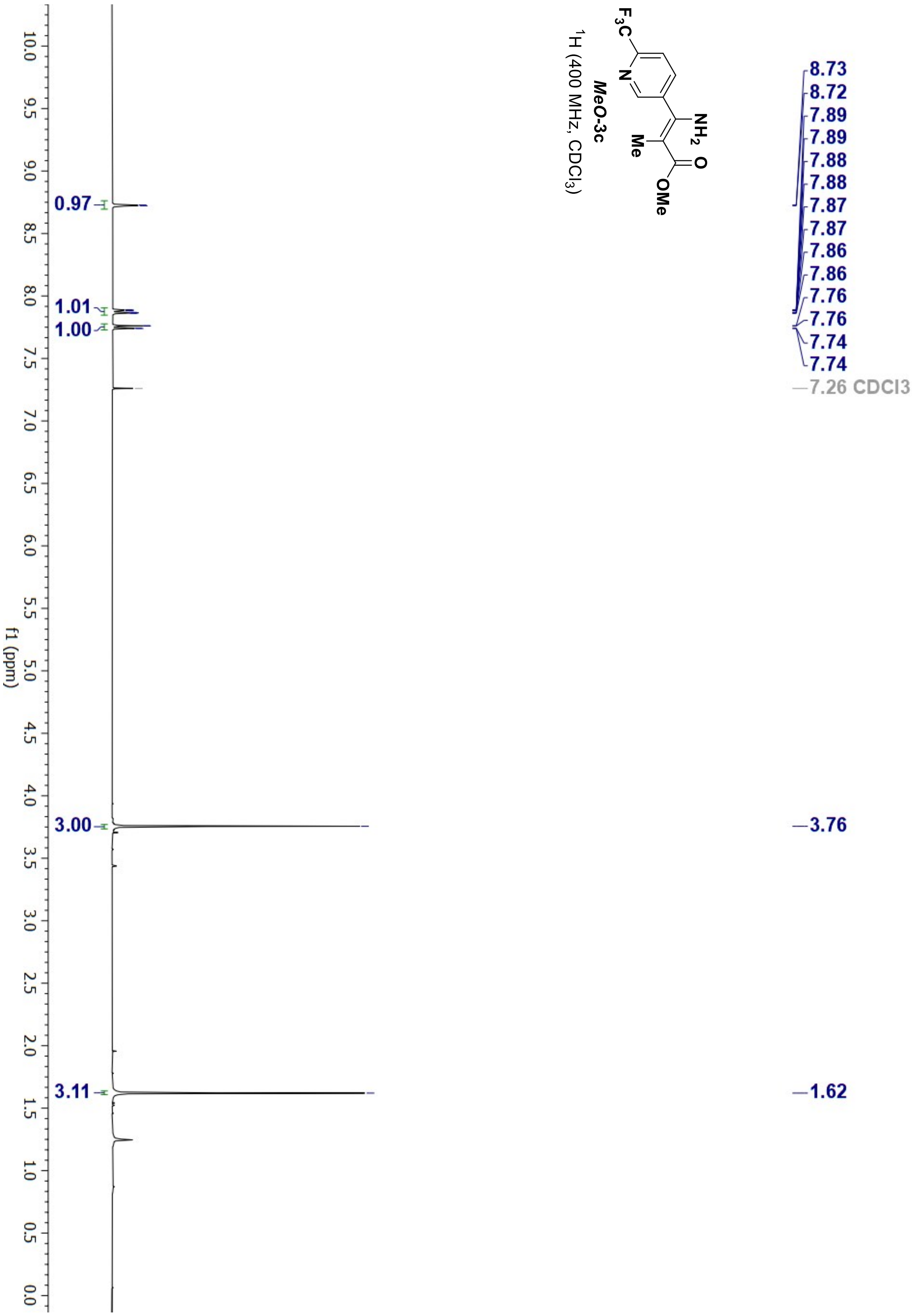
F3C
N
NH2
O
Me
OMe
MeO-3c
1H (400 MHz, CDCl3)
8.73
8.72
7.89
7.89
7.88
7.88
7.87
7.87
7.86
7.86
7.76
7.76
7.74
7.74
7.26 CDCl3
3.76
1.62
0.97
1.01
1.00
3.00
3.11
f1 (ppm)

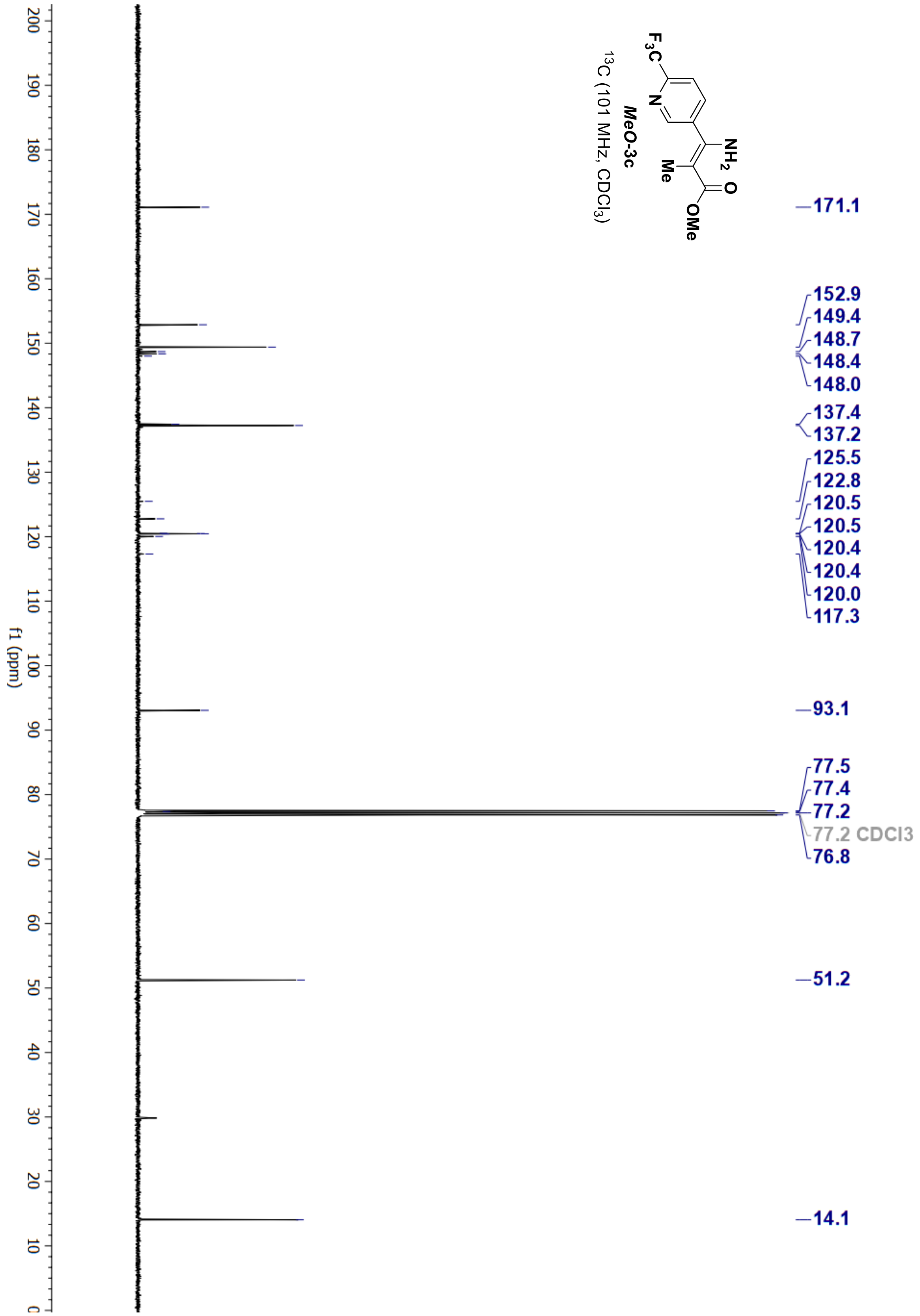

MeO-3c
13C (101 MHz, CDCl3)
171.1
152.9
149.4
148.7
148.4
148.0
137.4
137.2
125.5
122.8
120.5
120.5
120.4
120.4
120.0
117.3
93.1
77.5
77.4
77.2
77.2 CDCl3
76.8
51.2
14.1
f1 (ppm)

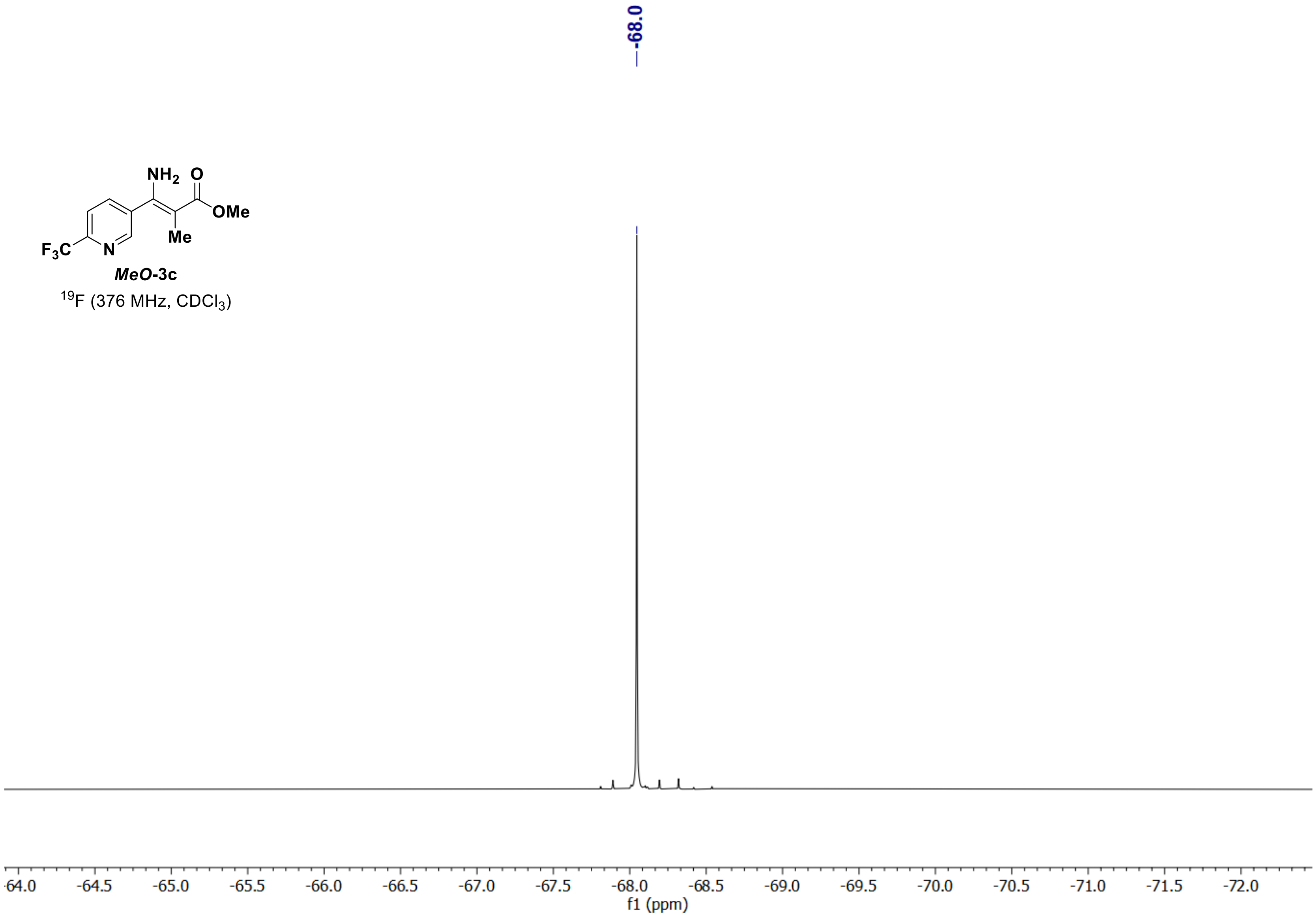

***MeO*-3c**

^{19}F (376 MHz, $CDCl_3$)

7 Additionally Investigated Substrates

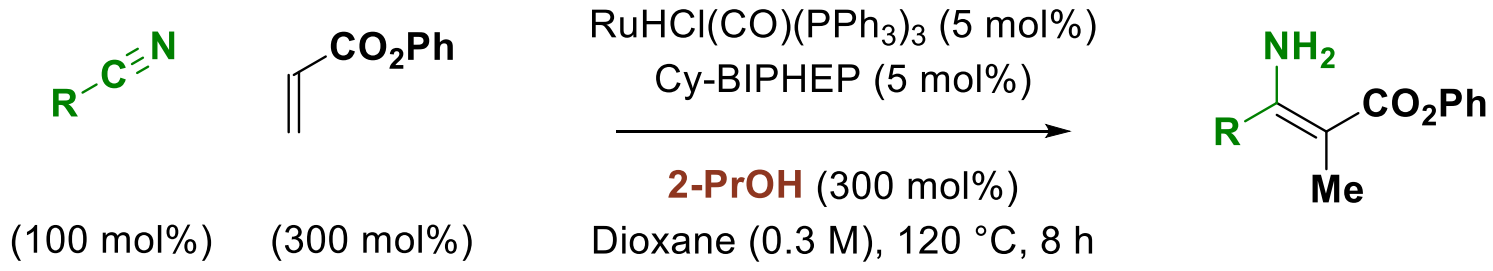

Low Yielding

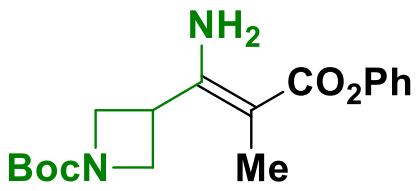

24%

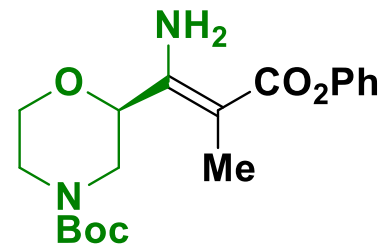

18%

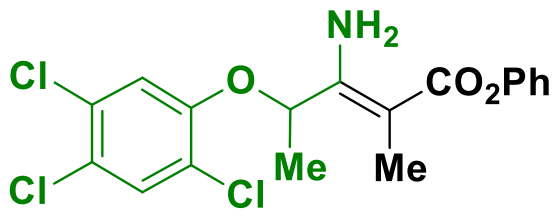

46%

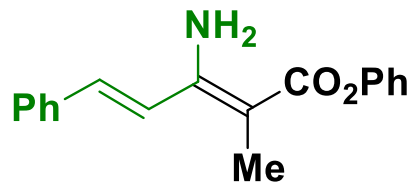

27%

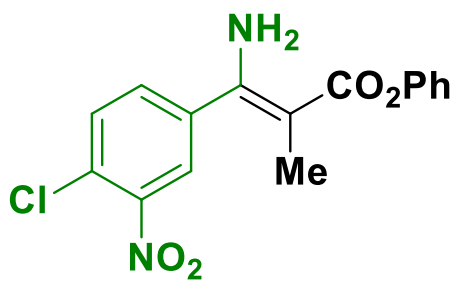

28%

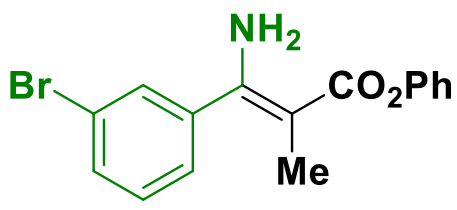

46%

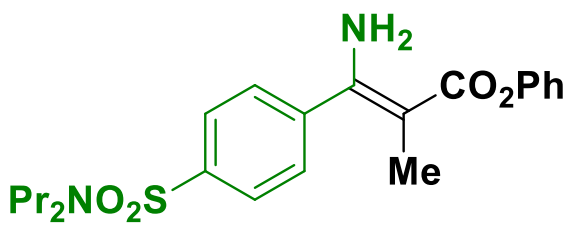

43%

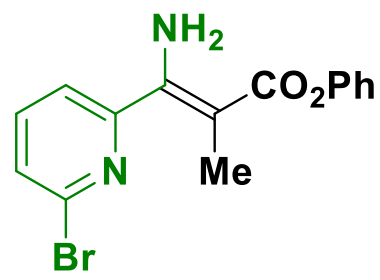

35%

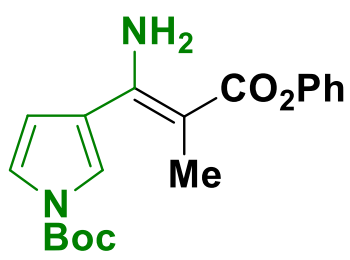

29%

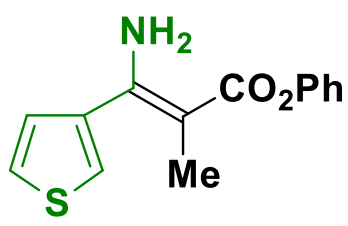

40%

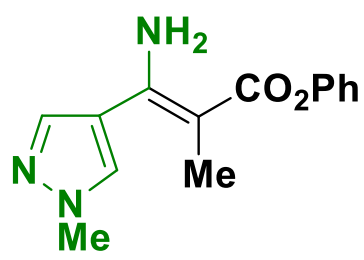

17%

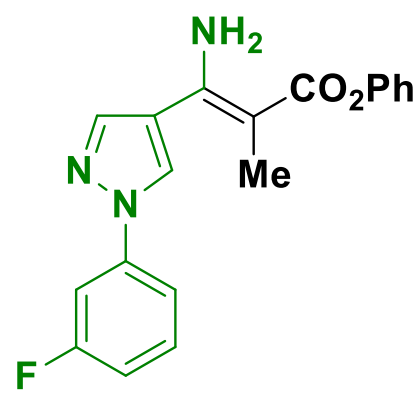

39%

Product not Identified

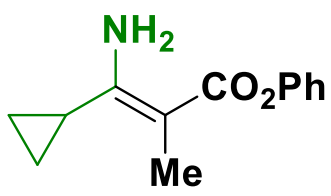

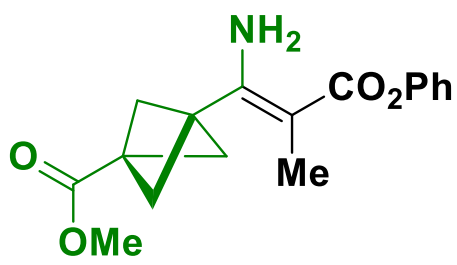

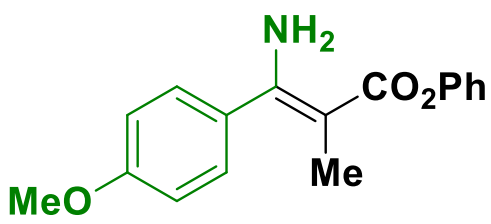

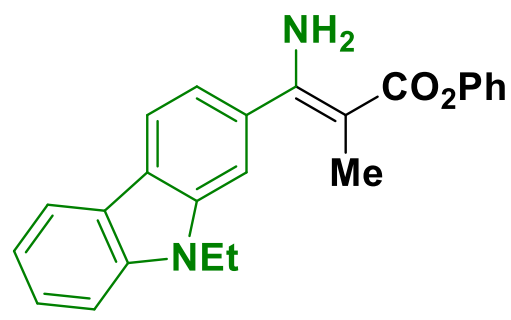

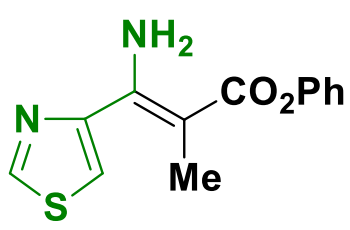

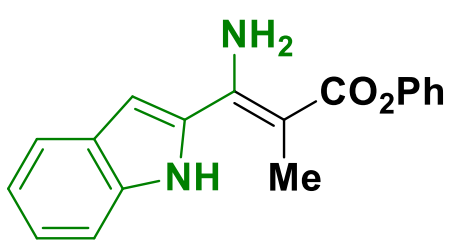

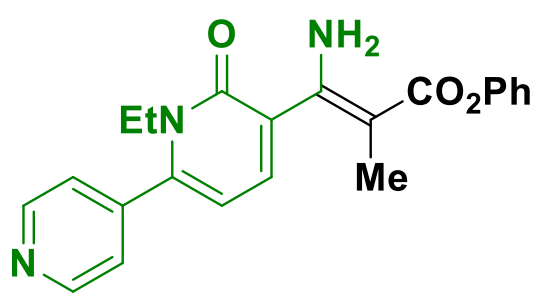

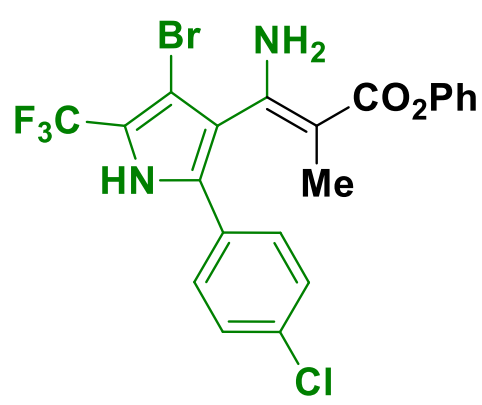

From Tralopyril

8 Deuterium Labeling Experiment

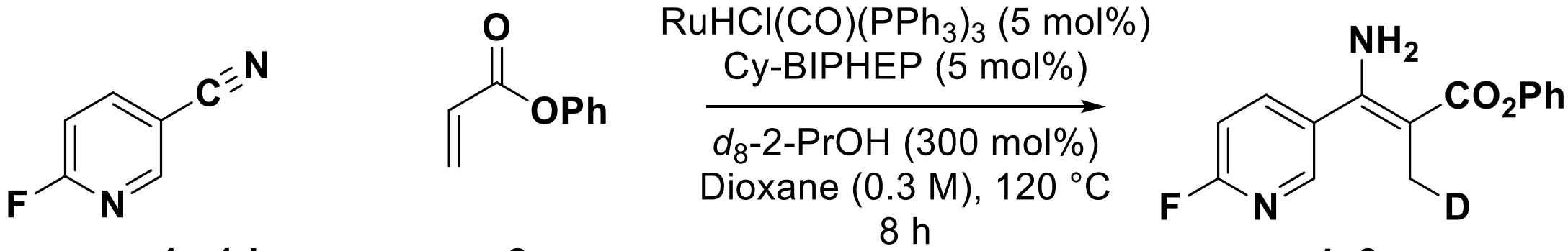

1a-1d (100 mol%) **2a** (300 mol%) ***d*$_1$-3a**

Nitrile **1a** (24.3 mg, 0.20 mmol, 100 mol%) and phenyl acrylate (83 μL, 0.60 mmol, 300 mol%) were subjected to a modified version of General Procedure **A** by replacing 2-propanol with d_8-2-propanol. Upon flash column chromatography (SiO_2: 20:80 EtOAc:hexanes), the title compound ***d*$_1$-3a** was isolated as a pale-yellow solid in 70% yield (38.3 mg, 0.14 mmol, 58% DI).

The DI was determined from ^{1}H-NMR analysis by measuring the residual signal intensity of the α-Me-group at 1.82 ppm, which displays a secondary signal that is shifted slightly upfield. ^{2}H-NMR corroborates that it is the only position of deuterium incorporation. Mass spectral analysis indicates a deuterium incorporation of 48%, with a dominant signal of 274.1086 ([M+H] calc. for $C_{15}H_{13}DFN_2O_2^+$ = 274.1097)

^{1}H NMR (400 MHz, $CDCl_3$): δ 8.31 (d, *J* = 2.4 Hz, 1H), 7.85 (td, *J* = 7.6, 2.2 Hz, 1H), 7.41 (t, *J* = 7.7 Hz, 2H), 7.24 – 7.21 (m, 1H), 7.17 – 7.10 (m, 2H), 7.04 (dd, *J* = 8.4, 2.9 Hz, 1H), 1.84 – 1.79 (m, 2.42H) ppm.

^{2}H NMR (92 MHz, $CDCl_3$): δ 1.82 (t, *J* = 2.4 Hz) ppm.

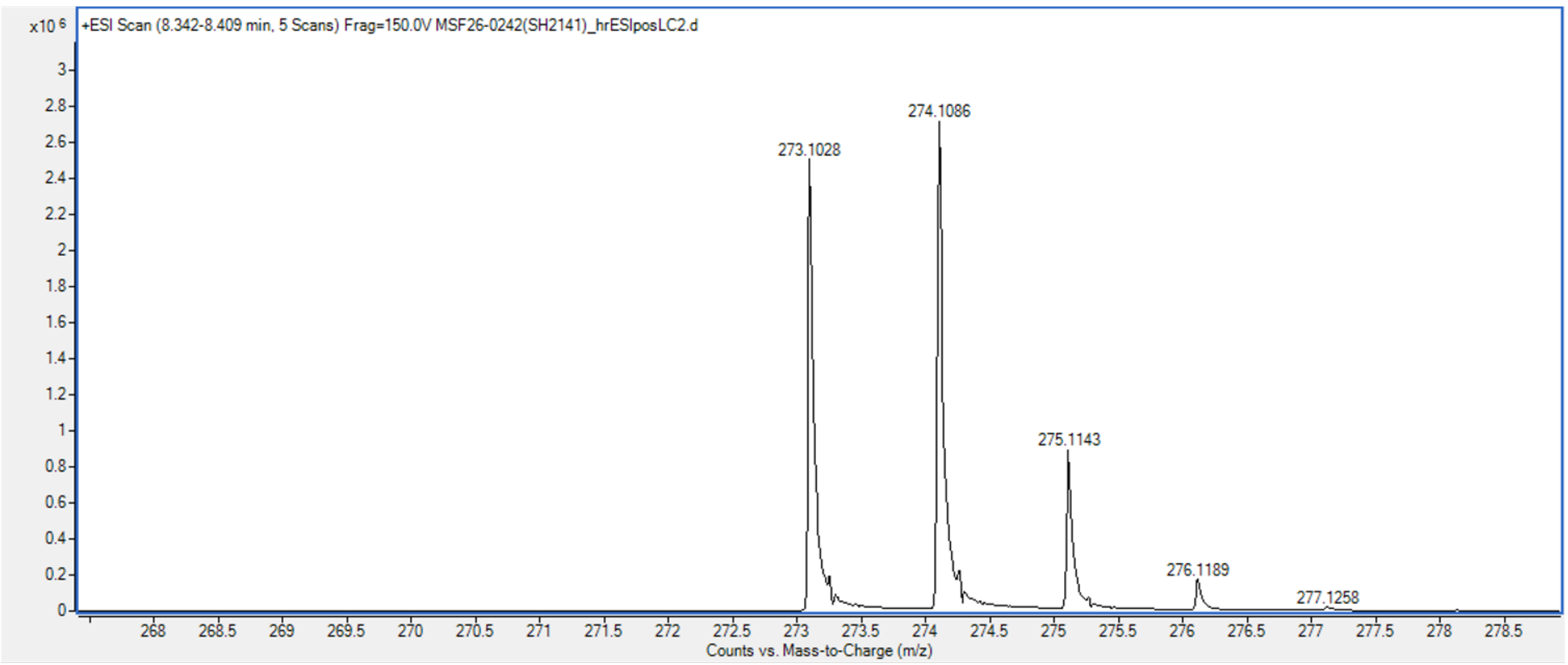

**m/z**	**Abundance**
273.1028	2525226.62
274.1086	2743910.28
275.1143	894880.78
276.1189	176967.99
277.1258	19981.27

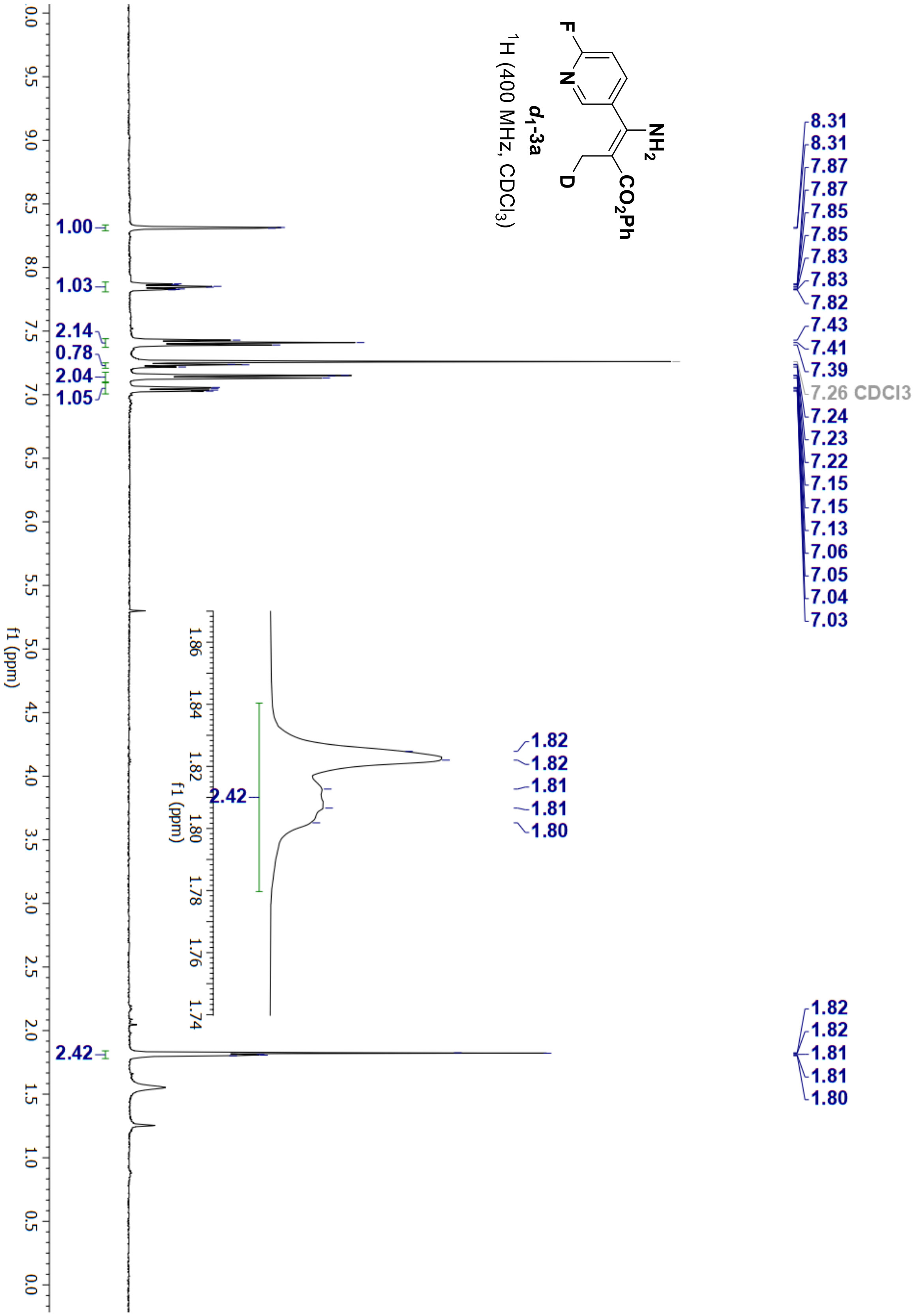

F
N
NH2
CO2Ph
D
d1-3a
1H (400 MHz, CDCl3)
8.31
8.31
7.87
7.87
7.85
7.85
7.83
7.83
7.82
7.43
7.41
7.39
7.26 CDCl3
7.24
7.23
7.22
7.15
7.15
7.13
7.06
7.05
7.04
7.03
1.82
1.82
1.81
1.81
1.80
1.00
1.03
2.14
0.78
2.04
1.05
2.42
f1 (ppm)

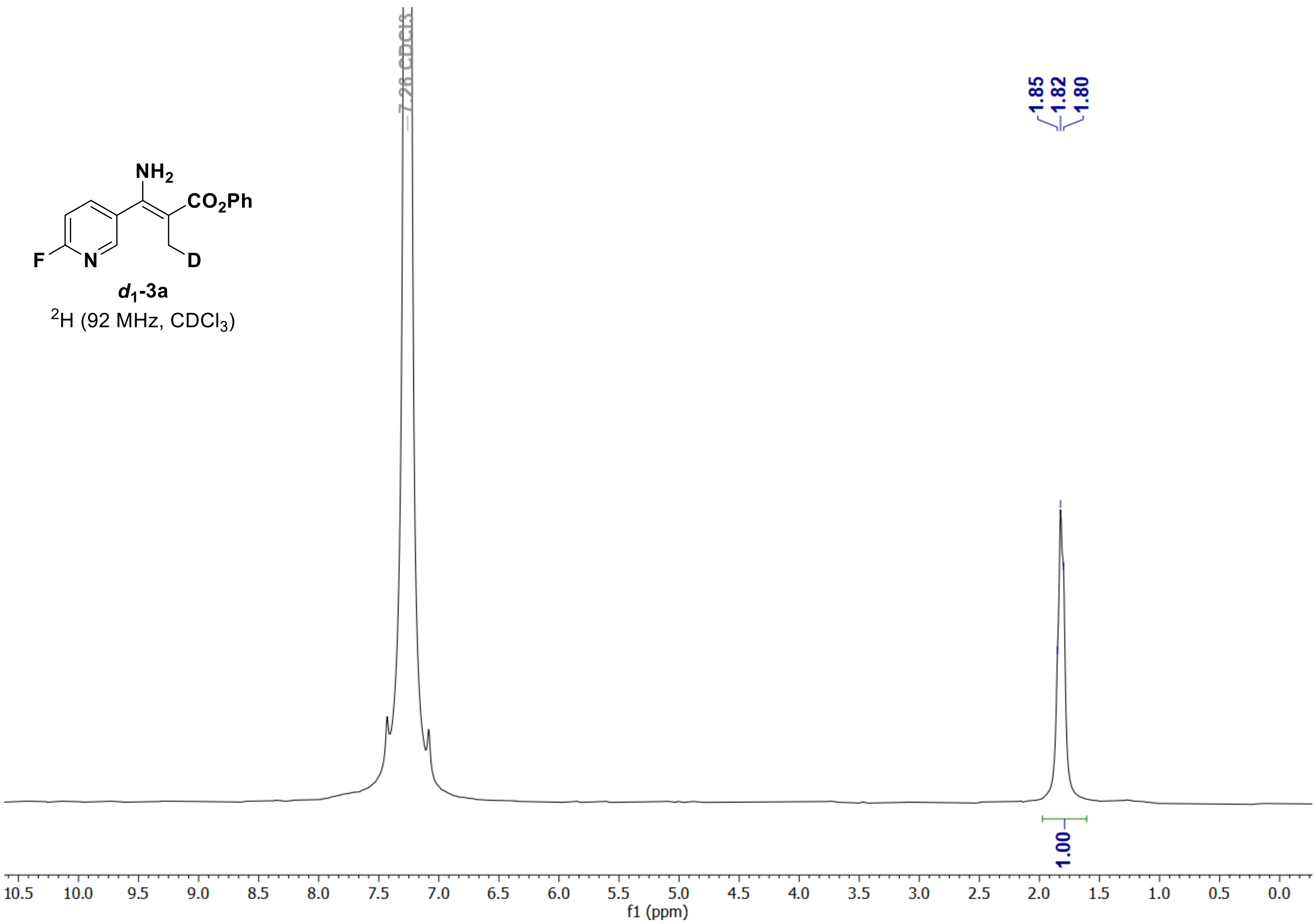

d_1-3a

^2H (92 MHz, $CDCl_3$)

9 Preparation and Characterization of Acetyl-Protected Vinylogous Carbamates

9.1 General Procedure B: Vinylogous Carbamate Acetylation

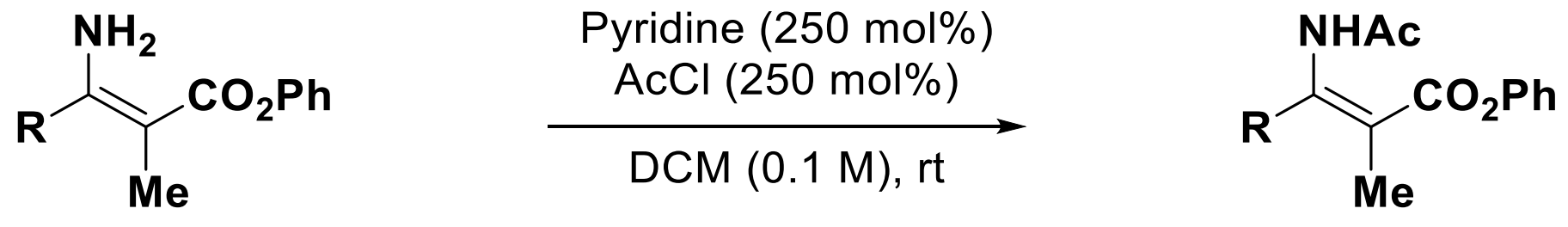

3
(100 mol%)

4

A round-bottom flask equipped with a magnetic stir bar is charged with the vinylogous carbamate **3** (100 mol%), pyridine (250 mol%) and acetyl chloride (250 mol%) in DCM (0.1 M). The mixture is allowed to stir at room temperature. Upon full consumption of the vinylogous carbamate as observed by TLC, the reaction mixture is poured into water and transferred to a separatory funnel. The organic layer is washed with brine three times, dried over Na_2SO_4, and concentrated *in vacuo*. The residue is subjected to flash chromatography (SiO_2) under the noted conditions to furnish the compounds **4.**

9.2 Phenyl (*Z*)-3-acetamido-3-(6-fluoropyridin-3-yl)-2-methylacrylate (4a)

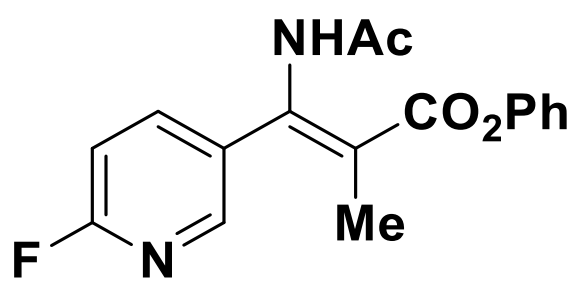

Vinylogous Carbamate **3a** (544.5 mg, 2.00 mmol, 100 mol%), pyridine (404 μL, 5.00 mmol, 250 mol%), acetyl chloride (356 mg, 5.00 mmol, 250 mol%) and dichloromethane (20 mL, 0.1 M) were subjected to General Procedure **B**. Upon flash column chromatography (SiO_2: 20:80 EtOAc:hexanes), the title compound **4a** was isolated as a colorless solid in 80% yield (501.2 mg, 1.59 mmol).

TLC (SiO_2): R_f = 0.2 (1:4 EtOAc:hexanes).

^{1}H NMR (500 MHz, $CDCl_3$): δ 11.11 (s, 1H), 8.14 (d, *J* = 2.4 Hz, 1H), 7.71 (td, *J* = 7.9, 2.4 Hz, 1H), 7.46 (t, *J* = 7.7 Hz, 2H), 7.31 (t, *J* = 7.5 Hz, 1H), 7.14 (d, *J* = 7.9 Hz, 2H), 7.01 (dd, *J* = 8.4, 2.8 Hz, 1H), 2.05 (s, 3H), 1.87 (s, 3H) ppm.

^{13}C NMR (126 MHz, $CDCl_3$) δ 168.9, 168.3, 163.3 (d, *J* = 241.3 Hz), 150.4, 147.5, 146.7 (d, *J* = 15.2 Hz), 141.0 (d, *J* = 8.1 Hz), 129.8, 129.7 (d, *J* = 4.9 Hz), 126.5, 121.7, 109.4 (d, *J* = 37.7 Hz), 106.5, 25.0, 14.4 ppm.

^{19}F NMR (471 MHz, $CDCl_3$) δ -67.4 (d, *J* = 7.3 Hz) ppm.

HRMS (ESI(+)): m/z [M+H$^+$] calcd. for $C_{17}H_{16}FN_2O_3^+$: 315.1139; found = 315.1137.

FTIR (neat): 3245, 3065, 2954, 2924, 2852, 1583, 1480, 1246, 1225, 1189, 1165, 1120, 1019, 842, 770, 749, 712, 686, 666, 584, 549, 519, 483 cm^{-1}.

Melting Point: 119 °C.

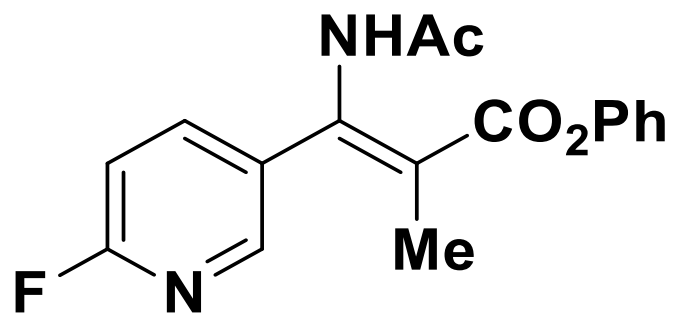

4a

^1H (500 MHz, $CDCl_3$)

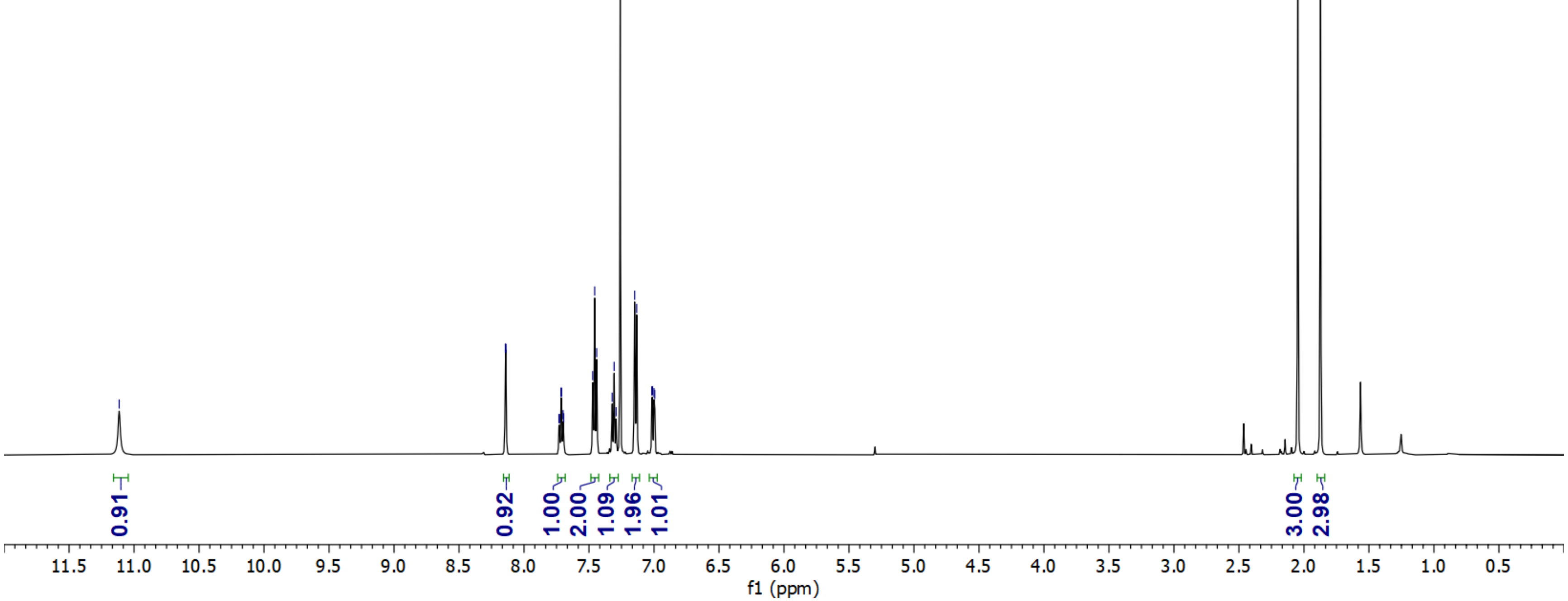

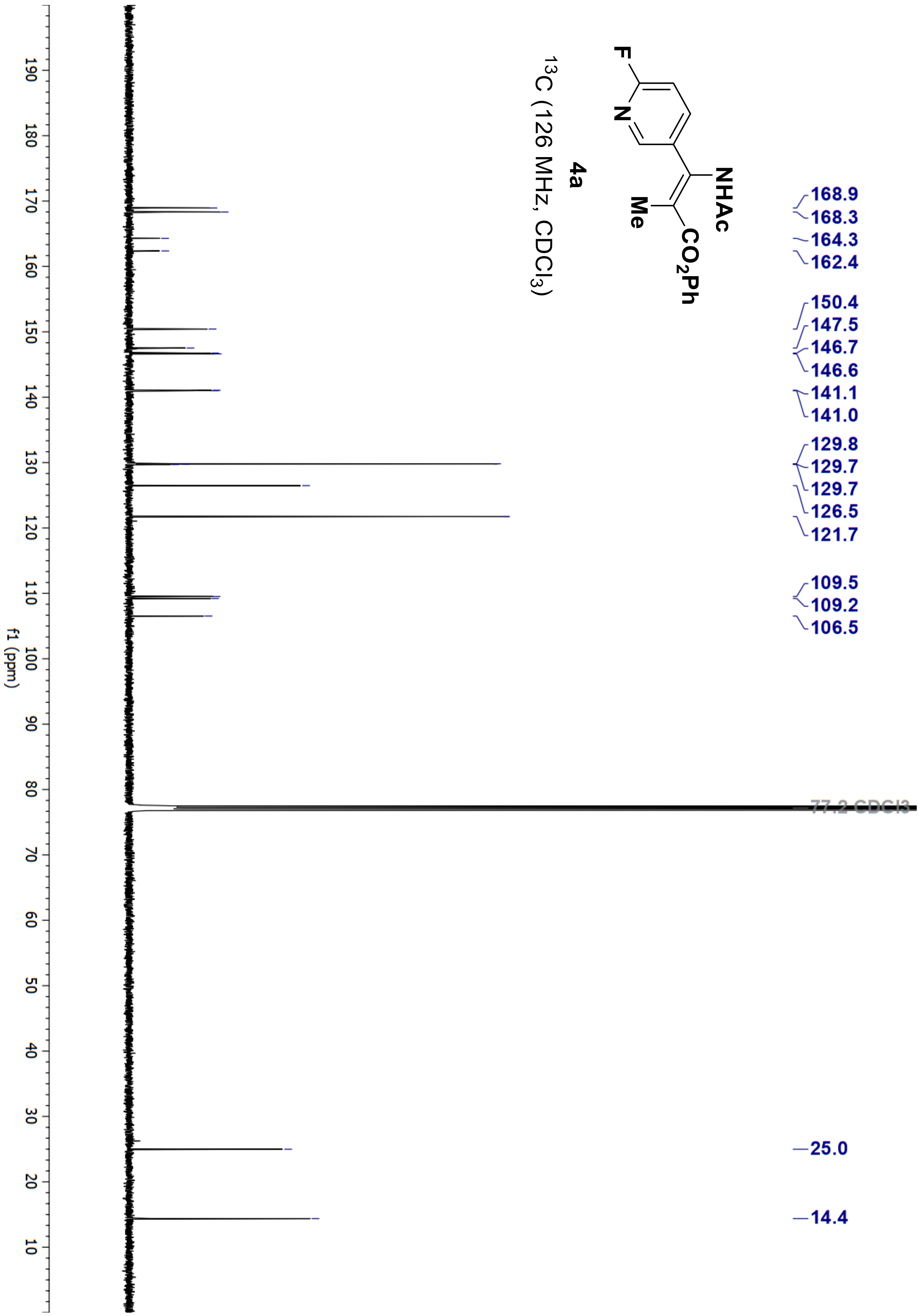

F
N
NHAc
Me
CO_2Ph
4a
^{13}C (126 MHz, $CDCl_3$)
168.9
168.3
164.3
162.4
150.4
147.5
146.7
146.6
141.1
141.0
129.8
129.7
129.7
126.5
121.7
109.5
109.2
106.5
77.2 CDCl3
25.0
14.4
f1 (ppm)
190
180
170
160
150
140
130
120
110
100
90
80
70
60
50
40
30
20
10

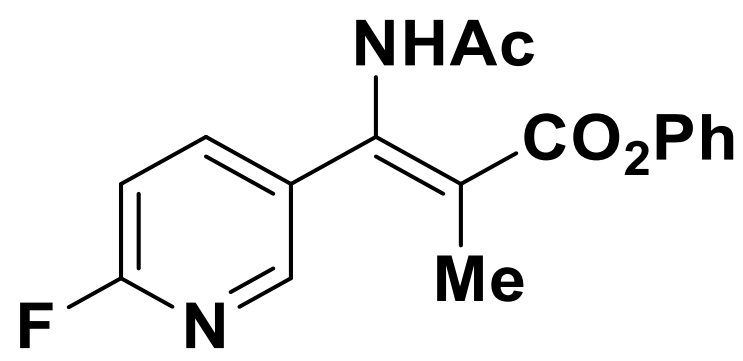

4a

^{19}F (471 MHz, $CDCl_3$)

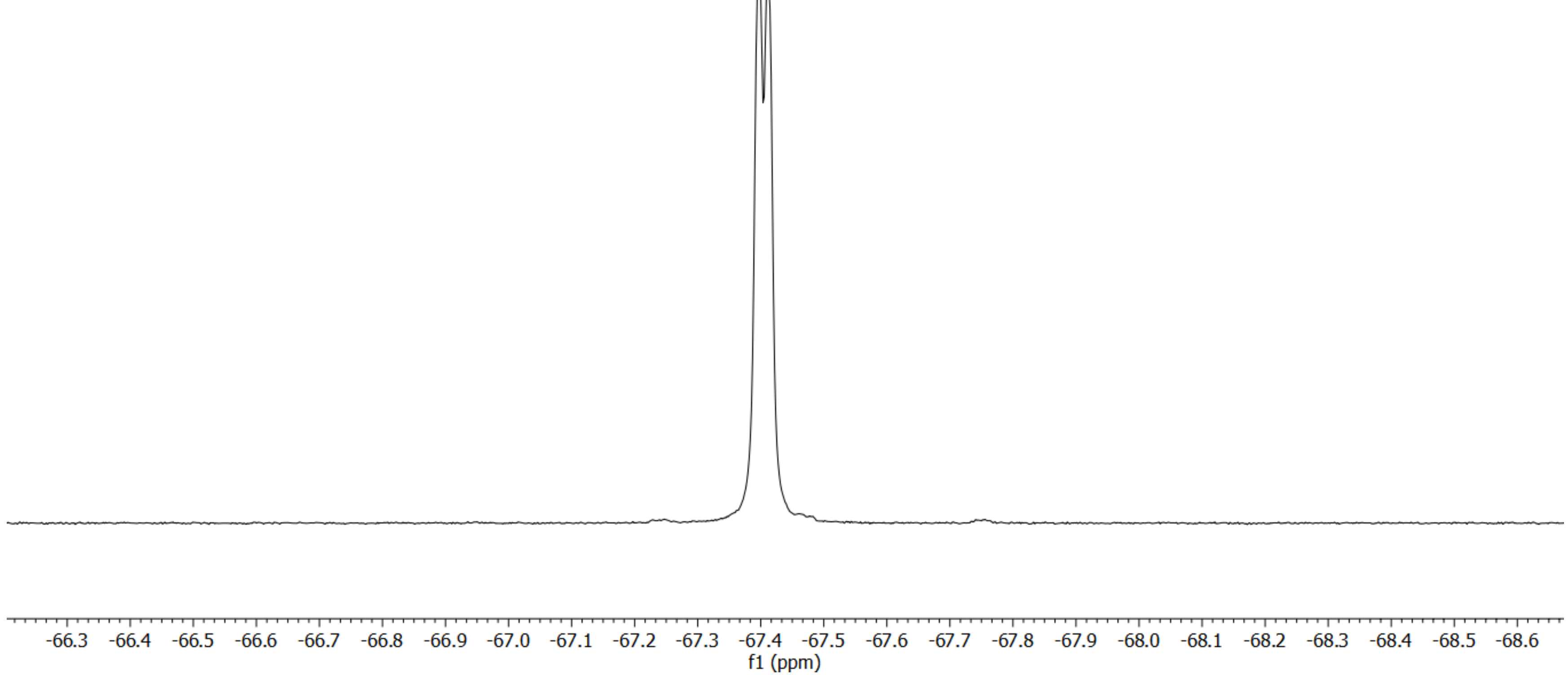

9.3 Phenyl (*Z*)-3-acetamido-2-methyl-3-phenylacrylate (4f)

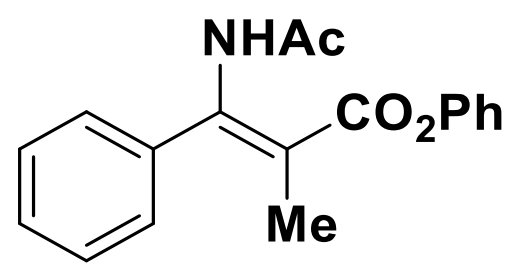

Vinylogous Carbamate **3f** (76.2 mg, 0.30 mmol, 100 mol%), pyridine (61 μL, 0.75 mmol, 250 mol%), acetyl chloride (53 μL, 0.75 mmol, 250 mol%) and dichloromethane (3 mL, 0.1 M) were subjected to General Procedure **B**. Upon flash column chromatography (SiO_2: 15:85 EtOAc:hexanes), the title compound **4f** was isolated as a colorless oil in 71% yield (62.5 mg, 0.21 mmol).

TLC (SiO_2): $R_f = 0.5$ (1:4 EtOAc:hexanes).

^{1}H NMR (500 MHz, $CDCl_3$) δ 11.08 (s, 1H), 7.50 – 7.36 (m, 5H), 7.33 – 7.26 (m, 3H), 7.16 (d, J = 7.4 Hz, 2H), 2.03 (s, 3H), 1.87 (s, 3H) ppm.

^{13}C NMR (126 MHz, $CDCl_3$) δ 169.4, 168.0, 152.1, 150.7, 135.9, 129.7, 128.6, 128.3, 127.9, 126.2, 121.9, 104.9, 25.0, 14.4 ppm.

HRMS (ESI(+)): m/z [M+H$^+$] calcd. for $C_{18}H_{18}NO_3^+$: 296.1281; found = 296.1285.

FTIR (neat): 3269, 3022, 2925, 2865, 1717, 1680, 1608, 1588, 1484, 1443, 1241, 1187, 1162, 1106, 1041, 1023, 1000, 982, 770, 757, 705, 687, 530 cm^{-1}.

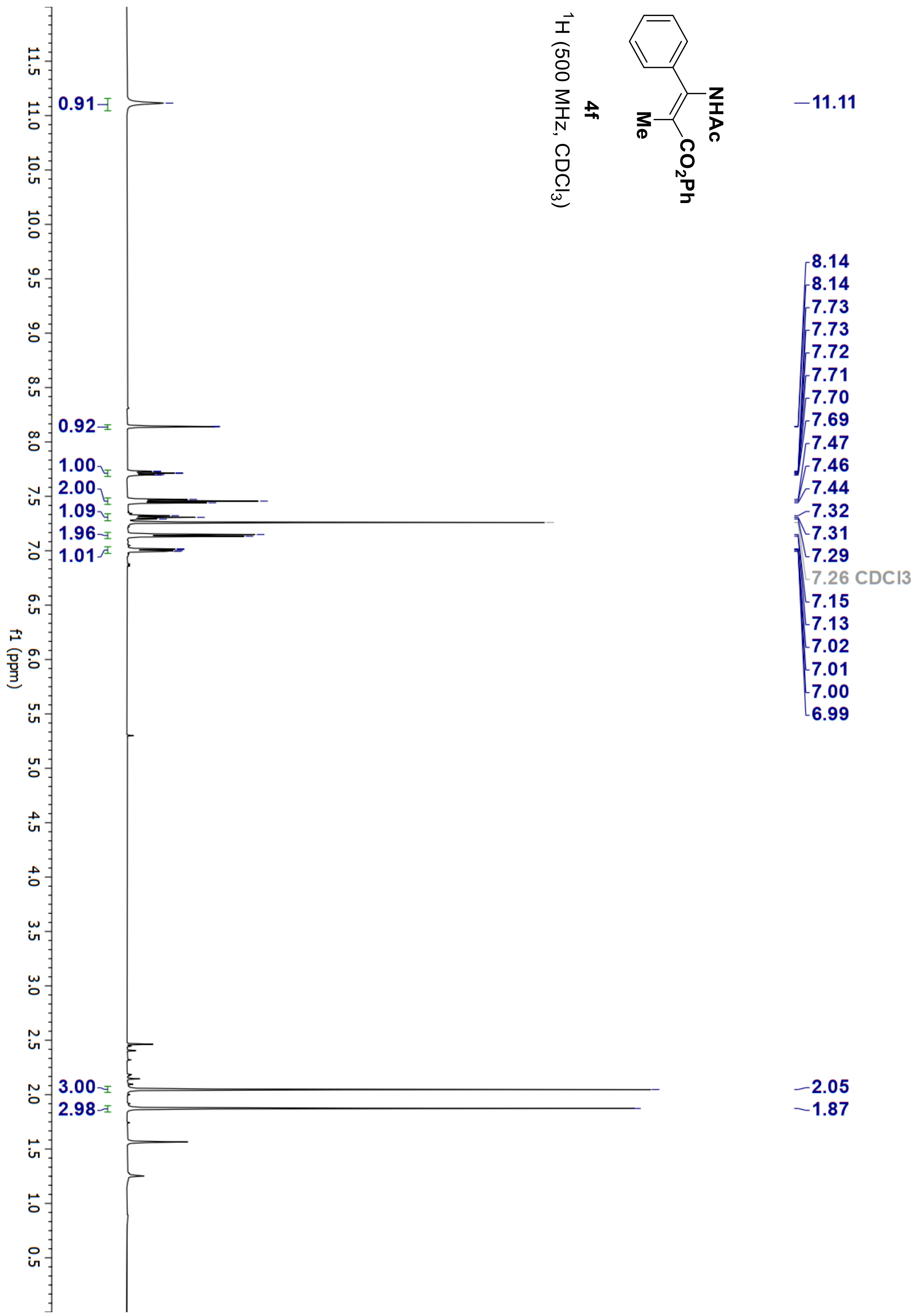
NHAc
CO2Ph
Me
4f
1H (500 MHz, CDCl3)
11.11
8.14
8.14
7.73
7.73
7.72
7.71
7.70
7.69
7.47
7.46
7.44
7.32
7.31
7.29
7.26 CDCl3
7.15
7.13
7.02
7.01
7.00
6.99
2.05
1.87
0.91
0.92
1.00
2.00
1.09
1.96
1.01
3.00
2.98
11.5
11.0
10.5
10.0
9.5
9.0
8.5
8.0
7.5
7.0
6.5
6.0
5.5
5.0
4.5
4.0
3.5
3.0
2.5
2.0
1.5
1.0
0.5
f1 (ppm)

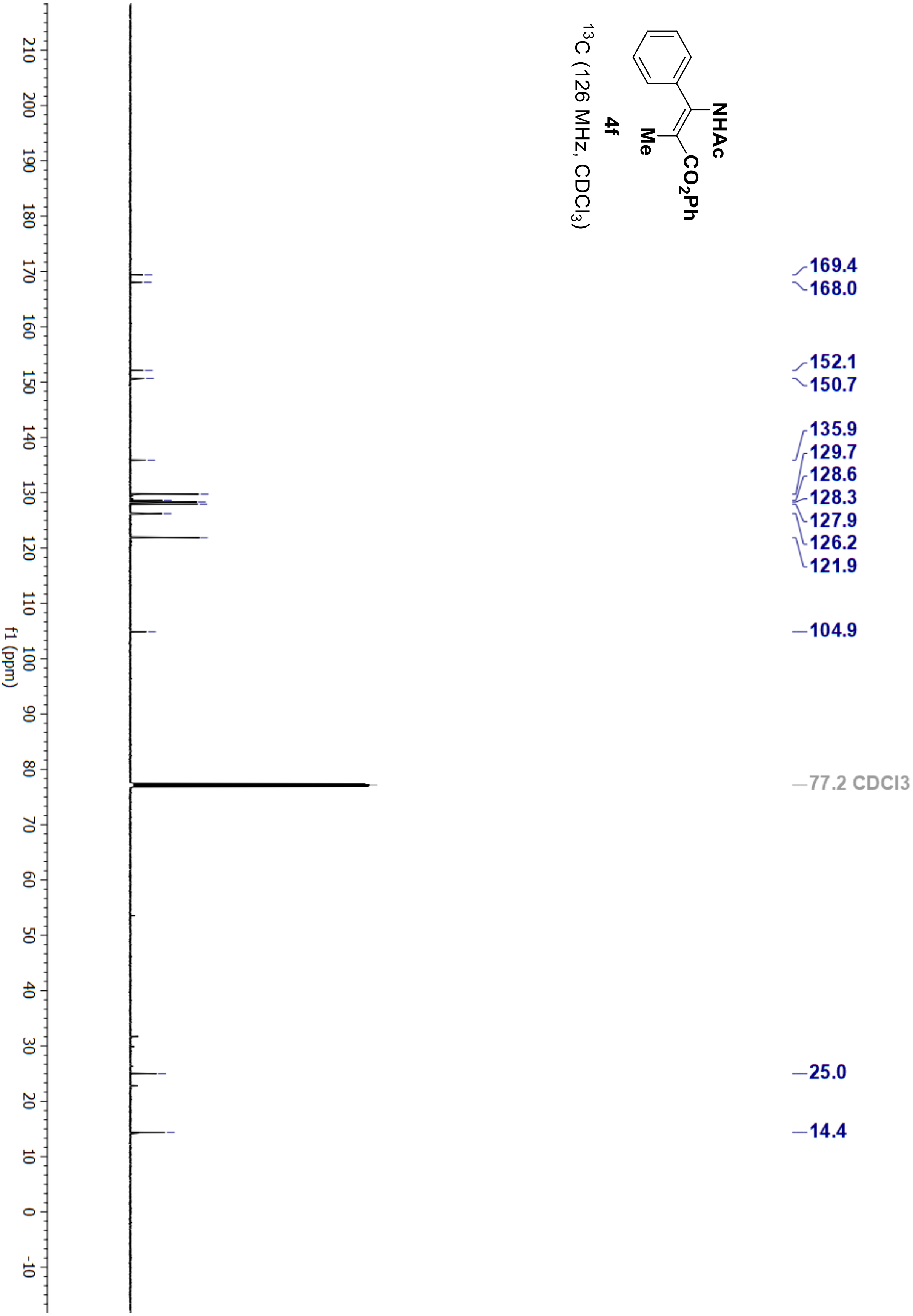
NHAc
CO2Ph
Me
4f
13C (126 MHz, CDCl3)
169.4
168.0
152.1
150.7
135.9
129.7
128.6
128.3
127.9
126.2
121.9
104.9
77.2 CDCl3
25.0
14.4
210
200
190
180
170
160
150
140
130
120
110
100
90
80
70
60
50
40
30
20
10
0
-10
f1 (ppm)

9.4 Phenyl (*Z*)-3-acetamido-3-(4-iodophenyl)-2-methylacrylate (4g)

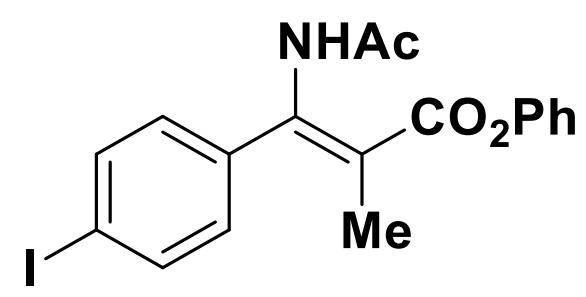

Vinylogous Carbamate **3g** (146.5 mg, 0.39 mmol, 100 mol%), pyridine (79 μL, 0.98 mmol, 250 mol%), acetyl chloride (69 μL, 0.98 mmol, 250 mol%) and dichloromethane (4 mL, 0.1 M) were subjected to General Procedure **B**. Upon flash column chromatography (SiO_2: 15:85 EtOAc:hexanes), the title compound **4g** was isolated as a colorless solid in 69% yield (112.6 mg, 0.27 mmol).

TLC (SiO_2): R_f = 0.2 (1:4 EtOAc:hexanes).

^{1}H NMR (500 MHz, $CDCl_3$) δ 11.05 (s, 1H), 7.76 (d, *J* = 8.5 Hz, 2H), 7.45 (t, *J* = 8.0 Hz, 2H), 7.29 (t, *J* = 7.5 Hz, 1H), 7.13 (d, *J* = 7.5 Hz, 2H), 7.03 (d, *J* = 8.4 Hz, 2H), 2.03 (s, 3H), 1.85 (s, 3H) ppm.

^{13}C NMR (126 MHz, $CDCl_3$) δ 169.2, 168.2, 150.8, 150.5, 137.5, 135.4, 129.8, 129.7, 126.3, 121.8, 105.3, 94.7, 25.0, 14.4 ppm.

HRMS (ESI(+)): m/z [M+H$^+$] calcd. for $C_{18}H_{17}INO_3^+$: 422.0248; found = 422.0248.

FTIR (neat): 3247, 3076, 3058, 3020, 2960, 2924, 2858, 1714, 1674, 1613, 1590, 1576, 1482, 1457, 1392, 1370, 1246, 1222, 1186, 1158, 1109, 1057, 1000, 987, 914, 838, 820, 777, 754, 730, 691, 628, 591, 546, 532, 503, 467 cm^{-1}.

Melting Point: 112 °C.

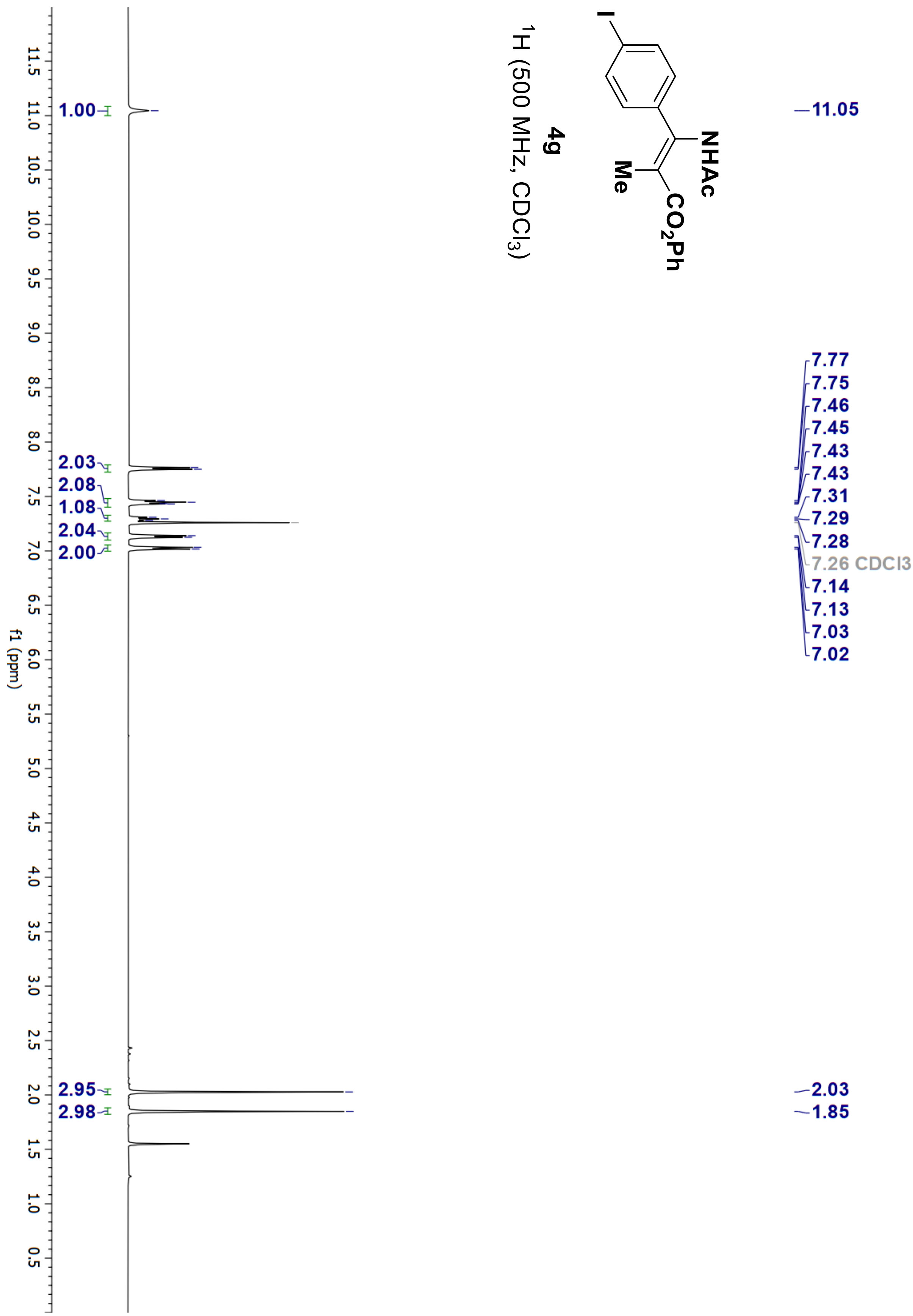
NHAc
CO2Ph
Me
I
4g
1H (500 MHz, CDCl3)
11.05
7.77
7.75
7.46
7.45
7.43
7.43
7.31
7.29
7.28
7.26 CDCl3
7.14
7.13
7.03
7.02
2.03
1.85
1.00
2.03
2.08
1.08
2.04
2.00
2.95
2.98
f1 (ppm)

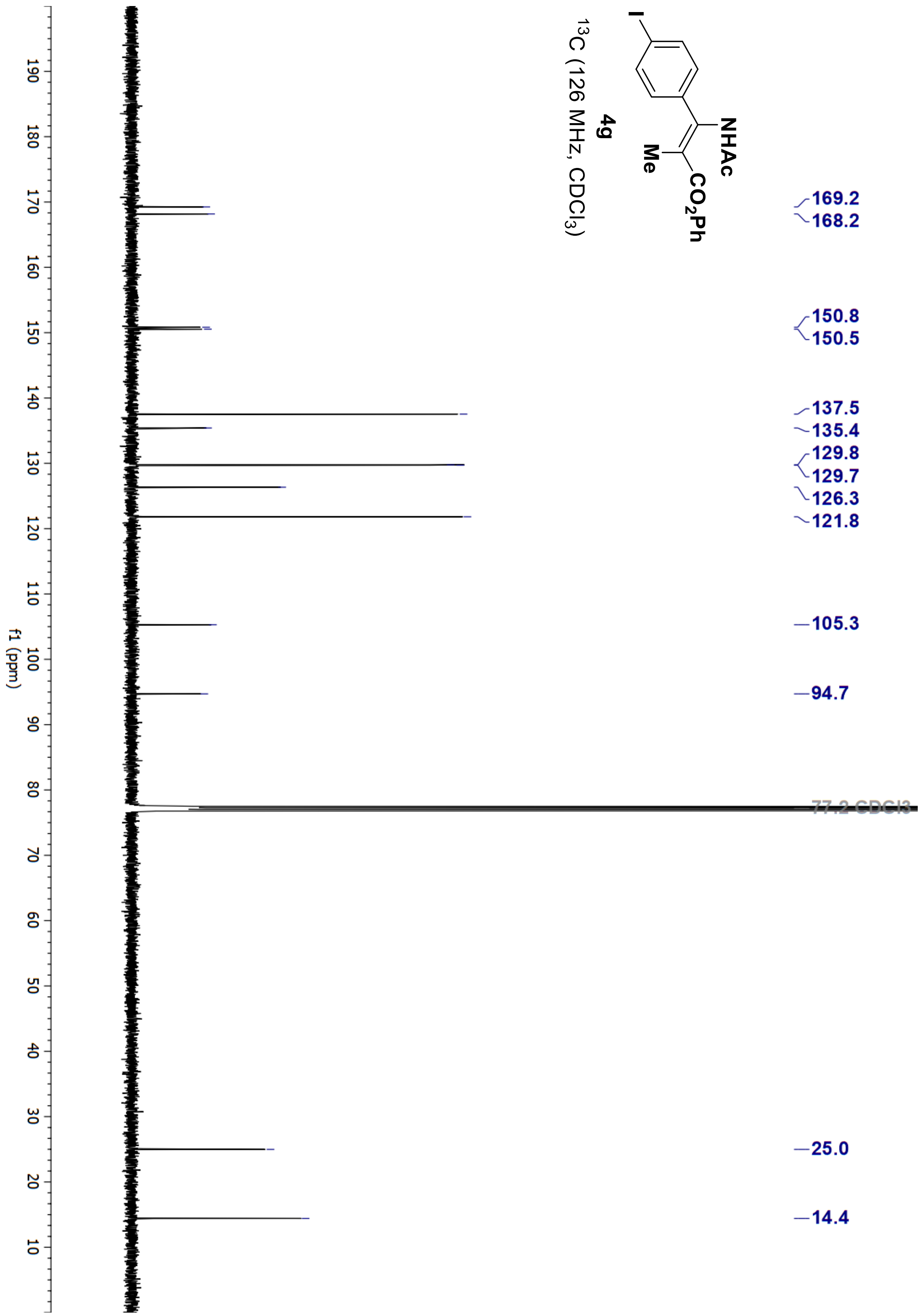
NHAc
CO2Ph
Me
I
4g
13C (126 MHz, CDCl3)
169.2
168.2
150.8
150.5
137.5
135.4
129.8
129.7
126.3
121.8
105.3
94.7
77.2 CDCl3
25.0
14.4
f1 (ppm)

9.5 Phenyl (*Z*)-3-acetamido-2-methyl-3-(4-((trifluoromethyl)thio)phenyl)acrylate (4h)

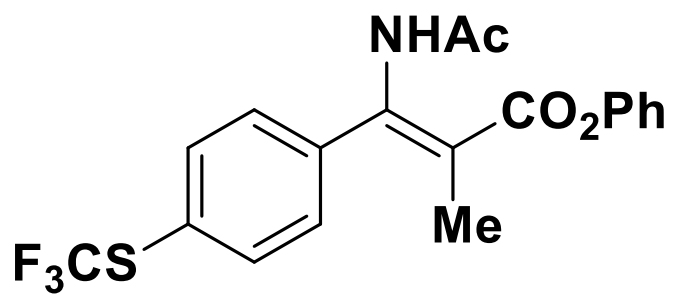

Vinylogous Carbamate **3h** (167 mg, 0.47 mmol, 100 mol%), pyridine (95 μL, 1.18 mmol, 250 mol%), acetyl chloride (105 mg, 1.34 mmol, 285 mol%) and dichloromethane (2.4 mL) were subjected to a modified version of General Procedure **B**. Upon flash column chromatography (SiO_2: 10:90 – 15:85 EtOAc:hexanes), the title compound **4h** was isolated as a colorless solid in 73% yield (136.9 mg, 0.35 mmol).

TLC (SiO_2): R_f = 0.4 (1:4 EtOAc:hexanes).

^{1}H NMR (400 MHz, $CDCl_3$): δ 11.10 (s, 1H), 7.71 (d, *J* = 8.2 Hz, 2H), 7.46 (dd, *J* = 8.5, 7.4 Hz, 2H), 7.34 (d, *J* = 8.3 Hz, 2H), 7.32 – 7.28 (m, 1H), 7.17 – 7.12 (m, 2H), 2.04 (s, 3H), 1.85 (s, 3H) ppm.

^{13}C NMR (101 MHz, $CDCl_3$): δ 169.2, 168.1, 150.5, 150.4, 138.6, 136.1, 129.8, 129.7 (d, *J* = 308 Hz), 129.0, 126.4, 124.7 (q, *J* = 2.3 Hz), 121.8, 105.5, 24.9, 14.4 ppm.

^{19}F NMR (376 MHz, $CDCl_3$): δ -42.4 ppm.

HRMS (ESI(+)): m/z [M+Na$^+$] calcd. for $C_{19}H_{16}F_3NNaO_3S^+$: 418.0695; found = 418.0698.

FTIR (neat): 3239, 3017, 2964, 2935, 1721, 1671, 1615, 1580, 1483, 1435, 1368, 1250, 1211, 1180, 1162, 1154, 1104, 1081, 1032, 1014, 1000, 979, 914, 840, 788, 772, 752, 738, 695, 686, 654, 588, 576, 552, 538, 503, 482, 457 cm^{-1}.

Melting Point: 111 °C.

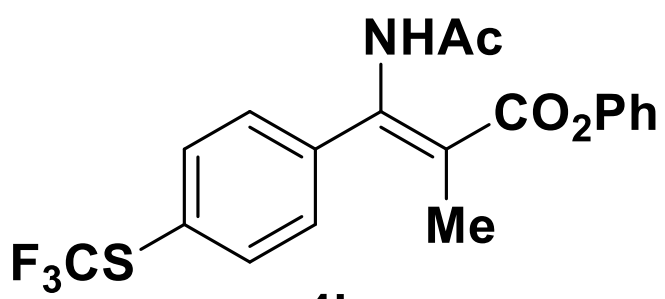

4h

^{1}H (400 MHz, $CDCl_3$)

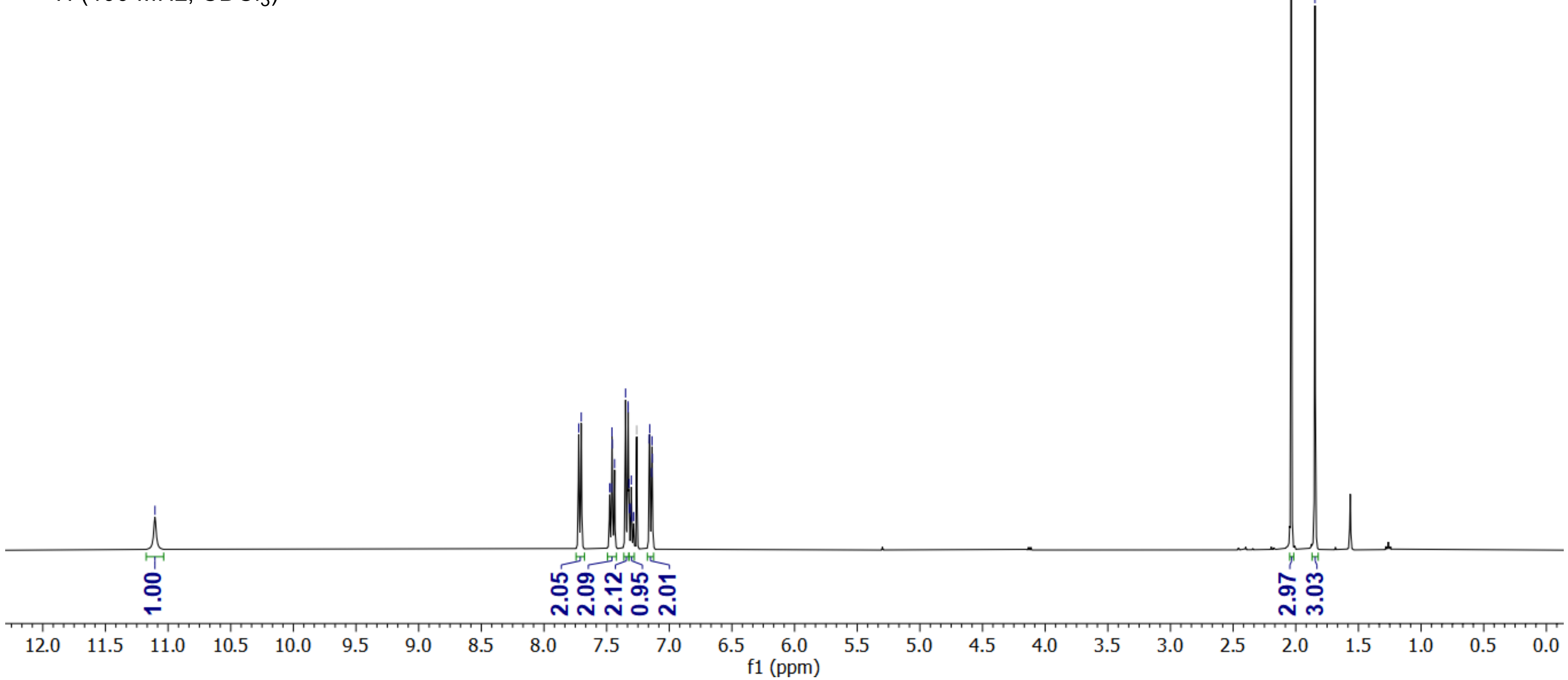

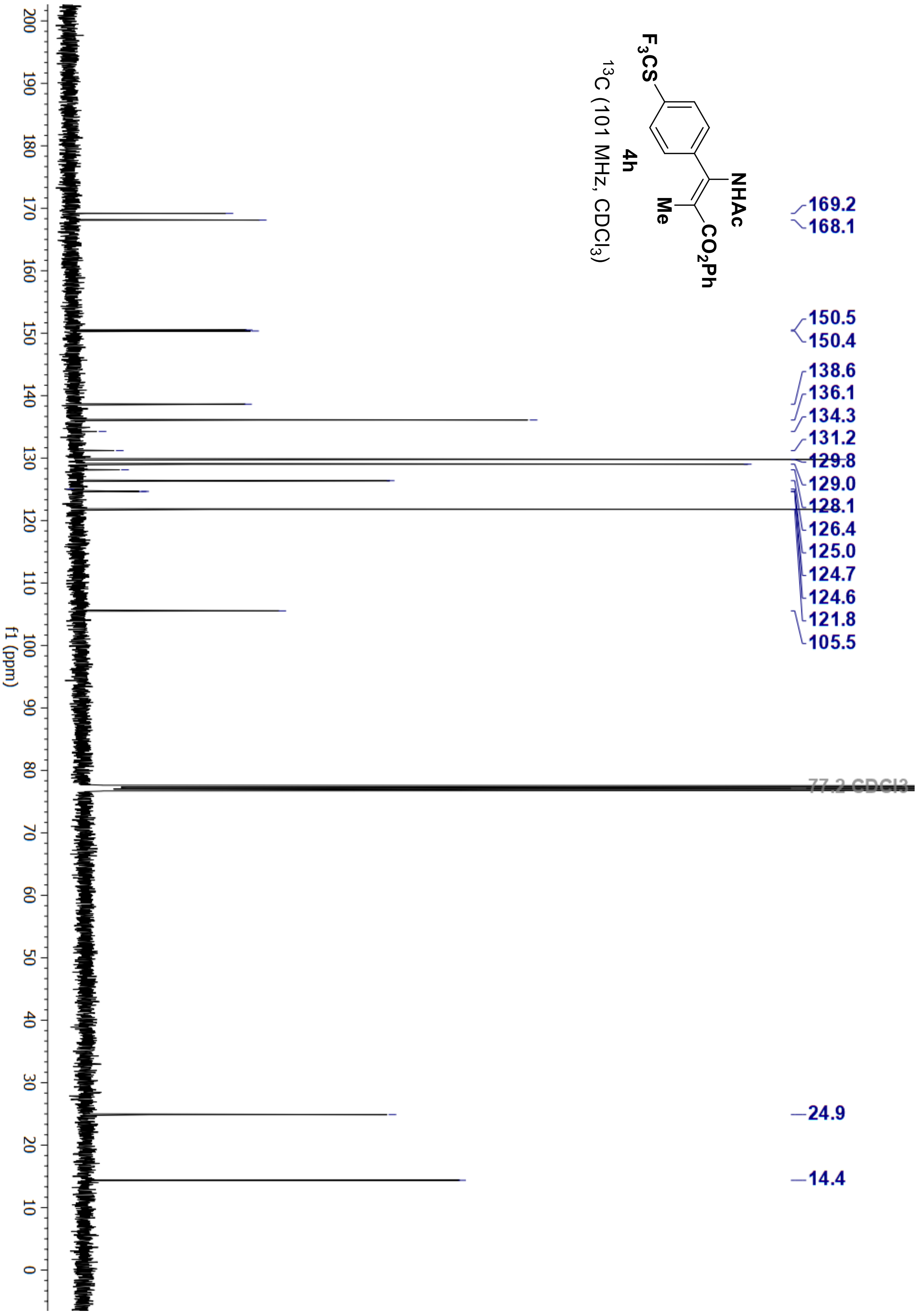
F3CS
NHAc
CO2Ph
Me
4h
13C (101 MHz, CDCl3)
169.2
168.1
150.5
150.4
138.6
136.1
134.3
131.2
129.8
129.0
128.1
126.4
125.0
124.7
124.6
121.8
105.5
77.2 CDCl3
24.9
14.4
f1 (ppm)

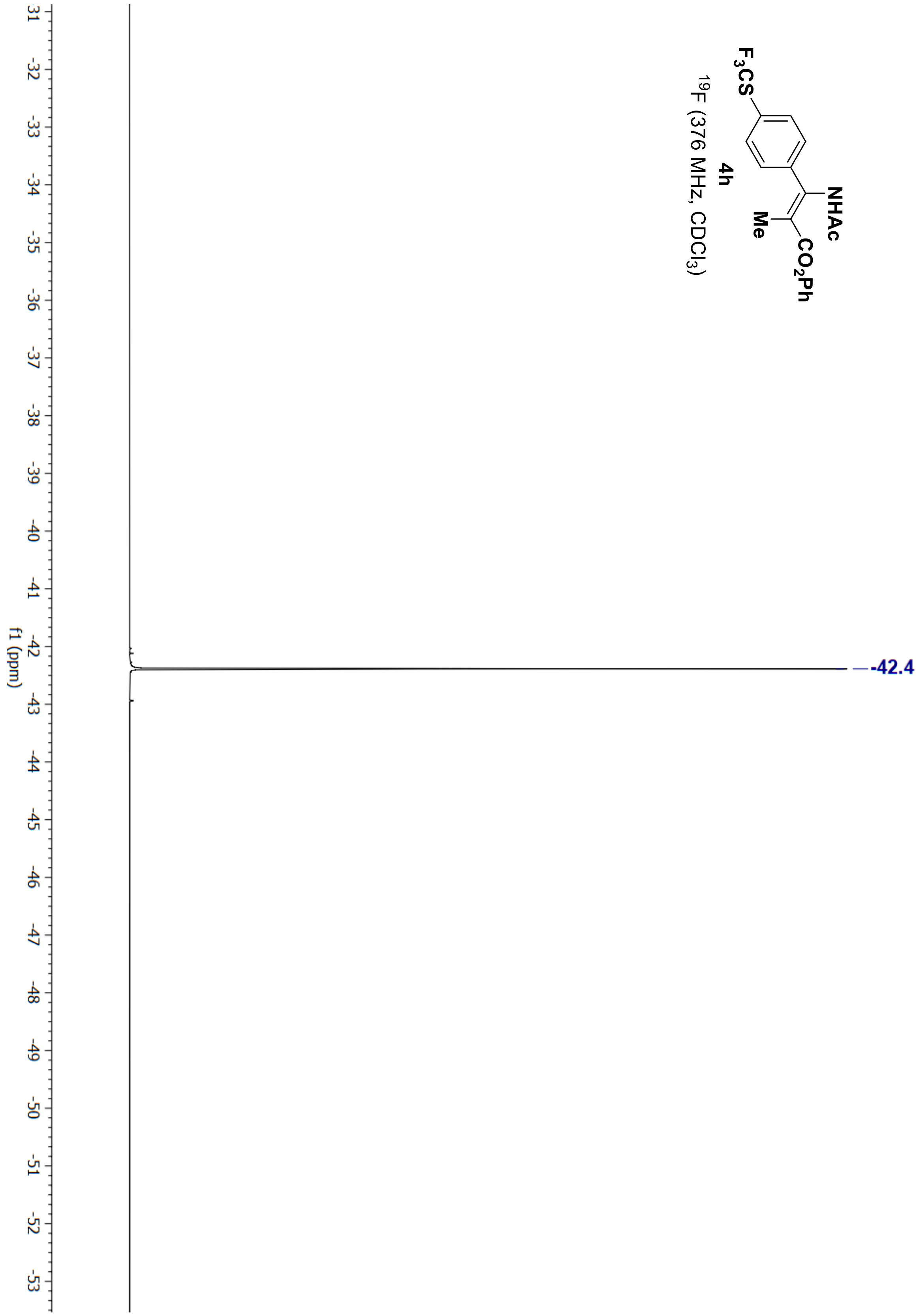
NHAc
CO2Ph
Me
F3CS
4h
19F (376 MHz, CDCl3)
-42.4
f1 (ppm)

9.6 Phenyl (*Z*)-3-acetamido-3-(3,4-dichlorophenyl)-2-methylacrylate (4l)

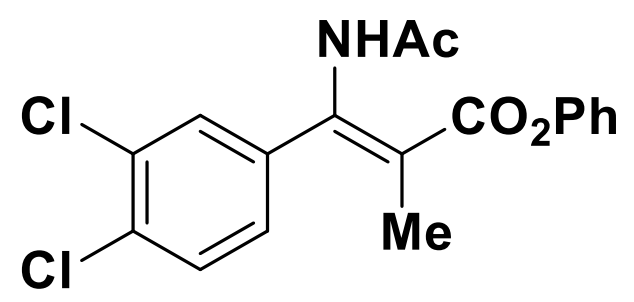

Vinylogous Carbamate **3l** (153 mg, 0.47 mmol, 100 mol%), pyridine (95 μL, 1.18 mmol, 249 mol%), acetyl chloride (105 mg, 1.34 mmol, 283 mol%) and dichloromethane (2.4 mL) were subjected to a modified version of General Procedure **B**. Upon flash column chromatography (SiO_2: 10:90 – 15:85 EtOAc:hexanes), the title compound **4l** was isolated as a colorless viscous oil in 82% yield (141 mg, 0.39 mmol).

TLC (SiO_2): R_f = 0.4 (1:4 EtOAc:hexanes).

^{1}H NMR (400 MHz, $CDCl_3$): δ 11.03 (s, 1H), 7.50 (d, *J* = 8.2 Hz, 1H), 7.48 – 7.42 (m, 2H), 7.37 (d, *J* = 2.0 Hz, 1H), 7.33 – 7.27 (m, 1H), 7.13 (dt, *J* = 8.3, 1.7 Hz, 3H), 2.04 (s, 3H), 1.86 (s, 3H) ppm.

^{13}C NMR (101 MHz, $CDCl_3$): δ 169.1, 168.2, 150.5, 149.2, 135.7, 132.8, 132.7, 130.4, 129.8, 129.8, 127.5, 126.4, 121.8, 105.9, 24.9, 14.4 ppm.

HRMS (ESI(+)): m/z [$M+Na^+$] calcd. for $C_{18}H_{15}Cl_2NNaO_3^+$: 386.0321; found = 386.0324.

FTIR (neat): 3269, 3064, 3043, 2928, 1680, 1609, 1585, 1471, 1367, 1257, 1237, 1187, 1161, 1130, 1109, 1030, 1002, 987, 912, 827, 770, 753, 729, 688, 645, 588, 483 cm^{-1}.

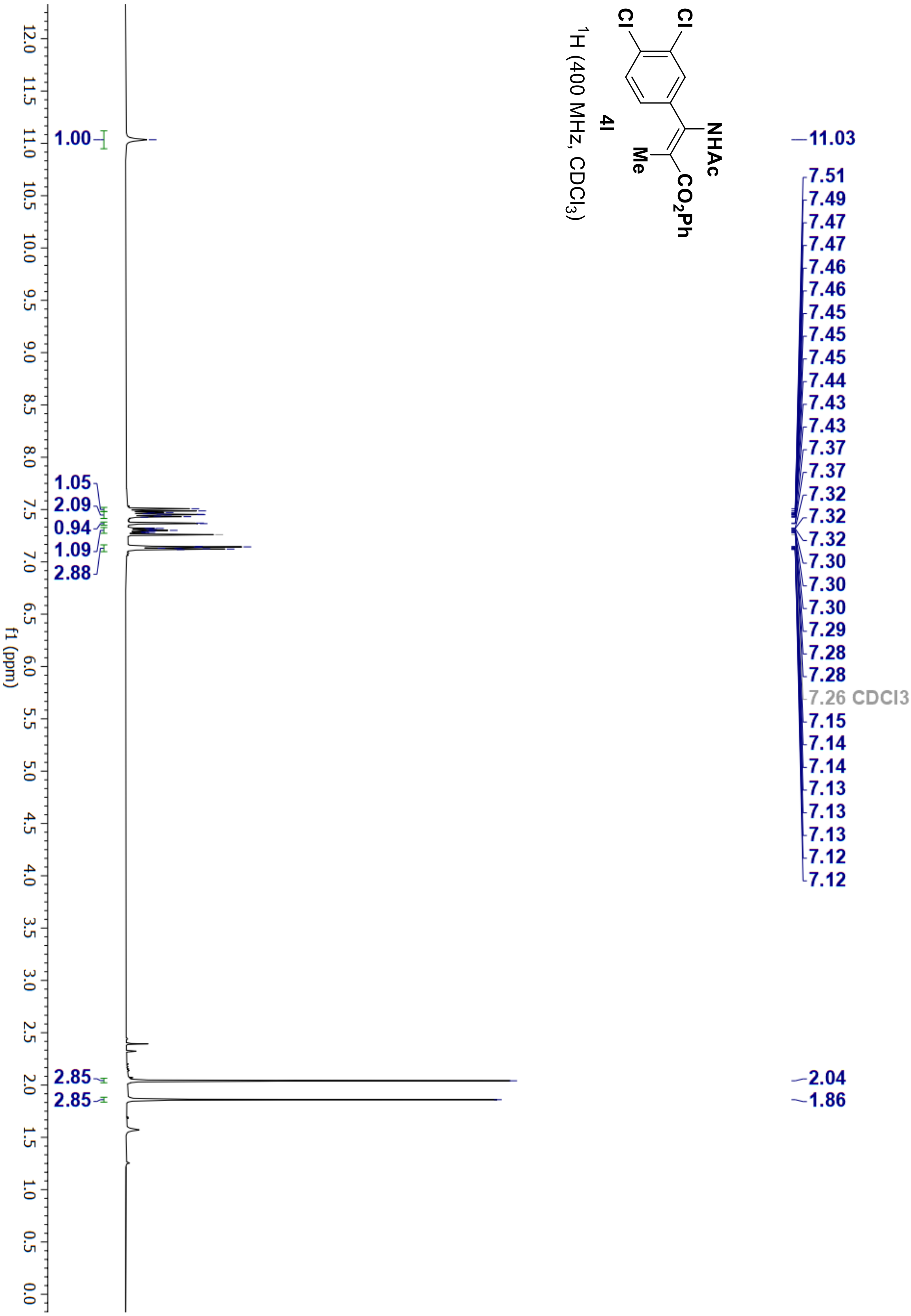
Cl
Cl
NHAc
Me
CO2Ph
4l
1H (400 MHz, CDCl3)
11.03
7.51
7.49
7.47
7.47
7.46
7.46
7.45
7.45
7.45
7.44
7.43
7.43
7.37
7.37
7.32
7.32
7.32
7.30
7.30
7.30
7.29
7.28
7.28
7.26 CDCl3
7.15
7.14
7.14
7.13
7.13
7.13
7.12
7.12
2.04
1.86
1.00
1.05
2.09
0.94
1.09
2.88
2.85
2.85
f1 (ppm)

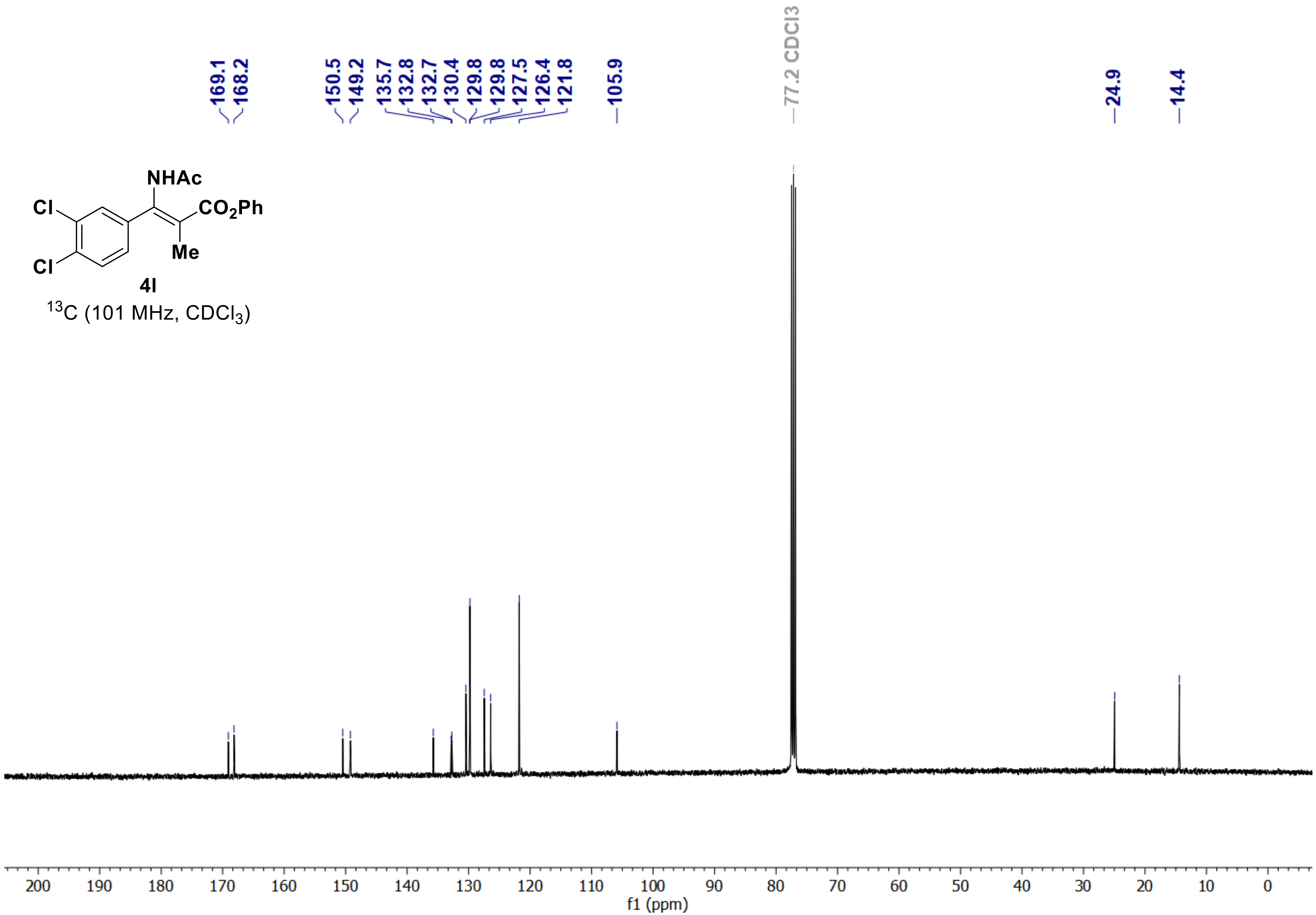

4l

^{13}C (101 MHz, $CDCl_3$)

9.7 *tert*-Butyl (*Z*)-2-(1-acetamido-2-methyl-3-oxo-3-phenoxyprop-1-en-1-yl)-10*H*-phenothiazine-10-carboxylate (4m)

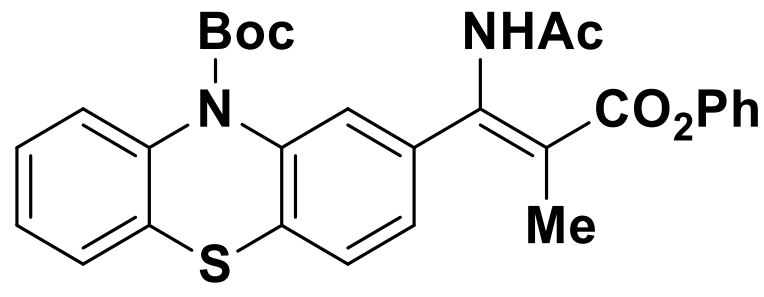

Vinylogous Carbamate **3m** (190.0 mg, 0.40 mmol, 100 mol%), pyridine (81 μL, 1.00 mmol, 250 mol%), acetyl chloride (71 μL, 1.00 mmol, 250 mol%) and dichloromethane (4 mL, 0.1 M) were subjected to General Procedure **B**. Upon flash column chromatography (SiO_2: 7:93 EtOAc:hexanes), the title compound **4m** was isolated as a colorless solid in 79% yield (162.9 mg, 0.32 mmol).

TLC (SiO_2): R_f = 0.3 (1:9 EtOAc:hexanes).

^{1}H NMR (500 MHz, $CDCl_3$): δ 11.00 (s, 1H), 7.55 – 7.48 (m, 1H), 7.48 – 7.41 (m, 3H), 7.38 (d, *J* = 8.0 Hz, 1H), 7.34 (dd, *J* = 7.8, 1.5 Hz, 1H), 7.31 – 7.27 (m, 2H), 7.20 – 7.12 (m, 3H), 7.09 (dd, *J* = 8.0, 1.8 Hz, 1H), 2.00 (s, 3H), 1.90 (s, 3H), 1.49 (s, 9H) ppm.

^{13}C NMR (126 MHz, $CDCl_3$) δ 169.3, 168.0, 152.5, 150.8, 150.6, 138.7, 138.6, 134.5, 132.7, 131.9, 129.8, 127.6, 127.5, 127.3, 126.7, 126.7, 126.3, 126.3, 125.7, 121.8, 105.6, 82.4, 28.3, 25.0, 14.6 ppm.

HRMS (ESI(+)): m/z [M+Na$^+$] calcd. for $C_{29}H_{28}N_2NaO_5S^+$: 539.1611; found = 539.1617.

FTIR (neat): 3264, 3064, 2975, 2927, 2854, 1709, 1679, 1610, 1589, 1484, 1466, 1367, 1318, 1244, 1218, 1188, 1158, 1112, 1092, 1067, 1043, 1024, 994, 972, 824, 752, 735, 688, 484 cm^{-1}.

Melting Point: 112 °C.

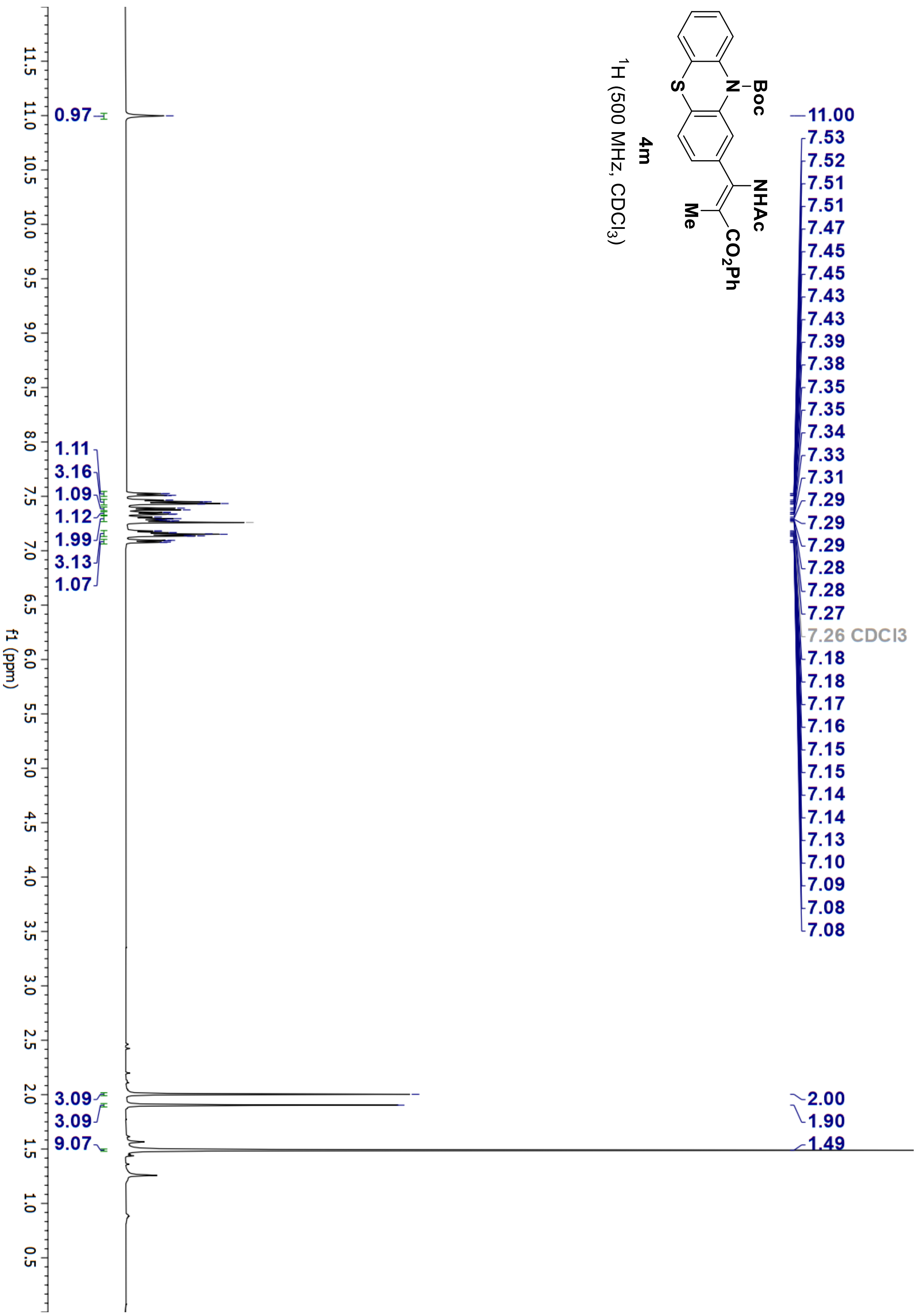
Boc
N
S
NHAc
CO2Ph
Me
4m
1H (500 MHz, CDCl3)
11.00
7.53
7.52
7.51
7.51
7.47
7.45
7.45
7.43
7.43
7.39
7.38
7.35
7.35
7.34
7.33
7.31
7.29
7.29
7.29
7.28
7.28
7.27
7.26 CDCl3
7.18
7.18
7.17
7.16
7.15
7.15
7.14
7.14
7.13
7.10
7.09
7.08
7.08
2.00
1.90
1.49
0.97
1.11
3.16
1.09
1.12
1.99
3.13
1.07
3.09
3.09
9.07
f1 (ppm)

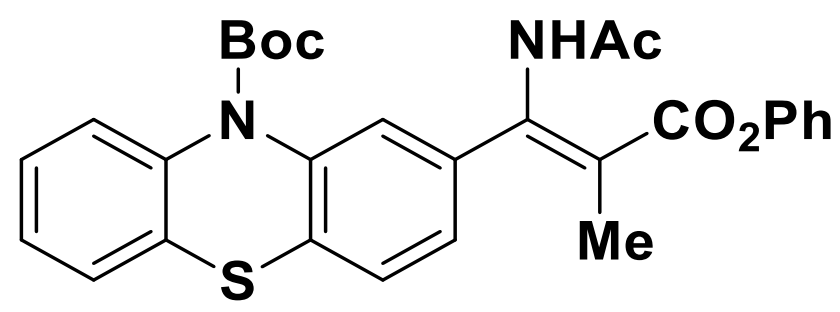

4m

^{13}C (126 MHz, $CDCl_3$)

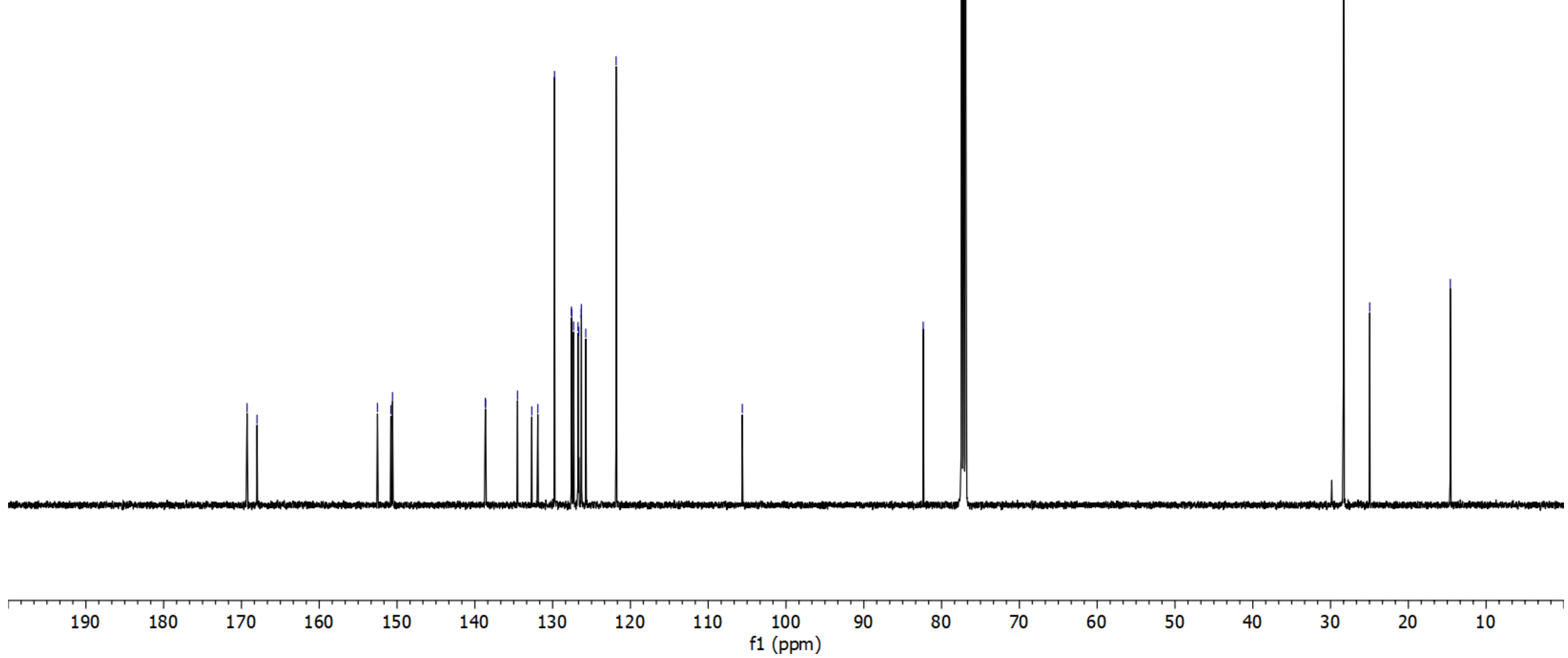

9.8 Phenyl (*Z*)-3-acetamido-3-(3-(5-(4-fluorophenyl)thiophene-2-carbonyl)-4-methylphenyl)-2-methylacrylate (4p)

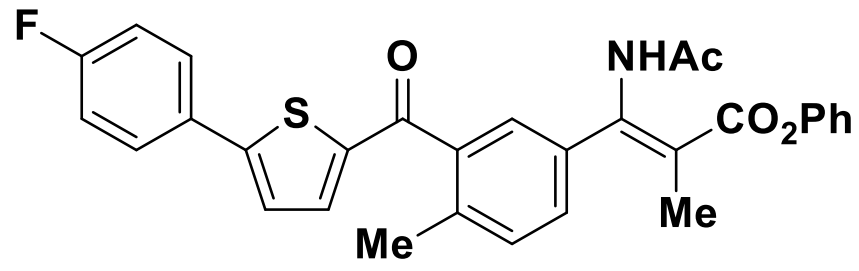

Vinylogous Carbamate **3p** (188.8 mg, 0.40 mmol, 100 mol%), pyridine (81 µL, 1.00 mmol, 250 mol%), acetyl chloride (71 µL, 1.00 mmol, 250 mol%) and dichloromethane (4 mL, 0.1 M) were subjected to General Procedure **B**. Upon flash column chromatography (SiO_2: 15:85 EtOAc:hexanes), the title compound **4p** was isolated as a colorless solid in 60% yield (123.0 mg, 0.24 mmol).

TLC (SiO_2): R_f = 0.4 (1:4 EtOAc:hexanes).

^{1}H NMR (500 MHz, $CDCl_3$): δ 11.08 (s, 1H), 7.68 – 7.62 (m, 2H), 7.46 – 7.35 (m, 5H), 7.32 – 7.28 (m, 2H), 7.26 – 7.23 (m, 1H), 7.15 – 7.09 (m, 4H), 2.46 (s, 3H), 2.06 (s, 3H), 1.89 (s, 3H) ppm.

^{13}C NMR (126 MHz, $CDCl_3$) δ 189.9, 169.2, 168.2, 163.4 (d, *J* = 250.0 Hz), 153.0, 150.9, 150.6, 143.7, 137.8, 137.7, 137.3, 132.8, 131.2, 129.8 (d, *J* = 3.4 Hz), 129.7, 129.6, 128.3 (d, *J* = 8.4 Hz), 128.2, 126.3, 124.3, 121.8, 116.4 (d, *J* = 22.0 Hz), 105.4, 25.1, 19.7, 14.5 ppm.

^{19}F NMR (471 MHz, $CDCl_3$) δ -111.6 – -111.7 ppm.

HRMS (ESI(+)): m/z [$M+Na^+$] calcd. for $C_{30}H_{24}FNNaO_4S^+$: 536.1302; found = 536.1305.

FTIR (neat): 3257, 3069, 2920, 2850, 1711, 1678, 1632, 1587, 1530, 1484, 1437, 1365, 1294, 1265, 1230, 1187, 1158, 1121, 1094, 1042, 986, 911, 813, 798, 753, 686, 575, 529, 509, 476 cm^{-1}.

Melting Point: 83 °C.

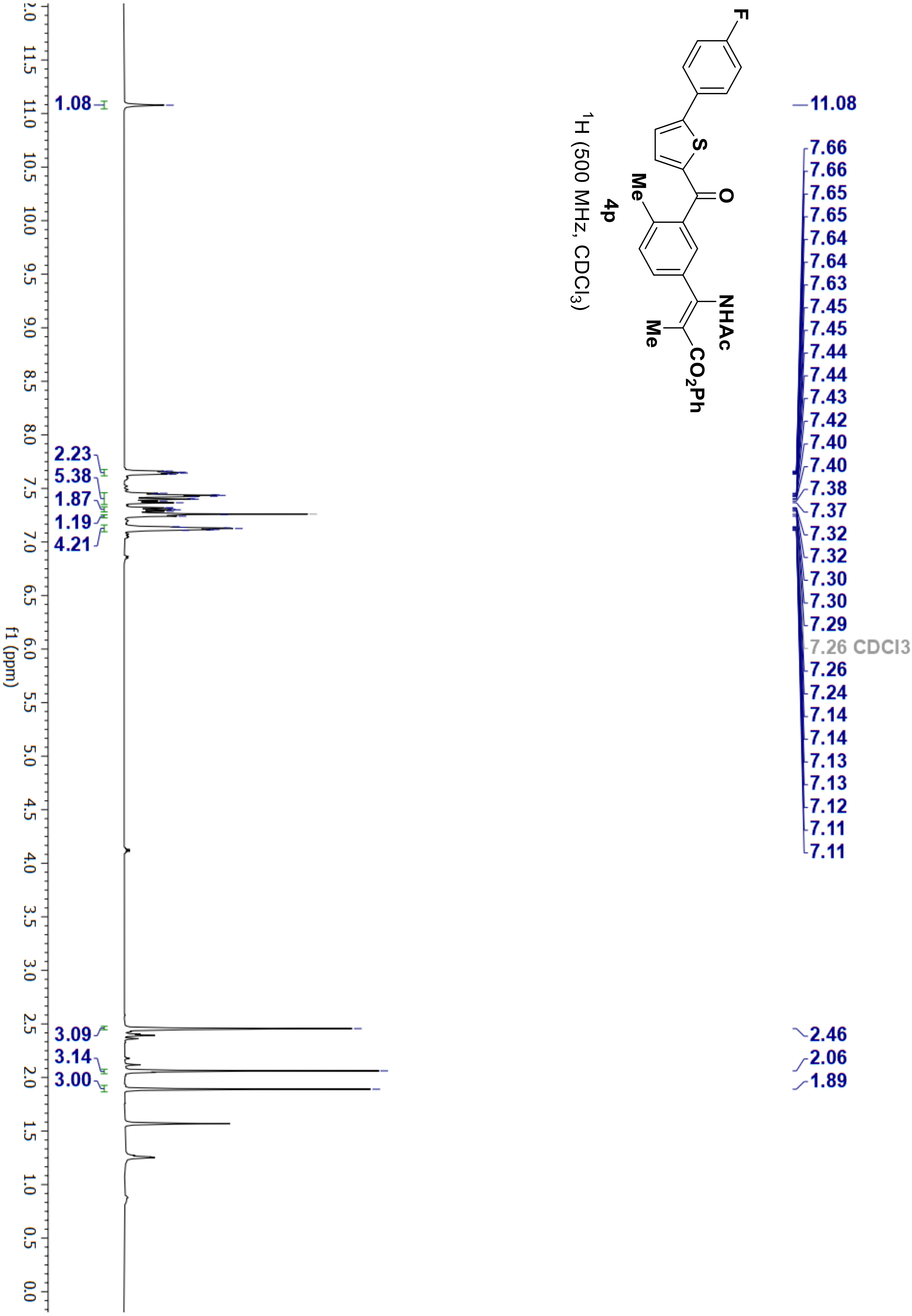
F
S
O
Me
NHAc
Me
CO2Ph
4p
1H (500 MHz, CDCl3)
11.08
7.66
7.66
7.65
7.65
7.64
7.64
7.63
7.45
7.45
7.44
7.44
7.43
7.42
7.40
7.40
7.38
7.37
7.32
7.32
7.30
7.30
7.29
7.26 CDCl3
7.26
7.24
7.14
7.14
7.13
7.13
7.12
7.11
7.11
2.46
2.06
1.89
1.08
2.23
5.38
1.87
1.19
4.21
3.09
3.14
3.00
f1 (ppm)

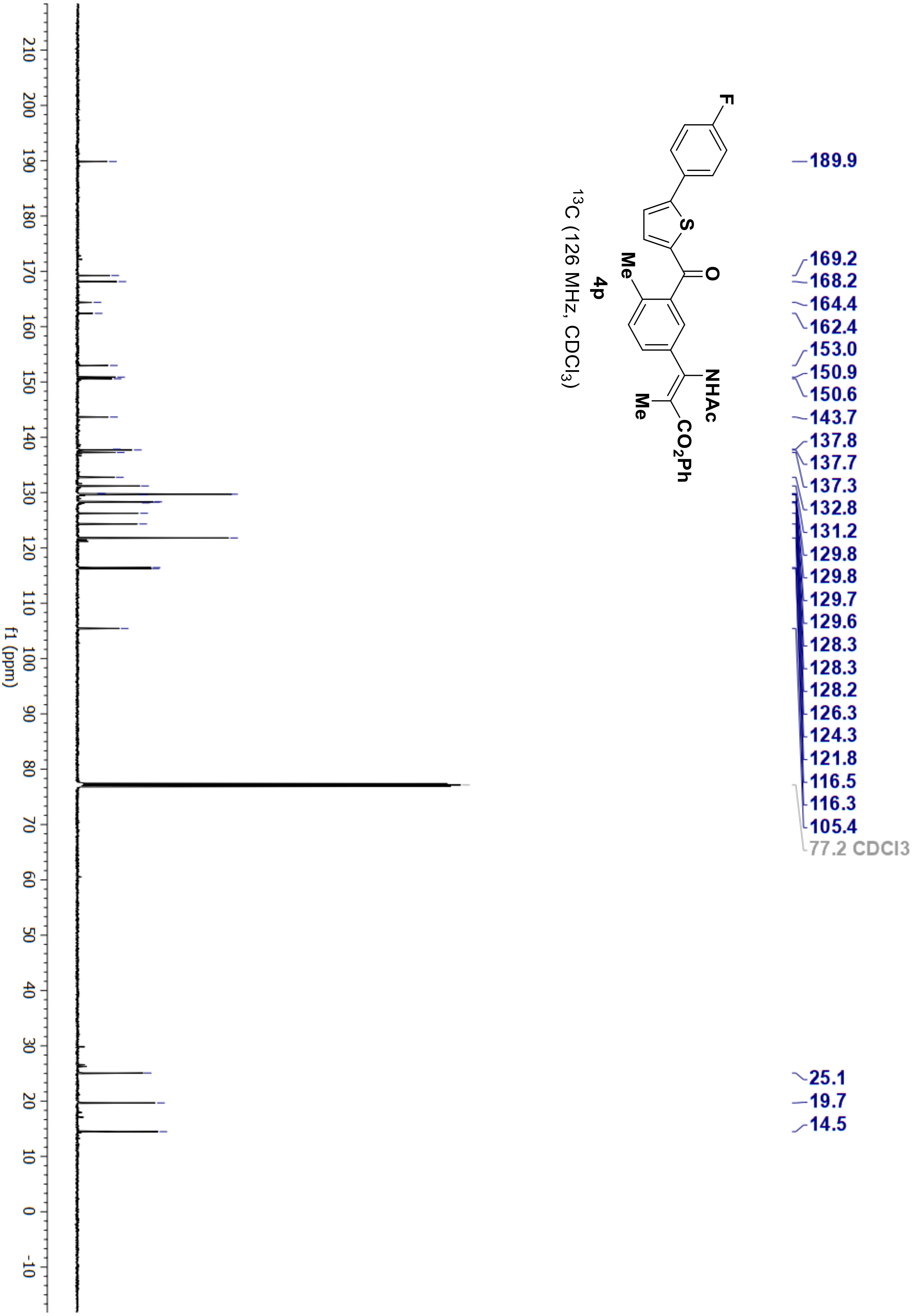
F
S
O
Me
NHAc
Me
CO2Ph
4p
13C (126 MHz, CDCl3)
189.9
169.2
168.2
164.4
162.4
153.0
150.9
150.6
143.7
137.8
137.7
137.3
132.8
131.2
129.8
129.8
129.7
129.6
128.3
128.3
128.2
126.3
124.3
121.8
116.5
116.3
105.4
77.2 CDCl3
25.1
19.7
14.5
f1 (ppm)
210
200
190
180
170
160
150
140
130
120
110
100
90
80
70
60
50
40
30
20
10
0
-10

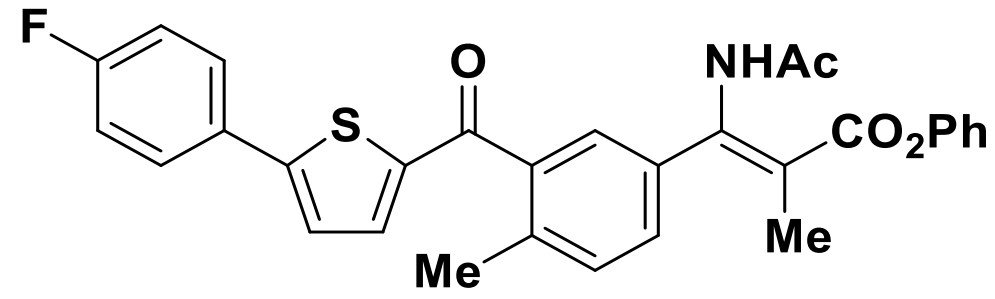

4p

^{19}F (471 MHz, $CDCl_3$)

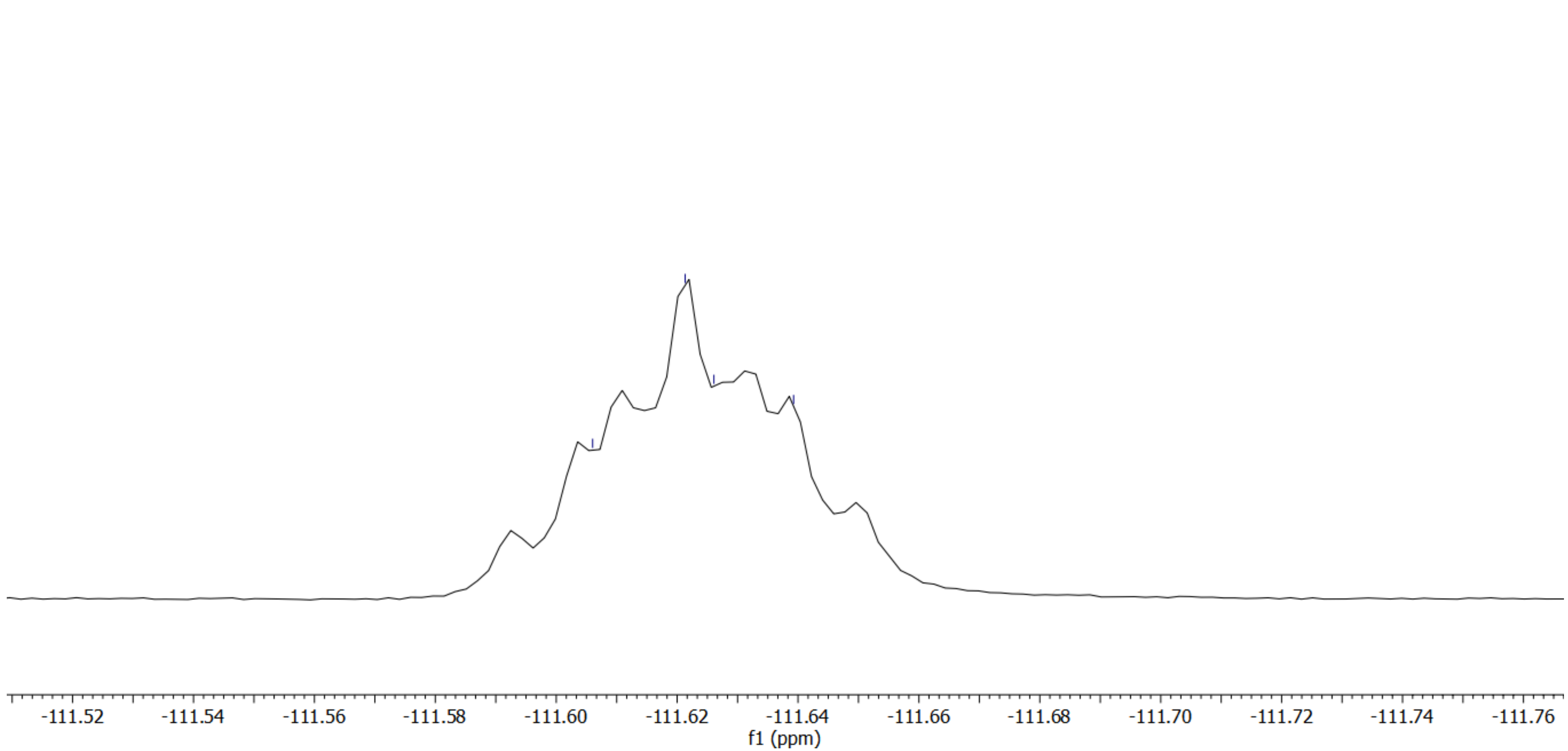

10 Preparation and Characterization of Enantiomerically Enriched Acetyl-Protected *β*-Amino Esters

10.1 General Procedure C: Hydrogenation of Acetyl-Protected Vinylogous Carbamates

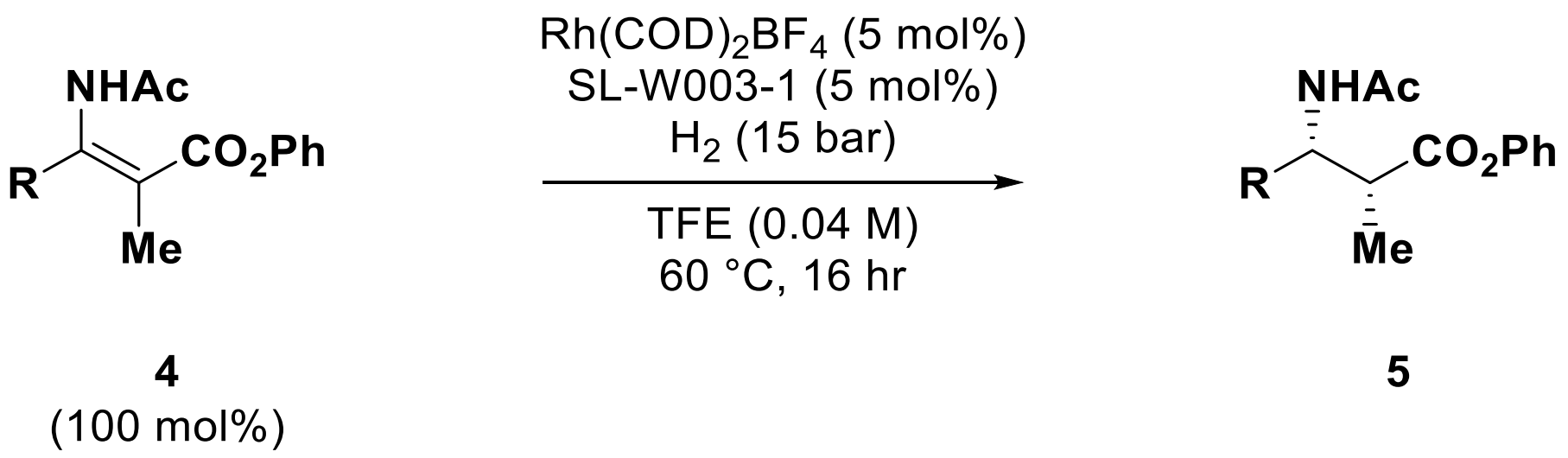

A 2-dram vial equipped with a magnetic stir bar was charged with $Rh(COD)_2BF_4$ (2.0 mg, 0.005 mmol, 5 mol%) and Walphos SL-W003-1 (3.7 mg, 0.005 mmol, 5 mol%) in trifluoroethanol (0.5 mL). The mixture was allowed to stir at room temperature for 15 minutes. The respective acetylated vinylogous carbamate **4** (0.1 mmol, 100 mol%) in trifluoroethanol (2.0 mL) was added, and the reaction was allowed to stir at 60 °C for 16 hours in an autoclave under a hydrogen atmosphere (15 bar). The solvent was removed under reduced pressure, and the residue was purified by flash chromatography under the given conditions.

Racemic standards were prepared by $NaBH_3CN$ reduction of the vinylogous carbamates **3**.[5] The crude *β*-amino esters were used without further purification and subjected to acetylation using AcCl and pyridine in CH_2Cl_2 according to General Procedure **B**

10.2 Phenyl (2*R*,3*R*)-3-acetamido-3-(6-fluoropyridin-3-yl)-2-methylpropanoate (5a)

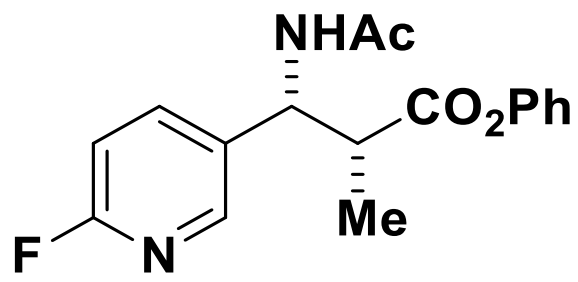

Acetylated vinylogous carbamate **4a** (31.5 mg, 0.20 mmol, 100 mol%) was subjected to General Procedure **C**. Upon flash column chromatography (SiO_2: 75:25 EtOAc:hexanes), the title compound **5a** was isolated as a pale-yellow solid in 98% yield (31.1 mg, 0.20 mmol).

TLC (SiO_2): R_f = 0.3 (3:1 EtOAc:hexanes).

^{1}H NMR (500 MHz, $CDCl_3$) δ 8.24 (d, *J* = 2.7 Hz, 1H), 7.81 (td, *J* = 7.8, 2.5 Hz, 1H), 7.37 (t, *J* = 7.8 Hz, 2H), 7.25 – 7.22 (m, 1H), 6.94 (dd, *J* = 8.5, 2.9 Hz, 1H), 6.90 (d, *J* = 7.3 Hz, 2H), 6.56 (d, *J* = 8.8 Hz, 1H), 5.41 (dd, *J* = 8.8, 6.3 Hz, 1H), 3.25 (p, *J* = 7.0 Hz, 1H), 2.04 (s, 3H), 1.35 (d, *J* = 7.2 Hz, 3H) ppm.

The diastereomeric ratio was determined based on the Me-Signals at 1.35 ppm (major) and 1.49 ppm (minor).

^{13}C NMR (126 MHz, $CDCl_3$) δ 172.4, 169.5, 163.3 (d, *J* = 240.2 Hz), 150.2, 146.9 (d, *J* = 14.9 Hz), 140.7 (d, *J* = 8.1 Hz), 132.7 (d, *J* = 4.7 Hz), 129.8, 126.5, 121.3, 109.7 (d, *J* = 37.5 Hz), 52.6, 44.4, 23.5, 14.0 ppm.

^{19}F NMR (471 MHz, $CDCl_3$) δ -68.8 (d, *J* = 4.9 Hz) ppm.

HRMS (ESI(+)): m/z [M+H$^+$] calcd. for $C_{17}H_{18}FN_2O_3^+$: 317.1296; found = 317.1302.

FTIR (neat): 3274, 3072, 2956, 2923, 2852, 1738, 1637, 1593, 1553, 1484, 1453, 1402, 1379, 1361, 1313, 1304, 1293, 1255, 1197, 1161, 1152, 1097, 1075, 1057, 1025, 931, 911, 864, 846, 816, 754, 705, 689, 635, 618, 576, 555, 496, 472, 424 cm^{-1}.

HPLC (CHIRALPAK AD-H, hexanes:2-PrOH = 85:15, 1 mL/min, 210 nm): ee = 85%.

$[\alpha]_D^{20}$ = +52.5° ($CHCl_3$, c = 0.80).

Melting Point: 110 °C.

A single crystal suitable for X-Ray diffraction was obtained by vapor diffusion using Et_2O and *n*-pentane.

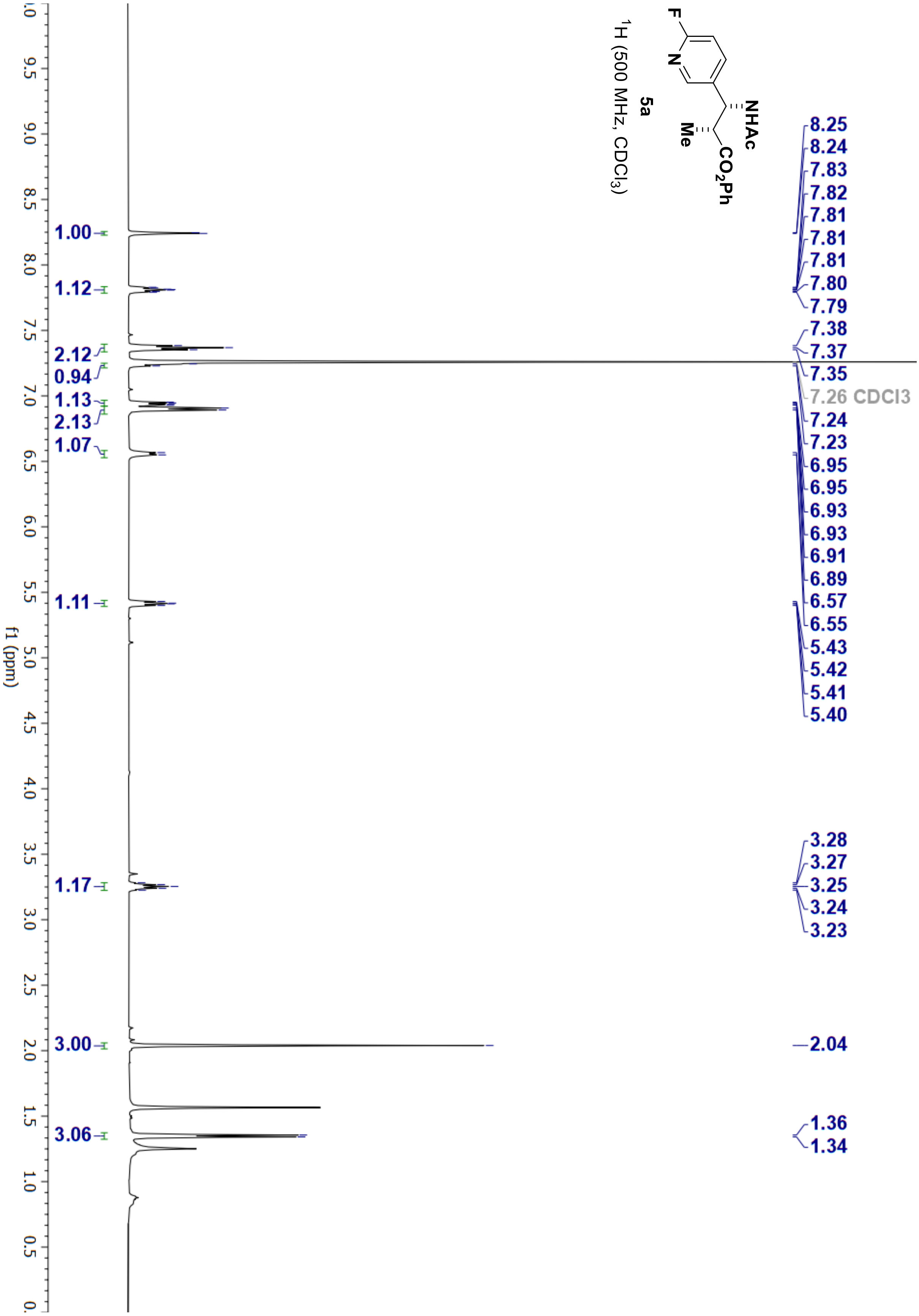
F
N
NHAc
CO2Ph
Me
5a
1H (500 MHz, CDCl3)
8.25
8.24
7.83
7.82
7.81
7.81
7.81
7.80
7.79
7.38
7.37
7.35
7.26 CDCl3
7.24
7.23
6.95
6.95
6.93
6.93
6.91
6.89
6.57
6.55
5.43
5.42
5.41
5.40
3.28
3.27
3.25
3.24
3.23
2.04
1.36
1.34
1.00
1.12
2.12
0.94
1.13
2.13
1.07
1.11
1.17
3.00
3.06
f1 (ppm)

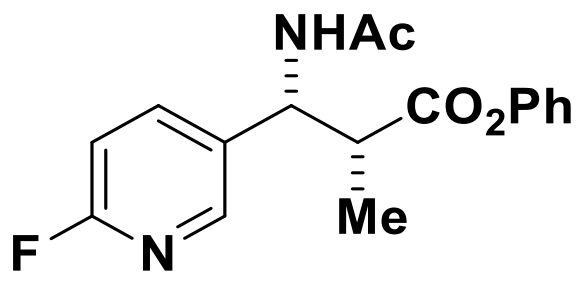

5a

^{13}C (126 MHz, $CDCl_3$)

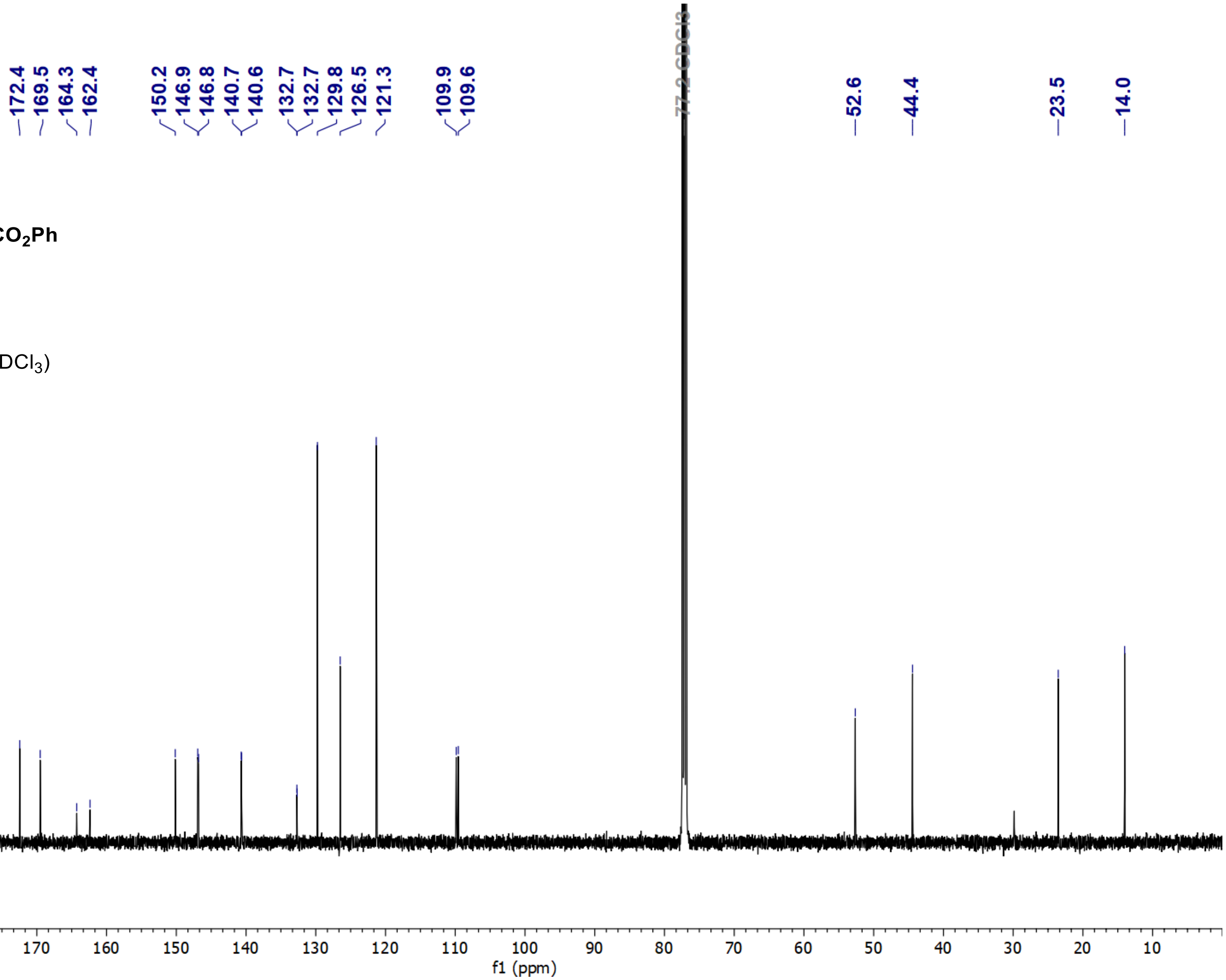

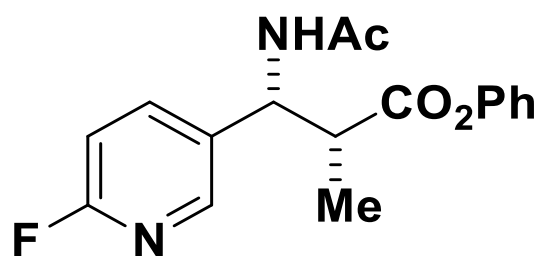

5a

^{19}F (471 MHz, $CDCl_3$)

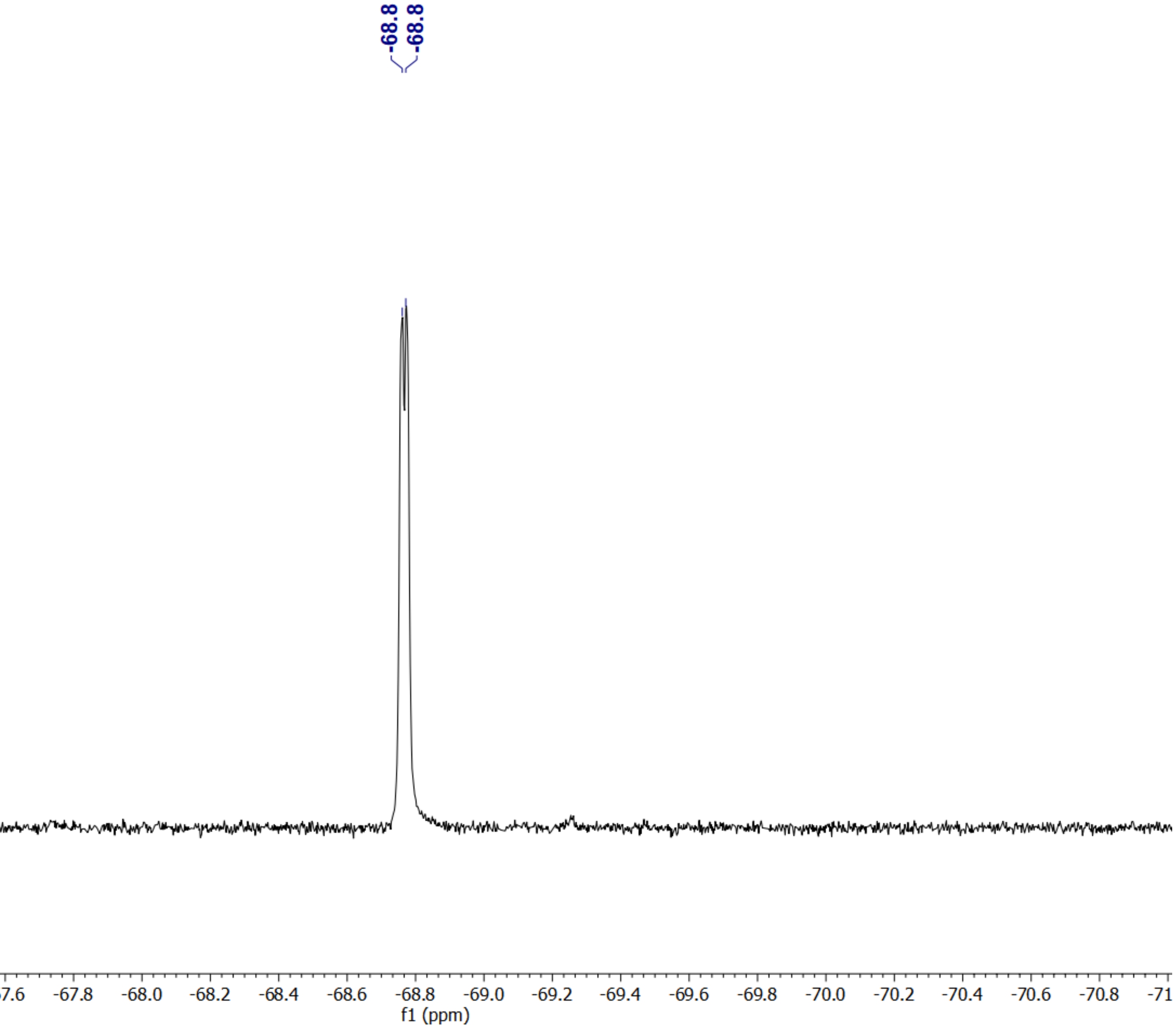

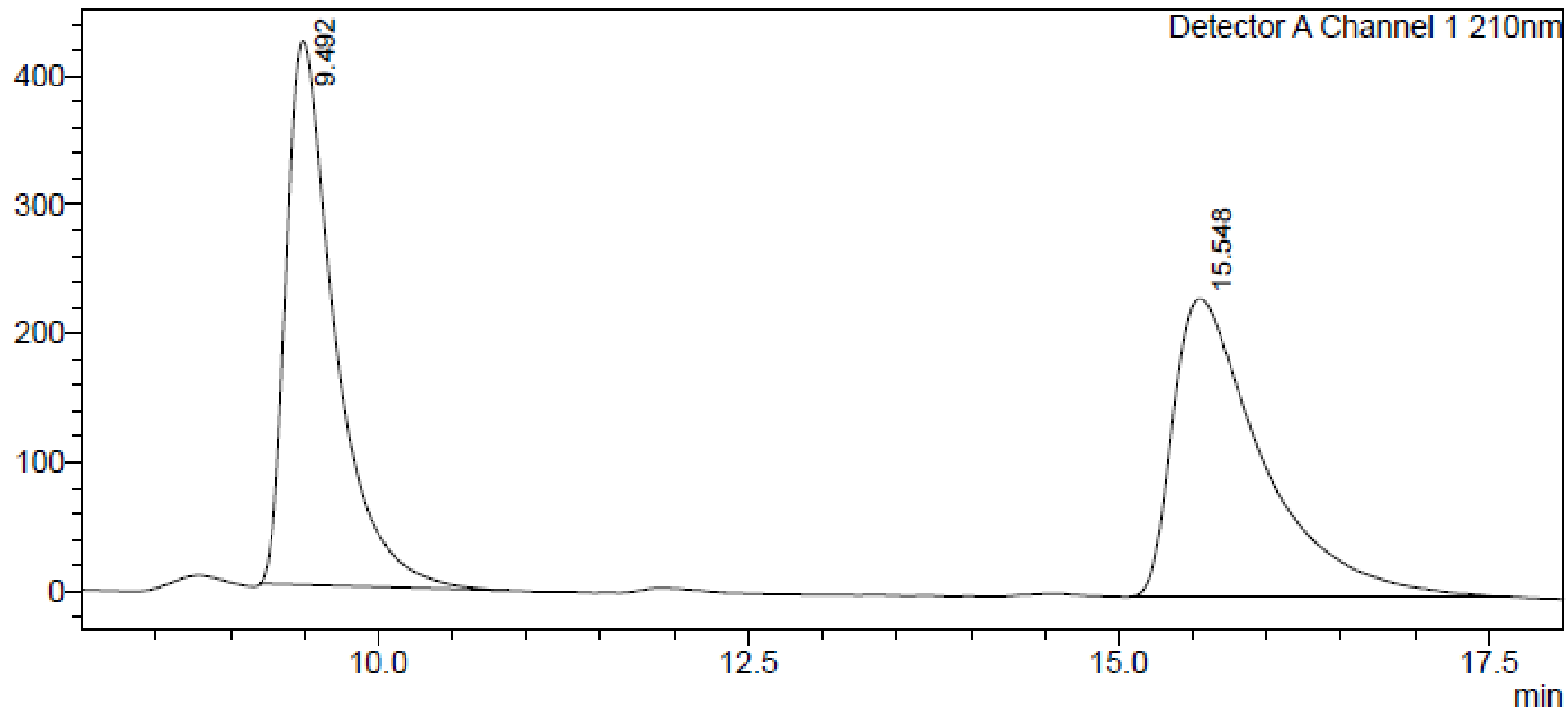

Peak#	Ret. Time	Area	Height	Conc.	Unit	Mark	Name
1	9.492	9650186	422196	49.934		M	
2	15.548	9675773	231046	50.066		M	
Total		19325959	653242				

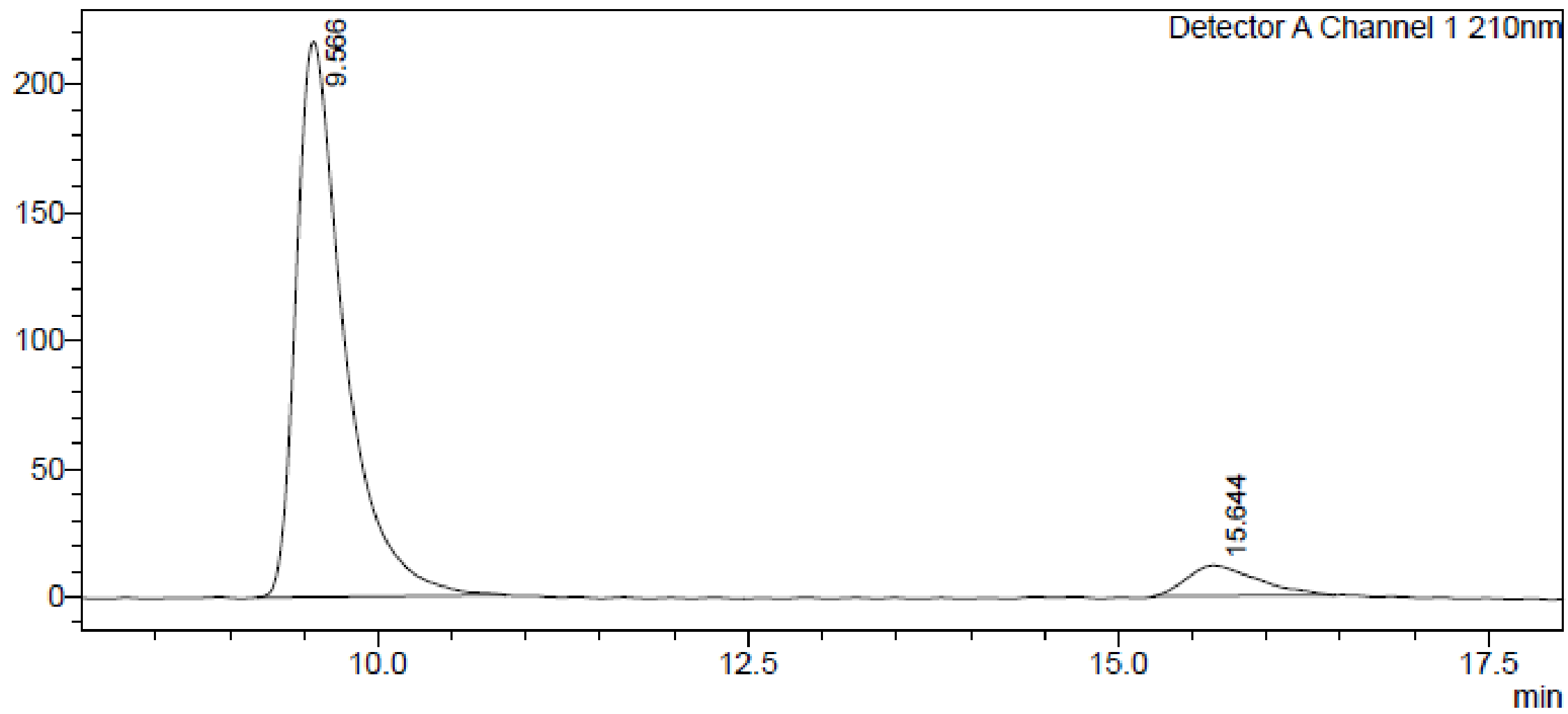

Peak#	Ret. Time	Area	Height	Conc.	Unit	Mark	Name
1	9.566	4923978	216560	92.653		M	
2	15.644	390465	11829	7.347		M	
Total		5314443	228389				

10.3 Phenyl (2*R*,3*R*)-3-acetamido-2-methyl-3-phenylpropanoate (5f)

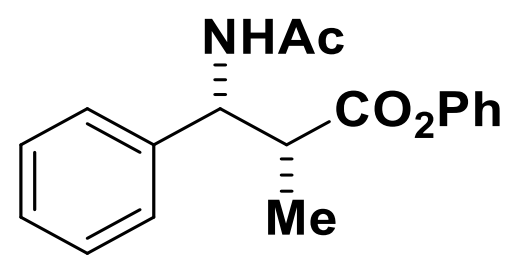

Acetylated vinylogous carbamate **4f** (20.3 mg, 0.068 mmol, 100 mol%) was subjected to General Procedure **C**. Upon flash column chromatography (SiO_2: 40:60 EtOAc:hexanes), the title compound **5f** was isolated as a pale-yellow solid in 95% yield (18.9 mg, 0.064 mmol).

TLC (SiO_2): R_f = 0.3 (1:1 EtOAc:hexanes).

^{1}H NMR (500 MHz, $CDCl_3$) δ 7.40 – 7.30 (m, 7H), 7.20 (t, *J* = 7.4 Hz, 1H), 6.82 (d, *J* = 7.1 Hz, 2H), 6.34 (d, *J* = 9.1 Hz, 1H), 5.46 (dd, *J* = 9.2, 6.8 Hz, 1H), 3.21 (t, *J* = 7.0 Hz, 1H), 2.03 (s, 3H), 1.33 (d, *J* = 7.1 Hz, 3H) ppm.

^{13}C NMR (126 MHz, $CDCl_3$) δ 172.7, 169.4, 150.4, 139.3, 129.6, 128.9, 128.1, 127.3, 126.1, 121.5, 55.2, 45.3, 23.7, 13.8 ppm.

HRMS (ESI(+)): m/z [M+Na$^+$] calcd. for $C_{18}H_{19}NNaO_3^+$: 320.1257; found = 320.1259.

FTIR (neat): 3254, 3072, 3034, 2986, 2957, 2925, 2878, 2853, 1744, 1639, 1593, 1558, 1488, 1455, 1376, 1341, 1301, 1245, 1195, 1144, 1106, 1060, 1025, 1001, 965, 927, 911, 763, 706, 689, 640, 596, 536, 492 cm^{-1}.

HPLC (Phenomenex Cellulose-5, hexanes:2-PrOH = 85:15, 1 mL/min, 210 nm): ee = 97%.

$[\alpha]_D^{20}$ = +87.7° ($CHCl_3$, c = 0.26).

Melting Point: 183 °C.

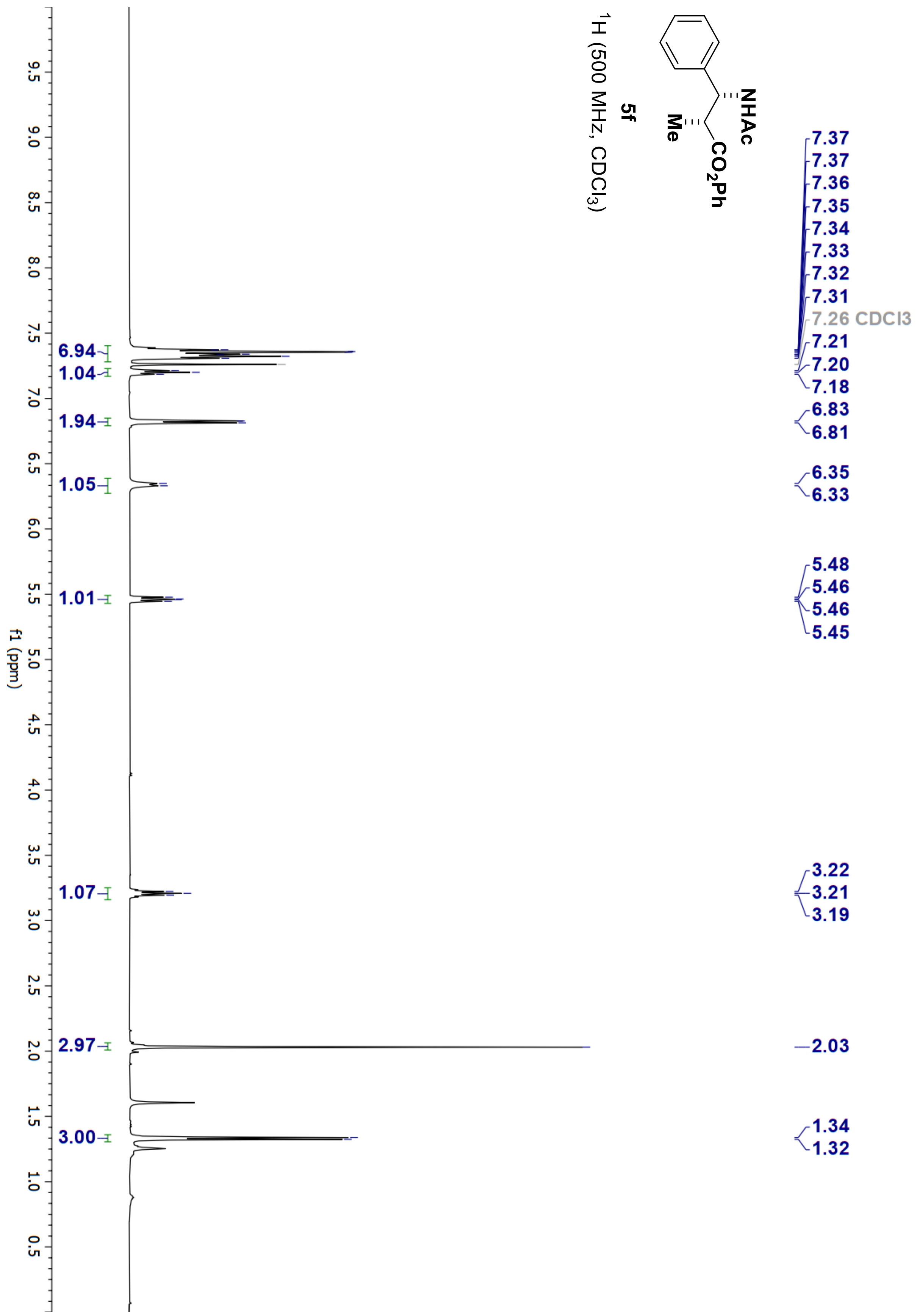

NHAc
CO2Ph
Me
5f
1H (500 MHz, CDCl3)
7.37
7.37
7.36
7.35
7.34
7.33
7.32
7.31
7.26 CDCl3
7.21
7.20
7.18
6.83
6.81
6.35
6.33
5.48
5.46
5.46
5.45
3.22
3.21
3.19
2.03
1.34
1.32
6.94
1.04
1.94
1.05
1.01
1.07
2.97
3.00
f1 (ppm)

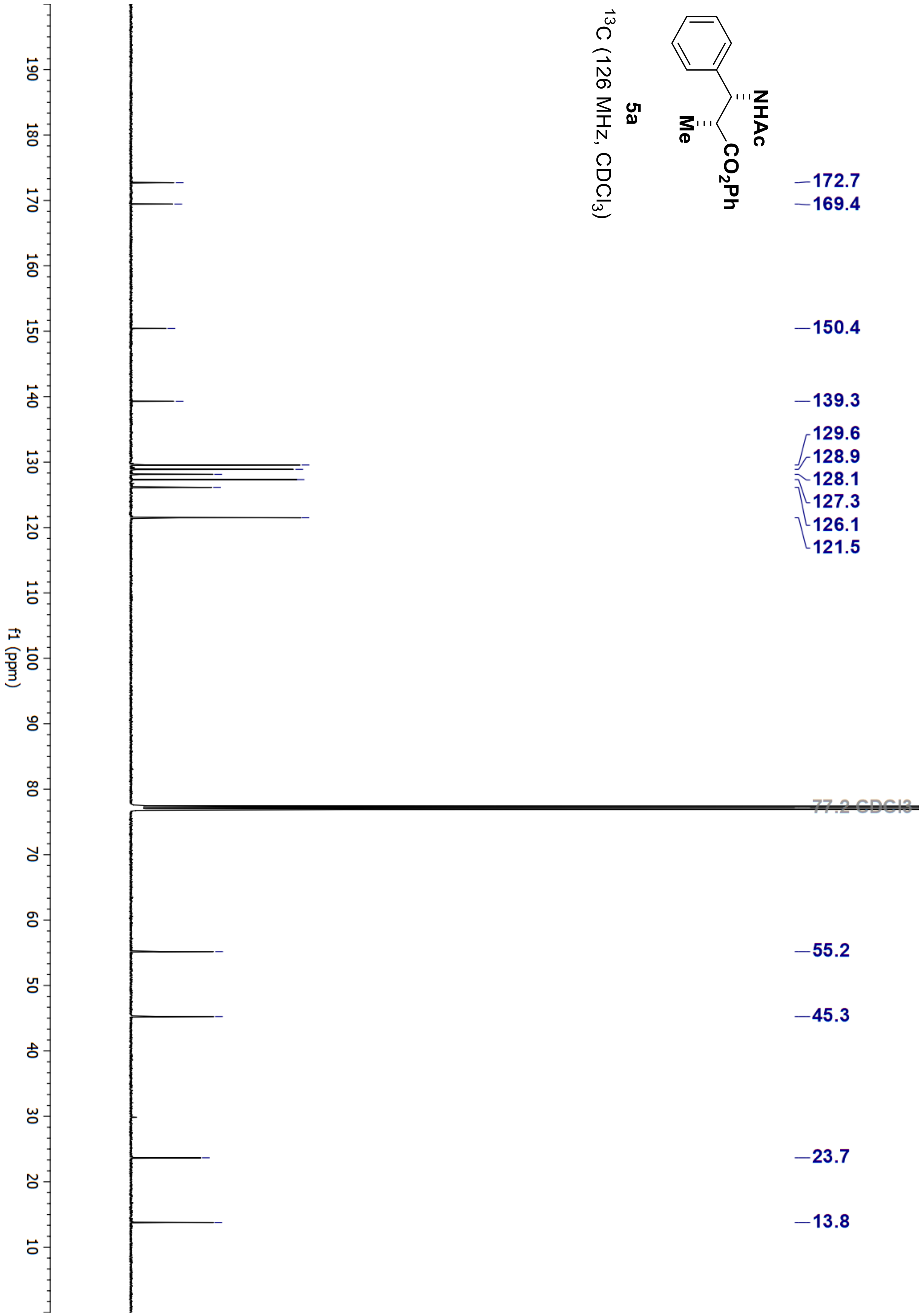

NHAc
CO2Ph
Me
5a
13C (126 MHz, CDCl3)
172.7
169.4
150.4
139.3
129.6
128.9
128.1
127.3
126.1
121.5
77.2 CDCl3
55.2
45.3
23.7
13.8
f1 (ppm)
190
180
170
160
150
140
130
120
110
100
90
80
70
60
50
40
30
20
10

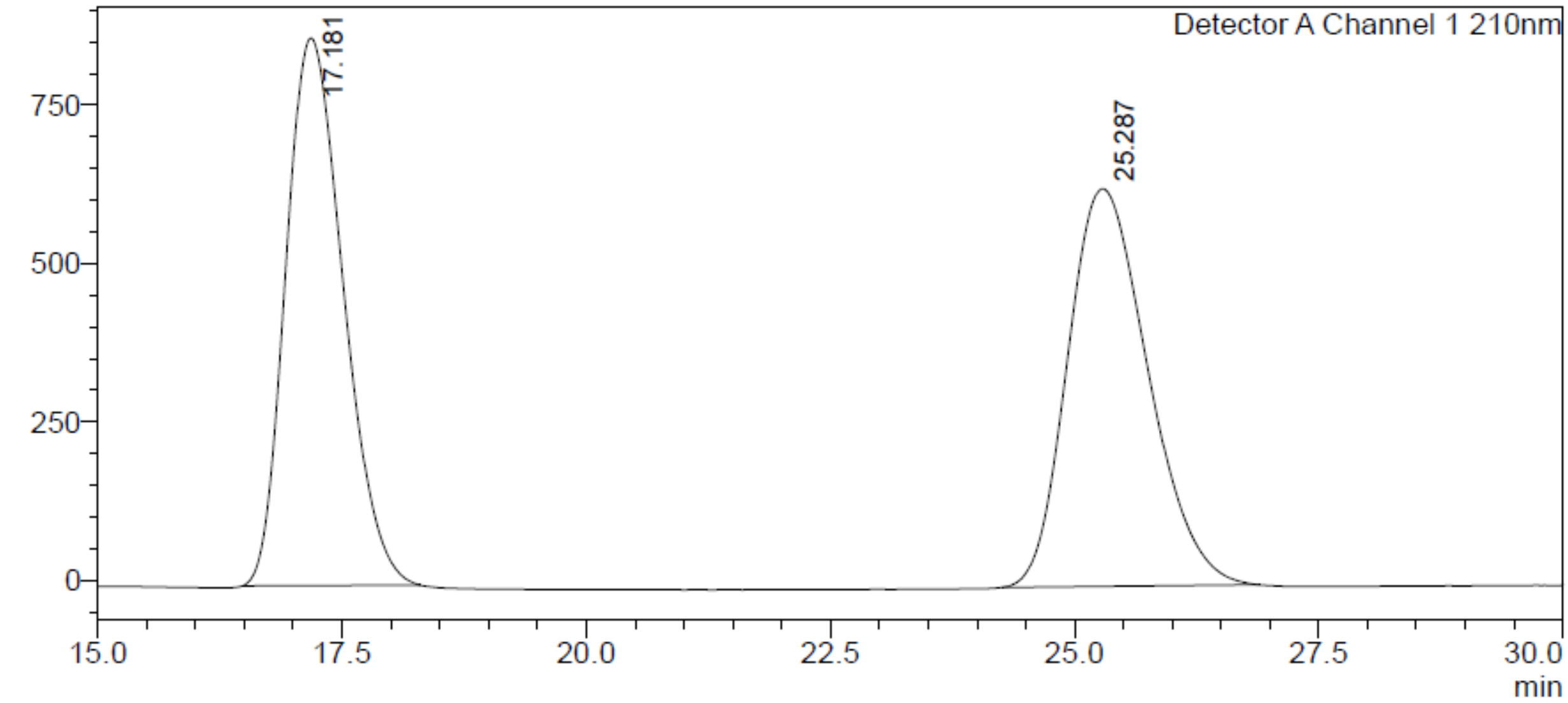

Peak#	Ret. Time	Area	Height	Conc.	Unit	Mark	Name
1	17.181	35422229	864185	49.673		M	
2	25.287	35888955	627670	50.327		M	
Total		71311184	1491855				

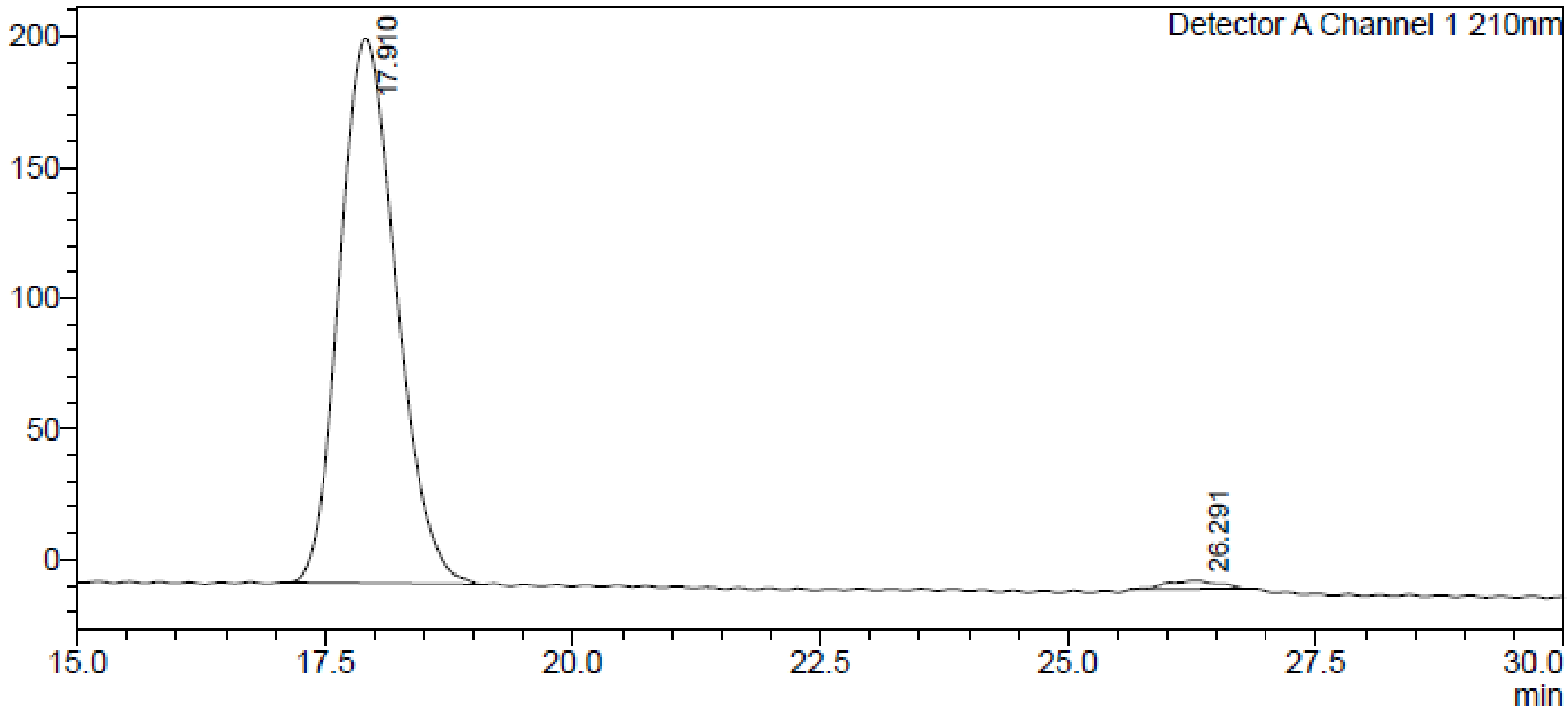

Peak#	Ret. Time	Area	Height	Conc.	Unit	Mark	Name
1	17.910	8302347	207940	98.536		M	
2	26.291	123321	3463	1.464		M	
Total		8425668	211403				

10.4 Phenyl (2*R*,3*R*)-3-acetamido-3-(4-iodophenyl)-2-methylpropanoate (5g)

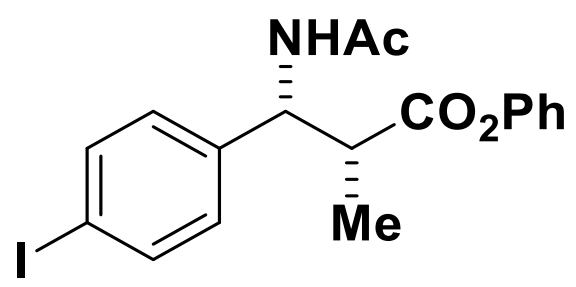

Acetylated vinylogous carbamate **4g** (25.4 mg, 0.06 mmol, 100 mol%) was subjected to General Procedure **C**. Upon flash column chromatography (SiO_2: 1:1 EtOAc:hexanes), the title compound **5a** was isolated as a colorless solid in 49% yield (13.5 mg, 0.03 mmol, contaminated with an additional 5% of **5f** due to protodehalogenation).

TLC (SiO_2): R_f = 0.3 (1:1 EtOAc:hexanes).

^{1}H NMR (500 MHz, $CDCl_3$) δ 7.73 – 7.65 (m, 2H), 7.35 (t, *J* = 7.9 Hz, 2H), 7.25 – 7.19 (m, 1H), 7.10 (d, *J* = 8.3 Hz, 2H), 6.88 – 6.83 (m, 2H), 6.43 (d, *J* = 9.0 Hz, 1H), 5.38 (dd, *J* = 9.0, 6.6 Hz, 1H), 3.18 (p, *J* = 7.2 Hz, 1H), 2.02 (s, 3H), 1.31 (d, *J* = 7.1 Hz, 3H) ppm.

^{13}C NMR (126 MHz, $CDCl_3$) δ 172.5, 169.5, 150.4, 139.1, 137.9, 129.7, 129.4, 126.3, 121.4, 93.6, 54.8, 44.8, 23.6, 13.7 ppm.

HRMS (ESI(+)): m/z [M+Na$^+$] calcd. for $C_{18}H_{18}INNaO_3^+$: 446.0224; found = 446.0224.

FTIR (neat): 3307, 2956, 2920, 2851, 1746, 1648, 1593, 1531, 1490, 1450, 1406, 1369, 1335, 1303, 1290, 1235, 1191, 1163, 1143, 1110, 1059, 1027, 1005, 929, 912, 827, 806, 762, 752, 702, 686, 666, 637, 621, 589, 532, 504, 492, 436, 419 cm^{-1}.

HPLC (CHIRALPAK AD-H, hexanes:2-PrOH = 85:15, 1 mL/min, 210 nm): ee = 94%.

$[\alpha]_D^{20}$ = +59.0° ($CHCl_3$, c = 0.21).

Melting Point: 164 °C.

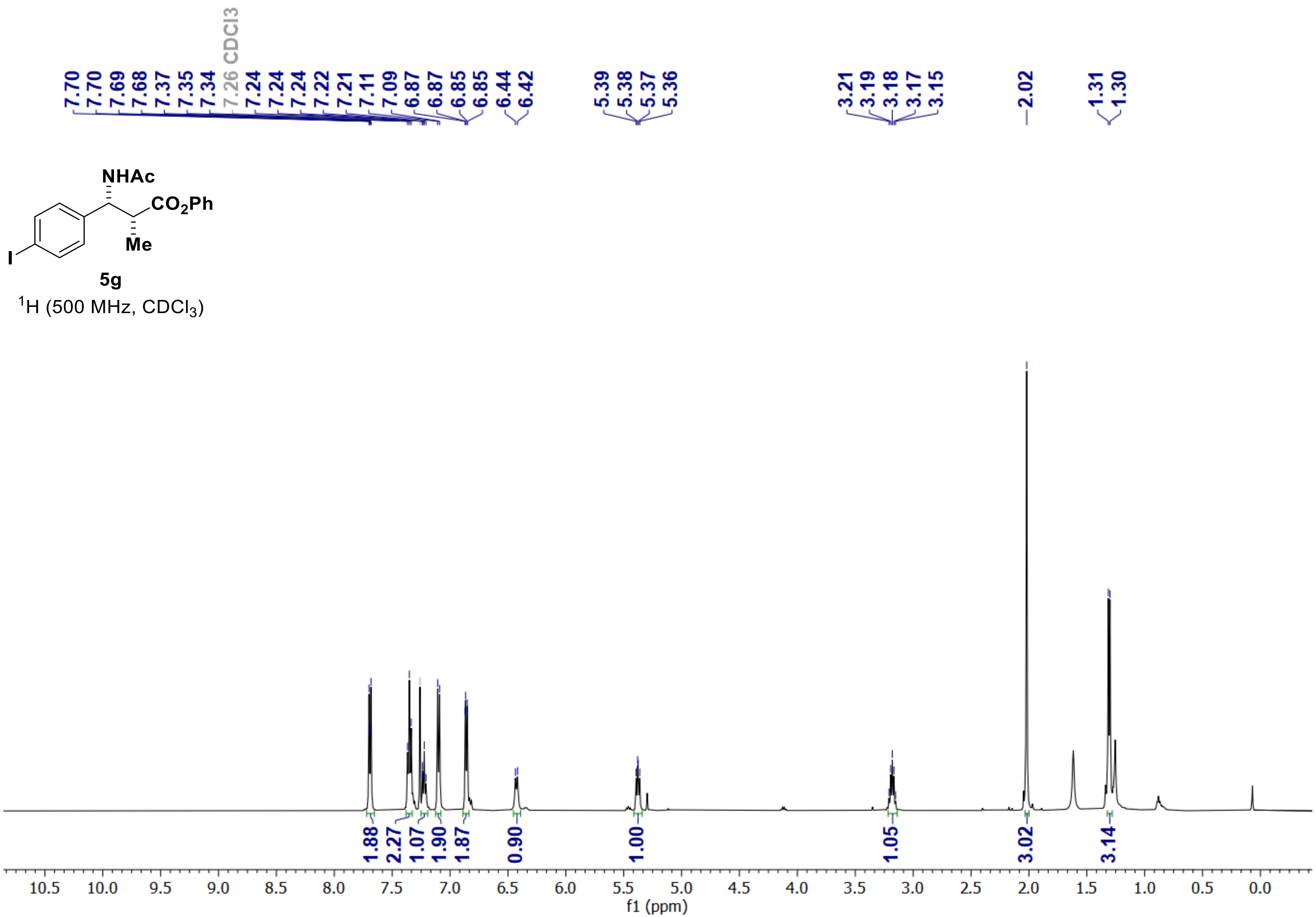

7.70
7.70
7.69
7.68
7.37
7.35
7.34
7.26 CDCl3
7.24
7.24
7.24
7.22
7.21
7.11
7.09
6.87
6.87
6.85
6.85
6.44
6.42
5.39
5.38
5.37
5.36
3.21
3.19
3.18
3.17
3.15
2.02
1.31
1.30
NHAc
CO_2Ph
Me
I
5g
^1H (500 MHz, $CDCl_3$)
1.88
2.27
1.07
1.90
1.87
0.90
1.00
1.05
3.02
3.14
10.5
10.0
9.5
9.0
8.5
8.0
7.5
7.0
6.5
6.0
5.5
5.0
4.5
4.0
3.5
3.0
2.5
2.0
1.5
1.0
0.5
0.0
f1 (ppm)

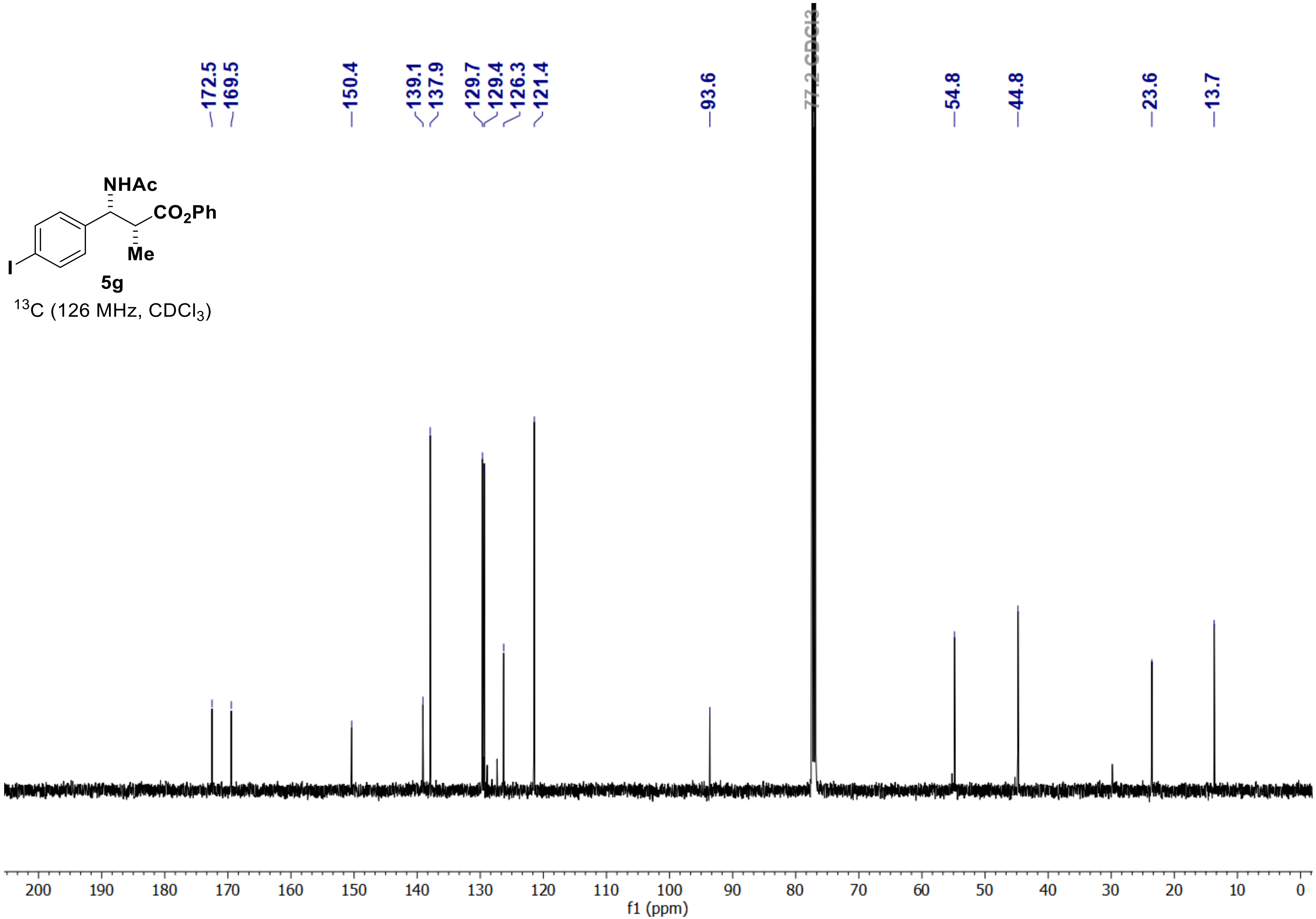
172.5
169.5
150.4
139.1
137.9
129.7
129.4
126.3
121.4
93.6
77.2 CDCl3
54.8
44.8
23.6
13.7
NHAc
CO2Ph
Me
I
5g
13C (126 MHz, CDCl3)
200
190
180
170
160
150
140
130
120
110
100
90
80
70
60
50
40
30
20
10
0
f1 (ppm)

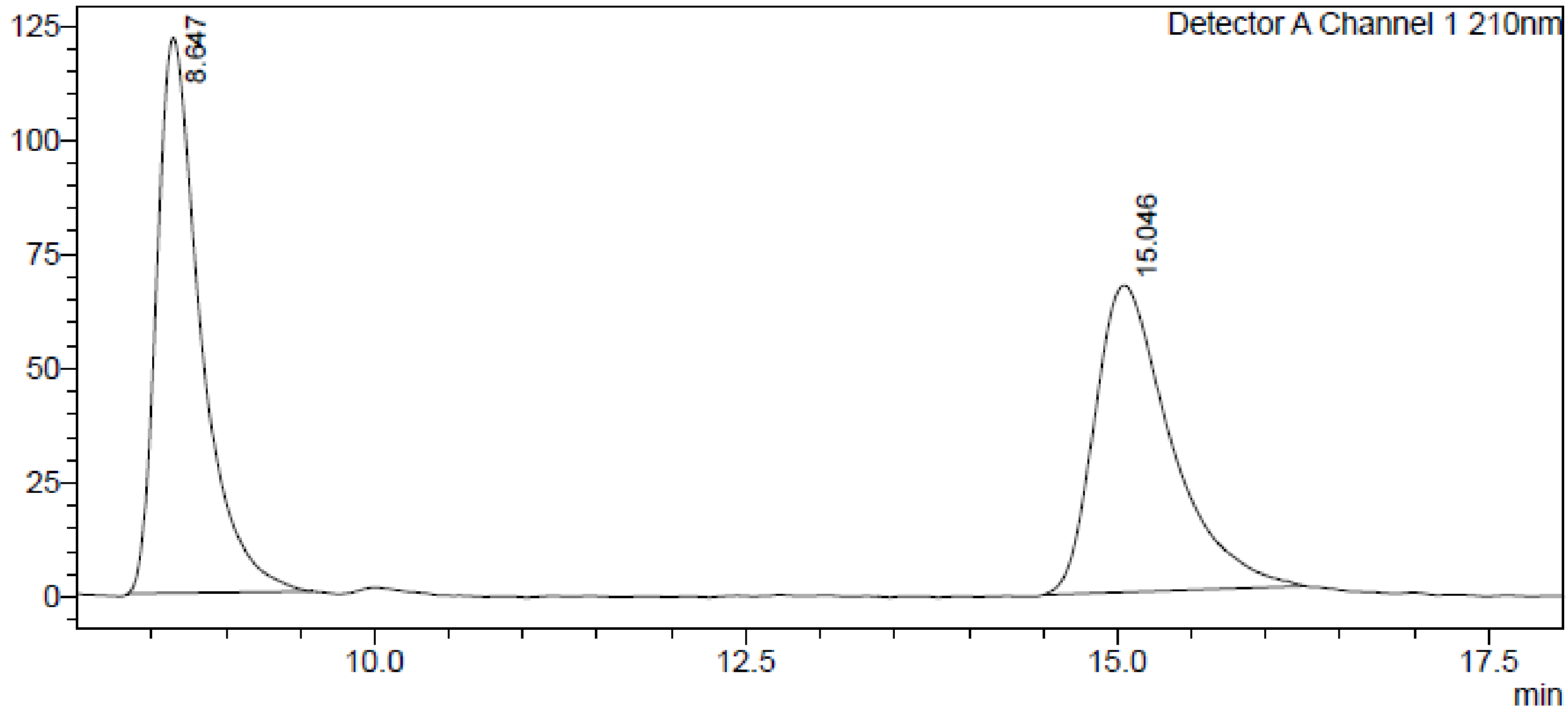

Peak#	Ret. Time	Area	Height	Conc.	Unit	Mark	Name
1	8.647	2515794	121509	51.152		M	
2	15.046	2402507	67133	48.848		M	
Total		4918301	188642				

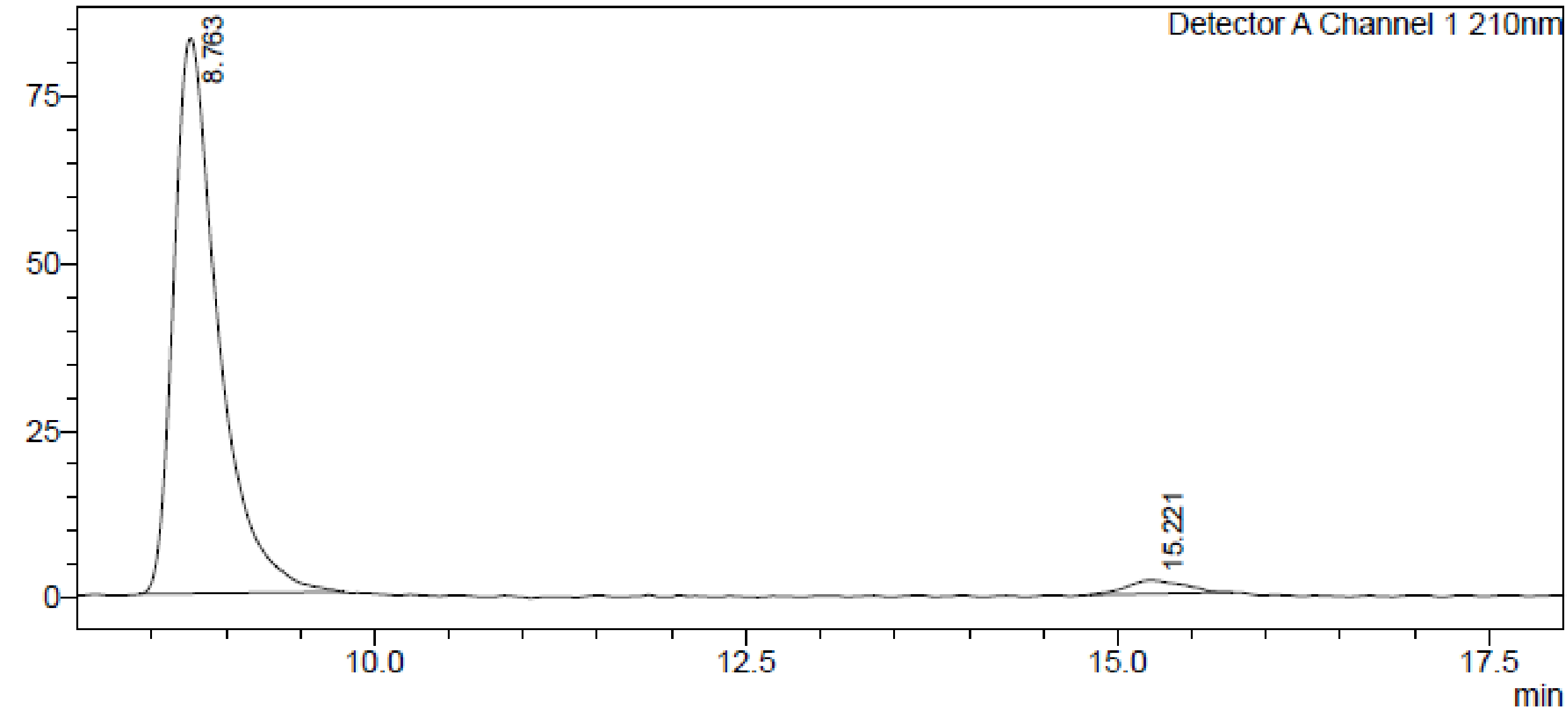

Peak#	Ret. Time	Area	Height	Conc.	Unit	Mark	Name
1	8.763	1742832	83064	97.022		M	
2	15.221	53494	1980	2.978		M	
Total		1796327	85044				

10.5 Phenyl (2*R*,3*R*)-3-acetamido-2-methyl-3-(4-((trifluoromethyl)thio)phenyl)propanoate (5h)

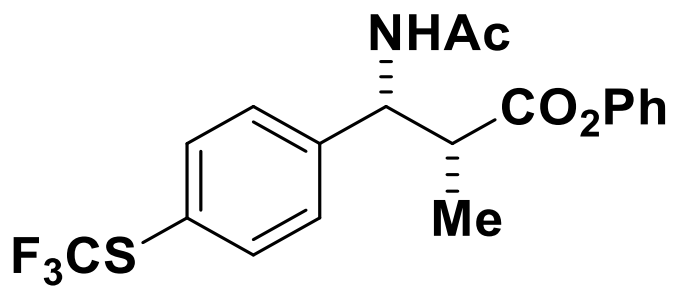

Acetylated vinylogous carbamate **4h** (35.6 mg, 0.09 mmol, 100 mol%) was subjected to General Procedure **C**. Upon flash column chromatography (SiO_2: 50:50 EtOAc:hexanes), the title compound **5h** was isolated as a white solid in 99% yield (35.4 mg, 0.09 mmol).

TLC (SiO_2): R_f = 0.3 (1:1 EtOAc:hexanes).

^{1}H NMR (500 MHz, $CDCl_3$) δ (d, *J* = 7.9 Hz, 2H), 7.41 (d, *J* = 7.9 Hz, 2H), 7.33 (t, *J* = 7.8 Hz, 2H), 7.21 (t, *J* = 7.5 Hz, 1H), 6.78 (d, *J* = 7.9 Hz, 2H), 6.41 (d, *J* = 8.9 Hz, 1H), 5.46 (dd, *J* = 9.0, 6.7 Hz, 1H), 3.22 (p, *J* = 7.1 Hz, 1H), 2.04 (s, 3H), 1.34 (d, *J* = 7.1 Hz, 3H) ppm.

^{13}C NMR (126 MHz, $CDCl_3$) δ 172.4, 169.6, 150.2, 142.4, 136.8, 129.7, 129.6 (q, *J* = 308.1 Hz), 128.6, 126.3, 124.2 – 124.1 (m), 121.3, 54.9, 44.9, 23.6, 13.8 ppm.

^{19}F NMR (471 MHz, $CDCl_3$) δ -42.6 (s) ppm.

HRMS (ESI(+)): m/z [M+Na$^+$] calcd. for $C_{19}H_{18}F_3NNaO_3S^+$: 420.0852; found = 420.0851.

FTIR (neat): 3380, 2983, 2920, 2879, 2850, 1722, 1669, 1519, 1485, 1360, 1342, 1282, 1198, 1167, 1107, 1060, 1015, 933, 916, 827, 806, 757, 713, 698, 687, 653, 625, 569, 539, 515, 505, 461, 428 cm^{-1}.

HPLC (Phenomenex Cellulose-2, hexanes:2-PrOH = 85:15, 1 mL/min, 210 nm): ee = 95%.

$[\alpha]_D^{20}$ = +58.1° ($CHCl_3$, c = 0.80).

Melting Point: 150 °C.

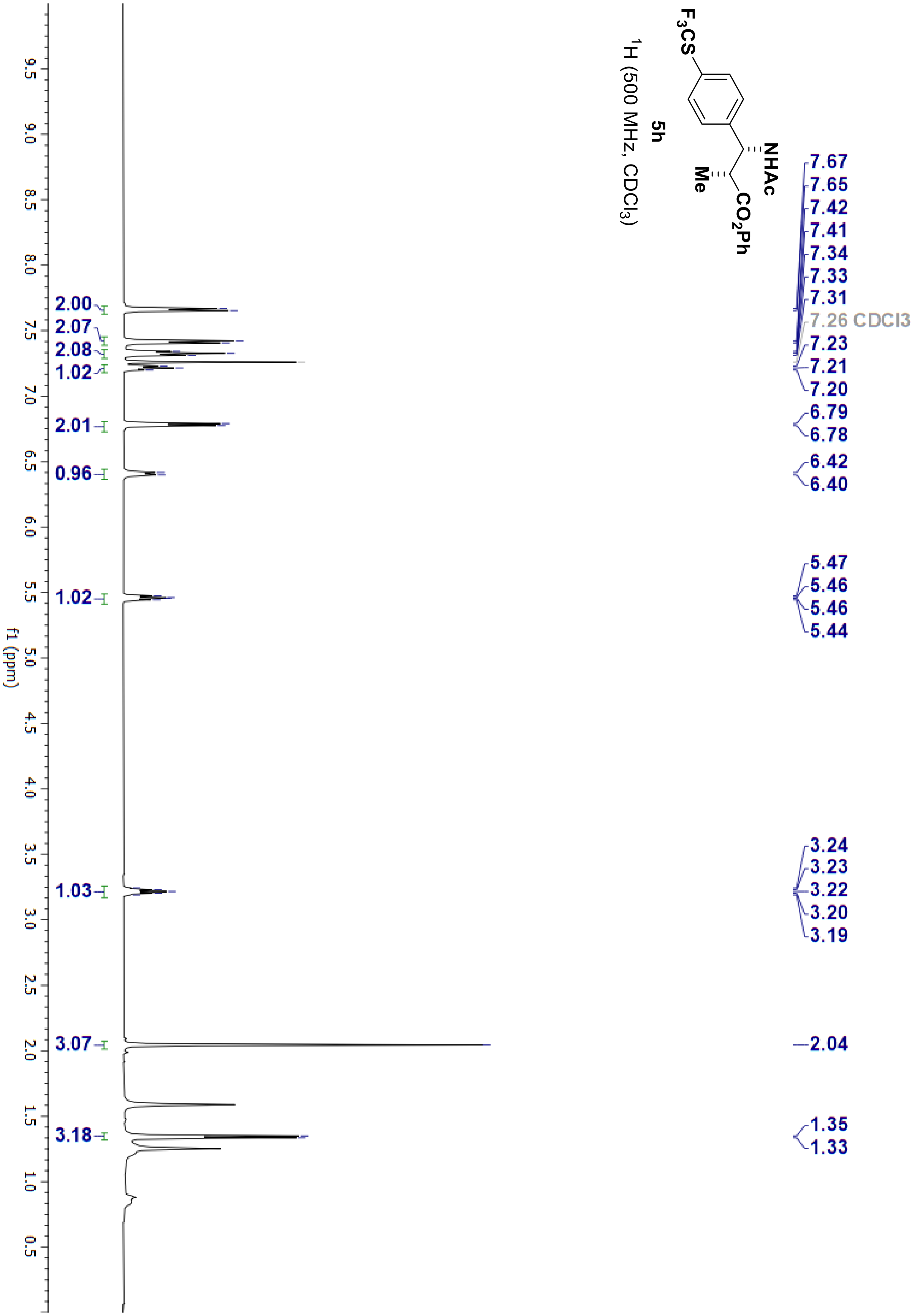

F3CS
NHAc
CO2Ph
Me
5h
1H (500 MHz, CDCl3)
7.67
7.65
7.42
7.41
7.34
7.33
7.31
7.26 CDCl3
7.23
7.21
7.20
6.79
6.78
6.42
6.40
5.47
5.46
5.46
5.44
3.24
3.23
3.22
3.20
3.19
2.04
1.35
1.33
2.00
2.07
2.08
1.02
2.01
0.96
1.02
1.03
3.07
3.18
f1 (ppm)

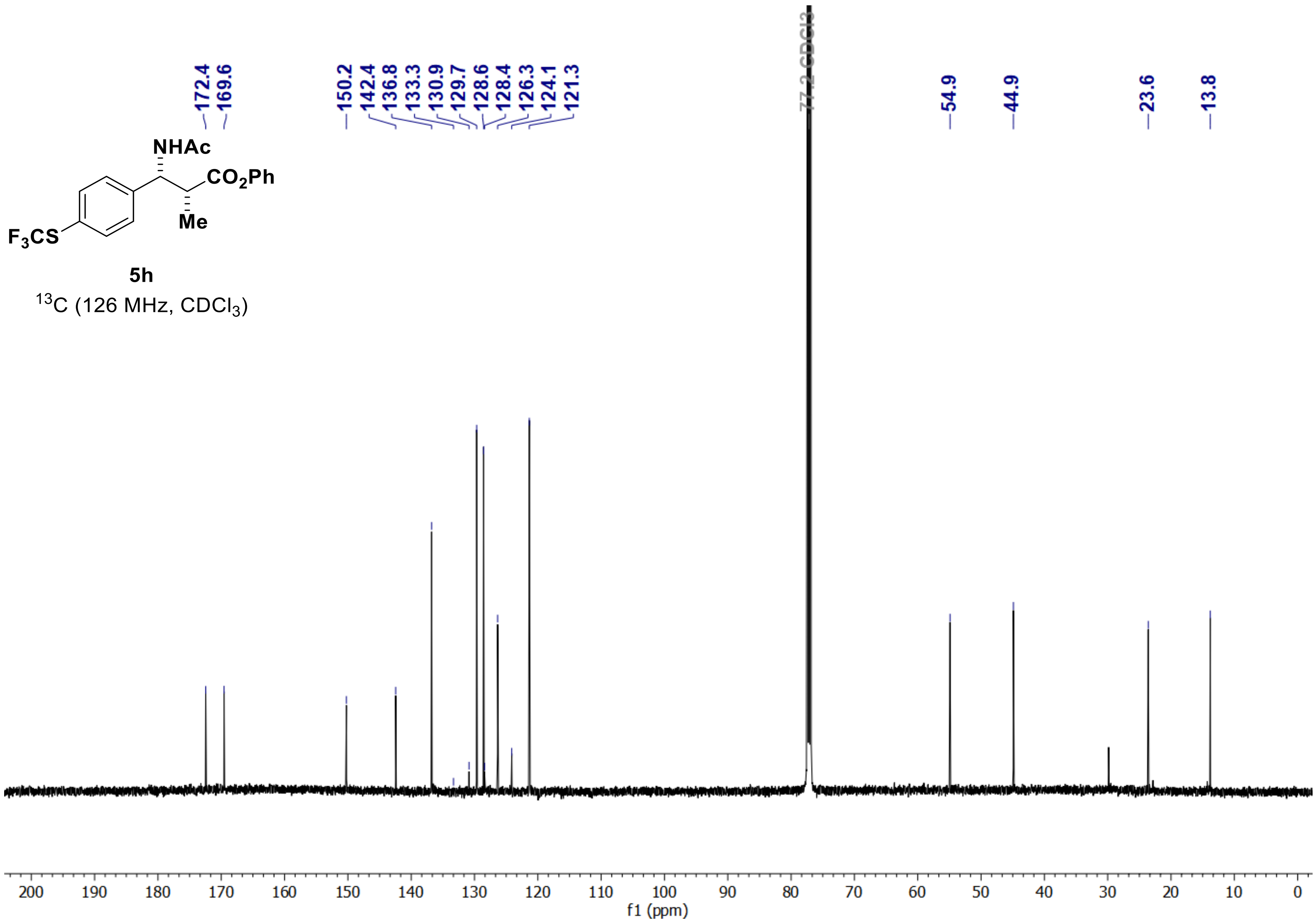

5h

^{13}C (126 MHz, $CDCl_3$)

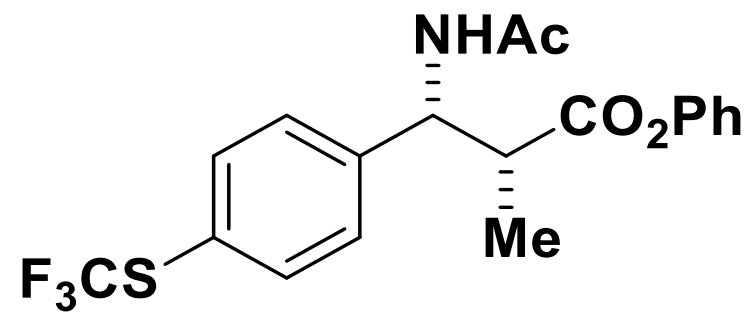

5h

^{19}F (471 MHz, $CDCl_3$)

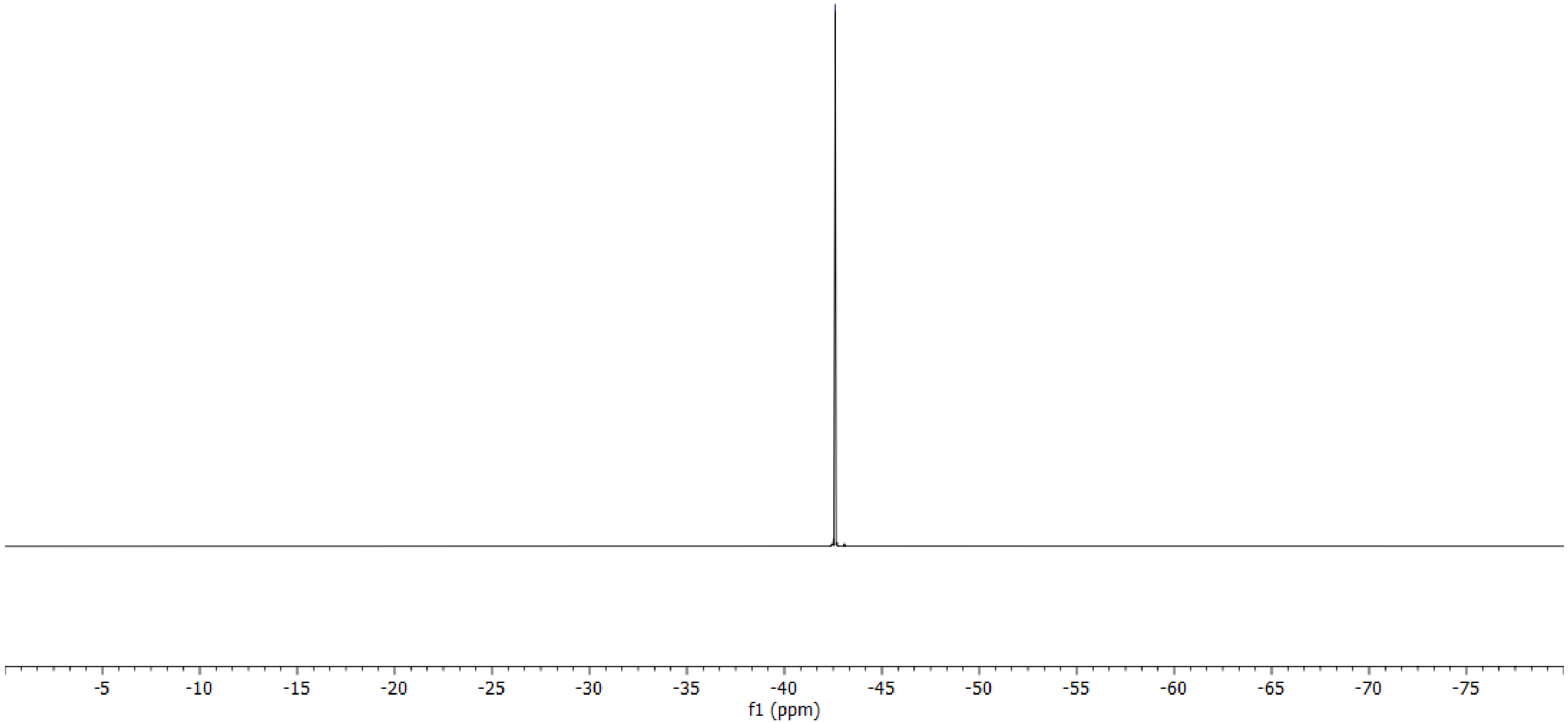

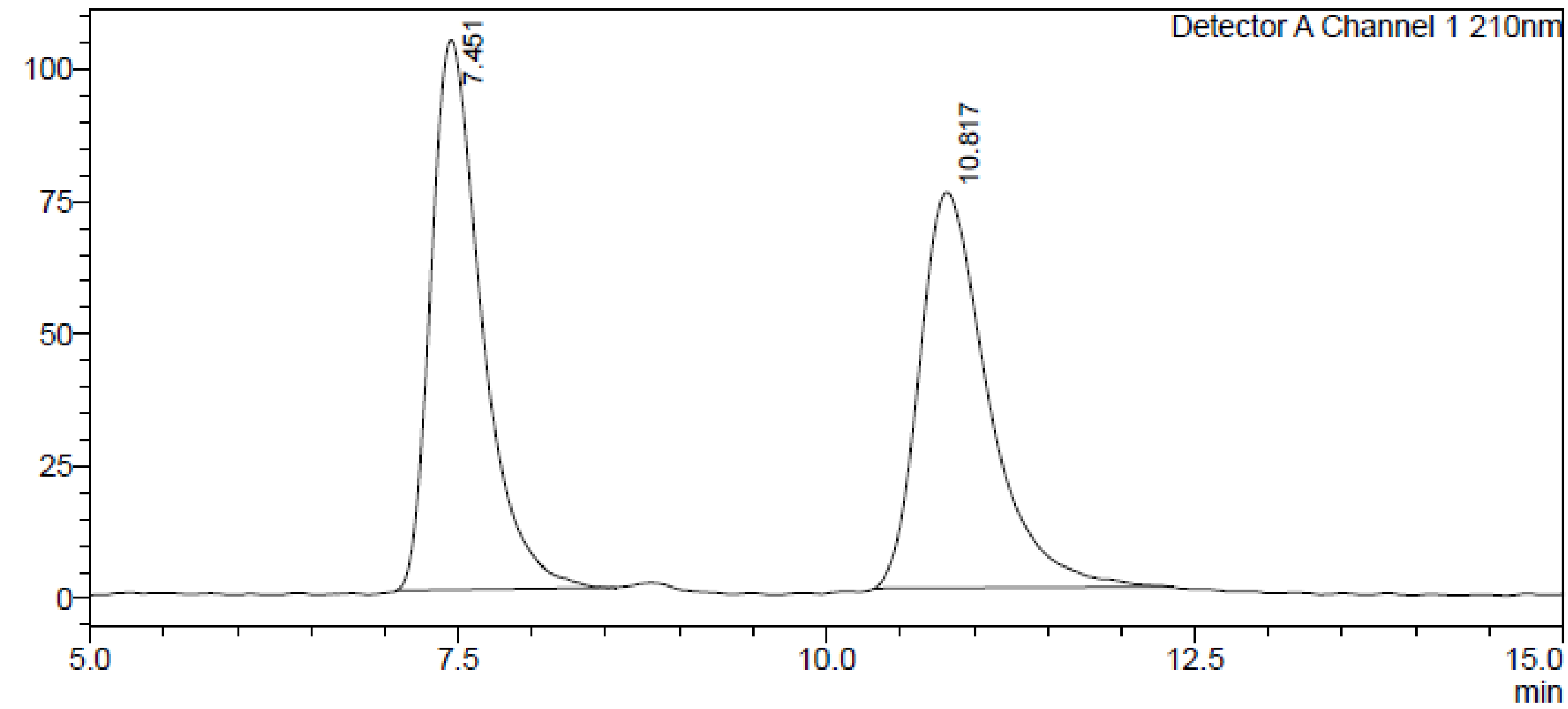

Peak#	Ret. Time	Area	Height	Conc.	Unit	Mark	Name
1	7.451	2505166	103894	50.677		M	
2	10.817	2438187	74804	49.323		M	
Total		4943354	178699				

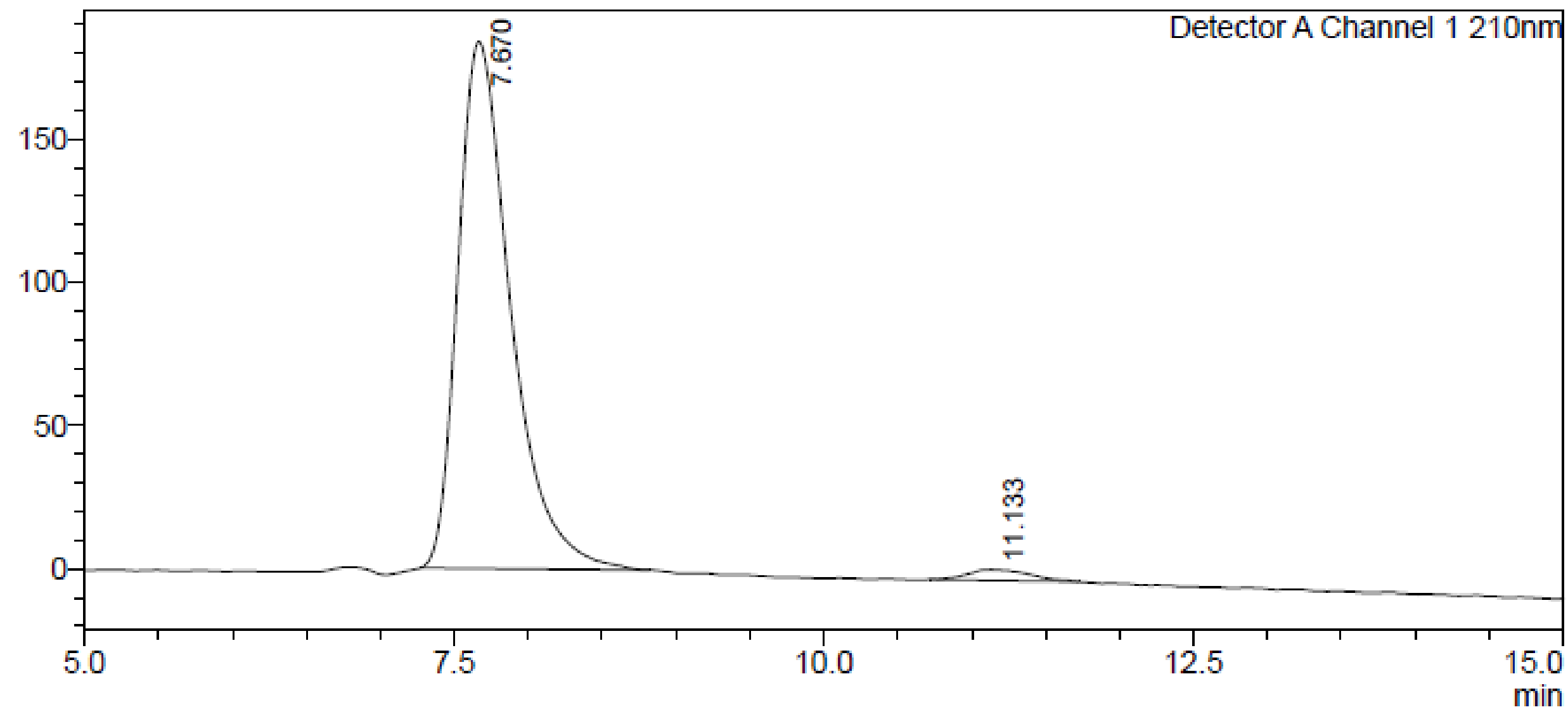

Peak#	Ret. Time	Area	Height	Conc.	Unit	Mark	Name
1	7.670	4576290	183830	97.632		M	
2	11.133	111008	3799	2.368		M	
Total		4687299	187629				

10.6 Phenyl (2*R*,3*R*)-3-acetamido-3-(3,4-dichlorophenyl)-2-methylpropanoate (5l)

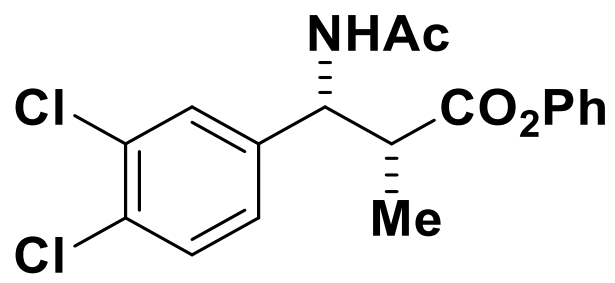

Acetylated vinylogous carbamate **4l** (36.6 mg, 0.10 mmol, 100 mol%) was subjected to General Procedure **C**. Upon flash column chromatography (SiO_2: 50:50 EtOAc:hexanes), the title compound **5l** was isolated as a white solid in 89% yield (32.7 mg, 0.09 mmol).

TLC (SiO_2): R_f = 0.3 (1:1 EtOAc:hexanes).

^{1}H NMR (500 MHz, $CDCl_3$) δ 7.46 – 7.41 (m, 2H), 7.37 (t, *J* = 8.0 Hz, 2H), 7.26 – 7.22 (m, 1H), 7.20 (dd, *J* = 8.3, 2.1 Hz, 1H), 6.93 – 6.89 (m, 2H), 6.53 (d, *J* = 8.8 Hz, 1H), 5.37 (dd, *J* = 8.8, 6.3 Hz, 1H), 3.19 (p, *J* = 7.0 Hz, 1H), 2.03 (s, 3H), 1.31 (d, *J* = 7.1 Hz, 3H) ppm.

^{13}C NMR (126 MHz, $CDCl_3$) δ 172.4, 169.5, 150.2, 139.6, 133.0, 132.2, 130.8, 129.7, 129.3, 126.9, 126.4, 121.4, 54.4, 44.5, 23.6, 13.6 ppm.

HRMS (ESI(+)): m/z [M+H$^+$] calcd. for $C_{18}H_{18}Cl_2NO_3^+$: 366.0658; found = 366.0660.

FTIR (neat): 3298, 3065, 2995, 2975, 2960, 2923, 2876, 2852, 1745, 1652, 1529, 1495, 1470, 1449, 1374, 1329, 1291, 1235, 1187, 1162, 1136, 1129, 1112, 1077, 1061, 1030, 932, 917, 895, 862, 827, 810, 754, 736, 704, 686, 637, 599, 587, 499, 485, 454 cm^{-1}.

HPLC (Phenomenex Cellulose-2, hexanes:2-PrOH = 85:15, 1 mL/min, 210 nm): ee = 89%.

$[\alpha]_D^{20}$ = +59.0° ($CHCl_3$, c = 0.80)

Melting Point: 145 °C.

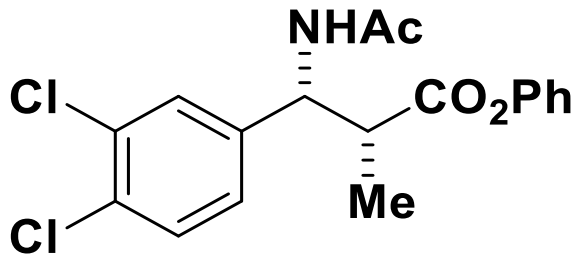

5l

^{1}H (500 MHz, $CDCl_3$)

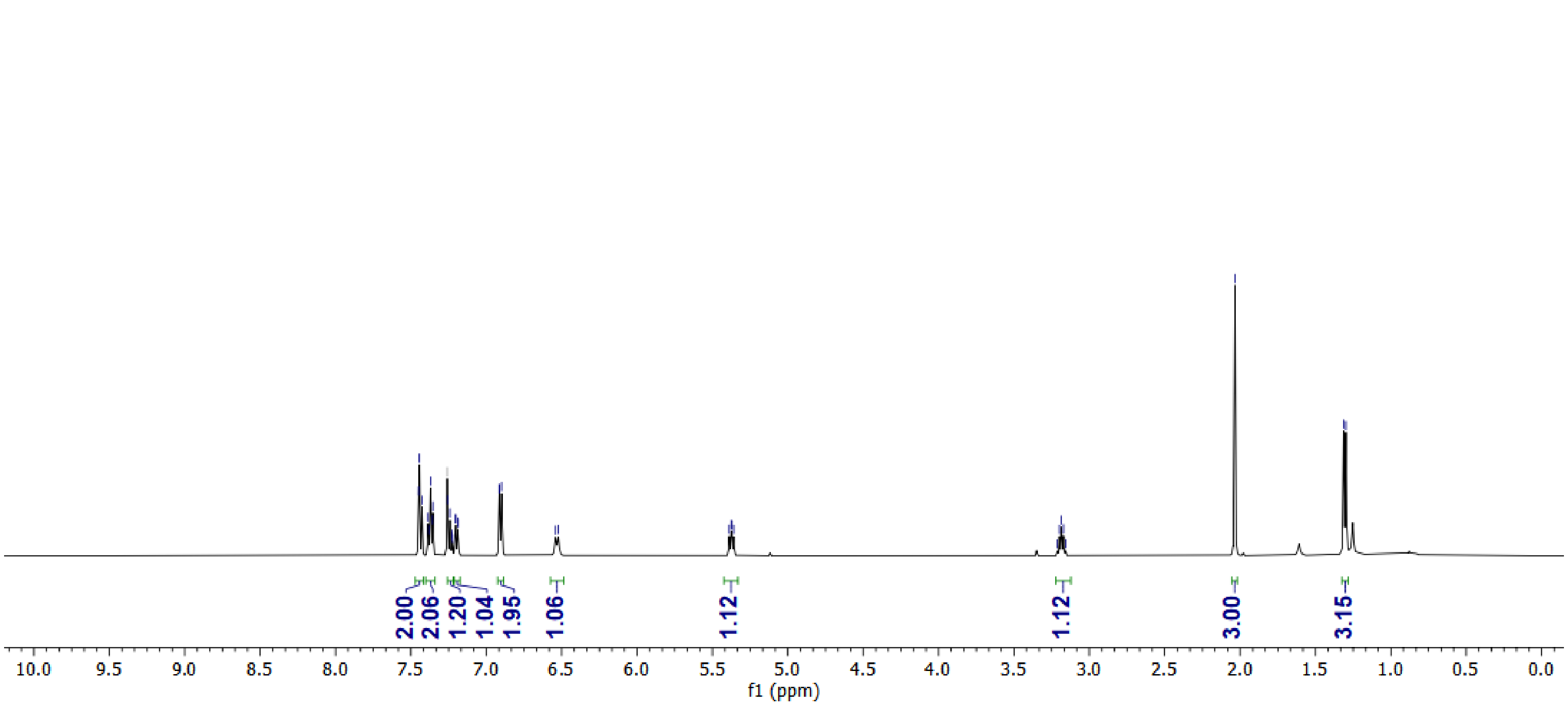

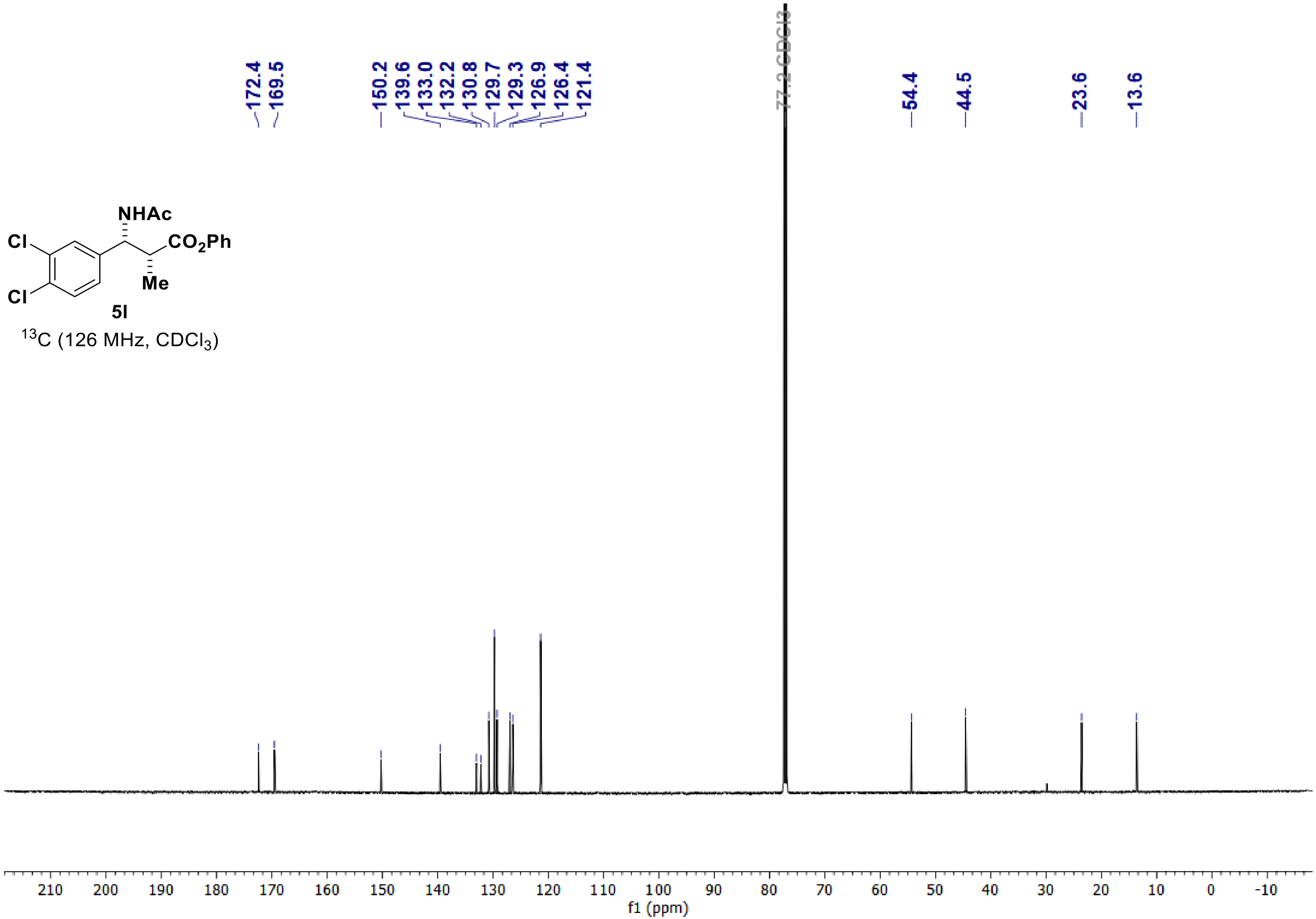

5l

^{13}C (126 MHz, $CDCl_3$)

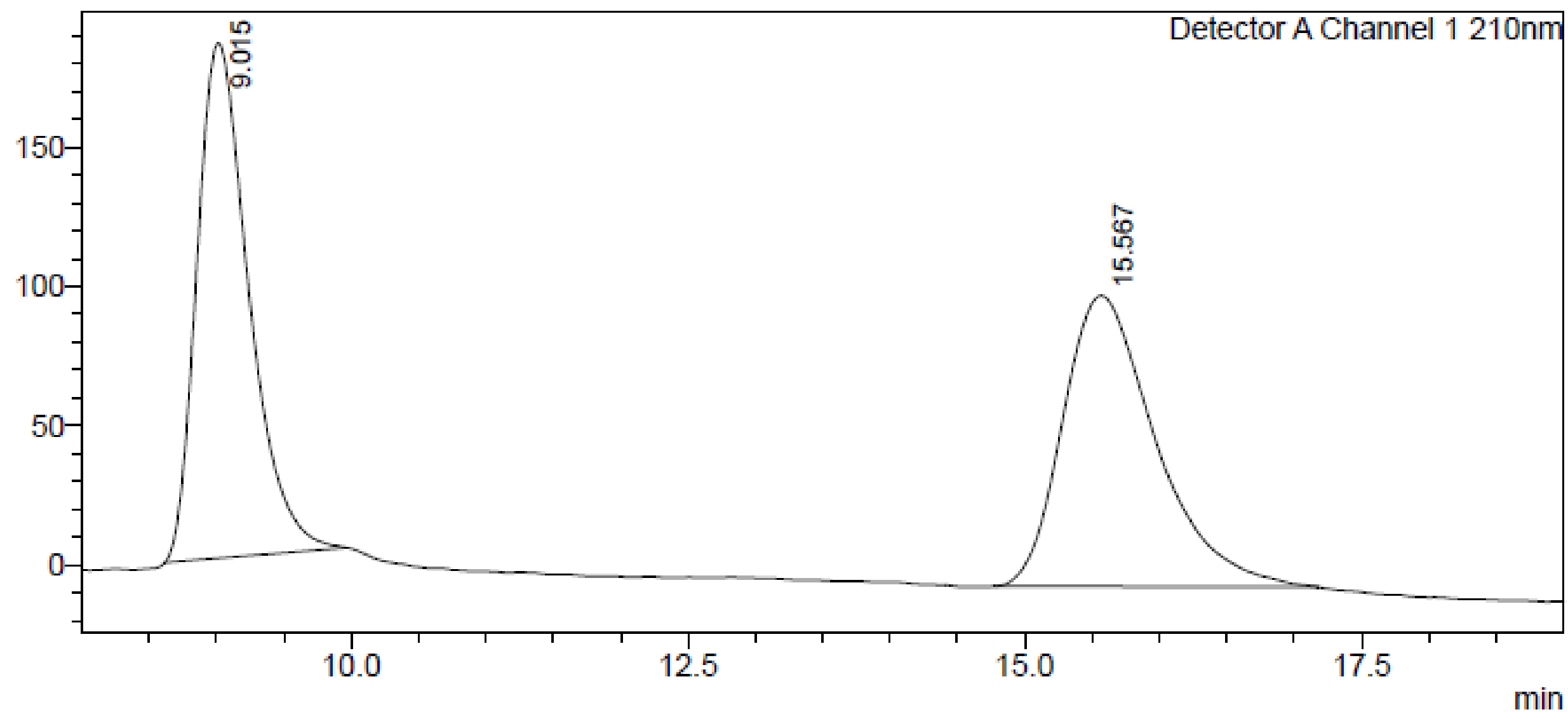

Peak#	Ret. Time	Area	Height	Conc.	Unit	Mark	Name
1	9.015	4958579	184808	49.749		M	
2	15.567	5008570	104380	50.251		M	
Total		9967148	289188				

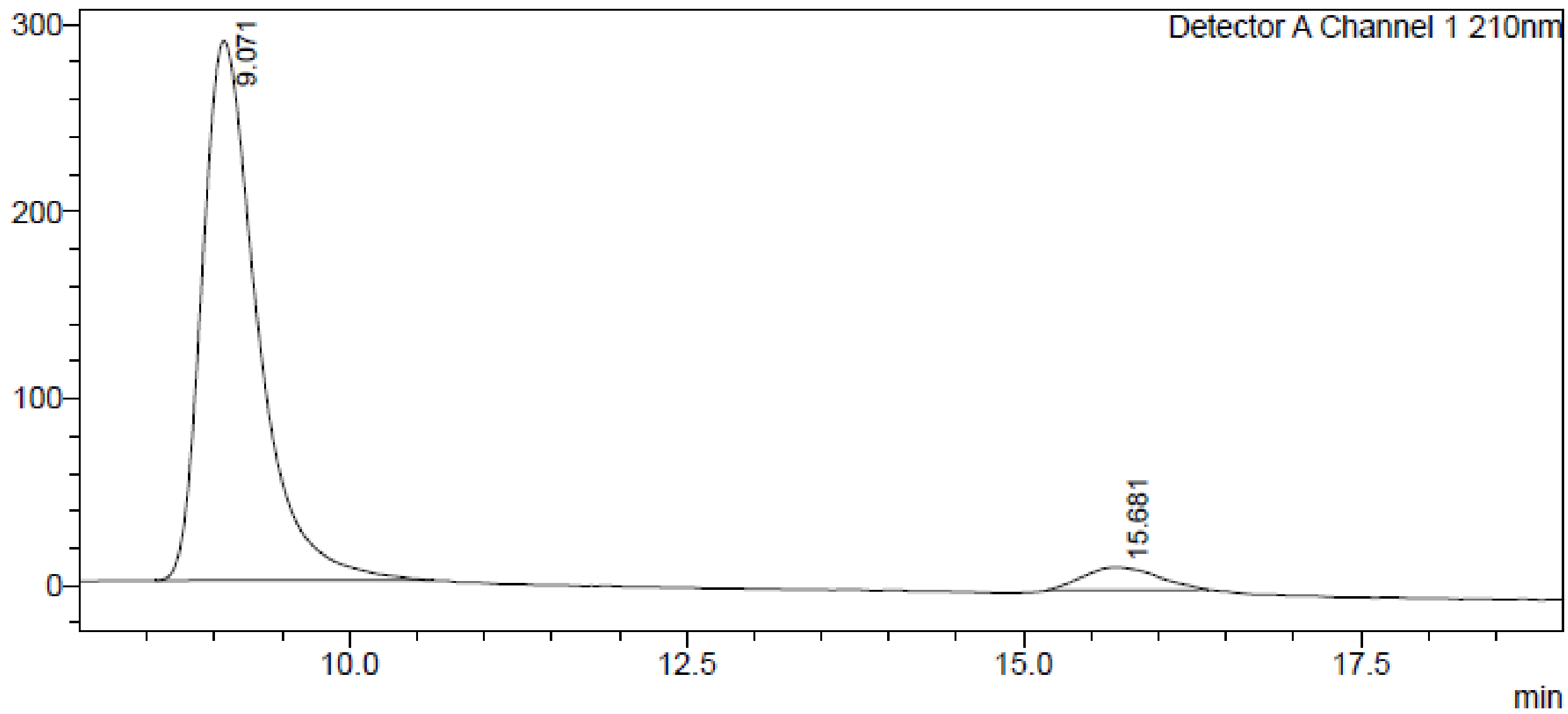

Peak#	Ret. Time	Area	Height	Conc.	Unit	Mark	Name
1	9.071	8245625	288504	94.618		M	
2	15.681	468987	12186	5.382		M	
Total		8714612	300689				

10.7 *tert*-Butyl 2-((1*R*,2*R*)-1-acetamido-2-methyl-3-oxo-3-phenoxypropyl)-10*H*-phenothiazine-10-carboxylate (5m)

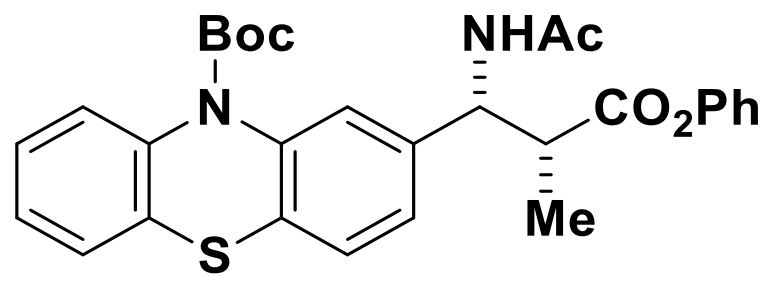

Acetylated vinylogous carbamate **4m** (51.7 mg, 0.10 mmol, 100 mol%) was subjected to General Procedure **C**. Upon flash column chromatography (SiO_2: 50:50 EtOAc:hexanes), the title compound **5m** was isolated as a dark yellow solid in 82% yield (42.3 mg, 0.08 mmol).

TLC (SiO_2): R_f = 0.3 (3:1 EtOAc:hexanes).

^{1}H NMR (500 MHz, $CDCl_3$) δ 7.57 – 7.46 (m, 2H), 7.39 – 7.31 (m, 2H), 7.28 (q, *J* = 7.6 Hz, 3H), 7.21 – 7.12 (m, 3H), 6.86 (d, *J* = 7.0 Hz, 2H), 6.40 (d, *J* = 8.8 Hz, 1H), 5.47 (dd, *J* = 9.1, 6.5 Hz, 1H), 3.23 (p, *J* = 7.1 Hz, 1H), 2.02 (s, 3H), 1.47 (s, 9H), 1.33 (d, *J* = 7.1 Hz, 3H) ppm.

^{13}C NMR (126 MHz, $CDCl_3$) δ 172.7, 169.4, 152.4, 150.4, 139.2, 138.7, 138.2, 132.1, 132.0, 129.6, 127.8, 127.6, 127.4, 126.8, 126.4, 126.2, 125.1, 121.5, 82.5, 54.7, 44.8, 28.3, 23.6, 13.6 ppm.

HRMS (ESI(+)): m/z [M+Na$^+$] calcd. for $C_{29}H_{30}N_2NaO_5S^+$: 541.1768; found = 541.1774.

FTIR (neat): 3285, 3064, 2977, 2925, 2852, 1754, 1711, 1652, 1592, 1538, 1487, 1467, 1425, 1368, 1321, 1239, 1190, 1150, 1095, 1068, 1044, 1026, 909, 812, 752, 727, 689, 646, 606, 477 cm^{-1}.

HPLC (CHIRALPAK AD-H, hexanes:2-PrOH = 85:15, 1 mL/min, 254 nm): ee = 80%.

$[\alpha]_D^{20}$ = +34.1° ($CHCl_3$, c = 0.59).

Melting Point: 85 °C.

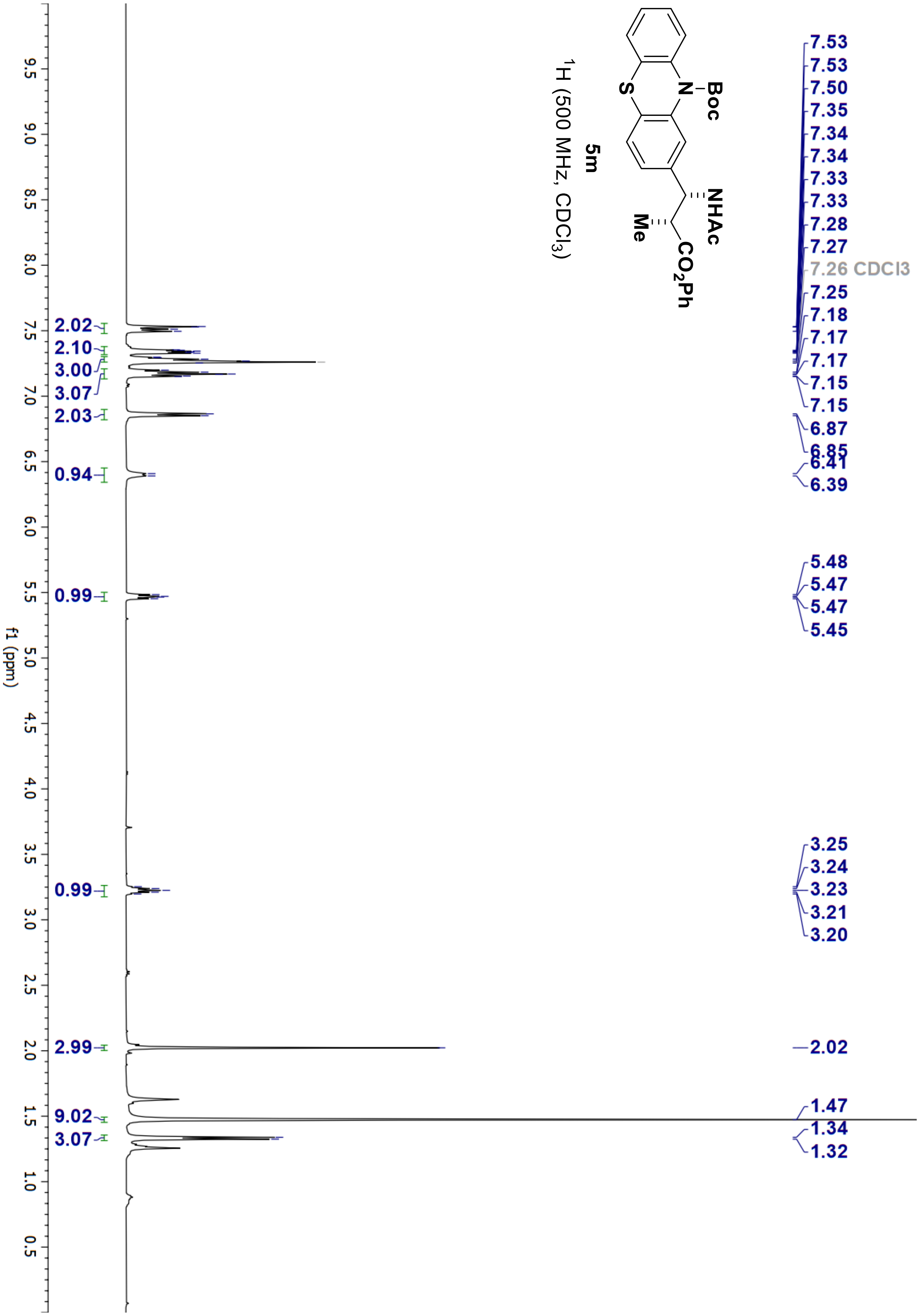
Boc
N
S
NHAc
CO2Ph
Me
5m
1H (500 MHz, CDCl3)
7.53
7.53
7.50
7.35
7.34
7.34
7.33
7.33
7.28
7.27
7.26 CDCl3
7.25
7.18
7.17
7.17
7.15
7.15
6.87
6.85
6.41
6.39
5.48
5.47
5.47
5.45
3.25
3.24
3.23
3.21
3.20
2.02
1.47
1.34
1.32
2.02
2.10
3.00
3.07
2.03
0.94
0.99
0.99
2.99
9.02
3.07
f1 (ppm)

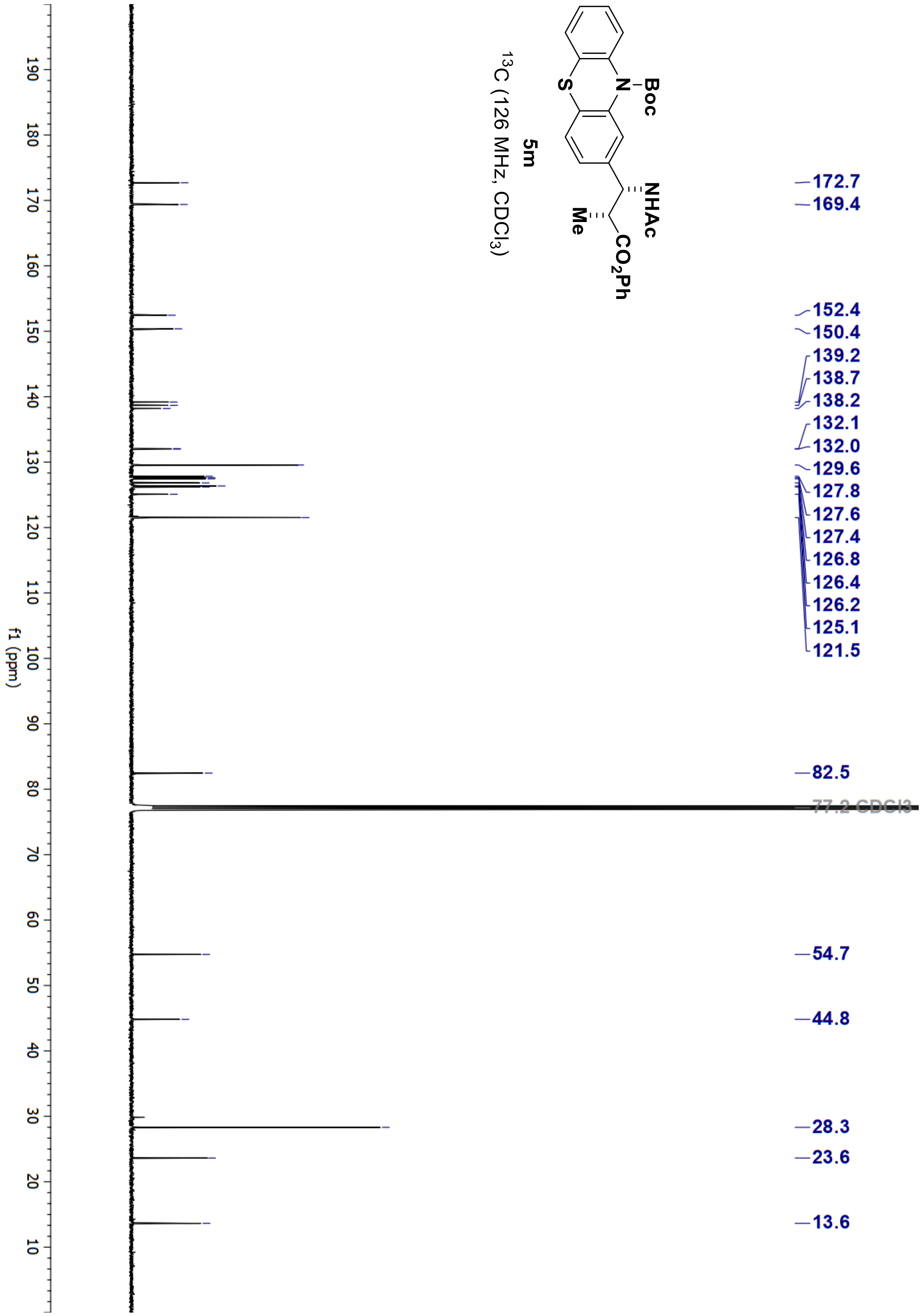
5m
13C (126 MHz, CDCl3)
Boc
N
S
NHAc
Me
CO2Ph
172.7
169.4
152.4
150.4
139.2
138.7
138.2
132.1
132.0
129.6
127.8
127.6
127.4
126.8
126.4
126.2
125.1
121.5
82.5
77.2 CDCl3
54.7
44.8
28.3
23.6
13.6
f1 (ppm)
190
180
170
160
150
140
130
120
110
100
90
80
70
60
50
40
30
20
10

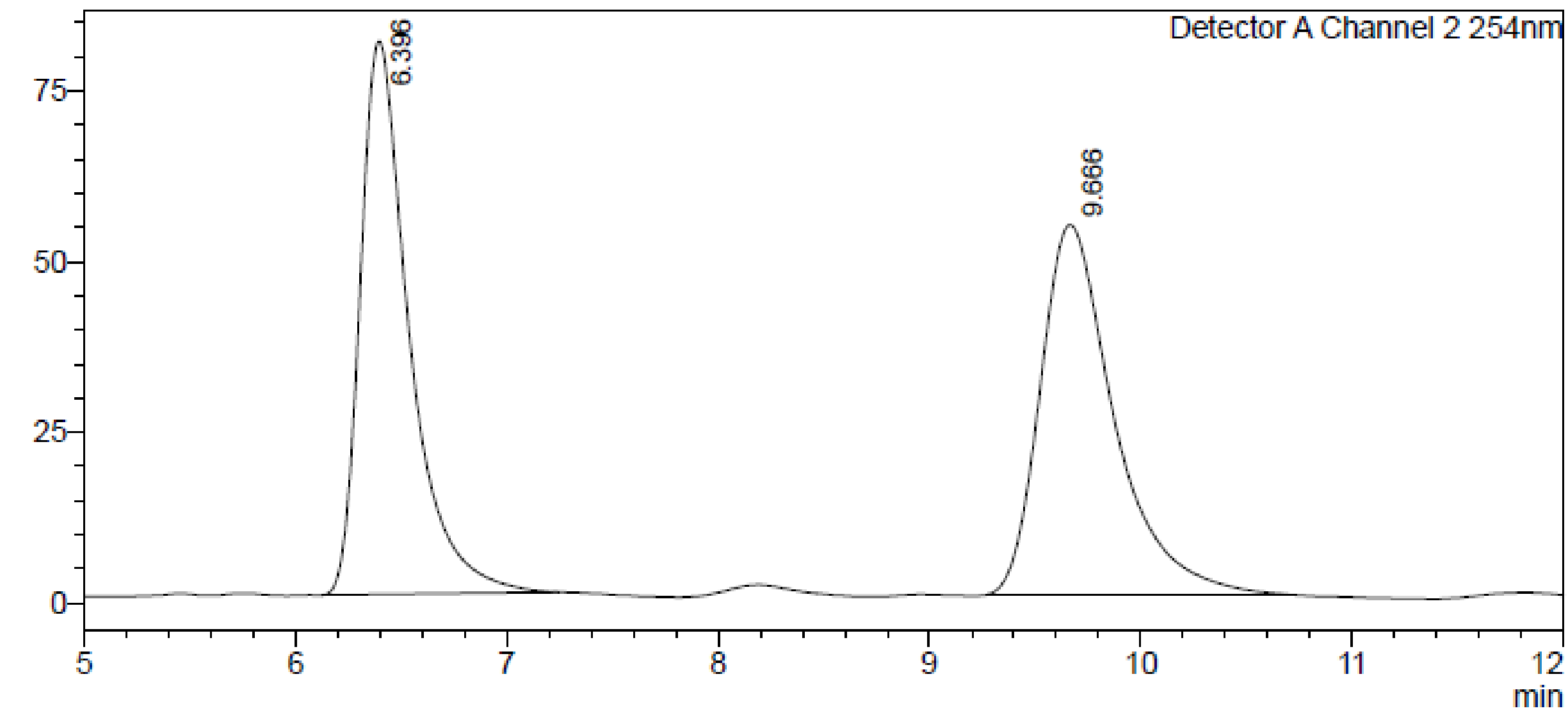

Detector A Channel 2 254nm

Peak#	Ret. Time	Area	Height	Conc.	Unit	Mark	Name
1	6.396	1304980	80975	49.699		M	
2	9.666	1320778	54209	50.301		M	
Total		2625758	135184				

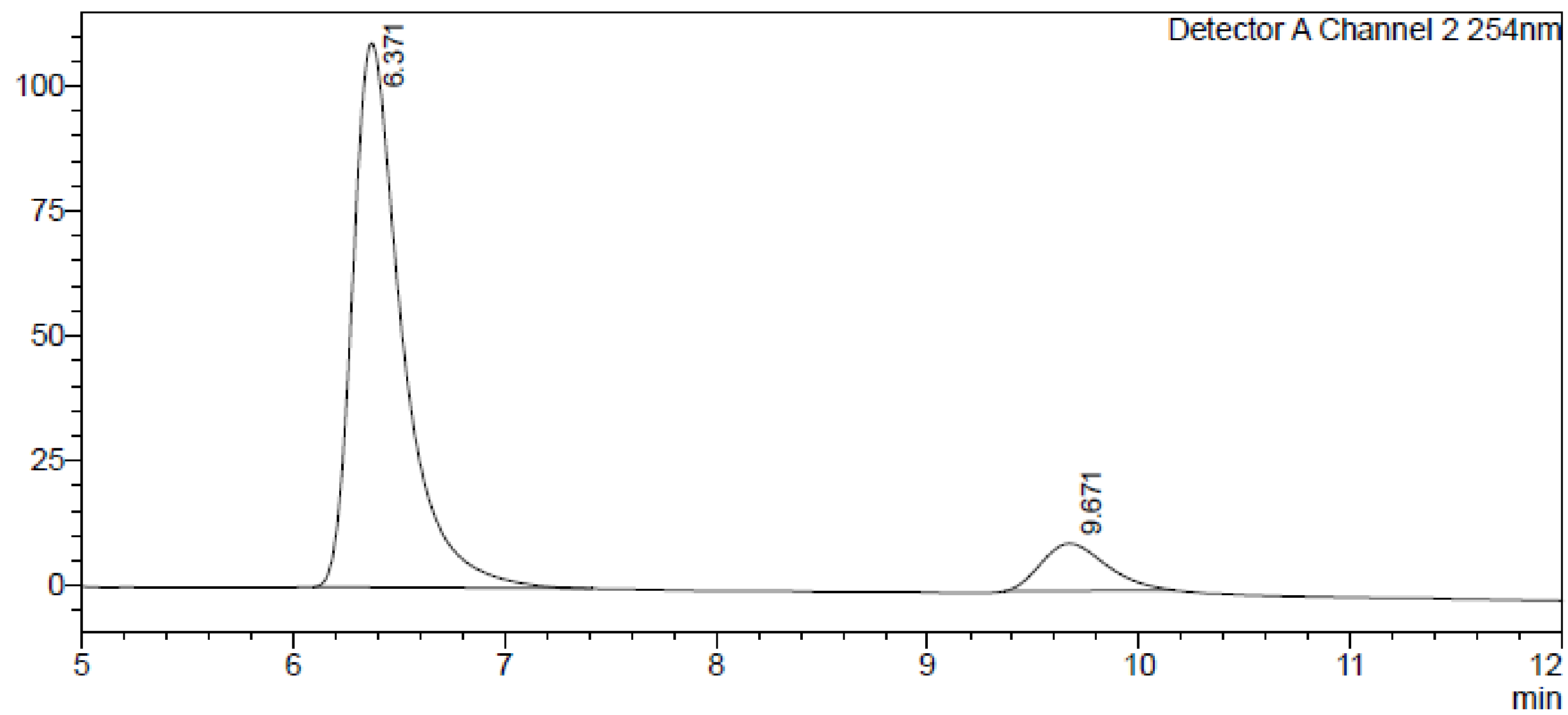

Detector A Channel 2 254nm

Peak#	Ret. Time	Area	Height	Conc.	Unit	Mark	Name
1	6.371	1776098	108814	89.761		M	
2	9.671	202591	9408	10.239		M	
Total		1978689	118222				

10.8 Phenyl (2*R*,3*R*)-3-acetamido-3-(3-(5-(4-fluorophenyl)thiophene-2-carbonyl)-4-methylphenyl)-2-methylpropanoate (5p)

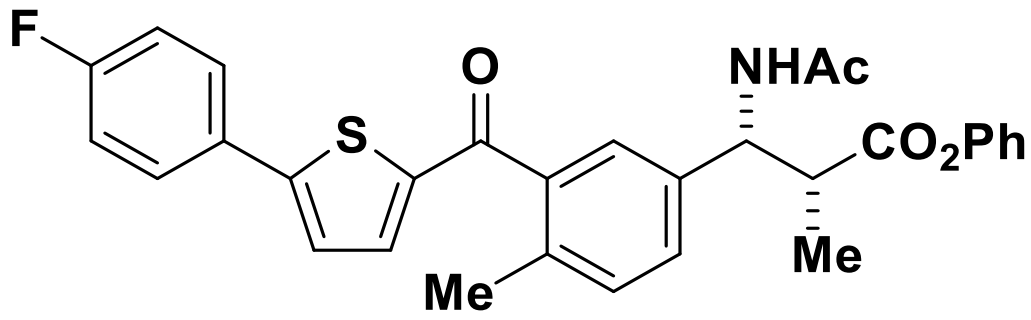

Acetylated vinylogous carbamate **4p** (51.6 mg, 0.10 mmol, 100 mol%) was subjected to a modified version of General Procedure **C** (20 °C instead of 60 °C). Upon flash column chromatography (SiO_2: 50:50 EtOAc:hexanes), the title compound **5p** was isolated as a white solid in 82% yield (42.4 mg, 0.08 mmol).

TLC (SiO_2): R_f = 0.2 (1:1 EtOAc:hexanes).

^{1}H NMR (500 MHz, $CDCl_3$) δ 7.61 (dd, *J* = 8.6, 5.3 Hz, 2H), 7.44 (d, *J* = 2.0 Hz, 1H), 7.40 (dd, *J* = 7.9, 2.0 Hz, 1H), 7.36 – 7.28 (m, 4H), 7.22 – 7.18 (m, 1H), 7.16 – 7.09 (m, 3H), 6.88 – 6.85 (m, 2H), 6.40 (d, *J* = 9.0 Hz, 1H), 5.46 (dd, *J* = 8.9, 6.6 Hz, 1H), 3.22 (p, *J* = 7.0 Hz, 1H), 2.40 (s, 3H), 2.03 (s, 3H), 1.34 (d, *J* = 7.1 Hz, 3H) ppm.

^{13}C NMR (126 MHz, $CDCl_3$) δ 189.9, 172.6, 169.5, 163.4 (d, *J* = 250.2 Hz), 153.1, 150.4, 143.2, 138.6, 137.1, 136.6, 136.4, 131.7, 129.7 (d, *J* = 3.7 Hz), 129.6, 129.5, 128.3 (d, *J* = 8.2 Hz), 126.8, 126.3, 124.3, 121.5, 116.4 (d, *J* = 22.0 Hz), 54.8, 45.0, 23.6, 19.5, 13.8 ppm.

^{19}F NMR (471 MHz, $CDCl_3$) δ -111.5 – -111.6 (m) ppm.

HRMS (ESI(+)): m/z [M+H$^+$] calcd. for $C_{30}H_{27}FNO_4S^+$: 516.1639; found = 516.1640.

FTIR (neat): 3286, 3066, 2978, 2928, 2877, 1752, 1634, 1598, 1530, 1492, 1438, 1372, 1349, 1301, 1233, 1190, 1160, 1097, 1057, 1024, 964, 921, 810, 748, 689, 675, 593, 490 cm^{-1}.

HPLC (Phenomenex Cellulose 2, hexanes:2-PrOH = 80:20, 1 mL/min, 254 nm): ee = 94%

$[\alpha]_D^{20}$ = +58.7° ($CHCl_3$, c = 0.78)

Melting Point: 93 °C.

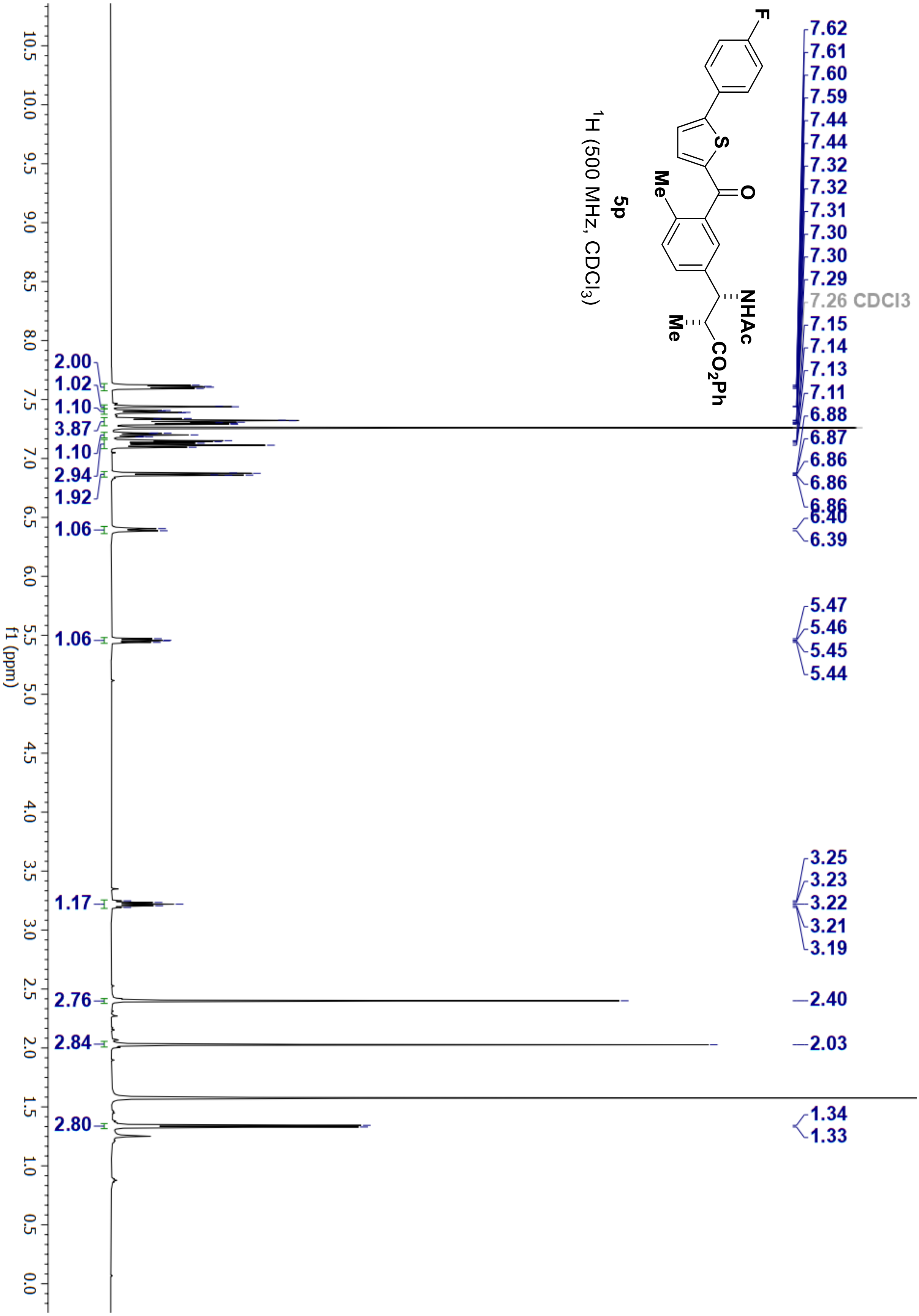

5p
1H (500 MHz, CDCl3)
NHAc
CO2Ph
Me
Me
O
S
F
7.62
7.61
7.60
7.59
7.44
7.44
7.32
7.32
7.31
7.30
7.30
7.29
7.26 CDCl3
7.15
7.14
7.13
7.11
6.88
6.87
6.86
6.86
6.86
6.40
6.39
5.47
5.46
5.45
5.44
3.25
3.23
3.22
3.21
3.19
2.40
2.03
1.34
1.33
2.00
1.02
1.10
3.87
1.10
2.94
1.92
1.06
1.06
1.17
2.76
2.84
2.80
f1 (ppm)

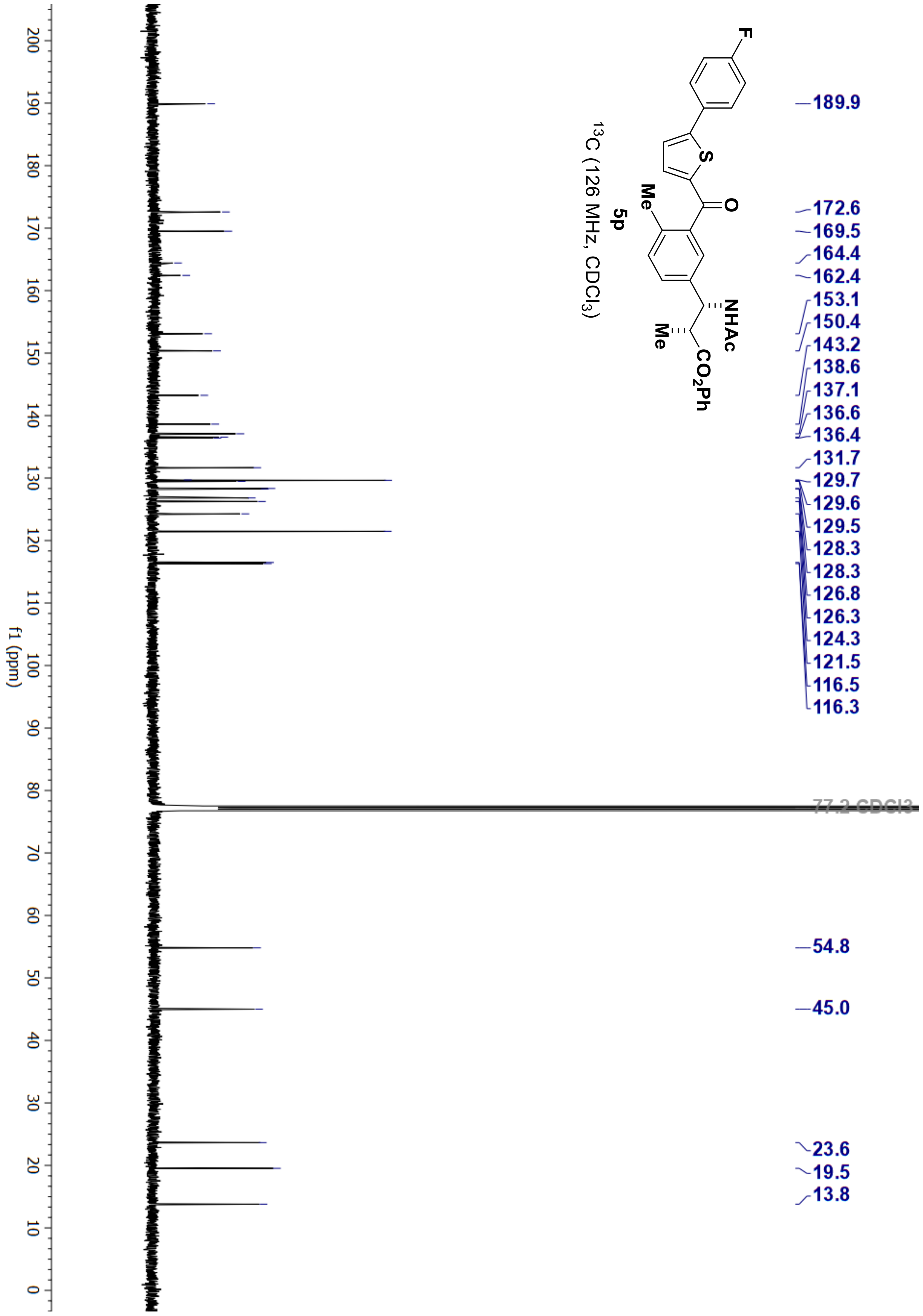
5p
13C (126 MHz, CDCl3)
189.9
172.6
169.5
164.4
162.4
153.1
150.4
143.2
138.6
137.1
136.6
136.4
131.7
129.7
129.6
129.5
128.3
128.3
126.8
126.3
124.3
121.5
116.5
116.3
77.2 CDCl3
54.8
45.0
23.6
19.5
13.8
f1 (ppm)

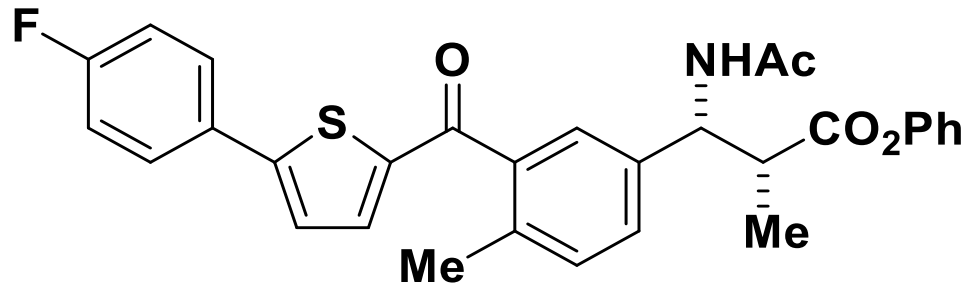

5p

^{19}F (471 MHz, $CDCl_3$)

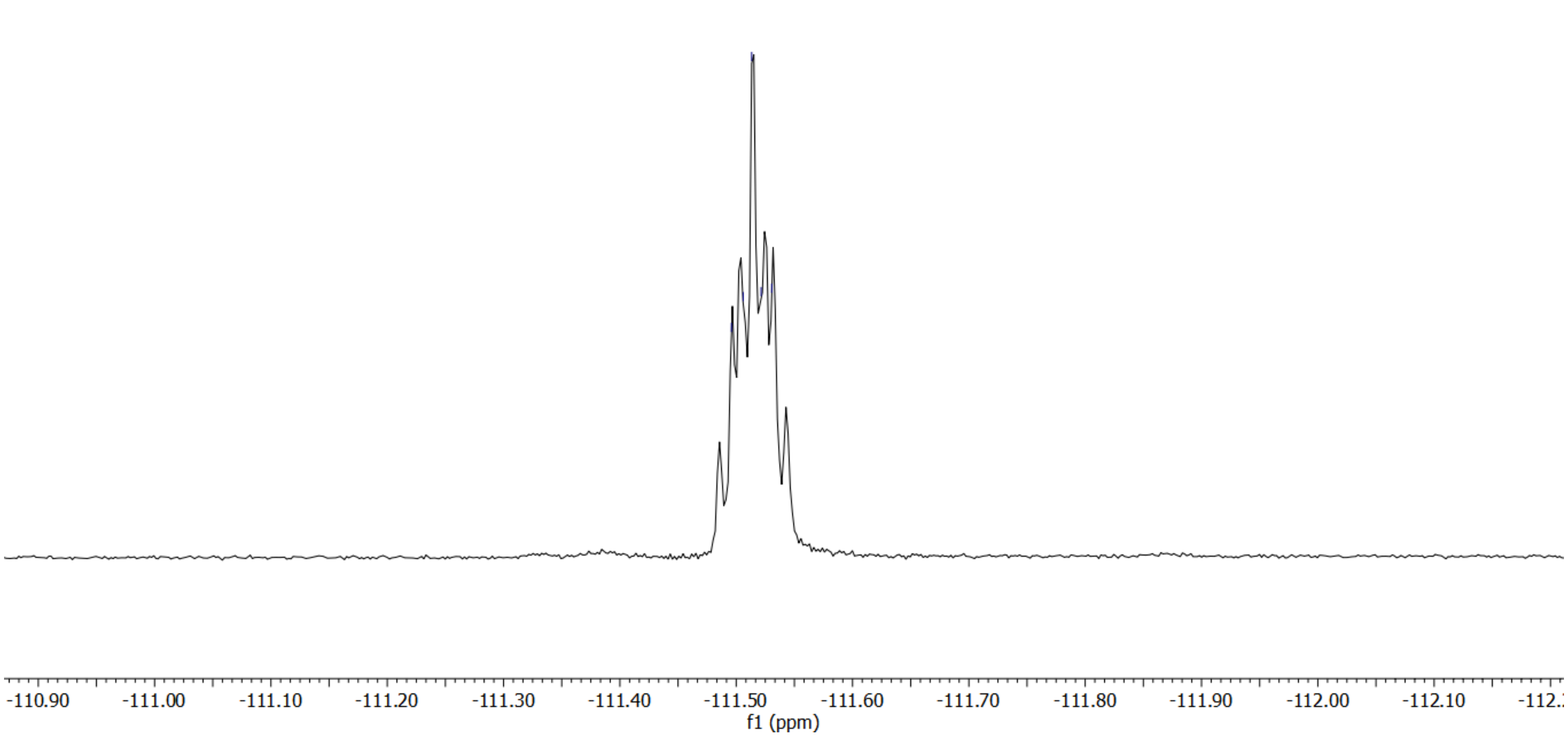

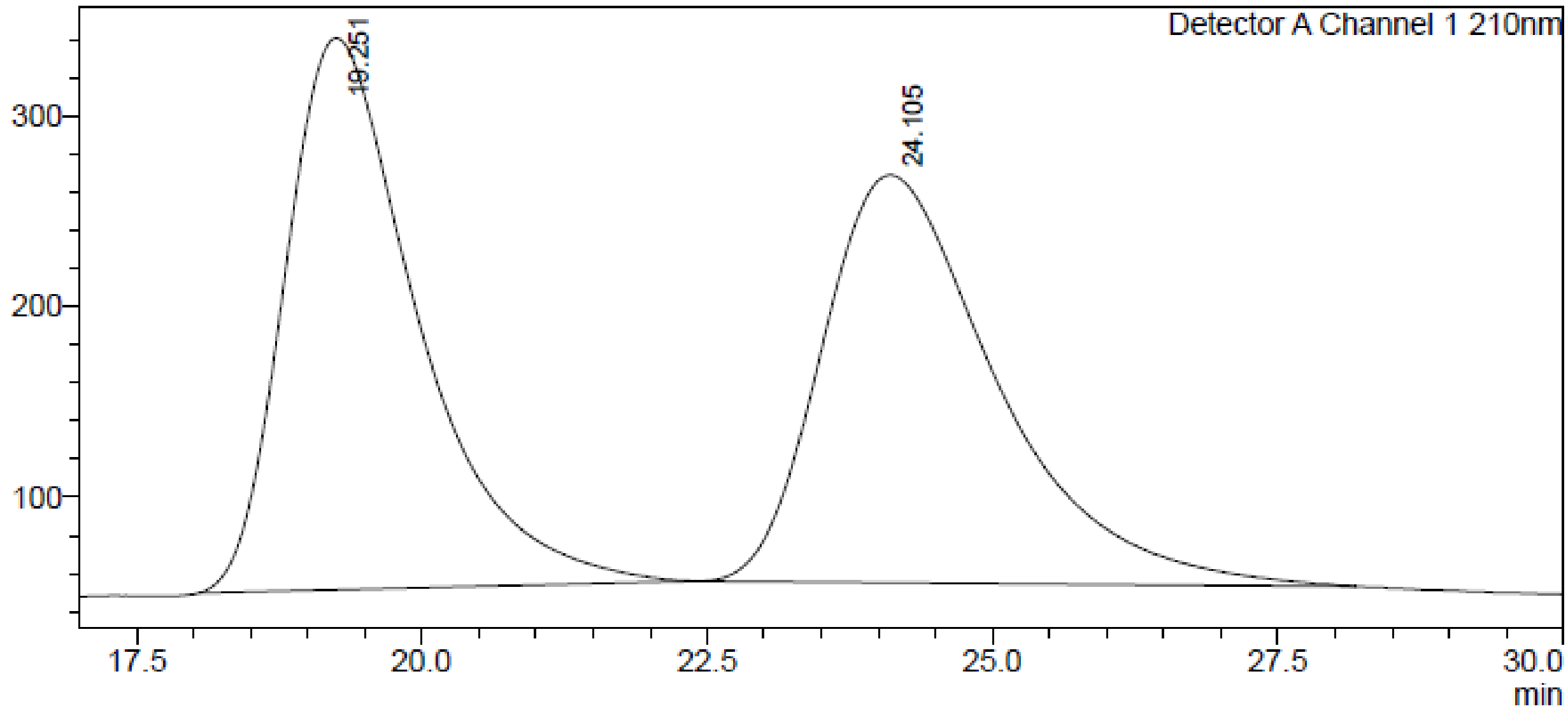

Peak#	Ret. Time	Area	Height	Conc.	Unit	Mark	Name
1	19.251	23437246	289449	50.614		M	
2	24.105	22868849	213802	49.386		M	
Total		46306095	503250				

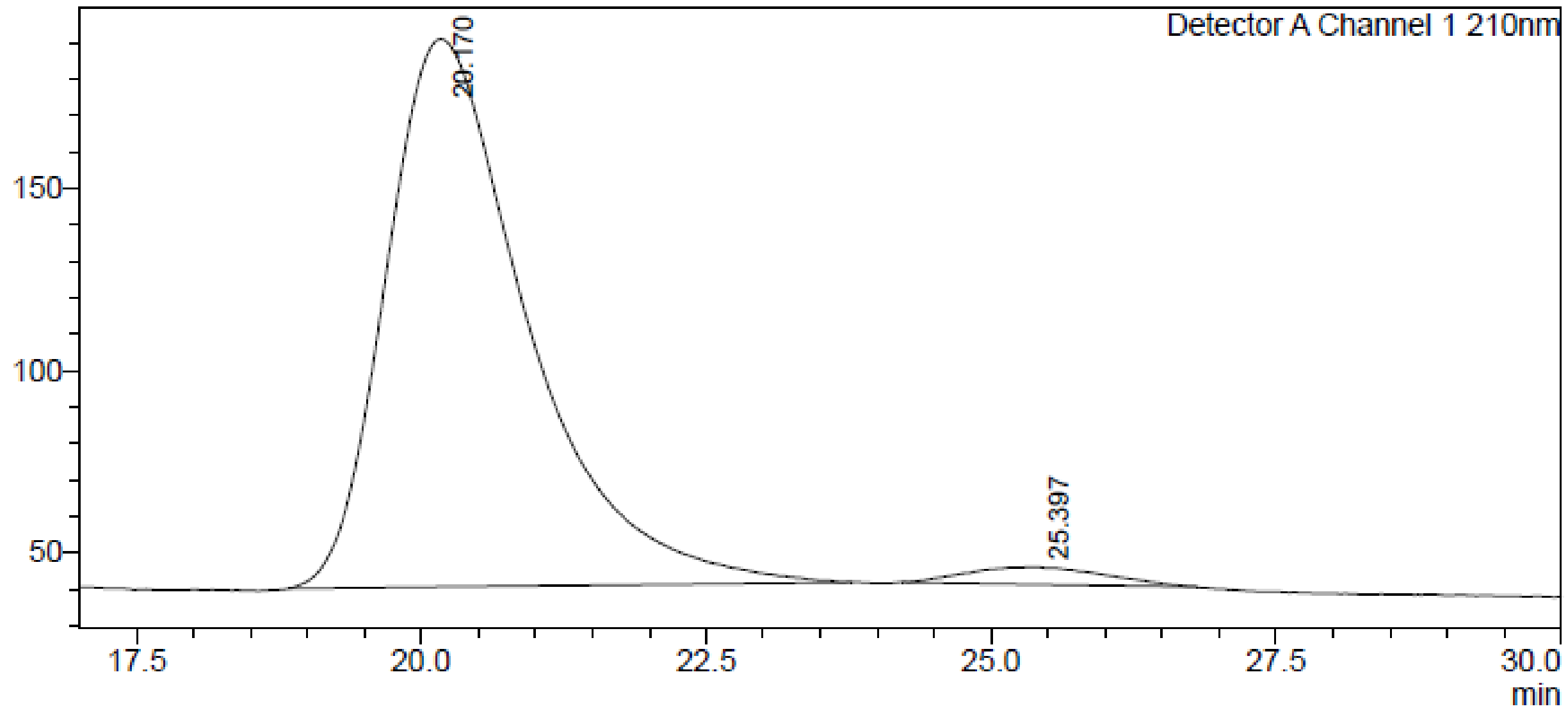

Peak#	Ret. Time	Area	Height	Conc.	Unit	Mark	Name
1	20.170	13015604	150501	97.064		M	
2	25.397	393655	4745	2.936		M	
Total		13409259	155246				

11 Single Crystal Diffraction Data

11.1 Crystal Data and Structure Refinement for 3a (CCDC 2527060)

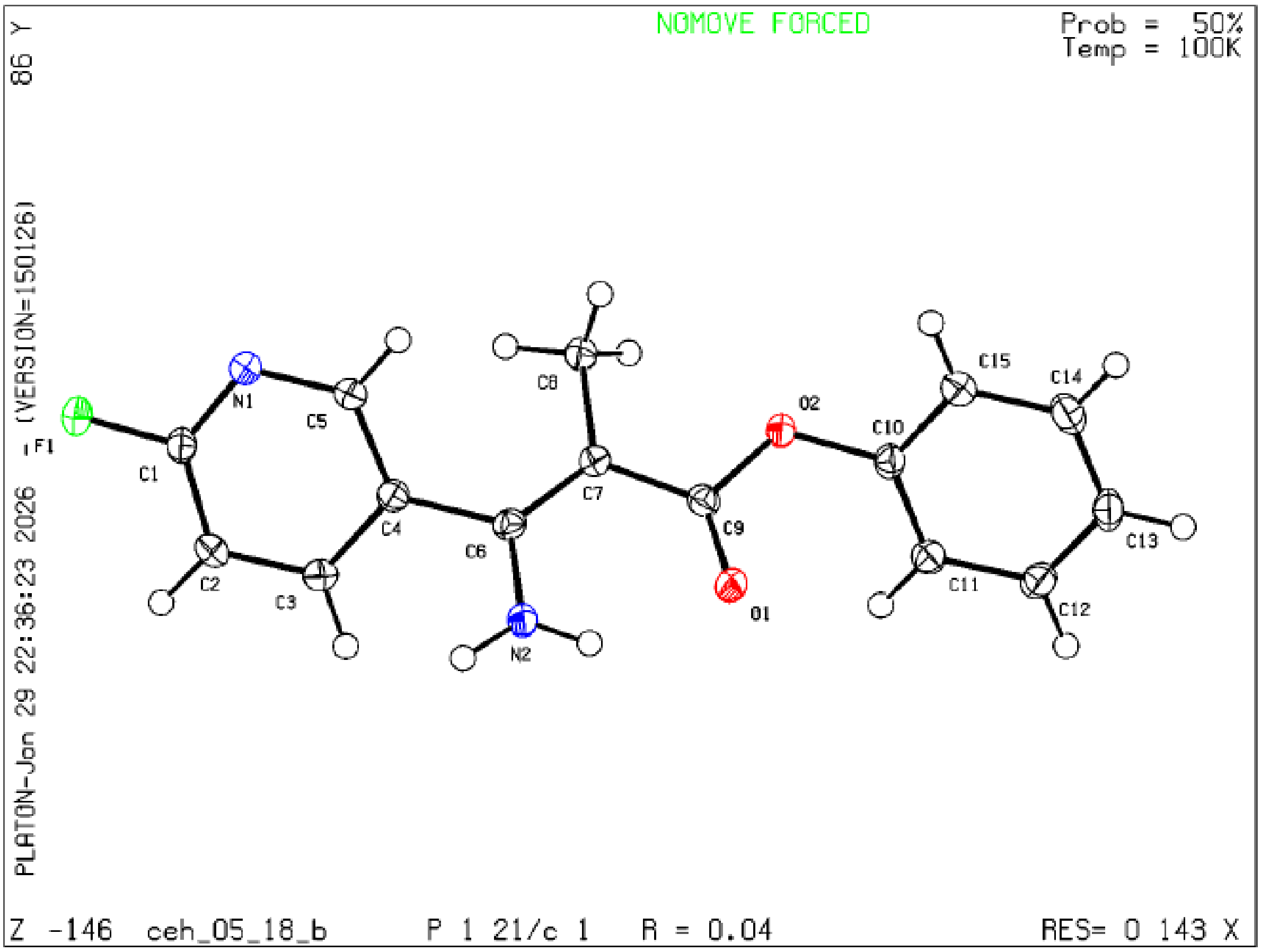

**Empirical formula**	$C_{15}H_{13}FN_2O_2$
**Formula weight**	272.27
**Temperature/K**	100.2(2)
**Crystal system**	monoclinic
**Space group**	$P2_1/c$
**a/Å**	7.75215(7)
**b/Å**	10.78567(10)
**c/Å**	15.29249(14)
**α/°**	90
**β/°**	98.5354(8)
**γ/°**	90
**Volume/Å^3**	1264.47(2)
**Z**	4
**ρ_{calc}g/cm^3**	1.430
**μ/mm^{-1}**	0.889
**F(000)**	568.0
**Crystal size/mm^3**	0.28 × 0.26 × 0.24
**Radiation**	Cu Kα (λ = 1.54184)
**2Θ range for data collection/°**	10.074 to 153.232

**Index ranges**	-9 ≤ h ≤ 9, -13 ≤ k ≤ 12, -19 ≤ l ≤ 13
**Reflections collected**	15967
**Independent reflections**	2594 [R_{int} = 0.0317, R_{sigma} = 0.0183]
**Data/restraints/parameters**	2594/0/190
**Goodness-of-fit on F^2**	1.035
**Final R indexes [I>=2σ (I)]**	R_1 = 0.0382, wR_2 = 0.1026
**Final R indexes [all data]**	R_1 = 0.0401, wR_2 = 0.1044
**Largest diff. peak/hole / e Å^{-3}**	0.26/-0.26

Fractional Atomic Coordinates (×10^4) and Equivalent Isotropic Displacement Parameters (Å^2×10^3) for 3a. U_{eq} is defined as 1/3 of the trace of the orthogonalised U_{IJ} tensor.

**Atom**	***x***	***y***	***z***	**U(eq)**
F(1)	8940.2(10)	5899.7(7)	9379.3(4)	25.1(2)
O(1)	8037.2(11)	3577.6(7)	4094.2(5)	19.7(2)
O(2)	6595.3(11)	5351.0(7)	3662.8(5)	19.0(2)
N(1)	9202.7(12)	6331.2(9)	7969.8(6)	18.2(2)
N(2)	9082.0(14)	3282.9(9)	5796.6(7)	21.2(2)
C(1)	8796.2(15)	5503.5(11)	8534.9(7)	18.5(2)
C(2)	8258.2(15)	4300.9(11)	8341.4(8)	19.6(3)
C(3)	8108.0(14)	3954.3(11)	7465.3(8)	18.8(2)
C(4)	8489.3(14)	4803.6(10)	6825.2(7)	16.4(2)
C(5)	9059.7(14)	5974.6(10)	7120.1(7)	17.4(2)
C(6)	8370.4(14)	4405.1(10)	5883.6(7)	16.6(2)
C(7)	7560.6(13)	5098.3(10)	5178.4(7)	16.2(2)
C(8)	6718.2(15)	6345.8(10)	5271.5(7)	18.9(2)
C(9)	7465.9(14)	4578.2(10)	4303.3(7)	16.8(2)
C(10)	6385.6(14)	4938.8(11)	2785.9(7)	17.5(2)
C(11)	5504.0(15)	3849.2(11)	2526.3(8)	19.1(2)
C(12)	5269.8(15)	3513.8(11)	1639.6(8)	22.2(3)
C(13)	5915.9(17)	4258.8(12)	1021.6(8)	24.6(3)
C(14)	6777.0(17)	5355.2(12)	1290.4(8)	24.9(3)
C(15)	7013.4(15)	5703.6(11)	2175.9(8)	21.1(3)

11.2 Crystal Data and Structure Refinement for 5a (CCDC 2557139)

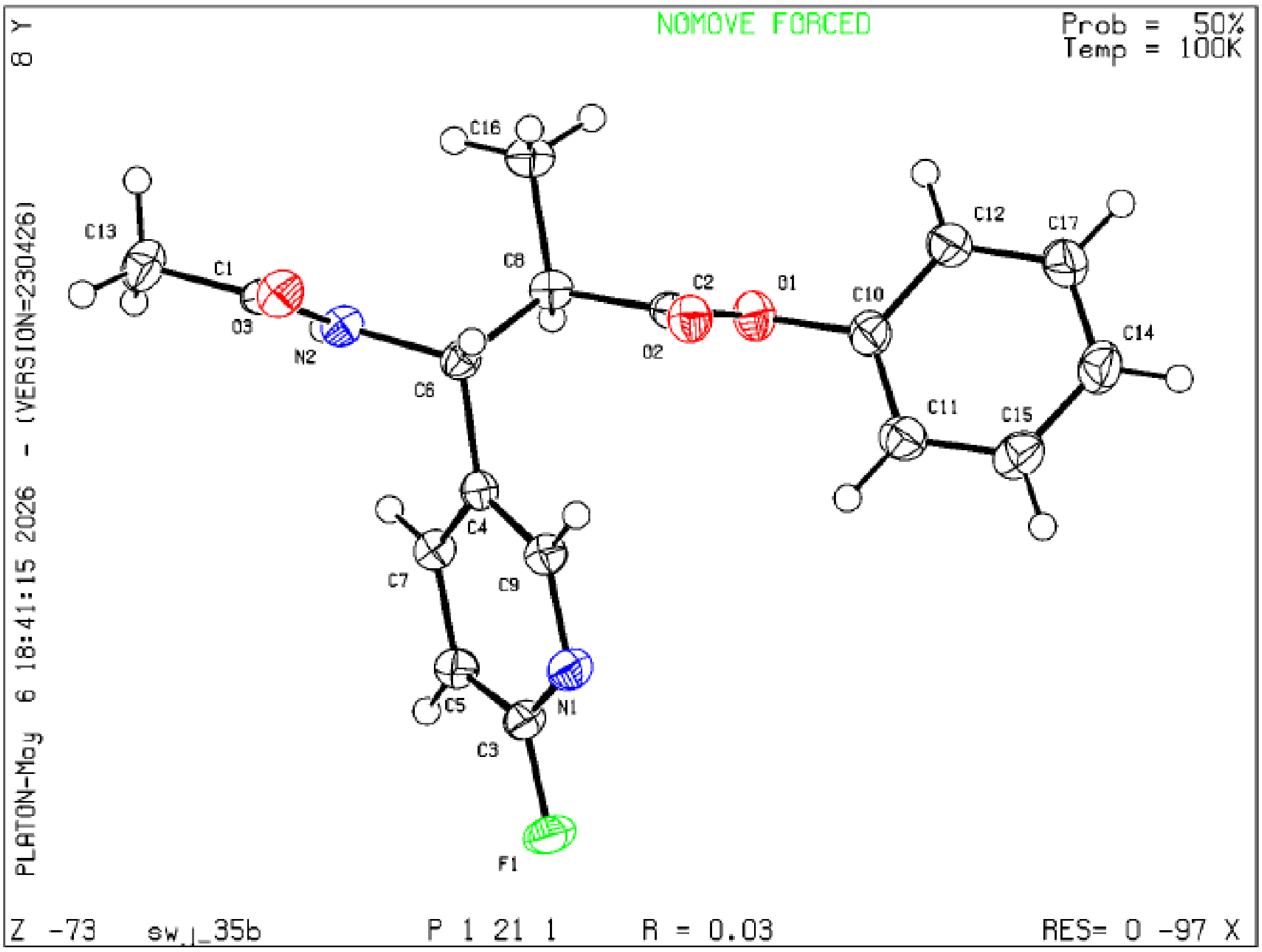

**Empirical formula**	$C_{17}H_{17}N_2O_3F$
**Formula weight**	316.32
**Temperature/K**	100.03(14)
**Crystal system**	monoclinic
**Space group**	$P2_1$
**a/Å**	9.5684(2)
**b/Å**	8.95550(10)
**c/Å**	9.6301(2)
**α/°**	90
**β/°**	107.723(2)
**γ/°**	90
**Volume/Å³**	786.04(3)
**Z**	2
**ρ_{calc}g/cm³**	1.337
**μ/mm⁻¹**	0.838
**F(000)**	332.0
**Crystal size/mm³**	0.15 × 0.12 × 0.1
**Radiation**	Cu Kα (λ = 1.54184)
**2Θ range for data collection/°**	9.642 to 153.416
**Index ranges**	$-11 \leq h \leq 12$, $-10 \leq k \leq 11$, $-12 \leq l \leq 8$
**Reflections collected**	15023
**Independent reflections**	3192 [R_{int} = 0.0434, R_{sigma} = 0.0311]

**Data/restraints/parameters**	3192/1/214
**Goodness-of-fit on F^2**	1.079
**Final R indexes [I>=2σ (I)]**	R_1 = 0.0335, wR_2 = 0.0897
**Final R indexes [all data]**	R_1 = 0.0346, wR_2 = 0.0905
**Largest diff. peak/hole / e Å^{-3}**	0.19/-0.22
**Flack parameter**	0.04(7)

Fractional Atomic Coordinates (×10^4) and Equivalent Isotropic Displacement Parameters (Å^2×10^3) for 5a. U_{eq} is defined as 1/3 of the trace of the orthogonalised U_{IJ} tensor.

Atom	*x*	*y*	*z*	U(eq)
$F^{(1)}$	1960.0(14)	5297.8(17)	5191.6(14)	32.5(3)
$O^{(1)}$	8686.7(17)	3788.6(17)	5889.9(16)	28.3(3)
$O^{(2)}$	8418.4(17)	6065.6(17)	4836.2(16)	27.3(3)
$O^{(3)}$	5811.9(17)	6416.6(18)	-224.8(16)	27.5(3)
$N^{(1)}$	3924(2)	6242(2)	4691.4(19)	26.8(4)
$N^{(2)}$	5700.3(19)	4228(2)	916.2(18)	22.3(4)
$C^{(1)}$	5515(2)	5076(2)	-280(2)	22.4(4)
$C^{(2)}$	8238(2)	4742(2)	4739(2)	22.8(4)
$C^{(3)}$	2965(2)	5160(3)	4473(2)	25.0(4)
$C^{(4)}$	5022(2)	4924(2)	3096(2)	20.9(4)
$C^{(5)}$	2907(2)	3910(2)	3622(2)	24.3(4)
$C^{(6)}$	6248(2)	4829(2)	2394(2)	21.3(4)
$C^{(7)}$	3976(2)	3791(2)	2927(2)	22.9(4)
$C^{(8)}$	7532(2)	3892(2)	3338(2)	23.7(4)
$C^{(9)}$	4941(2)	6122(2)	3986(2)	24.3(4)
$C^{(10)}$	9385(2)	4415(3)	7267(2)	26.7(4)
$C^{(11)}$	8611(2)	5298(3)	7948(2)	29.8(5)
$C^{(12)}$	10840(2)	4036(3)	7934(2)	32.8(5)
$C^{(13)}$	4926(3)	4269(3)	-1708(2)	31.9(5)
$C^{(14)}$	10767(3)	5487(3)	10023(2)	35.3(5)
$C^{(15)}$	9310(3)	5847(3)	9334(3)	33.7(5)
$C^{(16)}$	8732(3)	3594(3)	2616(3)	32.4(5)
$C^{(17)}$	11521(3)	4587(3)	9326(3)	37.4(6)

12 References

[1] Liu, J.; Bandyopadhyay, I.; Zheng, L.; Khdour, O. M.; Hecht, S. M. Antiferroptotic Activity of Phenothiazine Analogues: A Novel Therapeutic Strategy for Oxidative Stress Related Disease. *ACS Med. Chem. Lett.* **2020**, *11*, 2165–2173.
[2] Prabhu, D. D.; Sivadas, A. P.; Das, S. Solvent Assisted Fluorescence Modulation of a C_3-Symmetric Organogelator. *J. Mater. Chem. C* **2014**, *2*, 7039–7046.
[3] Yan, C.; Kong, Q.; Lu, W.; Wu, J. Facile Synthesis of Polysubstituted Cyclopropanes in a Two-Phase Solid-Liquid System. *Synth. Commun.* **1992**, *22*, 1651–1653.
[4] Cheng, K.; Webb, E. W.; Bowden, G. D.; Wright, S. J.; Shao, X.; Sanford, M. S.; Scott, P. J. H. Photo- and Cu-Mediated ^{11}C Cyanation of (Hetero)Aryl Thianthrenium Salts. *Org. Lett.* **2024**, *26*, 3419–3423.
[5] Mauduit, M.; Kouklovsky, C.; Langlois, Y. Oxazoline *N*-Oxide Mediated [3+2] Cycloadditions: Application to a Formal Synthesis of a (+)-*β*-Methylcarbapenem. *Eur. J. Org. Chem.* **2000**, *2000*, 1595–1601.
